# Wacker Oxidation of Methylenecyclobutanes: Scope and Selectivity in an Unusual Setting

**DOI:** 10.1002/anie.202215381

**Published:** 2023-01-12

**Authors:** Jan Sietmann, Marius Tenberge, Johannes M. Wahl

**Affiliations:** ^1^ Organisch-Chemisches Institut Westfälische Wilhelms-Universität Corrensstraße 36 48149 Münster Germany; ^2^ Department Chemie Johannes Gutenberg-Universität Duesbergweg 10–14 55128 Mainz Germany

**Keywords:** Desymmetrization, Strained Rings, 1,2-Rearrangment, Wacker Oxidation

## Abstract

Methylenecyclobutanes are found to undergo Wacker oxidation via a semi‐pinacol‐type rearrangement. Key to a successful process is the use of tert‐butyl nitrite as oxidant, which not only enables efficient catalyst turn‐over but also ensures high Markovnikov‐selectivity under mild conditions. Thus, cyclopentanones (26 examples) can be accessed in an overall good yield and excellent selectivity (up to 97 % yield, generally >99 : 1 ketone:aldehyde ratio). Stereochemical analysis of the reaction sequence reveals migration aptitudes in line with related 1,2‐shifts. By introducing a pyox ligand to palladium, prochiral methylenecyclobutanes can be desymmetrized, thus realizing the first enantioselective Wacker oxidation.

## Graphical abstract

Methylenecyclobutanes undergo Wacker oxidation towards cyclopentanones under mild reaction conditions. A semi‐pinacol‐type rearrangement is proposed to be responsible for the intermediary 1,2‐shift, which not only explains the formation of the products, but also rationalizes the reaction outcomes in terms of site‐, regio‐ and enantioselectivity.

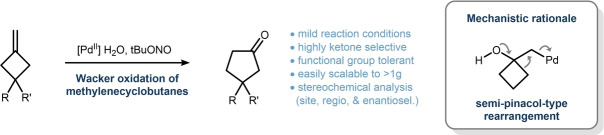

## Introduction

**Scheme 1:**
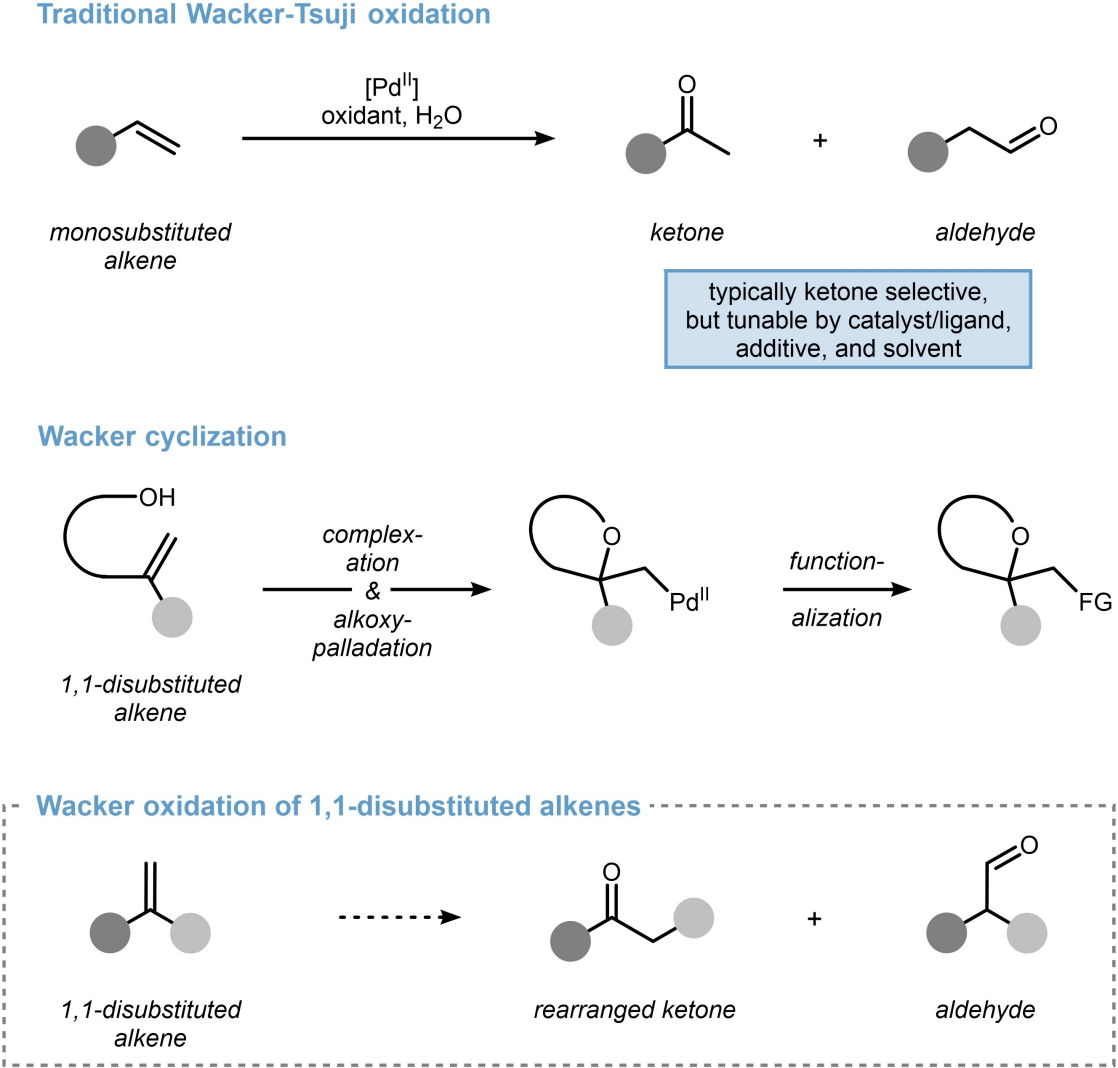
Current scope and limitations of the Wacker oxidation. FG=functional group.

Since its discovery in 1956,^[1]^ the Wacker oxidation continues to present an indispensable method to convert olefins into carbonyls.[^2^, ^3^] Traditionally, palladium chloride is used as the catalyst and copper chloride mediates an aerobic re‐oxidation in an aqueous reaction medium. The sequence works particularly well for monosubstituted alkenes, which can either undergo ketone‐selective^[4]^ or aldehyde‐selective[^5^, ^6^] oxidation based on the respective substrate bias and applied reaction conditions (Scheme 1, top).^[7]^ Internal alkenes can also be addressed, even though highly regioselective protocols are less frequent.^[8]^ In contrast, Wacker oxidation of 1,1‐disubstiuted alkenes remains mostly unexplored with the exception of palladium‐catalyzed intramolecular ring‐closure from pendent hydroxy‐groups, a process that is generally referred to as Wacker‐cyclization (Scheme 1, middle).^[9]^ While some rare reports describe classical Wacker oxidation of 1,1‐disubstituted alkenes towards aldehydes,^[10]^ ketones are generally not accessible via this sequence due to a lacking β‐hydrogen atom. However, we speculated that when incorporating a formal 1,2‐carbon shift within the Wacker oxidation, a rearranged ketone would become accessible from a 1,1‐disubstituted alkene precursor (Scheme 1, bottom). Within this research article, we summarize our results spanning from the aforementioned hypothesis towards developing the first highly efficient process.

**Scheme 2:**
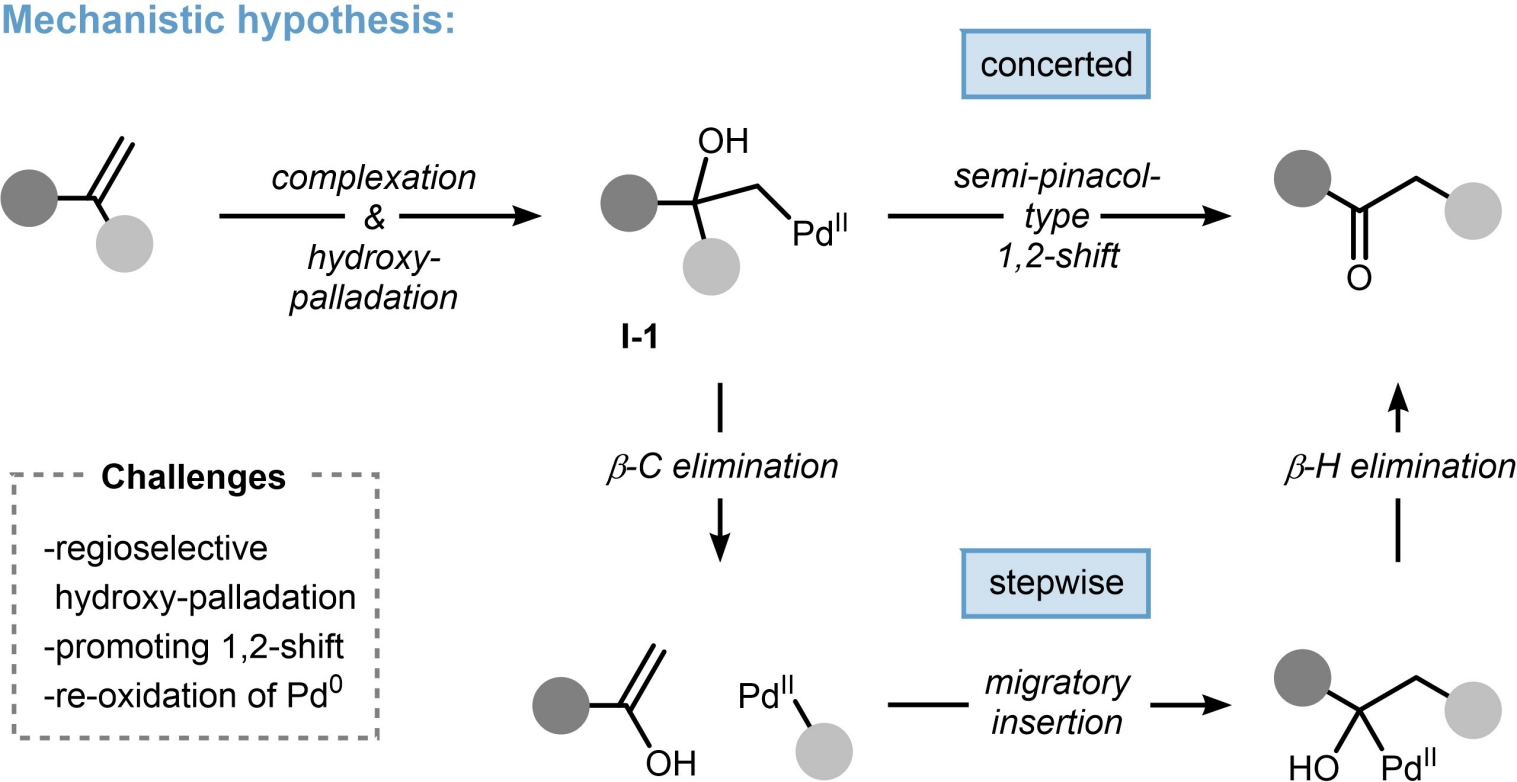
Mechanistic hypothesis for the key rearrangement of the Wacker oxidation of 1,1‐disubstituted alkenes.

Our detailed hypothesis on how to access the rearranged ketones is outlined in Scheme 2. Based on the plethora of mechanistic studies on hydroxypalladation of monosubstituted alkenes,^[11]^ we assumed that a related process should also be amenable to 1,1‐disubstituted alkenes leading to a respective intermediate of the general type **I‐1**. While this process might be reversible and generally not productive, **I‐1** shares some interesting similarities with the starting materials of semipinacol rearrangements, namely a tertiary alcohol with Pd^II^ as a latent leaving group in a 1,2‐relationship. Thus, a concerted 1,2‐shift towards the rearranged Wacker‐type ketone can be anticipated (Scheme 2, top). Alternatively, a stepwise 1,2‐shift via β‐carbon elimination and re‐insertion across the alkene may also be plausible (Scheme 2, bottom).

To achieve such a sequence, methylenecyclobutanes (MCBs) were envisioned as ideal substrates due to the wealth of palladium‐catalyzed 1,2‐shifts of alkenyl‐ and alkynylcyclobutanols to the corresponding cyclopentanones.[^12^, ^13^, ^14^] Furthermore, stoichiometric Pd^II^ nitrite^[15]^ was reported to promote oxidative ring expansion of MCB to cyclopentanone providing further evidence that — given a suitable oxidant can be identified — a Wacker reaction of this type of 1,1‐disubstituted alkene may be realized. First hints about such a catalytic process were disclosed by Boontanonda and Grigg in 1977, who described an oxidative ring‐expansion for three substrates.^[16]^

## Results and Discussion

We commenced our survey on identifying suitable Wacker conditions for MCB oxidation by using 3,3‐diphenylmethylenecyclobutane (**1 a**) as a model substrate (Table 1 highlights our major findings, for full optimization see Supporting Information). Under classical Wacker‐Tsuji conditions (entry 1), no conversion to the expected Wacker products **2 a** and **3 a** was observed. Switching the solvent to tBuOH indicated a small but detectible amount of carbonylic products (entry 2). While this result reveals the principial feasibility of alkene oxidation, the required re‐oxidation of the accumulating Pd^0^ was unsuccessful in this case. To address this limitation, a number of oxidants such as benzoquinone (BQ) or tert‐butyl hydroperoxide were tested, albeit without improvement (entries 3 & 4). By contrast, when using a palladium nitrite complex instead of palladium chloride,^[17]^ re‐oxidation under aerobic conditions using CuCl_2_ as mediator turned out to be successful. Thus, the rearranged ketone **2 a** was obtained in 77 % yield along with 12 % of the respective aldehyde **3 a** underpinning the importance of nitrite for this oxidation (compare entry 5 to entry 2). The solvent was found to be crucial for ketone‐to‐aldehyde selectivity with improved results for alcohols such as iPrOH or EtOH (entries 6 & 7). Similar observations were recently made by Kang and co‐workers.^[7c]^ Unfortunately, the improved ketone‐selectivity came at the expense of an overall drop in yield. However, it was found that the activity can be restored by switching the oxidant to tBuONO and the catalyst back to the commercially available palladium dichloride acetonitrile complex providing 95 % yield of cyclopentanone **2 a** while completely suppressing the formation of aldehyde side‐product (entry 8). Successful re‐oxidation of Pd^0^ by alkyl nitrite has been previously described.^[18]^ Further evaluation of the reagents indicated that tBuONO acts as the terminal oxidant in this process (entry 9), water is required for efficient turn‐over (entry 10), and chloride is essential as counter‐ion to the palladium catalyst (entry 11). Finally, the reaction time could be reduced to 3 h and the catalyst loading dropped to 5 mol % without any loss in performance (entry 12).

**Table 1:** Reaction optimization.

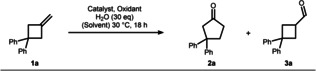					
#	Catalyst	Oxidant (equiv)	Solvent	Yield [%]	**2 a** : **3 a** ^[a]^
1	PdCl_2_	CuCl_2_ (0.4) O_2_	DMF/H_2_O	–	–
2	PdCl_2_(MeCN)_2_	CuCl_2_ (0.4)^[b]^	tBuOH	13	81 : 19
3	PdCl_2_(MeCN)_2_	BQ (1.0)	tBuOH	<1	–
4	PdCl_2_(MeCN)_2_	tBuOOH (1.0)	tBuOH	11	
5	Pd(NO_2_)Cl(MeCN)_2_	CuCl_2_ (0.4)^[b]^	tBuOH	88	88 : 12
6	Pd(NO_2_)Cl(MeCN)_2_	CuCl_2_ (0.4)^[b]^	iPrOH	44	91 : 9
7	Pd(NO_2_)Cl(MeCN)_2_	CuCl_2_ (0.4)^[b]^	EtOH	18	>99 : 1
8	PdCl_2_(MeCN)_2_	tBuONO (1.0)	EtOH	95	>99 : 1
9	PdCl_2_(MeCN)_2_	tBuONO (0.2) O_2_	EtOH	19	>99 : 1
10^[c]^	PdCl_2_(MeCN)_2_	tBuONO (1.0)	EtOH	36	>99 : 1
11	Pd(OAc)_2_	tBuONO (1.0)	EtOH	<1	–
12^[d]^	PdCl_2_(MeCN)_2_	tBuONO (1.0)	EtOH	95	>99 : 1

Reactions were run on a 0.1 mmol scale in 1 mL of solvent [0.1 M] using 10 mol % of catalst. [a] Yield and selectivity of **2 a** and **3 a** was determined by ^1^H NMR from the crude reaction mixture using mesitylene as an internal standard. [b] Atmospheric oxygen was used as the terminal oxidant. [c] The reaction was run without the addition of water and under an Ar atmosphere. [d] Reaction was run with 5 mol % catalyst and stopped after 3 h.

**Scheme 3:**
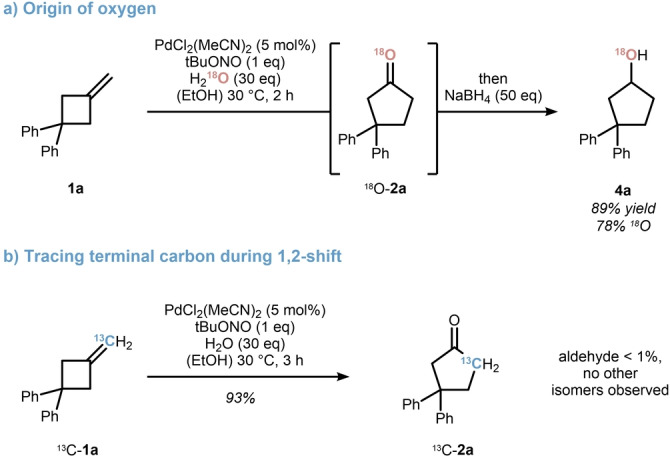
Labelling experiments to probe the mechanistic hypothesis.

To gain some insight into the origin of the oxygen atom of **1 a**, ^18^O‐labelled water was used as a mass‐sensitive tracer (Scheme 3a). To prevent any oxygen scrambling during work‐up and purification, the reaction was stopped by the addition of excess sodium borohydride after 2 h. Thus, cyclobutanol **4 a** was isolated as the sole product in 89 % yield. A 78 % ^18^O‐content was detected by mass spectrometry supporting an initial hydroxypalladation step. Next, terminally ^13^C‐labelled methylenecyclobutane ^13^C‐**1 a** was subjected to the reaction sequence providing cyclopentanone ^13^C‐**2 a** in agreement with a 1,2‐carbon shift. Any attempts to trap an intermediate arising from a β‐carbon elimination, such as running the reaction under a CO atmosphere, were unsuccessful. While this alludes to a concerted 1,2‐shift, it does not rule out a stepwise mechanism as suggested in Scheme 2.^[19]^

**Scheme 4:**
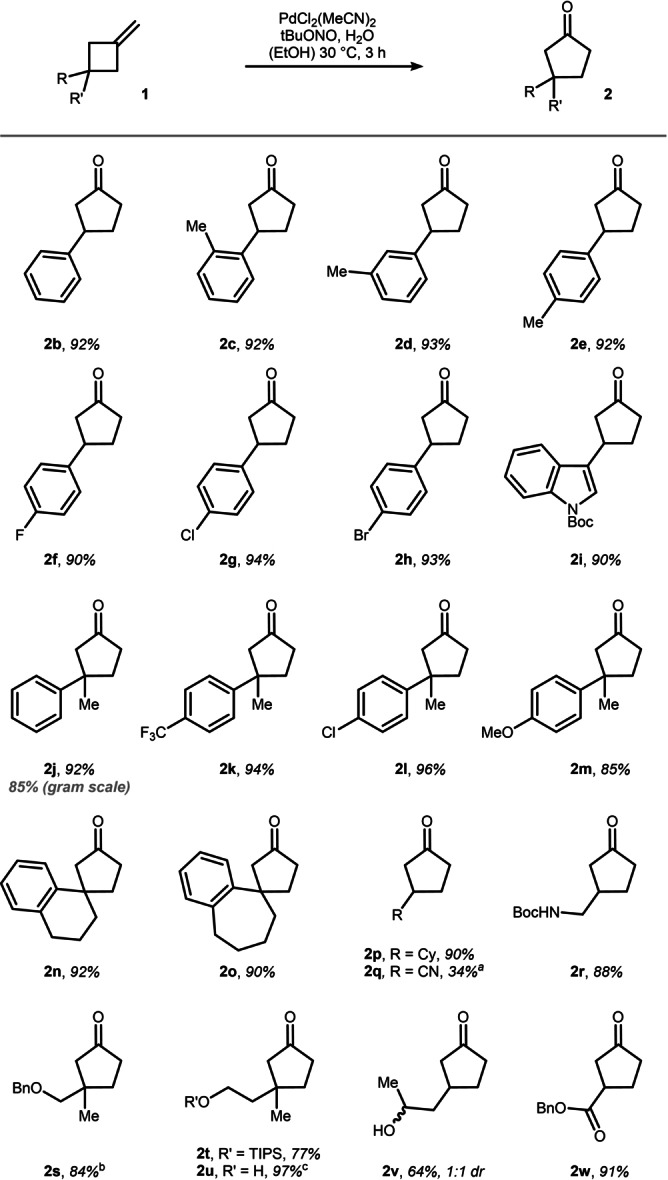
Reactions were run in EtOH [0.1 M] on a 0.3 mmol scale using 5 mol % of catalyst, 1 equiv of tBuONO, and 30 equiv of H_2_O. [a] Minor amounts of the respective aldehyde were formed for this substrate. [b] Reaction run at a 0.18 mmol scale. [c] Isolated as ketone:hemiketal mixture (66 : 34 ratio).

After having studied the mechanism and critical reaction parameters, we set out to explore the scope of the Wacker oxidation (Scheme 4). 3‐phenyl‐MCB (**1 b**) was a viable substrate as indicated by the formation of 3‐phenylcyclopentanone **2 b** in 92 % yield. Steric and electronic perturbation at the phenyl ring was well tolerated under the optimized conditions (**2 c**–**2 h**). Worth mentioning is bromo‐substitution (**2 h**), which did not interfere with the transiently formed Pd^0^. Heterocycles such as indole (**2 i**) were also compatible with the oxidative reaction protocol. The reaction works equally efficiently on a range of 3,3‐disubstituted MCBs (**2 j**–**2 m**) and can be easily run on gram‐scale with only minor deviations (**2 j**). Spirocyclic (**2 n**–**2 o**) and fully saturated cyclopentanones (**2 p**) were furnished by the Wacker oxidation in persistently ≥90 % yield. Interestingly, cyanocyclopentanone **2 q** was isolated in a moderate 34 % yield. While the low yield can be explained by potential HCN elimination and the volatility of the products, it is important to note that in this single case small amounts (4 %) of the corresponding aldehyde were detected by NMR analysis of the crude reaction mixture. In contrast, functional groups such as protected amines, ethers, free alcohols, and esters were also evaluated providing the respective cyclopentanones **2 r**–**2 w** in good to excellent yield without detectable aldehyde formation. The reaction was also found to be limited to methylenecyclobutanes as substrates. Smaller rings such as methylenecyclopropanes, as well as larger rings such as methylenecyclohexanes did not undergo ring expansion under the optimized conditions and the unreacted alkenes were detected in both cases (see Supporting Information for further details).

**Scheme 5:**
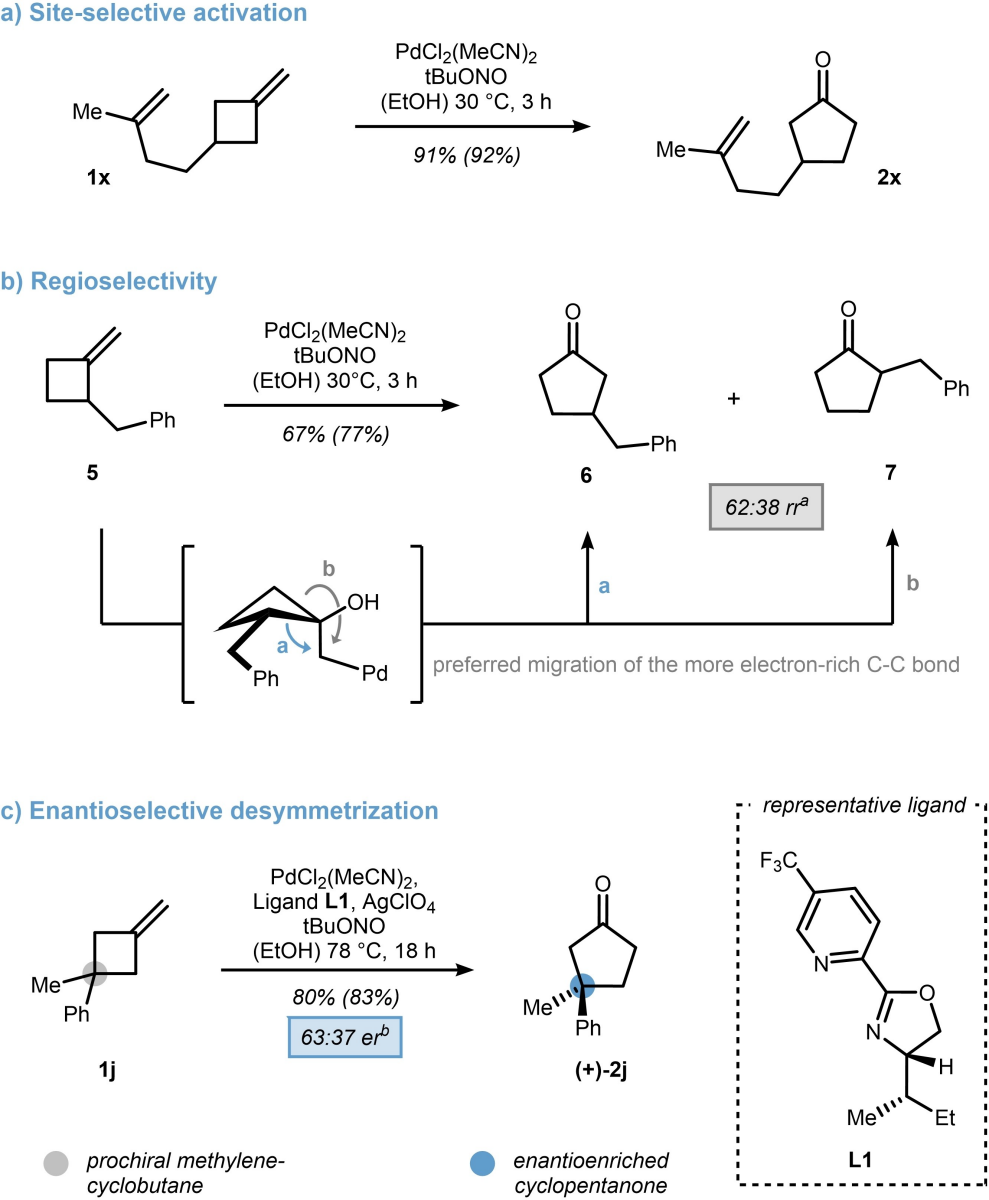
Selectivity analysis of the Wacker oxidation including a) alkene‐tethered, b) α‐substituted, and c) prochiral methylenecyclobutanes. Yield of the crude reaction mixture is given in paranthesis and was determined by ^1^H NMR using mesitylene as an internal standard. [a] regioisomeric ratio was determined from the crude reaction mixture by ^1^H NMR spectroscopy. [b] Enantiomeric ratio was determined by HPLC using a chiral column.

After exploring the substrate scope, we became interested in the selectivity of the oxidation process. Not surprisingly, the reaction was highly site‐selective as indicated by the oxidation of diene **1 x** to cyclopentanone **2 x** in 91 % isolated yield (Scheme 5, top). Furthermore, 2‐benzyl‐MCB **5** was used as a mechanistic probe to test the migration aptitudes of secondary vs. primary C−C bonds. A minor preference for the C‐3 functionalized cyclopentanone **6** over its C‐2 counterpart **7** was witnessed indicating a faster 1,2‐shift from the higher substituted bond (Scheme 4b, path a). Prochiral MCBs such as **1 j** bear the potential for enantioselective desymmetrization and prompted us to evaluate a small ligand set (14 ligands were tested, see Supporting Information for full details). As proof of concept, pyridine‐oxazoline (pyox) ligand **L1** is highlighted, which provides the cyclopentanone (+)‐**2 j** in a 63 : 37 enantiomeric ratio (er).^[20]^ To push the reaction to a reasonable conversion, raising the temperature to 78 °C and replacing the chloride counterion with a perchlorate was essential. This result not only resembles the first enantioselective desymmetrization of a MCB, but also provides further credibility to the key rearrangement occurring from a palladium‐mediated semipinacol shift.

## Conclusion

In summary, ketone‐selective Wacker oxidation of 1,1‐disubstituted alkenes was achieved via a semipinacol‐type rearrangement using strained MCBs. During the optimization, tert‐butylnitrite was found to play a crucial role in activating the palladium catalyst through nitrite supply and in mediating efficient re‐oxidation of Pd^0^. Thus, a number of cyclopentanones can be accessed with good functional group tolerance and in only 3 h at 30 °C. The stereochemical outcome of the reaction was studied and a suitable pyox‐ligand that allows desymmetrization of prochiral MCBs identified. Thus, enantioselective Wacker oxidation, which has been elusive until now, becomes possible providing a new entry to cyclopentanones as widely useful chiral building block.

## Conflict of interest

The authors declare no conflict of interest.

1

## Supporting information

As a service to our authors and readers, this journal provides supporting information supplied by the authors. Such materials are peer reviewed and may be re‐organized for online delivery, but are not copy‐edited or typeset. Technical support issues arising from supporting information (other than missing files) should be addressed to the authors.

Supporting Information

## Data Availability

The data that support the findings of this study are available in the Supporting Information of this article.
