# Supplementary material for: Wacker Oxidation of Methylenecyclobutanes: Scope and Selectivity in an Unusual Setting
Source: Angew Chem Int Ed Engl. 2023 Jan 12;62(7):e202215381. doi: 10.1002/anie.202215381 (PMC10108300; doi:10.1002/anie.202215381)

Angewandte Chemie
Eine Zeitschrift der Gesellschaft Deutscher Chemiker

Supporting Information

Wacker Oxidation of Methylenecyclobutanes: Scope and Selectivity in an Unusual Setting

*J. Sietmann, M. Tenberge, J. M. Wahl**

Contents

1 General ... 1

2 Synthesis of starting materials ... 3

2.1 General procedures ... 3

2.2 Syntheses of alkenes for [2+2]-cycloaddition ... 5

2.3 Syntheses of cyclobutanones ... 7

2.4 Syntheses of methylenecyclobutanes ... 12

2.5 Syntheses of other methylenecycloalkenes ... 26

2.6 Synthesis of ligand L1 ... 26

3 Optimization of reaction protocols ... 29

3.1 Optimization of the racemic reaction conditions ... 29

3.2 Reoptimization of standard reaction conditions for the asymmetric ring expansion ... 35

3.3 Ligand optimization ... 35

4 Isotope labeling experiments ... 38

4.1 ^{18}O-Labeling experiments ... 38

4.2 ^{13}C-Labeling experiments ... 40

5 Synthesis of cyclopentanones ... 43

5.1 General procedure E: Oxidative ring expansion ... 43

5.2 Syntheses ... 43

5.3 Unsuccessful substrates ... 54

5.4 Stereochemical analysis ... 54

6 References ... 57

7 ^{1}H, ^{13}C and ^{19}F NMR spectra ... 60

1 General

Nuclear magnetic resonance (NMR) spectra were recorded by the analytical departments of the Organisch-Chemisches Institut at the Westfälische Wilhelms-Universität and of the Department Chemie at Johannes Gutenberg-Universität Mainz. Following spectrometers were used: An *Avance II 400* (*Bruker*), a *DD2 500* (*Agilent*), a *DD2 600* (*Agilent*), an *Avance III HD 300* (*Bruker*), an *Avance III HD 400* (*Bruker*). Spectra were recorded at 26 °C (unless otherwise noted). Chemical shifts are reported in ppm with the solvent resonance as the internal standard (^{1}H NMR $CHCl_3$: δ = 7.26 ppm; ^{13}C NMR $CDCl_3$: δ = 77.16 ppm). The data is reported as follows: chemical shift, multiplicity (s = singlet, d = doublet, t = triplet, q = quartet, p = pentet, br = broad, m = multiplet or combinations of these), coupling constants (Hz) and integration. Apparent multiplicity, which occurs as a result of accidental equality of coupling constants to magnetically non-equivalent protons, is marked as app.

Infrared (IR) spectra were obtained on a *Perkin-Elmer 100 FT-IR* spectrometer or on a *Jasco FT/IR-4100* and are reported in wavenumbers (cm^{-1}). Bands are characterized as broad (br), strong (s), medium (m), and weak (w).

Melting points (M.P.) were measured on a *Büchi B-545* and are reported uncorrected.

High Resolution Mass Spectrometry (HRMS) was performed by the analytical department of the Organisch-Chemisches Institut at the Westfälische Wilhelms-Universität and of the Department Chemie at Johannes Gutenberg-Universität Mainz. Spectra were recorded on a *Bruker Daltonics MicroTof*, on a *Thermo-Fisher Scientific Orbitrap LTQ XL* or an *Agilent G6545AQ-ToF*. Signals are reported as mass to charge ratio *m*/*z*. GC-MS data was acquired using an *Agilent 7890A* Gas Chromatograph and an *Agilent 5975* or *5975 VL MSD* Inert Mass Selective Detector (EI) and is reported as *m*/*z* (relative intensity).

Optical rotations were measured on a *Perkin-Elmer 241 polarimeter* at 589 nm wavelength (Na D-line) using a standard 10 cm cell (1 mL). Specific rotations, $[\alpha]_{D}^{T}$, are reported in °·mL/(g·dm) at the specific temperature. Concentrations (c) are given in grams per 100 mL of the specific solvent.

Analytical high-performance liquid chromatography (HPLC) measurements were performed on the following system: *Knauer* HPLC Pump Smartline 1000 with degassing unit, *Knauer* Autosampler Smartline 3950, *Knauer* UV-detector Smartline

2550, *Knauer* RI-detector Smartline 2300. Separation was performed using Lux® i-Cellulose-5 (4.6 × 250 nm × 5 µm, *Phenomenex Ltd.*).

Purification was performed either with standard column chromatography (FC) techniques using 60 M silica gel (0.04–0.063 mm, *MACHEREY-NAGEL*), 40–63 µm silica gel (*VWR chemicals*) or Geduran® Si 60 (0.04–0.063 mm, *Millipore*) or on an automated flash chromatography system *Biotage Isolera One* utilizing *Biotage Sfär Silica D-Duo 60 µm* columns (5 g, 25 g, 100 g). Glass silica gel plates 60 F254 (*Merck*) were used for thin layer chromatography (TLC) using UV light (254/366 nm), $KMnO_4$ (1.5 g $KMnO_4$, 5 g $NaHCO_3$ and 5 mL NaOH 10% in 200 mL H_2O) and CAM (0.5 g $Ce(NH_4)_2(NO_3)_6$ and 24.0 g of $(NH_4)_6Mo_7O_{24}\cdot 4H_2O$, 28 mL H_2SO_4 in 200 mL H_2O) for detection.

Chemicals were purchased from *Alfa Aesar*, *Acros Organics*, *Sigma Aldrich*, *BLDpharm*, *FluoroChem*, *Carbolution* or *ABCR* and used as received. All work-up and purification procedures were carried out with pre-distilled technical grade solvents. Dry solvents were either dried with standard techniques (EtOH dried over activated 3 Å molecular sieves), or collected from a *MBraun MB SPS-800* (CH_2Cl_2, Et_2O, THF). A positive argon pressure was used to pass the solvents through the following columns:

CH_2Cl_2: 2 × MB-KOL-A
Et_2O: 1 × MB-KOL-A and 1 × MB-KOL MT2-250
THF: 2 × MB-KOL MT2-150°C

All reactions involving air or moisture sensitive reagents were carried out in oven-(125 °C) and flame-dried glassware under argon or nitrogen atmosphere using standard Schlenk techniques. Reactions requiring heating were conducted using aluminium blocks as heating source.

2 Synthesis of starting materials

2.1 General procedures

General procedure A: Wittig olefination

In an oven dried Schlenk tube methyltriphenylphosphomium bromide (1.35 eq.) was suspended in dry THF (0.1 M) at 0 °C. A solution of *n*-butyl lithium (1.30 eq., in *n*-hexane, 1.6 M) was added dropwise and the mixture was stirred for 30 min at 0 °C. The substrate was added to the yellow suspension and the mixture was stirred at room temperature (rt) until full conversion was monitored *via* TLC. Water (half amount of THF) was added, the layers were separated and the aq. layer was extracted with CH_2Cl_2 (3 × 50 mL). The combined org. layers were dried over $MgSO_4$, filtered and concentrated under reduced pressure. The product was isolated by flash column chromatography with the conditions given in the corresponding entry.

General procedures for the synthesis of cyclobutanones

General procedure B: [2+2] cycloaddition of keteneiminium salt

Following a modified procedure by *Chernykh et al.*,[1] a Schlenk tube was charged with dimethylacetamide (1.20 eq.) in 1,2-dichloroethane (0.5 M). The reaction solution was kept at rt with a water bath. Tf_2O (2.00 eq.) was added dropwise and the reaction mixture was stirred at rt for 10 min. A solution of the corresponding alkene (1.00 eq.) and 2,6-lutidine (2.00 eq.) in 1,2-dichloroethane (2.00 M) was added dropwise to the reaction mixture, which was stirred at 90 °C for 8 h. The reaction was allowed to cool to rt and water (20 mL) was added. The reaction mixture was stirred at 90 °C for 16 h. The mixture was allowed to cool to rt and the layers were separated. The aq. layer was extracted with CH_2Cl_2 (3 × 50 mL). The combined org. layers were dried over $MgSO_4$, filtered and the solvent was removed under reduced pressure. The product was isolated by flash column chromatography with the conditions given in the corresponding entry.

General procedure C: [2+2] cycloaddition of dichloroketene

Following a modified procedure by *Malkov et al.*,[2] a Schlenk flask was charged with Zn dust (6.00 eq.) and freshly distilled Et_2O (0.14 M). The corresponding alkene (1.00 eq.) was added to the suspension. The suspension was kept at rt with a water bath while a solution of trichloroacetyl chloride (2.50 eq.) and $POCl_3$ (1.10 eq.)

in freshly distilled Et_2O (0.5 M) was added. The water bath was removed and the suspension was stirred at 40 °C for 8 h. After complete conversion, the suspension was allowed to cool down to rt and filtered through a pad of Celite® and washed with CH_2Cl_2. The solvents were removed under reduced pressure and CH_2Cl_2 (100 mL) was added. The org. layer was washed with water (3 × 100 mL) and with sat. aq. $NaHCO_3$ sol. (3 × 100 mL). The org. layer was dried over $MgSO_4$, filtered and the solvent was removed under reduced pressure. The crude reaction mixture was used without further purification.

To a round bottom flask the crude reaction mixture of the first reaction step and glacial acetic acid (0.1 M) were added. The solution was kept at 20 °C with a water bath and Zn dust (4.00 eq.) was slowly added. The suspension was heated to 80 °C and stirred for 16 h. The mixture was allowed to cool down to rt, filtered through a pad of Celite® and washed with CH_2Cl_2. The solvent was removed under reduced pressure. The residue was redissolved in CH_2Cl_2 (50 mL) and the org. layer was washed with an sat. aq. $NaHCO_3$ sol. (3 × 50 mL) and water (3 × 50 mL). The org. layer was dried over $MgSO_4$, filtered and the solvent was removed under reduced pressure. The product was obtained by flash column chromatography with the conditions given in the corresponding entry.

General procedure D: [2+2] cycloaddition of dichloroketene under ultrasonication

Following a modified procedure by *Du et al.*,[3] a flame dried three neck flask equipped with condenser and dripping funnel und N_2-atmosphere was charged with Zn/Cu-Couple (2.00 eq.), the corresponding alkene (1.00 eq.) and dry Et_2O (0.2 M with respect to the alkene). The mixture was placed in an ultrasonic bath and a solution of trichloroacetyl chloride (1.50 eq.) in dry Et_2O (0.6 M) was added dropwise over 1 h under sonication. The temperature of the ultrasonic bath was maintained between 15–20 °C by addition of ice. After complete addition, the mixture was sonicated under these conditions for at least two additional hours or until TLC showed no further conversion. Then, water is added and the reaction mixture was filtered through a plug of Celite® which was washed with Et_2O. The filtrate was washed with water, sat. aq. $NaHCO_3$ sol. and brine. The combined org. layers were dried over $MgSO_4$, filtered and concentrated in vacuo. The crude product was used without further purification.

The crude product of the first step was dissolved in glacial acetic acid (1.2 M) and added to a stirred suspension of zinc dust (6.40 eq. with respect to the alkene from the first step, –100 mesh) in glacial acetic acid (0.4 mL per gram Zn). The mixture was heated to reflux for 4 h before it was allowed to cool to rt. Then, water is added and the reaction mixture was filtered through a plug of Celite® which was washed with Et_2O. The phases of the filtrate were separated and the aq. layer was extracted with Et_2O (2x). The combined org. layers were washed with water, sat. aq. $NaHCO_3$ sol. and brine. The org. phase was dried over $MgSO_4$, filtered and concentrated in vacuo. The product was obtained by flash column chromatography with the conditions given in the corresponding entry.

2.2 Syntheses of alkenes for [2+2]-cycloaddition

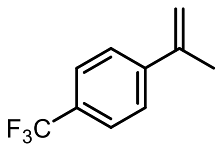

1-(Prop-1-en-2-yl)-4-(trifluoromethyl)benzene [S1]

Following general procedure **A** using 4-(trifluoromethyl)acetophenone (2.822 g, 15.00 mmol, 1.00 eq.) the desired product was obtained *via* FC (pentane) as colorless oil (2.353 g, 12.63 mmol, 84%).

^{1}H NMR (400 MHz, $CDCl_3$): δ = 7.62 – 7.52 (m, 4H), 5.45 (s, 1H), 5.20 (*app* p, $J \approx 1.4$ Hz, 1H), 2.20 – 2.15 (m, 3H). **^{13}C NMR (101 MHz, $CDCl_3$)**: δ = 144.9, 142.4, 129.6 (q, J = 32.4 Hz), 125.9, 125.3 (q, J = 3.8 Hz), 124.4 (q, J = 271.8 Hz), 114.9 – 114.4 (m), 21.8 (q, J = 5.1 Hz). **^{19}F{^{1}H} NMR (377 MHz, $CDCl_3$)**: δ = −62.49. Spectroscopic data was in agreement with that previously reported.[4]

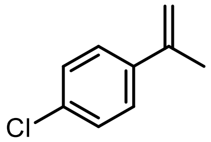

1-Chloro-4-(prop-1-en-2-yl)benzene [S2]

Following general procedure **A** using 4-chloroacetophenone (1.945 mL, 2.318 g, 15.00 mmol, 1.00 eq.) the desired product was obtained *via* FC (pentane) as colorless oil (1.962 g, 12.86 mmol, 86%).

^{1}H NMR (400 MHz, CDCl$_3$): δ = 7.42 – 7.36 (m, 2H), 7.33 – 7.27 (m, 2H), 5.36 (dq, J = 1.6, 0.8 Hz, 1H), 5.10 (p, J = 1.5 Hz, 1H), 2.13 (dd, J = 1.5, 0.8 Hz, 3H). **^{13}C NMR (101 MHz, CDCl$_3$)**: δ = 142.3, 139.8, 133.3, 128.5, 126.9, 113.1, 21.9. Spectroscopic data was in agreement with that previously reported.[5]

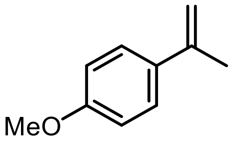

1-Methoxy-4-(prop-1-en-2-yl)benzene [S3]

Following general procedure **A** using 1-(4-methoxyphenyl)ethan-1-one (2.253 g, 15.00 mmol, 1.00 eq.) the desired product was obtained *via* FC (pentane) as colorless oil (1.067 g, 7.199 mmol, 48%).

^{1}H NMR (400 MHz, CDCl$_3$): δ = 7.48 – 7.38 (m, 2H), 6.95 – 6.82 (m, 2H), 5.30 (m, 1H), 5.00 (m, 1H), 3.82 (s, 3H), 2.14 (s, 3H). **^{13}C NMR (101 MHz, CDCl$_3$)**: δ = 159.2, 142.7, 133.9, 126.7, 113.7, 110.8, 55.4, 22.1. Spectroscopic data was in agreement with that previously reported.[6]

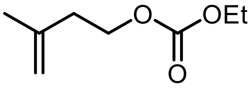

Ethyl (3-methylbut-3-en-1-yl) carbonate [S4]

Following a procedure by *Horn and Kazmaier*,[7] 3-methylbut-3-en-1-ol (1.51 mL, 15.0 mmol, 1.00 eq.) was dissolved in dry CH_2Cl_2 (20 mL) and cooled to 0° C. Pyridine (1.82 mL, 22.5 mmol, 1.50 eq.) was added followed by dropwise addition of ethyl chloroformate (1.71 mL, 18.0 mmol, 1.20 eq.). The mixture was gradually warmed to rt and stirred for 3 d. Then, Et_2O (10 mL) was added and it was washed with 1 M aq. HCl (2 × 50 mL). The aq. layer was extracted with Et_2O (20 mL). The combined org. layers were washed with brine (50 mL), dried over Na_2SO_4, filtered and concentrated *in vacuo* affording the desired product as colorless oil (2.05 g, 12.9 mmol, 86%).

IR (neat): $\tilde{\nu}$ = 2988 (w), 2938 (w), 2917 (w), 2353 (w), 2341 (w), 1746 (s), 1651 (w), 1457 (w), 1382 (w), 1256 (s), 1090 (w), 1025 (w), 952 (w), 894 (w), 849 (w), 793 (w), 733 (w). **^{1}H NMR (400 MHz, CDCl$_3$)**: δ = 4.90 – 4.70 (m, 2H), 4.23 (t, J = 6.9 Hz, 2H), 4.18 (q, J = 7.1 Hz, 2H), 2.42 – 2.34 (m, 2H), 1.76 (t, J = 1.2 Hz, 3H), 1.30 (t, J = 7.1 Hz, 3H). **^{13}C NMR (101 MHz, CDCl$_3$)**: δ = 155.3, 141.3, 112.7, 66.0, 64.0, 36.8,

22.6, 14.4. **HRMS (APCI, CH_3COONH_4)**: Calculated for $C_8H_{15}O_3^+$ $[M+H]^+$: 159.1012, found: 159.1016.

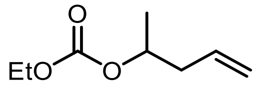

Ethyl pent-4-en-2-yl carbonate [S5]

Following a procedure by *Horn and Kazmaier*,[7] 4-penten-2-ol (2.06 mL, 20.0 mmol, 1.00 eq.) was dissolved in dry CH_2Cl_2 (20 mL) and cooled to 0 °C. Pyridine (2.42 mL, 30.0 mmol, 1.50 eq.) was added followed by dropwise addition of ethyl chloroformate (2.29 mL, 24.0 mmol, 1.20 eq.). The mixture was gradually warmed to rt and stirred for 18 h. Then, Et_2O (10 mL) was added and it was washed with 1 M aq. HCl (2 × 25 mL). The aq. layer was extracted with Et_2O (20 mL). The combined org. layers were washed with brine (25 mL), dried over $MgSO_4$, filtered and concentrated *in vacuo* affording the desired product as colorless oil (3.03 g, 19.1 mmol, 96%).

IR (neat): $\tilde{\nu}$ = 2982 (w), 2931 (w), 1743 (s), 1642 (w), 1461 (w), 1373 (m), 1261 (s), 1133 (w), 1055 (w), 1011 (m), 919 (w), 878 (w), 772 (m), 759 (w), 738 (m). **^{1}H NMR (400 MHz, $CDCl_3$)**: δ = 5.86 – 5.70 (m, 1H), 5.16 – 5.05 (m, 2H), 4.87 – 4.72 (m, 1H), 4.22 – 4.11 (m, 2H), 2.47 – 2.36 (m, 1H), 2.36 – 2.25 (m, 1H), 1.33 – 1.24 (m, 6H). **^{13}C NMR (101 MHz, $CDCl_3$)**: δ = 154.8, 133.4, 118.2, 74.2, 63.8, 40.4, 19.6, 14.4. **HRMS (APCI, CH_3COONH_4)**: Calculated for $C_8H_{14}O_3^+$ $[M+H]^+$: 159.1016, found: 159.1013.

2.3 Syntheses of cyclobutanones

Cyclobutanones **S6**–**S15** were synthesized according to literature procedures.[8] Cyclobutanone **S16** (CAS [198995-91-4]) was commercially available.

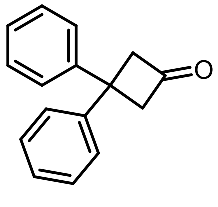

3,3-Diphenylcyclobutan-1-one [S17]

Following general procedure **B** using ethene-1,1-diyldibenzene (2.163 g, 12.00 mmol, 1.00 eq.) the desired product was obtained *via* FC (pentane:Et_2O, 20:1) as an off-white solid (1.478 g, 6.649 mmol, 55%).

^{1}H NMR (400 MHz, CDCl$_3$): δ = 7.33 – 7.23 (m, 8H), 7.22 – 7.14 (m, 2H), 3.77 (s, 4H). **^{13}C NMR (101 MHz, CDCl$_3$)**: δ = 205.7, 147.3, 128.8, 126.8, 126.6, 60.6, 42.1. Spectroscopic data was in agreement with that previously reported.[9]

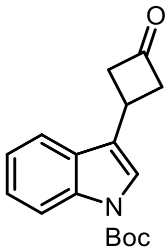

tert-Butyl 3-(3-oxocyclobutyl)-1*H*-indole-1-carboxylate [S18]

Following general procedure **C** using *tert*-butyl 3-vinyl-1*H*-indole-1-carboxylate (1.300 g, 5.00 mmol, 1.00 eq.) the desired product was obtained *via* FC (pentane:EtOAc; 20:1→10:1) as colorless oil (544 mg, 1.907 mmol, 36%).

IR (neat): $\tilde{\nu}$ = 3126 (w), 2980 (w), 2930 (w), 1780 (s), 1711 (s), 1628 (w), 1608 (w), 1475 (m), 1457 (s), 1433 (w), 1376 (s), 1301 (m), 1277 (s), 1259 (s), 1217 (m), 1202 (m), 1154 (s), 1105 (m), 1087 (s), 1044 (m), 1024 (m), 1016 (s), 934 (w), 859 (m), 848 (m), 828 (m), 816 (m), 765 (s), 747 (s), 724 (m), 673 (m). **^{1}H NMR (599 MHz, CDCl$_3$)**: δ = 8.16 (br s, 1H), 7.48 – 7.46 (m, 1H), 7.45 (br s, 1H), 7.40 – 7.31 (m, 1H), 7.28 – 7.24 (m, 1H), 3.83 – 3.75 (m, 1H), 3.63 – 3.54 (m, 2H), 3.34 – 3.26 (m, 2H), 1.68 (s, 9H). **^{13}C NMR (151 MHz, CDCl$_3$)**: δ = 206.8, 149.8, 136.1, 129.7, 124.9, 123.2, 122.8, 121.8, 119.2, 115.7, 84.0, 53.4, 28.4, 20.4. **HRMS (ESI)**: Calculated for $C_{17}H_{19}NO_3Na^+$ $[M+Na]^+$: 308.1254, found: 308.1263.

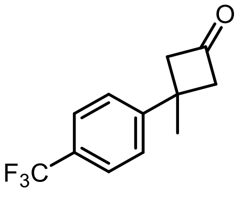

3-Methyl-3-(4-(trifluoromethyl)phenyl)cyclobutan-1-one [S19]

Following general procedure **B** using styrene **S1** (930.9 mg, 5.00 mmol, 1.00 eq.) the desired product was obtained *via* FC (pentane:Et_2O; 10:1) as colorless oil (621.2 mg, 2.722 mmol, 54%).

IR (neat): $\tilde{\nu}$ = 2962 (br), 2925 (br), 2871 (br), 1785 (s), 1619 (w), 1451 (w), 1411 (w), 1389 (w), 1325 (s), 1300 (m), 1163 (m), 1108 (s), 1086 (s), 1064 (s), 1015 (m), 955 (w), 875 (w), 840 (m), 718 (w), 672 (w). **^{1}H NMR (599 MHz, CDCl$_3$)**: δ = 7.63 (d,

J = 8.0 Hz, 2H), 7.43 (d, J = 8.0 Hz, 2H), 3.51 – 3.44 (m, 2H), 3.20 – 3.13 (m, 2H), 1.63 (s, 3H). **^{13}C NMR (151 MHz, CDCl$_3$)**: δ = 205.5, 152.3, 128.9 (q, J = 32.5 Hz), 126.3, 125.8 (q, J = 3.7 Hz), 124.3 (q, J = 271.9 Hz), 59.3, 34.3, 31.0. **^{19}F NMR (377 MHz, CDCl$_3$)**: δ = −62.46. **HRMS (EI)**: Calculated for $C_{12}H_{11}F_3O^+$ [M]$^+$: 228.0756, found: 228.0760.

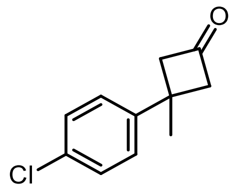

3-(4-Chlorophenyl)-3-methylcyclobutan-1-one [S20]

Following general procedure **B** using styrene **S2** (763.1 mg, 5.00 mmol, 1.00 eq.) the desired product was obtained *via* FC (pentane:Et_2O; 10:1) as colorless oil (787.3 mg, 4.044 mmol, 81%).

^{1}H NMR (400 MHz, CDCl$_3$): δ = 7.37 – 7.29 (m, 2H), 7.27 – 7.20 (m, 2H), 3.48 – 3.37 (m, 2H), 3.17 – 3.06 (m, 2H), 1.59 (s, 3H). **^{13}C NMR (101 MHz, CDCl$_3$)**: δ = 206.0, 146.9, 132.3, 128.8, 127.3, 59.4, 33.8, 31.1. Spectroscopic data was in agreement with that previously reported.[10]

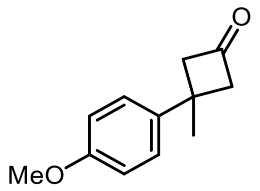

3-(4-Methoxyphenyl)-3-methylcyclobutan-1-one [S21]

Following general procedure **B** using styrene **S3** (1.066 g, 7.196 mmol, 1.00 eq.) the desired product was obtained *via* FC (pentane:Et_2O; 10:1) as colorless oil (827.4 mg, 4.349 mmol, 60%).

IR (neat): $\tilde{\nu}$ = 3035 (w), 2956 (br), 2920 (w), 2837 (w), 1779 (s), 1612 (m), 1581 (w), 1513 (s), 1465 (m), 1445 (m), 1413 (w), 1378 (m), 1302 (m), 1245 (s), 1179 (s), 1142 (m), 1110 (m), 1077 (m), 1032 (s), 912 (w), 870 (m), 829 (s), 787 (m), 731 (m), 672 (w). **^{1}H NMR (599 MHz, CDCl$_3$)**: δ = 7.27 – 7.20 (m, 2H), 6.93 – 6.87 (m, 2H), 3.81 (s, 3H), 3.48 – 3.39 (m, 2H), 3.14 – 3.05 (m, 2H), 1.59 (s, 3H). **^{13}C NMR (151 MHz, CDCl$_3$)**: δ = 207.0, 158.1, 140.5, 126.9, 114.1, 59.6, 55.5, 33.4, 31.2. **HRMS (EI)**: Calculated for $C_{12}H_{14}O_2^+$ [M]$^+$: 190.0987, found: 190.0988.

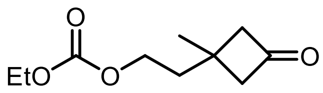

Ethyl (2-(1-methyl-3-oxocyclobutyl)ethyl) carbonate [S22]

Following general procedure **D** using alkene **S4** (2.00 g, 12.7 mmol, 1.00 eq.), Zn/Cu couple (1.65 g, 25.3 mmol, 2.00 eq.) and trichloroacetyl chloride (2.14 mL, 19.0 mmol, 1.50 eq.) in the first step and zinc dust (5.30 g, 81.1 mmol, 6.40 eq.) and acetic acid (10 mL) in the second step, the desired product was obtained *via* automated FC (CyH:EtOAc; 95:5→80:20) as colorless oil (745 mg, 3.72 mmol, 29%).

IR (neat): $\tilde{\nu}$ = 2970 (w), 2917 (w), 2877 (w), 2241 (w), 1784 (s), 1742 (s), 1459 (w), 1403 (w), 1386 (w), 1368 (m), 1252 (s), 1188 (w), 1144 (w), 1085 (w), 1005 (m), 879 (w), 792 (w), 733 (w), 644 (w). **^{1}H NMR (400 MHz, $CDCl_3$)**: δ = 4.24 (t, J = 6.9 Hz, 2H), 4.18 (q, J = 7.1 Hz, 2H), 2.99 – 2.87 (m, 2H), 2.83 – 2.71 (m, 2H), 2.02 (t, J = 6.9 Hz, 2H), 1.34 (s, 3H), 1.29 (t, J = 7.1 Hz, 3H). **^{13}C NMR (101 MHz, $CDCl_3$)**: δ = 207.4, 155.2, 65.3, 64.2, 58.8, 39.5, 27.7, 25.6, 14.4. **HRMS (ESI)**: Calculated for $C_{10}H_{16}O_4Na^+$ $[M+Na]^+$: 223.0941, found: 223.0943.

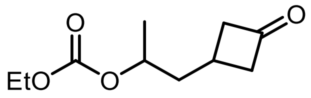

Ethyl (1-(3-oxocyclobutyl)propan-2-yl) carbonate [S23]

Following general procedure **D** using alkene **S5** (3.13 g, 15.8 mmol, 1.00 eq.), Zn/Cu couple (4.07 g, 31.5 mmol, 2.00 eq.) and trichloroacetyl chloride (4.30 g, 2.64 mL, 23.6 mmol, 1.50 eq.) in the first step and zinc dust (6.80 g, 104 mmol, 6.59 eq.) and acetic acid (15 mL) in the second step, the desired product was obtained *via* automated FC (CH_2Cl_2:MeOH; 100:0→99:1) as colorless oil (220 mg, 1.10 mmol, 7%).

IR (neat): $\tilde{\nu}$ = 2980 (w), 2942 (w), 2346 (w), 1785 (s), 1738 (s), 1461 (w), 1374 (m), 1346 (w), 1257 (s), 1173 (w), 1140 (w), 1103 (m), 1053 (w), 1009 (m), 924 (w), 862 (w), 819 (w), 792 (m), 672 (w), 640 (m), 618 (m), 607 (m). **^{1}H NMR (400 MHz, $CDCl_3$)**: δ = 4.79 (dqd, J = 8.0, 6.3, 4.7 Hz, 1H), 4.18 (q, J = 7.1 Hz, 2H), 3.26 – 3.08 (m, 2H), 2.84 – 2.69 (m, 2H), 2.49 (dtt, J = 15.3, 8.5, 6.7 Hz, 1H), 2.03 – 1.93 (m, 1H), 1.85 – 1.74 (m, 1H), 1.36 – 1.31 (d, J = 6.3, 3H), 1.30 (t, J = 7.1 Hz, 3H). **^{13}C NMR (101 MHz, $CDCl_3$)**: δ = 207.5, 154.9, 74.1, 64.0, 53.0, 52.8, 42.3, 20.8, 20.3, 14.4. **HRMS (ESI)**: Calculated for $C_{10}H_{17}O_4^+$ $[M+H]^+$: 201.1121, found 201.1119.

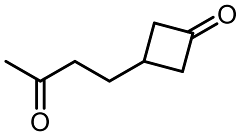

3-(3-Oxobutyl)cyclobutan-1-one [S24]

Following a procedure of *Wu et al.*,[11] to a solution of hex-5-en-2-one (1.002 g, 1.182 ml, 10.00 mmol, 1.00 eq.) in toluene (60.0 ml), ethane-1,2-diol (1.862 g, 1.678 ml, 30.00 mmol, 3.00 eq.) and PTSA (95.1 mg, 0.50 mmol, 0.05 eq.) were added. 2-(But-3-en-1-yl)-2-methyl-1,3-dioxolane was obtained as described and used in the next step without further purification.

Following general procedure **C** using 2-(but-3-en-1-yl)-2-methyl-1,3-dioxolane (1.784 g, 12.55 mmol, 1.00 eq.) the desired product was obtained *via* FC (pentane:Et_2O; 5:1→2:1) as colorless oil (392.2 mg, 2.798 mmol, 18% over two steps).

IR (neat): $\tilde{\nu}$ = 2926 (w), 1775 (s), 1711 (s), 1412 (m), 1358 (m), 1209 (m), 1167 (m), 1106 (m), 1067 (m), 996 (m), 889 (w), 735 (m), 703 (w). **^{1}H NMR (599 MHz, $CDCl_3$)**: δ = 3.19 – 3.09 (m, 2H), 2.74 – 2.59 (m, 2H), 2.48 (t, J = 7.4 Hz, 2H), 2.37 (ttt, J = 8.7, 7.7, 6.3 Hz, 1H), 2.16 (s, 3H), 1.87 (q, J = 7.5 Hz, 2H). **^{13}C NMR (151 MHz, $CDCl_3$)**: δ = 208.0, 207.5, 52.5, 42.2, 30.11, 30.09, 23.5. **HRMS (ESI)**: Calculated for $C_8H_{12}O_2Na^+$ $[M+Na]^+$: 163.0730, found: 163.0727.

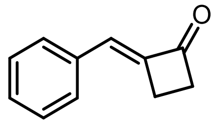

2-Benzylidenecyclobutan-1-one [S25]

An oven dried Schlenk tube was charged with $Ca(OH)_2$ (111 mg, 1.50 mmol, 0.10 eq.). Then, a solution of benzaldehyde (1.52 mL, 15.0 mmol, 1.00 eq.) and cyclobutanone (3.35 mL, 45.0 mmol, 3.00 eq.) in EtOH (45 mL) was added under N_2-atmosphere. It was stirred at 80 °C for 24 h. The solvent was removed under reduced pressure. The desired product was obtained *via* automated FC (CyH:EtOAc; 95:5) as colorless oil (1.15 g, 7.25 mmol, 48%).

^{1}H NMR (400 MHz, $CDCl_3$): δ = 7.55 – 7.49 (m, 2H), 7.45 – 7.38 (m, 3H), 7.04 (t, J = 2.8 Hz, 1H), 3.21 – 3.11 (m, 2H), 3.06 – 2.96 (m, 2H). **^{13}C NMR (101 MHz, $CDCl_3$)**: δ = 199.8, 146.3, 134.7, 130.2, 130.2, 129.1, 126.6, 45.9, 23.7. Spectroscopic data was in agreement with that previously reported.[12]

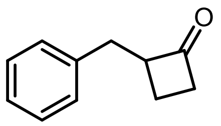

2-Benzylcyclobutan-1-one [S26]

A flask was charged with Pd/C (5% *w/w*, 345 mg, 0.16 mmol, 2.35 mol%) and purged with N_2. Cyclobutanone **S25** (1.09 g, 6.90 mmol, 1.00 eq.) was dissolved in dry THF/EtOH (4:1) and added to the flask *via* syringe. Then, the reaction mixture was purged with H_2 for ~15 min using a H_2-balloon. Thereafter, the mixture was stirred for 6 h at rt under H_2-atmosphere. The mixture was filtered through Celite® with EtOAc and concentrated *in vacuo*. The crude residue was purified *via* automated FC (CyH:EtOAc; 98:2→92:8). The product was obtained as colorless oil (613 mg, 3.83 mmol, 55%).

^{1}H NMR (400 MHz, $CDCl_3$): δ = 7.33 – 7.27 (m, 2H), 7.25 – 7.16 (m, 3H), 3.67 – 3.54 (m, 1H), 3.10 – 2.99 (m, 2H), 2.92 – 2.76 (m, 2H), 2.23 – 2.11 (m, 1H), 1.82 – 1.69 (m, 1H). **^{13}C NMR (101 MHz, $CDCl_3$)**: δ = 211.1, 139.0, 128.9, 128.6, 126.4, 61.3, 44.6, 35.3, 16.7. Spectroscopic data was in agreement with that previously reported.[13]

2.4 Syntheses of methylenecyclobutanes

Methylenecyclobutane (MCB) **1q** (CAS [15760-35-7]) was commercially available.

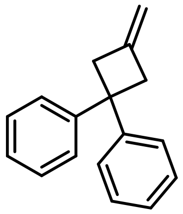

(3-Methylenecyclobutane-1,1-diyl)dibenzene [1a]

Following general procedure **A** using cyclobutanone **S17** (881 mg, 4.00 mmol, 1.00 eq.) the desired product was obtained *via* FC (pentane:Et_2O; 20:1) as colorless oil (690 mg, 3.132 mmol, 78%).

^{1}H NMR (400 MHz, $CDCl_3$): δ = 7.36 – 7.26 (m, 8H), 7.24 – 7.14 (m, 2H), 4.91 (p, J = 2.4 Hz, 2H), 3.47 (t, J = 2.4 Hz, 4H). **^{13}C NMR (101 MHz, $CDCl_3$)**: δ = 149.2, 144.0, 128.4, 126.6, 125.9, 106.7, 46.7, 46.2. Spectroscopic data was in agreement with that previously reported.[14]

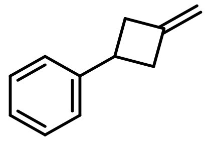

(3-Methylenecyclobutyl)benzene [1b]

Following general procedure **A** using cyclobutanone **S6** (717.0 mg, 4.90 mmol, 1.00 eq.) the desired product was obtained *via* FC (pentane) as colorless oil (152.2 mg, 1.055 mmol, 22%).

^{1}H NMR (400 MHz, $CDCl_3$): δ = 7.38 – 7.25 (m, 4H), 7.24 – 7.19 (m, 1H), 4.85 (dt, J = 4.5, 2.2 Hz, 2H), 3.55 (*app* p, $J \approx$ 8.4 Hz, 1H), 3.18 – 3.06 (m, 2H), 2.94 – 2.82 (m, 2H). **^{13}C NMR (101 MHz, $CDCl_3$)**: δ = 146.2, 145.8, 128.5, 126.6, 126.1, 105.8, 39.9, 35.0. Spectroscopic data was in agreement with that previously reported.[15]

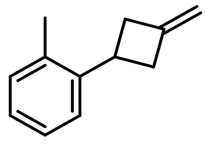

1-Methyl-2-(3-methylenecyclobutyl)benzene [1c]

Following general procedure **A** using cyclobutanone **S7** (160.2 mg, 1.00 mmol, 1.00 eq.) the desired product was obtained *via* FC (pentane) as colorless oil (80.0 mg, 0.506 mmol, 51%).

IR (neat): $\tilde{\nu}$ = 3072 (w), 3021 (w), 2956 (m), 2915 (m), 1678 (m), 1605 (w), 1489 (m), 1459 (m), 1417 (w), 1380 (w), 1330 (w), 1111 (w), 1058 (w), 1030 (w), 872 (s), 751 (s), 721 (s). **^{1}H NMR (599 MHz, $CDCl_3$)**: δ = 7.32 (d, J = 7.6 Hz, 1H), 7.25 – 7.20 (m, 1H), 7.20 – 7.12 (m, 2H), 4.86 (dq, J = 4.8, 2.2 Hz, 2H), 3.67 (*app* p, $J \approx$ 8.6 Hz, 1H), 3.14 – 3.06 (m, 2H), 2.93 – 2.85 (m, 2H), 2.29 (s, 3H). **^{13}C NMR (151 MHz, $CDCl_3$)**: δ = 146.1, 142.8, 136.2, 130.2, 126.1, 126.1, 125.2, 105.4, 38.5, 32.9, 19.8. **HRMS (EI)**: Calculated for $C_{12}H_{14}^{+}$ $[M]^{+}$: 158.1090, found: 158.1091.

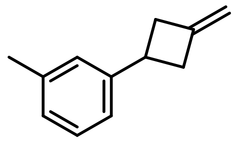

1-Methyl-3-(3-methylenecyclobutyl)benzene [1d]

Following general procedure **A** using cyclobutanone **S8** (160.2 mg, 1.00 mmol, 1.00 eq.) the desired product was obtained *via* FC (pentane) as colorless oil (97.0 mg, 0.613 mmol, 61%).

IR (neat): $\tilde{\nu}$ = 3018 (w), 3955 (m), 2915 (m), 1678 (m), 1608 (m), 1489 (m), 1442 (w), 1412 (w), 1331 (w), 1094 (w), 1039 (w), 872 (s), 818 (w), 778 (s), 698 (s). **^{1}H NMR (599 MHz, CDCl$_3$)**: δ = 7.23 (t, J = 7.5 Hz, 1H), 7.13 – 7.08 (m, 2H), 7.04 (d, J = 7.5 Hz, 1H), 4.86 (*app* p, $J \approx$ 2.3 Hz, 2H), 3.52 (*app* p, $J \approx$ 8.4 Hz, 1H), 3.15 – 3.08 (m, 2H), 2.92 – 2.84 (m, 2H), 2.37 (s, 3H). **^{13}C NMR (151 MHz, CDCl$_3$)**: δ = 146.3, 145.8, 138.0, 128.4, 127.4, 126.9, 123.6, 105.7, 39.8, 34.9, 21.6. **HRMS (EI)**: Calculated for $C_{12}H_{14}^+$ $[M]^+$: 158.1090, found: 158.1089.

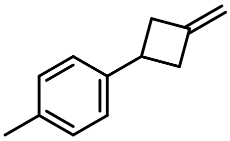

1-Methyl-4-(3-methylenecyclobutyl)benzene [1e]

Following general procedure **A** using cyclobutanone **S9** (160.2 mg, 1.00 mmol, 1.00 eq.) the desired product was obtained *via* FC (pentane) as colorless oil (137.0 mg, 0.866 mmol, 87%).

IR (neat): $\tilde{\nu}$ = 2954 (m), 2914 (m), 1675 (w), 1515 (m), 1438 (w), 1409 (w), 1110 (w), 1021 (w), 872 (s), 807 (s), 717 (w). **^{1}H NMR (599 MHz, CDCl$_3$)**: δ = 7.20 (d, J = 7.9 Hz, 2H), 7.15 (d, J = 7.9 Hz, 2H), 4.86 (*app* p, $J \approx$ 2.4 Hz, 2H), 3.52 (*app* p, $J \approx$ 8.4 Hz, 1H), 3.15 – 3.07 (m, 2H), 2.92 – 2.82 (m, 2H), 2.36 (s, 3H). **^{13}C NMR (151 MHz, CDCl$_3$)**: δ = 146.4, 142.8, 135.6, 129.2, 126.5, 105.7, 40.0, 34.7, 21.1. **HRMS (EI)**: Calculated for $C_{12}H_{14}^+$ $[M]^+$: 158.1090, found: 158.1089.

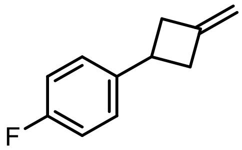

1-Fluoro-4-(3-methylenecyclobutyl)benzene [1f]

Following general procedure **A** using cyclobutanone **S10** (164.2 mg, 1.00 mmol, 1.00 eq.) the desired product was obtained *via* FC (pentane) as colorless oil (83.0 mg, 0.511 mmol, 51%).

IR (neat): $\tilde{\nu}$ = 3074 (w), 2957 (w), 2915 (w), 1678 (m), 1605 (m), 1509 (s), 1438 (w), 1410 (w), 1332 (w), 1226 (s), 1158 (m), 1096 (m), 1015 (w), 876 (s), 825 (s), 725 (w). **^{1}H NMR (599 MHz, CDCl$_3$)**: δ = 7.26 – 7.20 (m, 2H), 7.04 – 6.97 (m, 2H), 4.85 (*app* p, $J \approx$ 2.4 Hz, 2H), 3.51 (*app* p, $J \approx$ 8.3 Hz, 1H), 3.11 (ddq, J = 15.2, 8.6, 2.1 Hz, 2H), 2.86 – 2.78 (m, 2H). **^{13}C NMR (151 MHz, CDCl$_3$)**: δ = 161.4 (d, J = 243.7 Hz), 145.8, 141.5

(d, J = 3.2 Hz), 128.0 (d, *J* = 7.8 Hz), 115.2 (d, *J* = 21.2 Hz), 106.1, 40.0, 34.4. **^{19}F NMR (564 MHz, CDCl$_3$)** δ = −117.45. **HRMS (EI)**: Calculated for $C_{11}H_{11}F^+$ $[M]^+$: 163.0838, found: 163.0839.

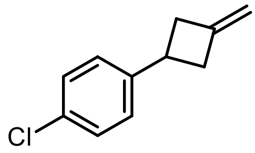

1-Chloro-4-(3-methylenecyclobutyl)benzene [1g]

Following general procedure **A** using cyclobutanone **S11** (180.6 mg, 1.00 mmol, 1.00 eq.) the desired product was obtained *via* FC (pentane) as colorless oil (134.0 mg, 0.750 mmol, 75%).

IR (neat): $\tilde{\nu}$ = 3075 (w), 2956 (w), 2914 (w), 1679 (m), 1510 (w), 1491 (s), 1436 (w), 1408 (w), 1331 (w), 1091 (s), 1014 (s), 875 (s), 815 (s), 752 (w), 711 (m), 689 (w). **^{1}H NMR (599 MHz, CDCl$_3$)**: δ = 7.28 (d, *J* = 8.4 Hz, 2H), 7.24 – 7.18 (m, 2H), 4.86 (*app* p, *J* ≈ 2.4 Hz, 2H), 3.50 (*app* p, *J* ≈ 8.3 Hz, 1H), 3.12 (*app* ddq, *J* ≈ 15.1, 8.7, 2.1 Hz, 2H), 2.86 – 2.77 (m, 2H). **^{13}C NMR (151 MHz, CDCl$_3$)**: δ = 145.6, 144.3, 131.8, 128.6, 128.0, 106.2, 39.8, 34.5. **HRMS (EI)**: Calculated for $C_{11}H_{10}Cl^-$ $[M\text{-}H]^-$: 177.0466, found: 177.0465.

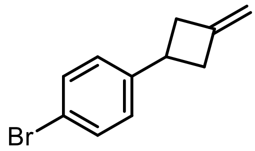

1-Bromo-4-(3-methylenecyclobutyl)benzene [1h]

Following general procedure **A** using cyclobutanone **S12** (225.1 mg, 1.00 mmol, 1.00 eq.) the desired product was obtained *via* FC (pentane) as colorless oil (156.0 mg, 0.699 mmol, 70%).

IR (neat): $\tilde{\nu}$ = 3074 (w), 2955 (w), 2913 (w), 1678 (m), 1592 (w), 1487 (s), 1436 (w), 1418 (w), 1398 (m), 1330 (w), 1203 (w), 1101 (w), 1073 (m), 1009 (s), 960 (w), 875 (s), 812 (s), 750 (w), 710 (m), 677 (w). **^{1}H NMR (599 MHz, CDCl$_3$)**: δ = 7.46 – 7.40 (m, 2H), 7.19 – 7.12 (m, 2H), 4.85 (*app* p, *J* ≈ 2.4 Hz, 2H), 3.48 (*app* p, *J* ≈ 8.3 Hz, 1H), 3.11 (dddd, *J* = 17.1, 8.8, 4.2, 2.1 Hz, 2H), 2.85 – 2.77 (m, 2H). **^{13}C NMR (151 MHz, CDCl$_3$)**: δ = 145.6, 144.8, 131.5, 128.4, 119.8, 106.2, 39.8, 34.5. **HRMS (EI)**: Calculated for $C_{11}H_{11}Br^+$ $[M]^+$: 224.0019, found: 224.0018.

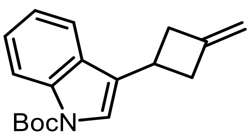

tert-Butyl 3-(3-methylenecyclobutyl)-1*H*-indole-1-carboxylate [1i]

Following general procedure **A** using cyclobutanone **S18** (428.0 mg, 1.50 mmol, 1.00 eq.) the desired product was obtained *via* FC (pentane:Et_2O; 100:1) as colorless oil (265.3 mg, 0.936 mmol, 62%).

IR (neat): $\tilde{\nu}$ = 3071 (w), 2979 (w), 2917 (w), 1727 (s), 1678 (w), 1610 (w), 1570 (w), 1476 (m), 1452 (s), 1368 (s), 1339 (m), 1320 (m), 1308 (m), 1298 (m), 1249 (s), 1219 (m), 1152 (s), 1086 (s), 1043 (m), 1017 (m), 935 (w), 872 (m), 852 (m), 835 (w), 800 (w), 763 (s), 742 (s), 660 (w). **^{1}H NMR (599 MHz, $CDCl_3$)**: δ = 8.13 (s, 1H), 7.51 (d, *J* = 7.8 Hz, 1H), 7.40 (s, 1H), 7.35 – 7.29 (m, 1H), 7.25 – 7.20 (m, 1H), 4.87 (m, 2H), 3.67 (*app* tt, *J* ≈ 8.8, 7.5 Hz, 1H), 3.19 (*app* ddp, *J* ≈ 14.3, 8.5, 1.8 Hz, 2H), 2.98 – 2.89 (m, 2H), 1.68 (s, 9H). **^{13}C NMR (151 MHz, $CDCl_3$)**: δ = 150.0, 146.5, 136.0, 130.1, 125.1, 124.5, 122.5, 121.5, 119.5, 115.5, 106.4, 83.5, 38.5, 28.4, 26.6. **HRMS (ESI)**: Calculated for $C_{18}H_{21}NO_2Na^+$ [M+Na]$^+$: 306.1465, found: 306.1465.

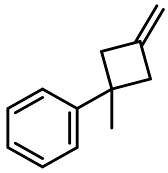

(1-Methyl-3-methylenecyclobutyl)benzene [1j]

Following general procedure **A** using cyclobutanone **S13** (1.602 g, 10.00 mmol, 1.00 eq.) the desired product was obtained *via* FC (pentane) as colorless oil (1.429 g, 9.030 mmol, 90%).

IR (neat): $\tilde{\nu}$ = 3060 (w), 3025 (w), 2953 (m), 2913 (w), 1677 (m), 1603 (w), 1495 (m), 1445 (m), 1412 (w), 1371 (w), 1304 (w),1093 (w), 1077 (w), 1028 (m), 906 (w), 874 (s), 761 (s), 697 (s). **^{1}H NMR (400 MHz, $CDCl_3$)**: δ = 7.37 – 7.29 (m, 2H), 7.25 – 7.22 (m, 2H), 7.22 – 7.16 (m, 1H), 4.89 (*app* tt, *J* ≈ 2.7, 2.0 Hz, 2H), 3.13 – 3.03 (m, 2H), 2.77 – 2.67 (m, 2H), 1.48 (s, 3H). **^{13}C NMR (101 MHz, $CDCl_3$)**: δ = 150.9, 144.9, 128.3, 125.6, 125.3, 107.2, 44.9, 38.7, 30.5. **HRMS (EI)**: Calculated for $C_{12}H_{13}^-$ [M-H]$^-$: 157.1012, found: 157.1009.

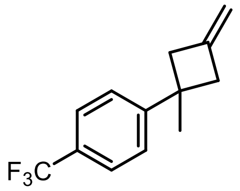

1-(1-Methyl-3-methylenecyclobutyl)-4-(trifluoromethyl)benzene [1k]

Following general procedure **A** using cyclobutanone **S19** (500.0 mg, 2.191 mmol, 1.00 eq.) the desired product was obtained *via* FC (pentane) as colorless oil (422.6 mg, 1.868 mmol, 85%).

IR (neat): $\tilde{\nu}$ = 2958 (w), 2919 (w), 2868 (w), 1680 (w), 1619 (m), 1455 (w), 1410 (m), 1373 (w), 1324 (s), 1300 (m), 1235 (w), 1163 (s), 1120 (s), 1106 (s), 1092 (s), 1080 (s), 1064 (s), 1036 (w), 1015 (m), 953 (w), 879 (m), 839 (s), 773 (w), 724 (w), 671 (m). **^{1}H NMR (599 MHz, CDCl$_3$)**: δ = 7.62 – 7.56 (m, 2H), 7.37 – 7.31 (m, 2H), 4.92 (*app* tt, *J* ≈ 2.7, 2.1 Hz, 2H), 3.12 – 3.04 (m, 2H), 2.77 (*app* dq, *J* ≈ 15.3, 2.0 Hz, 2H), 1.50 (s, 3H). **^{13}C NMR (151 MHz, CDCl$_3$)**: δ = 154.9, 143.8, 128.0 (q, *J* = 32.3 Hz), 125.9 – 125.6 (m), 125.4, 124.5 (q, *J* = 271.7 Hz), 107.8, 44.8, 38.9, 30.3 (d, *J* = 3.7 Hz). **^{19}F NMR (377 MHz, CDCl$_3$)**: δ = −62.28. **HRMS (EI)**: Calculated for $C_{13}H_{13}F_3^+$ [M]$^+$: 226.0964, found: 226.0966.

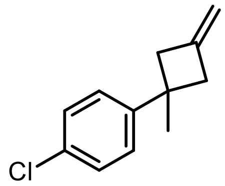

1-Chloro-4-(1-methyl-3-methylenecyclobutyl)benzene [1l]

Following general procedure **A** using cyclobutanone **S20** (584.0 g, 3.00 mmol, 1.00 eq.) the desired product was obtained *via* FC (pentane) as colorless oil (413.6 mg, 2.146 mmol, 72%).

IR (neat): $\tilde{\nu}$ = 3076 (w), 2955 (m), 2913 (m), 2865 (w), 1895 (w), 1771 (w), 1678 (m), 1494 (s), 1452 (m), 1399 (m), 1372 (m), 1299 (m), 1226 (w), 1194 (w), 1095 (s), 1013 (s), 876 (s), 824 (s), 739 (m), 720 (m), 695 (m). **^{1}H NMR (599 MHz, CDCl$_3$)**: δ = 7.30 – 7.26 (m, 2H), 7.19 – 7.13 (m, 2H), 4.89 (*app* ddd, *J* ≈ 4.7, 2.4, 1.4 Hz, 2H), 3.07 – 2.99 (m, 2H), 2.72 (*app* dq, *J* ≈ 15.3, 2.0 Hz, 2H), 1.46 (s, 3H). **^{13}C NMR (151 MHz, CDCl$_3$)**: δ = 149.3, 144.2, 131.4, 128.4, 126.9, 107.5, 44.9, 38.4, 30.3. **HRMS (EI)**: Calculated for $C_{12}H_{13}Cl^+$ [M]$^+$: 192.0700, found: 192.0705.

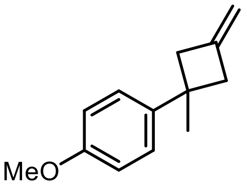

1-Methoxy-4-(1-methyl-3-methylenecyclobutyl)benzene [1m]

Following general procedure **A** using cyclobutanone **S21** (476.6 mg, 2.50 mmol, 1.00 eq.) the desired product was obtained *via* FC (pentane) as colorless oil (371.5 mg, 1.973 mmol, 79%).

IR (neat): $\tilde{\nu}$ = 3073 (w), 3034 (w), 2986 (w), 2952 (m), 2911 (m), 2834 (w), 1677 (m), 1612 (m), 1582 (w), 1512 (s), 1464 (m), 1455 (m), 1442 (m), 1410 (m), 1371 (w), 1302 (m), 1244 (s), 1177 (s), 1106 (m), 1093 (m), 1034 (s), 1012 (m), 874 (s), 808 (m), 789 (m), 730 (w), 715 (w). **^{1}H NMR (599 MHz, $CDCl_3$)**: δ = 7.19 – 7.15 (m, 2H), 6.90 – 6.85 (m, 2H), 4.91 – 4.86 (m, 2H), 3.81 (s, 3H), 3.05 (*app* dq, *J* ≈ 13.4, 3.0 Hz, 2H), 2.74 – 2.67 (m, 2H), 1.47 (s, 3H). **^{13}C NMR (151 MHz, $CDCl_3$)**: δ = 157.4, 144.8, 142.9, 126.2, 113.6, 107.0, 55.3, 45.0, 37.9, 30.3. **HRMS (EI)**: Calculated for $C_{13}H_{16}O^+$ $[M]^+$: 188.1196, found: 188.1196.

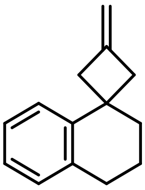

3-Methylene-3',4'-dihydro-2'*H*-spiro[cyclobutane-1,1'-naphthalene] [1n]

Following general procedure **A** using cyclobutanone **S14** (372.6 mg, 2.00 mmol, 1.00 eq.) the desired product was obtained *via* FC (pentane) as colorless oil (284.0 mg, 1.541 mmol, 77%).

IR (neat): $\tilde{\nu}$ = 3071 (w), 3016 (w), 2984 (w), 2917 (m), 2857 (w), 1675 (m), 1604 (w), 1489 (m), 1451 (m), 1434 (m), 1409 (m), 1339 (w), 1281 (w), 1043 (w), 1010 (w), 955 (w), 929 (w), 873 (s), 783 (w), 752 (s), 726 (s). **^{1}H NMR (599 MHz, $CDCl_3$)**: δ = 7.59 (dd, *J* = 7.8, 1.3 Hz, 1H), 7.22 (dddt, *J* = 7.9, 7.2, 1.5, 0.7 Hz, 1H), 7.12 (td, *J* = 7.4, 1.3 Hz, 1H), 7.07 (ddd, *J* = 7.6, 1.6, 0.7 Hz, 1H), 5.00 – 4.94 (m, 2H), 3.04 (ddt, *J* = 14.7, 4.9, 2.6 Hz, 2H), 2.81 (t, *J* = 6.4 Hz, 2H), 2.75 – 2.67 (m, 2H), 1.99 – 1.93 (m, 2H), 1.83 – 1.75 (m, 2H). **^{13}C NMR (151 MHz, $CDCl_3$)**: δ = 145.5, 144.2, 136.6, 128.9, 126.4, 126.2, 125.8, 107.7, 47.3, 37.2, 37.0, 30.6, 20.5. **HRMS (EI)**: Calculated for $C_{14}H_{16}^+$ $[M]^+$: 184.1247, found: 184.1246.

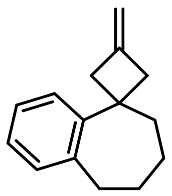

3'-Methylene-6,7,8,9-tetrahydrospiro[benzo[7]annulene-5,1'-cyclobutane] [1o]

Following general procedure **A** using 6,7,8,9-tetrahydro-5*H*-benzo[7]annulen-5-one (1.070 g, 6.68 mmol, 1.00 eq.) the desired product 5-methylene-6,7,8,9-tetrahydro-5H-benzo[7]annulene was obtained and used in the next step.

Following general procedure **B** using 5-methylene-6,7,8,9-tetrahydro-5*H*-benzo[7]annulene (474.7 mg, 3.00 mmol, 1.00 eq.) the desired product 6,7,8,9-tetrahydrospiro[benzo[7]annulene-5,1'-cyclobutan]-3'-one was obtained and used in the next step.

Following general procedure **A** using 6,7,8,9-tetrahydrospiro[benzo[7]annulene-5,1'-cyclobutan]-3'-one (372.6 mg, 2.00 mmol, 1.00 eq.) the desired product was obtained *via* FC (pentane) as colorless oil (284.0 mg, 1.541 mmol, 38% over 3 steps).

IR (neat): $\tilde{\nu}$ = 3070 (w), 3017 (w), 2921 (m), 2854 (m), 1680 (m), 1600 (w), 1488 (m), 1450 (m), 1412 (m), 1368 (w), 1350 (w), 1316 (w), 1254 (w), 1112 (w), 1084 (w), 1041 (m), 1010 (w), 933 (m), 908 (w), 871 (s), 794 (w), 757 (s), 745 (s), 711 (m). **^{1}H NMR (599 MHz, CDCl$_3$)**: δ = 7.22 (dd, *J* = 7.6, 1.5 Hz, 1H), 7.17 (td, *J* = 7.4, 1.7 Hz, 1H), 7.11 (td, *J* = 7.2, 1.5 Hz, 1H), 7.06 (dd, *J* = 7.3, 1.6 Hz, 1H), 4.86 (tt, *J* = 2.7, 1.9 Hz, 2H), 3.09 – 3.01 (m, 2H), 2.88 (*app* dq, *J* ≈ 14.5, 1.9 Hz, 2H), 2.73 – 2.67 (m, 2H), 1.96 – 1.89 (m, 2H), 1.86 – 1.80 (m, 2H), 1.61 – 1.55 (m, 2H). **^{13}C NMR (151 MHz, CDCl$_3$)**: δ = 147.5, 145.4, 142.9, 129.6, 126.1, 125.8, 125.2, 106.0, 43.0, 42.2, 36.7, 35.4, 27.6, 27.5. **HRMS (EI)**: Calculated for $C_{15}H_{18}^+$ [M]$^+$: 198.1403, found: 198.1402.

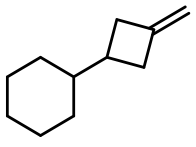

(3-Methylenecyclobutyl)cyclohexane [1p]

Following procedure **A** using cyclobutanone **S15** (90.0 mg, 0.591 mmol, 1.00 eq.) the desired product was obtained *via* FC (pentane) as colorless oil (58.8 mg, 0.391 mmol, 66%).

IR (neat): $\tilde{\nu}$ = 3073 (w), 2986 (w), 2920 (s), 2850 (s), 2158 (w), 2027 (w), 1976 (w), 1751 (w), 1676 (m), 1514 (w), 1495 (w), 1448 (m), 1419 (w), 1343 (w), 1283 (w), 1262 (w), 1242 (w), 1181 (w), 1031 (w), 985 (w), 871 (s), 828 (w), 753 (m), 698 (m), 673 (w). **^{1}H NMR (599 MHz, $CDCl_3$)**: δ = 4.73 – 4.68 (m, 2H), 2.67 (dddd, *J* = 16.6, 8.4, 4.1, 2.1 Hz, 2H), 2.40 – 2.30 (m, 2H), 1.92 (dddd, *J* = 15.8, 9.8, 8.4, 7.4 Hz, 1H), 1.74 – 1.67 (m, 4H), 1.65 (dtt, *J* = 11.3, 3.2, 1.5 Hz, 1H), 1.26 – 1.07 (m, 4H), 0.79 (dddd, *J* = 13.9, 11.6, 9.2, 2.2 Hz, 2H). **^{13}C NMR (151 MHz, $CDCl_3$)**: δ = 147.8, 104.9, 44.1, 36.6, 36.2, 30.4, 26.7, 26.2. **HRMS (EI)**: Calculated for $C_{11}H_{18}^+$ [M]$^+$: 150.1403, found: 150.1404.

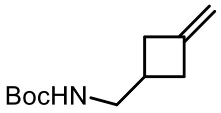

tert-Butyl ((3-methylenecyclobutyl)methyl)carbamate [1r]

To a solution of MCB **1q** (931.3 mg, 1.021 ml, 10.00 mmol, 1.00 eq.) in dry THF (50 ml) under argon atmosphere $LiAlH_4$ (759 mg, 20.0 mmol, 2.00 eq.) was added in small portions at 0 °C. The reaction mixture was stirred for 16 h at rt and H_2O (20 ml) was added. The phases were separated and the aq. layer was extracted with Et_2O (3 × 20 ml). The combined org. layers were dried over $MgSO_4$ and concentrated *in vacuo*. The crude product was used in the next step without further purification.

Following a procedure by *Achaogen Inc.*[16] using (3-methylenecyclobutyl)-methanamine (194.3 mg, 2.00 mmol, 1.00 eq.) the desired product was obtained *via* FC (pentane:Et_2O; 20:1→10:1) as colorless oil (193.8 mg, 0.982 mmol, 30% over two steps).

^{1}H NMR (400 MHz, $CDCl_3$): δ = 4.78 (*app* p, *J* ≈ 2.3 Hz, 2H), 4.63 – 4.47 (br s, 1H), 3.22 (br d, *J* = 6.5 Hz, 2H), 2.84 – 2.72 (m, 2H), 2.50 – 2.31 (m, 3H), 1.46 (s, 9H). **^{13}C NMR (101 MHz, $CDCl_3$)**: δ = 156.2, 146.2, 106.7, 79.3, 45.6, 35.4, 30.1, 28.5. Spectroscopic data was in agreement with that previously reported.[16]

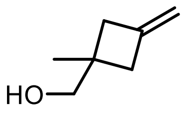

(1-Methyl-3-methylenecyclobutyl)methanol [S27]

In analogy to a procedure by *Pfizer Inc.*,[17] MCB **1q** (0.51 mL, 5.0 mmol, 1.00 eq.) was dissolved in dry Et_2O (3.5 mL) and cooled to −78 °C. Then, lithium bis(trimethylsilyl)

amide solution (1 M in THF, 5.50 mL, 5.50 mmol, 1.10 eq.) was added dropwise and it was stirred for 1 h at −78 °C. Thereafter, methyl iodide (0.37 mL, 6.00 mmol, 1.20 eq.) was added and the mixture was gradually warmed to −40 °C under stirring for 3 h. Subsequently, sat. aq. NH_4Cl sol. (5 mL) was added. The phases were separated and the aq. layer was extracted with Et_2O (3 × 15 mL). The combined org. phases were dried over Na_2SO_4, filtered and the solvent was removed *in vacuo*. The crude product was filtered through a plug of silica with Et_2O, concentrated and used in the next step without further purification.

The crude carbonitrile was dissolved in a mixture of EtOH/H_2O (6 mL, 1:1 *v*/*v*) and potassium hydroxide (701 mg, 12.5 mmol, 2.50 eq.) was added. The mixture was heated to reflux for 16 h. Then, it was cooled to <10 °C and acidified to pH <1 by addition of conc. aq. HCl. The phases were separated and the aq. layer was extracted with EtOAc (3 × 10 mL). The combined org. layers were dried over $MgSO_4$, filtered and concentrated *in vacuo*. 1-methyl-3-methylenecyclobutane-1-carboxylic acid was obtained as colorless oil and used in the next step without further purification.

In analogy to a procedure of *Cowling and Goodby*,[18] $LiAlH_4$ (303 mg, 7.99 mmol, 2.00 eq.) was added to a solution of the acid (504 mg, 4.00 mmol, 1.00 eq.) in dry Et_2O (60 mL) in a single portion. It was heated to reflux for 3.5 h. Thereafter, it was cooled to <10 °C and sat. aq. Na_2SO_4 (10 mL) was added. It was stirred for additional 16 h before the resulting precipitate was filtered off and washed with Et_2O (20 mL). The filtrate was dried over $MgSO_4$, filtered and the solvent was removed *in vacuo*. The desired product was obtained *via* filtration through a plug of silica with Et_2O as mixture with Et_2O (460 mg, 90% *w*/*w*, 3.67 mmol, 73%) and was used as is in the ring expansion. *Note:* The product is fairly volatile.

^{1}H NMR (400 MHz, $CDCl_3$): δ = 4.82 (p, J = 2.4 Hz, 2H), 3.53 (s, 2H), 2.61 – 2.46 (m, 2H), 2.40 – 2.28 (m, 2H), 1.52 – 1.42 (m, 1H), 1.20 (s, 3H). **^{13}C NMR (101 MHz, $CDCl_3$)**: δ = 144.6, 107.7, 70.6, 40.3, 35.7, 29.8, 23.5. Spectroscopic data was in agreement with that previously reported.[17]

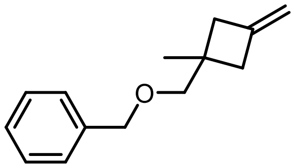

(((1-Methyl-3-methylenecyclobutyl)methoxy)methyl)benzene [1s]

In an oven dried Schlenk tube sodium hydride (29 mg, 0.72 mmol, 1.10 eq.) was suspended in *N*,*N*-dimethylformamide (5 mL) and cooled to 0 °C. Then, MCB **S27** (73 mg, 0.65 mmol, 1.00 eq.) dissolved in *N*,*N*-dimethylformamide (2 mL) was added dropwise. After stirring at 0 °C for approx. 5 min the mixture was allowed to warm to rt and stirred for additional 30 min. It was cooled again to 0 °C and benzyl bromide (0.09 mL, 0.72 mmol, 1.10 eq.) was added. The mixture was allowed to warm to rt and stirred for 3 h. The progress of the reaction was observed *via* TLC control (CH_2Cl_2:MeOH; 98:2). Then, sat. aq. NH_4Cl sol. (2 mL) was added and it was diluted with water (10 mL). It was extracted with dichloromethane (4 × 10 mL). The comb. org. layers were washed with brine (10 mL), dried over $MgSO_4$, filtered and the solvent was removed under reduced pressure. The residue, still containing larger amounts of DMF, was taken up in Et_2O (10 mL) and washed with aq. LiCl sol. (3 × 15 mL). The org. phase was dried over $MgSO_4$, filtered and the solvent was removed under reduced pressure. The desired product was obtained *via* automated FC (pentane:EtOAc; 100:0→95:5) as mixture with Bn_2O (58 mg, 72% *w*/*w*, 0.21 mmol, 32%) and was used as is in the ring expansion.

IR (neat): $\tilde{\nu}$ = 2952 (m), 2913 (m), 2855 (w), 2332 (w), 1676 (w), 1497 (w), 1454 (w), 1358 (w), 1162 (w), 1095 (m), 1028 (w), 908 (w), 876 (m), 811 (w), 739 (s), 697 (s), 649 (m), 634 (m), 619 (s), 609 (s), 590 (m). **^{1}H NMR (400 MHz, $CDCl_3$)**: δ = 7.40–7.26 (m, 7H)[1], 4.80 (p, J = 2.4 Hz, 2H), 4.57 (s, Bn_2O), 4.56 (s, 2H), 3.35 (s, 2H), 2.65–2.55 (m, 2H), 2.37–2.28 (m, 2H), 1.22 (s, 3H). **^{13}C NMR (101 MHz, $CDCl_3$)**: δ = 145.3, 139.0, 138.4 (Bn_2O), 128.6 (Bn_2O), 128.5, 128.0 (Bn_2O), 127.8 (Bn_2O), 127.6, 127.6, 107.4, 77.9, 73.4, 72.3 (Bn_2O), 41.0, 34.8, 24.1. **HRMS (APCI, CH_3COONH_4)**: Calculated for $C_{14}H_{19}O^+$ [M+H]$^+$: 203.1430, found 203.1430.

[1] The increased integral in the aromatic region is caused by overlap with the signals of residual Bn_2O.

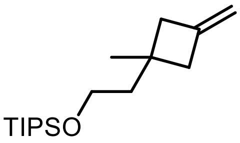

Triisopropyl(2-(1-methyl-3-methylenecyclobutyl)ethoxy)silane [1t]

A tube was charged with MCB **1u** (100 mg, 0.73 mmol, 1.00 eq.) and DMF (2 mL). Imidazole (124 mg, 1.82 mmol, 2.50 eq.) was added and the tube was set under N_2-atmosphere. Then, chloro(triisopropyl)silane (0.22 mL, 1.03 mmol, 1.41 eq.) was added *via* syringe and the mixture was stirred at rt for 4 h. The progress of the reaction was observed *via* TLC control (pentane:EtOAc; 50:50). It was diluted with CH_2Cl_2 (3 mL) and the org. phase was washed with sat. aq. LiCl sol. (4 × 5 mL). The comb. org. layers were dried over $MgSO_4$, filtered and concentrated *in vacuo*. The residue was taken up in CH_2Cl_2 and filtered through a short silica plug, which was eluted with CH_2Cl_2, to afford the product as colorless oil (168 mg, 0.60 mmol, 82%).

IR (neat): $\tilde{\nu}$ = 2942 (s), 2868 (s), 2360 (w), 1739 (m), 1678 (m), 1466 (m), 1383 (w), 1292 (w), 1245 (m), 1204 (w), 1098 (s), 1071 (m), 997 (m), 949 (w), 878 (s), 781 (m), 758 (w), 740 (s), 680 (s). **^{1}H NMR (400 MHz, CDCl$_3$)**: δ = 4.78 (p, *J* = 2.4 Hz, 2H), 3.73 (t, *J* = 7.1 Hz, 2H), 2.58–2.49 (m, 2H), 2.38–2.26 (m, 2H), 1.75 (t, *J* = 7.1 Hz, 2H), 1.15 (s, 3H), 1.08–1.04 (m, 21H). **^{13}C NMR (101 MHz, CDCl$_3$)**: δ = 146.2, 106.9, 60.8, 44.3, 44.2, 32.9, 25.8, 18.2, 12.1. **HRMS (ESI)**: Calculated for $C_{17}H_{35}OSi^+$ $[M+H]^+$: 283.2452, found 283.2446.

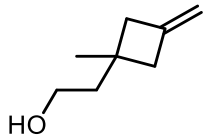

2-(1-Methyl-3-methylenecyclobutyl)ethan-1-ol [1u]

Following general procedure **A** using cyclobutanone **S22** (719 mg, 3.59 mmol, 1.00 eq), ethyl (2-(1-methyl-3-methylenecyclo-butyl)ethyl) carbonate was obtained after filtration through a silica plug (CyH:EtOAc; 95:5).

The crude carbonate was dissolved in MeOH (10 mL) and K_2CO_3 (78 mg, 0.56 mmol, 0.16 eq.) was added. The mixture was stirred at rt for 18 h. It was acidified to pH = 1 with 1 M aq. HCl and extracted with CH_2Cl_2 (4 × 15 mL). The combined org. phases were dried over $MgSO_4$, filtered and the solvent was removed *in vacuo*. The desired product was obtained after filtration through a plug of silica with CH_2Cl_2 as colorless oil (267 mg, 2.11 mmol, 59%).

IR (neat): $\tilde{\nu}$ = 3395 (br), 2954 (s), 2362 (m), 2342 (m), 2238 (w), 1746 (w), 1678 (m), 1461 (m), 1377 (m), 1332 (w), 1274 (w), 1147 (w), 1050 (s), 1015 (m), 908 (m), 876 (s), 814 (w), 728 (m), 645 (m). **^{1}H NMR (400 MHz, $CDCl_3$)**: δ = 4.79 (p, *J* = 2.4 Hz, 2H), 3.73 – 3.64 (m, 2H), 2.57 – 2.47 (m, 2H), 2.41 – 2.29 (m, 2H), 1.80 – 1.73 (m, 2H), 1.15 (s, 3H). **^{13}C NMR (101 MHz, $CDCl_3$)**: δ = 145.5, 107.2, 60.4, 44.1, 44.1, 32.8, 25.9. **HRMS (APCI, CH_3COONH_4)**: Calculated for $C_8H_{15}O^+$ $[M+H]^+$: 127.1118, found: 127.1114.

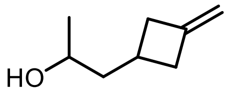

1-(3-Methylenecyclobutyl)propan-2-ol [1v]

Following general procedure **A** using cyclobutanone **S23** (202 mg, 1.01 mmol, 1.00 eq.) ethyl (1-(3-methylenecyclobutyl)propan-2-yl) carbonate was obtained as a colorless oil (171 mg, 0.86 mmol, 85%) which was used without further purification.

The crude carbonate (171 mg, 0.86 mmol, 1.00 eq.) was dissolved in methanol (5 mL) and potassium carbonate (30 mg, 0.22 mmol, 0.25 eq.) was added. The mixture was stirred at rt for 4 h. Then, 1 M aq. HCl (2 mL) and water (5 mL) were added and it was extracted with CH_2Cl_2 (4 × 5 mL). The comb. org. phases were dried over $MgSO_4$, filtered and concentrated *in vacuo*. The product was obtained as colorless oil (71 mg, 0.56 mmol, 65%) and used without further purification.

IR (neat): $\tilde{\nu}$ = 3389 (br), 2965 (s), 2913 (s), 1783 (w), 1732 (w), 1676 (m), 1461 (m), 1417 (m), 1375 (m), 1269 (m), 1135 (m), 1057 (m), 1003 (m), 939 (m), 873 (s), 833 (w), 705 (s), 672 (s), 661 (s), 638 (s), 626 (s). **^{1}H NMR (400 MHz, $CDCl_3$)**: δ = 4.72 (p, *J* = 2.3 Hz, 2H), 3.77 (dqd, *J* = 7.4, 6.2, 5.2 Hz, 1H), 2.87–2.71 (m, 2H), 2.46–2.27 (m, 3H), 1.68 (ddd, *J* = 13.8, 7.4, 6.5 Hz, 1H), 1.57 (ddd, *J* = 13.7, 7.7, 5.2 Hz, 1H), 1.50 (s, 1H), 1.18 (d, *J* = 6.2 Hz, 3H). **^{13}C NMR (101 MHz, $CDCl_3$)**: δ = 147.4, 105.6, 67.1, 46.1, 38.1, 38.0, 27.3, 23.8. **HRMS (ESI)**: Calculated for $C_8H_{15}O^+$ $[M+H]^+$: 127.1118, found 127.1117.

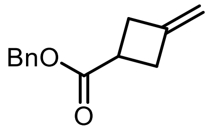

Benzyl 3-methylenecyclobutane-1-carboxylate [1w]

Following general procedure **A** using cyclobutanone **S16** (612.7 mg, 3.00 mmol, 1.00 eq.) the desired product was obtained *via* FC (pentane:Et_2O; 20:1) as colorless oil (242.1 mg, 1.197 mmol, 40%).

^{1}H NMR (400 MHz, $CDCl_3$): δ = 7.45 – 7.32 (m, 5H), 5.17 (s, 2H), 4.84 (*app* p, $J \approx 2.5$ Hz, 2H), 3.27 – 3.15 (m, 1H), 3.06 (*app* dddd, $J \approx 14.5, 7.9, 3.2, 1.5$ Hz, 2H), 2.99 – 2.89 (m, 2H). **^{13}C NMR (101 MHz, $CDCl_3$)**: δ = 175.0, 144.3, 136.1, 128.7, 128.4, 128.3, 107.1, 66.5, 35.6, 33.3. Spectroscopic data was in agreement with that previously reported.[19]

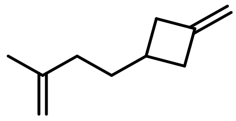

1-(3-Methylbut-3-en-1-yl)-3-methylenecyclobutane [1x]

Following general procedure **A** using cyclobutanone **S24** (294.2 g, 2.10 mmol, 1.00 eq.) the desired product was obtained *via* FC (pentane) as colorless oil (168.9 g, 1.240 mmol, 59%) with residues of pentane.

IR (neat): $\tilde{v}$ = 3074 (w), 2925 (s), 2851 (m), 1677 (m), 1650 (m), 1450 (m), 1416 (m), 1374 (m), 1200 (w), 884 (s), 872 (s), 761 (w). **^{1}H NMR (599 MHz, $CDCl_3$)**: δ = 4.73 (p, J = 2.3 Hz, 2H), 4.69 (dq, J = 2.2, 0.7 Hz, 1H), 4.66 (dt, J = 2.3, 1.2 Hz, 1H), 2.82 – 2.71 (m, 2H), 2.36 – 2.17 (m, 3H), 1.99 – 1.92 (m, 2H), 1.72 (s, J = 1.2 Hz, 3H), 1.64 – 1.55 (m, 2H), 1.25 – 1.18 (m, pentane), 0.89 (t, J = 7.1 Hz, pentane). **^{13}C NMR (151 MHz, $CDCl_3$)**: δ = 147.8, 146.1, 109.8, 105.6, 37.7, 35.9, 34.7, 34.3 (pentane), 30.2, 22.6, 22.5 (pentane), 14.2 (pentane). **HRMS (ESI Nanospray)**: Calculated for $C_{10}H_{16}Ag^+$ $[M+Ag]^+$: 243.0298, 245.0294; found: 243.0299, 245.0295.

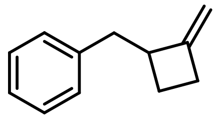

(2-Methylidenecyclobutyl)methylbenzene [5]

Following general procedure **A** using cyclobutanone **S26** (128 mg, 0.800 mmol, 1.00 eq.) the desired product was obtained *via* FC (pentane) as a colorless oil (78 mg, 0.49 mmol, 62%).

IR (neat): $\tilde{\nu}$ = 3484 (br), 3068 (w), 3025 (w), 2979 (w), 2921 (w), 2375 (w), 1673 (w), 1602 (w), 1496 (w), 1454 (w), 1158 (w), 1090 (w), 1031 (w), 905 (w), 874 (m), 806 (w), 740 (m), 697 (s), 650 (m), 635 (m), 618 (s), 602 (s), 588 (s). **^{1}H NMR (400 MHz, $CDCl_3$)**: δ = 7.35–7.24 (m, 2H), 7.24–7.15 (m, 3H), 4.73 (dq, *J* = 7.7, 2.4 Hz, 2H), 3.24 (tdd, *J* = 11.6, 5.8, 2.7 Hz, 1H), 2.95 (dd, *J* = 14.0, 6.1 Hz, 1H), 2.74 (dd, *J* = 14.0, 9.1 Hz, 1H), 2.70–2.52 (m, 2H), 2.07 (dtd, *J* = 10.8, 9.0, 5.0 Hz, 1H), 1.72 (dddd, *J* = 10.9, 9.3, 8.2, 7.3 Hz, 1H). **^{13}C NMR (101 MHz, $CDCl_3$)**: δ = 154.4, 140.7, 128.9, 128.4, 126.0, 103.9, 45.5, 40.5, 29.1, 24.0. **HRMS (APCI, CH_3COONH_4)**: Calculated for $C_{12}H_{15}^+$ $[M+H]^+$: 159.1168, found 159.1165.

2.5 Syntheses of other methylenecycloalkenes

1-bromo-4-(2-methylenecyclopropyl)benzene [**S28**] [20] and (4-methylenecyclohexyl)-benzene [**S29**] [21] were synthesized according to literature procedures.

2.6 Synthesis of ligand L1

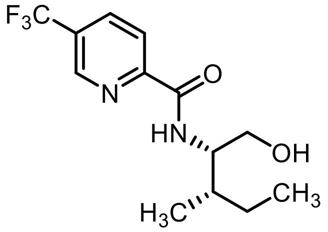

N-((2*S*,3*S*)-1-Hydroxy-3-methylpentan-2-yl)-5-(trifluoromethyl)picolinamide [S30]

Following a modified procedure of *Lutjen et al.*,[22] a flask was charged with 5-(trifluoromethyl)picolinic acid (573 mg, 3.00 mmol, 1.00 eq.), (*S*)-isoleucinol (352 mg, 3.00 mmol, 1.00 eq.), 1-ethyl-3-(3-dimethylaminopropyl)carbodiimide hydrochloride (633 mg, 3.30 mmol, 1.10 eq.) and *N*,*N*-dimethyl aminopyridine (37 mg, 0.30 mmol,

0.1 eq.). Then, CH_2Cl_2 (12 mL) was added and the mixture was stirred at rt for 18 h. Water was added and the phases were separated. The aq. layer was extracted with CH_2Cl_2 (10 mL). The combined org. phases were washed with sat. aq. $NaHCO_3$ sol. (15 mL). The aq. layer was extracted with CH_2Cl_2 (10 mL). The combined org. phases were washed with brine (15 mL), dried over $MgSO_4$, filtered and concentrated *in vacuo.* The desired product was obtained *via* automated FC (CyH:EtOAc; 80:20→20:80) as pale yellow oil (480 mg, 1.65 mmol, 55%).

IR (neat): $\tilde{\nu}$ = 3394 (br), 2968 (w), 2927 (w), 2877 (w), 2332 (w), 2241 (w), 1669 (m), 1578 (w), 1528 (s), 1465 (w), 1386 (w), 1326 (s), 1167 (s), 1134 (s), 1076 (s), 1018 (m), 906 (w), 869 (m), 797 (w), 781 (w), 702 (w), 640 (m). **^{1}H NMR (400 MHz, $CDCl_3$)**: δ = 8.80 (d, *J* = 2.2 Hz, 1H), 8.30 (d, *J* = 8.2 Hz, 1H), 8.21 (d, *J* = 8.8 Hz, 1H), 8.08 (dd, *J* = 8.4, 2.2 Hz, 1H), 4.06 – 3.96 (m, 1H), 3.89 – 3.75 (m, 2H), 2.86 (s, 1H), 1.87 – 1.74 (m, 1H), 1.64 – 1.50 (m, 1H), 1.30 – 1.15 (m, 1H), 1.00 (d, *J* = 6.8 Hz, 3H), 0.93 (t, *J* = 7.4 Hz, 3H). **^{13}C NMR (101 MHz, $CDCl_3$)**: δ = 163.7, 152.7, 145.3 (d, *J* = 4.0 Hz), 135.0 (q, *J* = 3.5 Hz), 129.0 (q, *J* = 33.3 Hz), 123.25 (q, *J* = 272.5 Hz), 122.3, 63.8, 56.7, 35.9, 25.6, 15.8, 11.5. **^{19}F NMR (282 MHz, $CDCl_3$)** δ = −62.45 (s, 3F). **HRMS (ESI)**: Calculated for $C_{13}H_{18}F_3N_2O_2^+$ [M+H]$^+$: 291.1315, found: 291.1314.

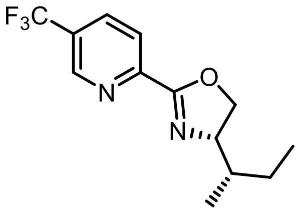

(*S*)-4-((*S*)-*sec*-Butyl)-2-(5-(trifluoromethyl)pyridin-2-yl)-4,5-dihydrooxazole [L1]

In an oven dried Schlenk tube the amide **S30** (431 mg, 1.49 mmol, 1.00 eq.) was suspended in dry CH_2Cl_2 (15 mL). The mixture was cooled to 0 °C and dry NEt_3 (0.52 mL, 3.7 mmol, 2.50 eq.) was added. Then, mesyl chloride (0.14 mL, 1.9 mmol, 1.25 eq.) was added dropwise. After complete addition the reaction mixture was stirred for 15 min at 0 °C before it was gradually warmed to rt and stirred for additional 16 h. Water was added and the phases were separated. The aq. layer was extracted with CH_2Cl_2 (4 × 10 mL). The combined org. phases were dried over $MgSO_4$, filtered and concentrated *in vacuo.* The residue was taken up in MeOH (10 mL). Sodium hydroxide (178 mg, 4.46 mmol, 3.00 eq.) was added and the mixture was heated to reflux for 3 h. After cooling to rt the mixture was concentrated and water was added. It was extracted with CH_2Cl_2 (3 × 10 mL). The combined org. phases dried over Na_2SO_4, filtered and

concentrated *in vacuo*. The product was obtained by flash column chromatography with the conditions given in the corresponding entry. The desired product was obtained *via* automated FC (CyH:EtOAc; 80:20→60:40) as colorless solid (831 mg, 3.05 mmol, 62%).

M.P.: 94–95 °C. **IR (neat)**: $\tilde{\nu}$ = 3469 (br), 3042 (w), 2968 (w), 2927 (w), 2882 (w), 2325 (w), 2245 (w), 1746 (w), 1637 (m), 1604 (w), 1577 (w), 1494 (w), 1462 (w), 1402 (m), 1332 (s), 1282 (w), 1248 (w), 1165 (s), 1122 (s), 1097 (s), 1077 (m), 1055 (w), 1015 (m), 962 (m), 943 (w), 909 (m), 872 (m), 730 (m), 711 (m), 677 (w). **^{1}H NMR (400 MHz, CDCl$_3$)**: δ = 8.93 (dt, *J* = 2.4, 0.9 Hz, 1H), 8.17 (dt, *J* = 8.2, 0.8 Hz, 1H), 8.00 (ddd, *J* = 8.2, 2.3, 0.8 Hz, 1H), 4.56 – 4.47 (m, 1H), 4.37 – 4.27 (m, 1H), 4.27 – 4.19 (m, 1H), 1.80 – 1.69 (m, 1H), 1.69 – 1.57 (m, 1H), 1.31 – 1.18 (m, 1H), 0.94 (t, *J* = 7.4 Hz, 3H), 0.87 (d, *J* = 6.8 Hz, 3H). **^{13}C NMR (101 MHz, CDCl$_3$)**: δ = 161.6, 150.1 (q, *J* = 1.6 Hz), 146.7 (q, *J* = 4.0 Hz), 134.0 (q, *J* = 3.5 Hz), 128.1 (q, *J* = 33.3 Hz), 123.7, 123.3 (q, *J* = 272.7 Hz), 71.8, 70.7, 39.1, 26.2, 14.5, 11.6. **^{19}F NMR (282 MHz, CDCl$_3$)** δ = −62.50 (s, 3F). **HRMS (ESI)**: Calculated for $C_{13}H_{16}F_3N_2O^+$ [M+H]$^+$: 273.1209, found: 273.1211. **Optical Rotation**: $[\alpha]_D^{25}$ = −55.7 (*c* = 1.00, CHCl$_3$).

3 Optimization of reaction protocols

3.1 Optimization of the racemic reaction conditions

Table 1: Initial optimization using Pd(NO$_2$)Cl(MeCN)$_2$ and MCB **1a** as model substrate.

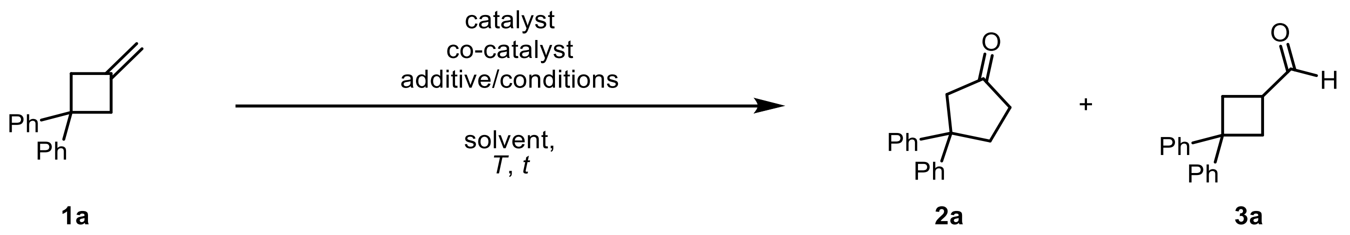

Entry	Catalyst	Cat-load. [mol%]	Co-cat.	eq.	Additive/ Conditions	eq.	Solvent	Conc. [mol/l]	*T* [°C]	*t* [h]	Yield 2a	Yield 3a
**1**	Pd(NO$_2$)Cl(MeCN)$_2$	5	CuCl$_2$	0.20	O$_2$-atm.	-	*t*BuOH	0.2	30	18	**10**	-
**2**	Pd(NO$_2$)Cl(MeCN)$_2$	10	CuCl$_2$	0.40	H$_2$O$_2$	5.00	*t*BuOH	0.1	30	17	**81**	11
**3**	Pd(NO$_2$)Cl(MeCN)$_2$	10	CuCl$_2$	0.40	H$_2$O$_2$	5.00	*t*BuOH	0.1	30	5	**74**	10
**4**	Pd(NO$_2$)Cl(MeCN)$_2$	10	CuCl$_2$	0.40	H$_2$O$_2$	5.00	*t*BuOH	0.1	30	1	**16**	2
**5**	-	-	CuCl$_2$	0.40	H$_2$O$_2$	5.00	*t*BuOH	0.1	30	20	**0**	-
**6**	Pd(NO$_2$)Cl(MeCN)$_2$	10	CuCl$_2$	0.40	-	-	*t*BuOH	0.1	30	17	**78**	14
**7**	Pd(NO$_2$)Cl(MeCN)$_2$	10	CuCl$_2$	0.40	O$_2$-atm.	-	*t*BuOH	0.1	30	20	**70**	14
**8**	Pd(NO$_2$)Cl(MeCN)$_2$	10	CuCl$_2$	0.40	Ar-atm.	-	*t*BuOH	0.1	30	20	**77**	13
**9**[a]	Pd(NO$_2$)Cl(MeCN)$_2$	10	CuCl$_2$	0.40	Ar-atm.	-	*t*BuOH	0.1	30	20	**66**	13
**10**	Pd(NO$_2$)Cl(MeCN)$_2$	10	CuCl$_2$	0.40	mCPBA	5.00	*t*BuOH	0.1	30	19	**0**	-
**11**	Pd(NO$_2$)Cl(MeCN)$_2$	10	CuCl$_2$	0.40	TBHP	5.00	*t*BuOH	0.1	30	19	**11**	-
**12**	Pd(NO$_2$)Cl(MeCN)$_2$	10	CuCl$_2$	0.40	Cum.OOH	5.00	*t*BuOH	0.1	30	20	**12**	2
**13**	Pd(NO$_2$)Cl(MeCN)$_2$	10	CuCl$_2$	0.40	NMO	1.00	*t*BuOH	0.1	30	17	**3**	-
**14**	Pd(NO$_2$)Cl(MeCN)$_2$	10	CuCl$_2$	0.40	H$_2$O	28.3	*t*BuOH	0.1	30	17	**76**	10

**15**	Pd(NO$_2$)Cl(MeCN)$_2$	10	CuCl$_2$	0.40	H$_2$O	5.00	dry *t*BuOH	0.1	30	16	**82**	11
**16**	Pd(NO$_2$)Cl(MeCN)$_2$	10	CuCl$_2$	0.40	H$_2$O	30.0	dry *t*BuOH	0.1	30	16	**89**	11
**17**	Pd(NO$_2$)Cl(MeCN)$_2$	10	CuCl$_2$	0.40	H$_2$O	50.0	dry *t*BuOH	0.1	30	16	**41**	5
**18**	Pd(NO$_2$)Cl(MeCN)$_2$	10	CuCl$_2$	0.40	H$_2$O	300.0	dry *t*BuOH	0.1	30	16	**26**	2
**19**	Pd(NO$_2$)Cl(MeCN)$_2$	10	CuCl$_2$	0.10	H$_2$O	30.0	dry *t*BuOH	0.1	30	17	**75**	11
**20**	Pd(NO$_2$)Cl(MeCN)$_2$	5	CuCl$_2$	0.20	H$_2$O	30.0	dry *t*BuOH	0.1	30	21	**69**	10
**21**	Pd(NO$_2$)Cl(MeCN)$_2$	10	-		-	-	*t*BuOH	0.1	30	20	**6**	1
**22**	Pd(NO$_2$)Cl(MeCN)$_2$	10	CuCl$_2$	1.00	H$_2$O	30.0	dry *t*BuOH	0.1	30	17	**85**	8
**23**	Pd(NO$_2$)Cl(MeCN)$_2$	10	CuCl	0.40	H$_2$O$_2$	5.00	*t*BuOH	0.1	30	17	**8**	1
**24**	Pd(NO$_2$)Cl(MeCN)$_2$	10	Cu(OAc)$_2$	0.40	-	-	*t*BuOH	0.1	30	20	**5**	1
**25**	Pd(NO$_2$)Cl(MeCN)$_2$	10	CuCl$_2$	0.40	-	-	*t*AmylOH	0.1	30	16	**49**	5
**26**	Pd(NO$_2$)Cl(MeCN)$_2$	10	CuCl$_2$	0.40	-	-	dry *t*BuOH	0.1	30	16	**66**	19
**27**	Pd(NO$_2$)Cl(MeCN)$_2$	10	CuCl$_2$	0.40	H$_2$O	30.0	abs. EtOH:*t*BuOH (1:1)	0.1	30	21	**77**	0
**28**	Pd(NO$_2$)Cl(MeCN)$_2$	10	CuCl$_2$	0.40	H$_2$O	30.0	iPrOH	0.1	30	17	**61**	7
**29**	Pd(NO$_2$)Cl(MeCN)$_2$	10	CuCl$_2$	0.40	H$_2$O	30.0	acetone	0.1	30	17	**52**	4
**30**	Pd(NO$_2$)Cl(MeCN)$_2$	10	CuCl$_2$	0.40	H$_2$O	30.0	CH$_2$Cl$_2$	0.1	30	17	**0**	0
**31**	Pd(NO$_2$)Cl(MeCN)$_2$	10	CuCl$_2$	0.40	H$_2$O	30.0	abs. EtOH	0.1	30	18	**9**	0
**32**	Pd(NO$_2$)Cl(MeCN)$_2$	10	CuCl$_2$	0.40	H$_2$O	30.0	dry EtOH	0.1	30	17	**10**	0
**33**	Pd(NO$_2$)Cl(MeCN)$_2$	10	CuCl$_2$	0.40	H$_2$O	30.0	EtOH : CH$_2$Cl$_2$ (1:1)	0.1	30	18	**31**	1
**34**	Pd(NO$_2$)Cl(MeCN)$_2$	10	CuCl$_2$	0.40	H$_2$O	30.0	EtOH : CH$_2$Cl$_2$ (1:9)	0.1	30	18	**1**	0
**35**	Pd(NO$_2$)Cl(MeCN)$_2$	10	CuCl$_2$	0.40	H$_2$O	30.0	EtOH : acetone (1:1)	0.1	30	18	**25**	1
**36**	Pd(NO$_2$)Cl(MeCN)$_2$	10	CuCl$_2$	0.40	H$_2$O	30.0	EtOH : acetone (1:9)	0.1	30	18	**51**	3
**37**	Pd(NO$_2$)Cl(MeCN)$_2$	10	CuCl$_2$	0.40	H$_2$O	30.0	*L*-Menthol	0.1	45	18	**26**	1
**38**	Pd(NO$_2$)Cl(MeCN)$_2$	10	CuCl$_2$	0.40	H$_2$O	30.0	*L*-Menthol : *t*BuOH (1:1)	0.1	30	18	**88**	2
**39**	Pd(NO$_2$)Cl(MeCN)$_2$	10	CuCl$_2$	0.40	H$_2$O	30.0	*L*-Menthol : CH$_2$Cl$_2$ (1:1)	0.1	30	18	**2**	0
**40**	Pd(NO$_2$)Cl(MeCN)$_2$	8	CuCl$_2$	0.40	H$_2$O	30.0	*L*-Menthol : acetone (1:1)	0.1	30	18	**55**	0
**41**	Pd(NO$_2$)Cl(MeCN)$_2$	9	CuCl$_2$	0.40	H$_2$O	30.0	*L*-Menthol : acetone (1:3)	0.1	30	18	**59**	3
**42**	Pd(NO$_2$)Cl(MeCN)$_2$	10	CuCl$_2$	0.40	H$_2$O	30.0	*L*-Menthol : acetone (1:9)	0.1	30	18	**61**	3
**43**[b]	Pd(NO$_2$)Cl(MeCN)$_2$	10	CuCl$_2$	0.40	H$_2$O	30.0	*L*-Menthol : acetone (1:9)	0.1	30	20	**90**	4

**44**[b]	$Pd(NO_2)Cl(MeCN)_2$	10	$CuCl_2$	0.40	H_2O	30.0	glycol : dry *t*BuOH (1:1)	0.1	30	18	**44**	0
**45**[b]	$Pd(NO_2)Cl(MeCN)_2$	10	$CuCl_2$	0.40	H_2O	30.0	glycol	0.1	30	18	**52**	0
**46**[b]	$Pd(NO_2)Cl(MeCN)_2$	10	$CuCl_2$	0.40	H_2O	30.0	dry *t*BuOH	0.1	30	18	**78**	2
**47**[c]	$Pd(NO_2)Cl(MeCN)_2$	10	$CuCl_2$	0.40	H_2O	30.0	dry *t*BuOH	0.1	30	17	**0**	0

Reactions were run on 0.10 mmol scale. An aqueous work up was performed. Yields were determined *via* ^{1}H-NMR analysis using mesitylene as internal standard. [a] Reaction mixture was purged with argon. [b] MCB **1j** was used instead of MCB **1a**. [c] *t*BuPyrOx ligand added (0.20 eq.).

Table 2: Catalyst screening using MCB **1a** as model substrate.

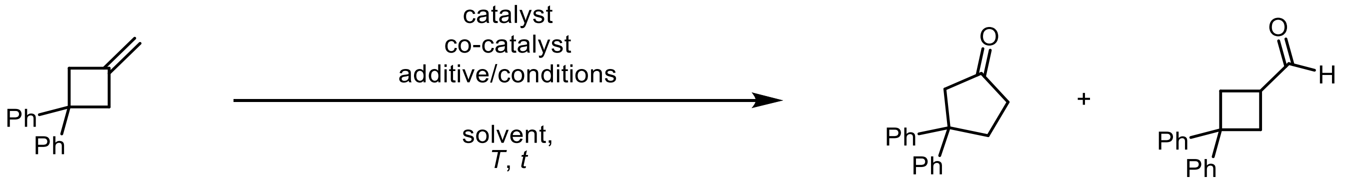

1a **2a** **3a**

Entry	Catalyst	Cat-load. [mol%]	Co-cat.	eq.	Additive/ Conditions	eq.	Solvent	Conc. [mol/l]	*T* [°C]	*t* [h]	Yield 2a	Yield 3a
**1**	$PdCl_2$	10	$CuCl_2$	0.40	H_2O_2	5.00	*t*BuOH	0.1	30	20	**19**	5
**2**	$PdCl_2$	10	$CuCl_2$	0.40	H_2O_2	5.00	*t*BuOH	0.1	60	20	**17**	10
**3**	$PdCl_2$	10	$CuCl_2$	0.40	-	-	*t*BuOH	0.1	30	16	**2**	
**4**	$Pd(OAc)_2$	10	$CuCl_2$	0.40	H_2O_2	5.00	*t*BuOH	0.1	30	20	**10**	3
**5**	$Pd(OAc)_2$	10	$Cu(OAc)_2$	0.40	H_2O_2	5.00	*t*BuOH	0.05	30	20	**4**	-
**6**	$PdCl_2(MeCN)_2$	10	$CuCl_2$	0.40	-	-	*t*BuOH	0.1	30	16	**8**	5
**7**	$Pd(NO_2)Cl(Quinox)$	10	$CuCl_2$	0.40	H_2O	30.0	dry *t*BuOH	0.1	30	17	**0**	0
**8**	$PdCl_2(MeCN)_2$+$AgNO_2$	10	$CuCl_2$	0.40	H_2O	30.0	dry *t*BuOH	0.1	30	17	**89**	11
**9**	$PdCl_2(PPh_3)_2$+$AgNO_2$	10	$CuCl_2$	0.40	H_2O	30.0	dry *t*BuOH	0.1	30	17	**0**	0
**10**	$PdCl_2(dppf)(CH_2Cl_2)$+$AgNO_2$	10	$CuCl_2$	0.40	H_2O	30.0	dry *t*BuOH	0.1	30	17	**0**	0
**11**	$PdCl_2(PPh_3)_2$+$AgNO_2$	10	$CuCl_2$	0.40	H_2O	30.0	dry EtOH	0.1	30	17	**0**	0
**12**	$PdCl_2(dppf)(CH_2Cl_2)$+$AgNO_2$	10	$CuCl_2$	0.40	H_2O	30.0	dry EtOH	0.1	30	17	**0**	0
**13**	$AgNO_2$	10	$CuCl_2$	0.10	H_2O	30.0	dry *t*BuOH	0.1	30	21	**0**	0

**14**	Pd(PhCN)$_2$Cl$_2$	7.5	*t*BuONO	0.20	O_2-atm.	-	dry EtOH + 0.2 H_2O	0.1	30	20	**13**	0
**15**	Pd(PhCN)$_2$Cl$_2$	7.5	*t*BuONO	0.50	O_2-atm.	-	dry EtOH + 0.2 H_2O	0.1	rt	40	**35**	0
**16**	Pd(PhCN)$_2$Cl$_2$	7.5	*t*BuONO	0.20	O_2-atm.	-	dry EtOH + 0.2 H_2O	0.1	rt	40	**14**	0

Reactions were run on 0.10 mmol scale. An aqueous work up was performed. Yields were determined *via* ^{1}H-NMR analysis using mesitylene as internal standard.

Table 3: Continued optimization of the reaction conditions using MCB **1j** as model substrate.

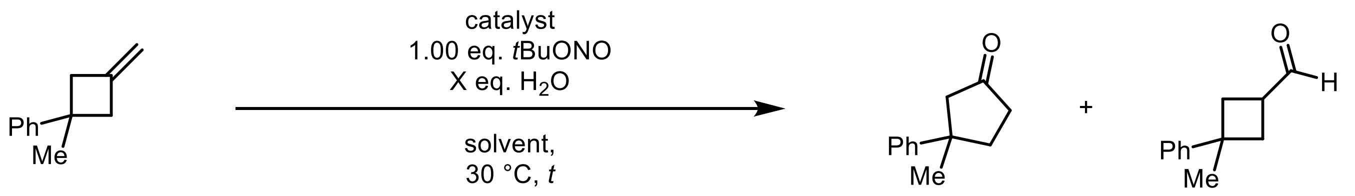

1j **2j** **3j**

Entry	Catalyst	Additive/Conditions	eq.	Solvent	*t* [h]	Yield 2j	Yield 3j
**1**	Pd(PhCN)$_2$Cl$_2$	H_2O	2.00	dry EtOH	40	**85**	0
**2**	Pd(PhCN)$_2$Cl$_2$	Ar-atm.	-	dry EtOH	18	**29**	0
**3**	-	H_2O	2.00	dry EtOH	16	**0**	0
**4**	Pd(CF$_3$PyrOx Ph)Cl$_2$	H_2O	2.00	dry EtOH	18	**83**	0
**5**	Pd(TFA)$_2$	H_2O	2.00	dry EtOH	18	**18**	0
**6**[a]	Pd(TFA)$_2$	H_2O	2.00	dry EtOH	18	**13**	0
**7**	Pd(OAc)$_2$	H_2O	2.00	dry EtOH	18	**0**	0
**8**[a]	Pd(OAc)$_2$	H_2O	2.00	dry EtOH	18	**0**	0
**9**	Pd(PhCN)$_2$Cl$_2$	H_2O	2.00	MeCN	18	**0**	0
**10**	Pd(PhCN)$_2$Cl$_2$	H_2O	2.00	DMF	18	**29**	0
**11**	Pd(PhCN)$_2$Cl$_2$	H_2O	2.00	acetone	18	**54**	0
**12**	Pd(PhCN)$_2$Cl$_2$	H_2O	2.00	glycol	16	**33**	0
**13**	Pd(PhCN)$_2$Cl$_2$	H_2O	2.00	glycol : dry EtOH (1:1)	16	**54**	0
**14**[b]	Pd(PhCN)$_2$Cl$_2$	H_2O	2.00	dry EtOH	16	**88**	0
**15**[c]	Pd(PhCN)$_2$Cl$_2$	H_2O	2.00	dry EtOH	16	**88**	0

**16**	$Pd(PhCN)_2Cl_2$	H_2O	2.00	dry EtOH	4	**81**	0
**17**	$Pd(PhCN)_2Cl_2$	H_2O	2.00	dry EtOH	7	**79**	0
**18**	$Pd(PhCN)_2Cl_2$	H_2O	2.00	dry EtOH	1	**57**	0
**19**	$Pd(PhCN)_2Cl_2$	H_2O	10.0	dry EtOH	18	**92**	0
**20**	$Pd(PhCN)_2Cl_2$	H_2O	30.0	dry EtOH	18	**91**	0
**21**	$Pd(PhCN)_2Cl_2$	H_2O	10.0	dry EtOH	1	**79**	0
**22**	$Pd(PhCN)_2Cl_2$	H_2O	30.0	dry EtOH	1	**87**	0
**23**	$Pd(PhCN)_2Cl_2$	H_2O	50.0	dry EtOH	1	**96**	0
**24**	$Pd(PhCN)_2Cl_2$	H_2O	30.0	dry EtOH	3	**94**	0

Reactions were run on 0.10 mmol scale, using 10 mol% catalyst in 1 mL solvent (0.1 M). An aqueous work up was performed. Yields were determined *via* ^{1}H-NMR analysis using mesitylene as internal standard. [a] 20 mol% PhCN were added to the reaction mixture. [b] Reaction run on 0.30 mmol scale. [c] Direct concentration of the reaction mixture without aqueous work up.

Table 4: Continued optimization of the reaction conditions using MCB **1j** as model substrate omitting the aqueous work up.

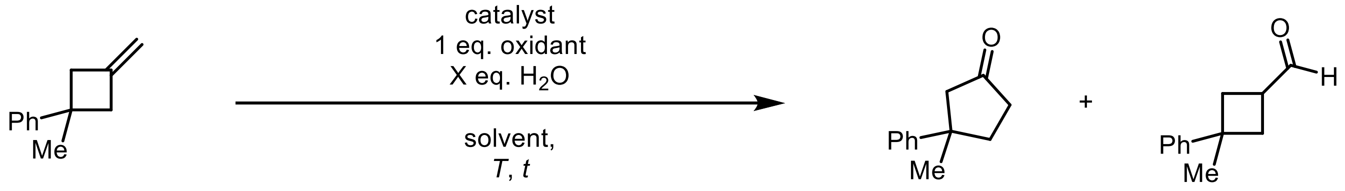

1j **2j** **3j**

Entry	Catalyst	Cat-load. [mol%]	Oxidant	Additive/Conditions	eq.	Solvent	*T* [°C]	*t* [h]	Yield 2a	Yield 3a
**1**	$Pd(PhCN)_2Cl_2$	10	*t*BuONO	H_2O	30.0	dry EtOH	30	3	**99**	0
**2**	$Pd(PhCN)_2Cl_2$	10	*t*BuONO	H_2O	50.0	dry EtOH	30	1	**91**	0
**3**	$Pd(PhCN)_2Cl_2$	10	*t*BuONO	H_2O	50.0	dry EtOH	30	0.5	**37**	0
**4**	$PdCl_2(PPh_3)_2$	5	*t*BuONO	H_2O	30.0	dry EtOH	30	3	**0**	0
**5**	$PdCl_2(MeCN)_2$	10	*t*BuONO	H_2O	30.0	dry EtOH	30	3	**100**	0
**6**	$PdCl_2(MeCN)_2$	10	*t*BuONO	H_2O	30.0	abs. EtOH	30	3	**100**	0
**7**	$PdCl_2(MeCN)_2$	10	benzoquinone	H_2O	30.0	dry EtOH	30	3	**0**	0

**8**	$PdCl_2(MeCN)_2$	5	benzoquinone	-	-	DMF : H_2O (2:1)	30	3	**0**	0
**9**	$PdCl_2(MeCN)_2$	5	benzoquinone	H_2O	30.0	*t*AmylOH	30	3	**0**	0
**10**	**$PdCl_2(MeCN)_2$**	**5**	*t*BuONO	**H_2O**	**30.0**	**dry EtOH**	**30**	**3**	**100**	0
**11**	$PdCl_2(MeCN)_2$	2.5	*t*BuONO	H_2O	30.0	dry EtOH	30	3	**74**	0
**12[a]**	$PdCl_2(MeCN)_2$	5	*t*BuONO	H_2O	30.0	dry EtOH	30	3	**74**	0
**13[a]**	$PdCl_2(MeCN)_2$	5	*t*BuONO	-	-	dry EtOH	30	3	**0**	0
**14[a]**	$PdCl_2(MeCN)_2$	5	*t*BuONO	-	-	dry CH_2Cl_2	30	3	**22**	0
**15**	$PdCl_2(MeCN)_2$	5	*t*BuONO	H_2O	30.0	dry EtOH	rt	3	**88**	0
**16**	$PdCl_2(MeCN)_2$	5	*t*BuONO	H_2O	30.0	dry EtOH	0	3	**28**	0
**17[b]**	$PdCl_2(MeCN)_2$	5	*t*BuONO	H_2O	30.0	dry EtOH	30	3	**0**	0
**18[b]**	$PdCl_2(MeCN)_2$	5	*t*BuONO	H_2O	30.0	$CHCl_3$	30	3	**0**	0
**19[b]**	$PdCl_2(MeCN)_2$	5	*t*BuONO	-	-	$CHCl_3$	30	3	**0**	0

Reactions were run on 0.10 mmol scale in 1 mL solvent (0.1 M). No aqueous work up was performed; the reaction mixture was directly concentrated and submitted for ^{1}H-NMR analysis for yield determination. Yields were determined *via* ^{1}H-NMR analysis using mesitylene as internal standard. [a] Addition of 2,2-dimethyl-1,3-propanediol (30.0 eq.). [b] Addition of (1*S*,2*R*)-1-amino-2-indanol (1.00 eq.).

3.2 Reoptimization of standard reaction conditions for the asymmetric ring expansion

The standard reaction conditions optimized for the racemic oxidative ring-expansion had to be reoptimized for an asymmetric approach as it turned out that the catalyst is not active when complexed with a bidentate ligand. The optimization results are summarized in Table S5.

Table S5: Optimization of reaction conditions for the asymmetric oxidative ring-expansion of methylenecyclobutanes.

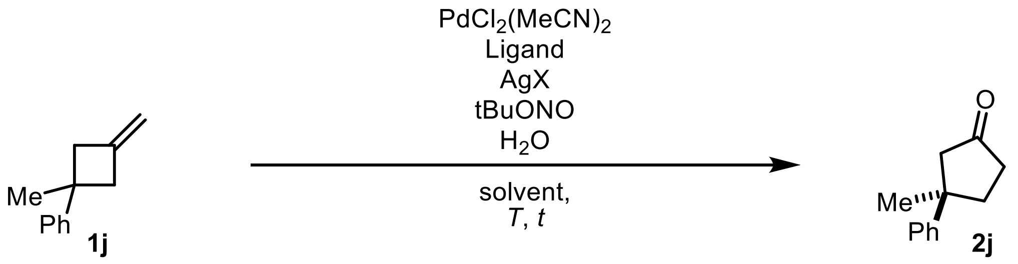

Entry	Ligand	AgX	AgX [mol%]	*T* [°C]	Solvent	Changes to GP	NMR Yield[d]	Yield	*er*
**1[a]**	L1	-	-	30	EtOH dry	No $AgClO_4$	-	-	-
**2[a]**	L1	-	-	78	EtOH dry	No $AgClO_4$	-	-	-
**3[a,c]**	-	$AgClO_4$	15	78	EtOH dry	No Cat & Ligand	-	-	-
**4[a]**	L1	$AgClO_4$	15	30	EtOH dry	30 °C	-	-	-
**5[a]**	L1	$AgClO_4$	10	78	EtOH dry		66%	57%	62:38
**6[a]**	L1	$AgClO_4$	20	78	EtOH dry		74%	63%	63:37
**7[b]**	L1	$AgSbF_6$	15	78	EtOH dry		76%	69%	63:37
**8[b]**	L1	$AgClO_4$	11	78	EtOH dry		83%	80%	63:37
**9[b]**	L1	$AgClO_4$	11	60	$CHCl_3$		-	-	-
**10[b]**	L1	$AgClO_4$	11	60	Acetone		-	-	-
**11[b]**	L1	$AgClO_4$	11	60	MTBE		-	-	-
**12[b]**	L1	$AgClO_4$	11	60	HFIP		-	-	-
**13[b]**	L1	$AgClO_4$	11	78	*t*BuOH		40%	37%	60:40

Reactions were run on 0.20 mmol scale in 2 mL solvent (0.1 M) using 10 mol% catalyst, 11 mol% ligand, 1.00 eq. *t*BuONO and 30.0 eq. water. [a] Reaction run for 4 h. [b] Reaction run for 18 h. [c] Reaction performed without addition of catalyst. [d] Determined *via* ^{1}H-NMR analysis using mesitylene as internal standard.

3.3 Ligand optimization

The complete details of the ligand screening are shown in Table S6 with the corresponding ligand structures given in Figure S1. Following ligands were either

commercially available or synthesized *via* literature procedures: **L2**,[23] **L3**,[24] **L4**,[25] **L5**,[26] **L6**,[27] **L7**,[28] **L8**,[29] **L9** (CAS [409312-96-5]), **L10**,[30] **L11** (CAS [218290-24-5]), **L12**,[31] **L13** (CAS [492-08-0]), **L14** (CAS [657408-07-6]).

Table S6: Ligand screening conditions for the asymmetric oxidative ring-expansion of methylenecyclobutanes.

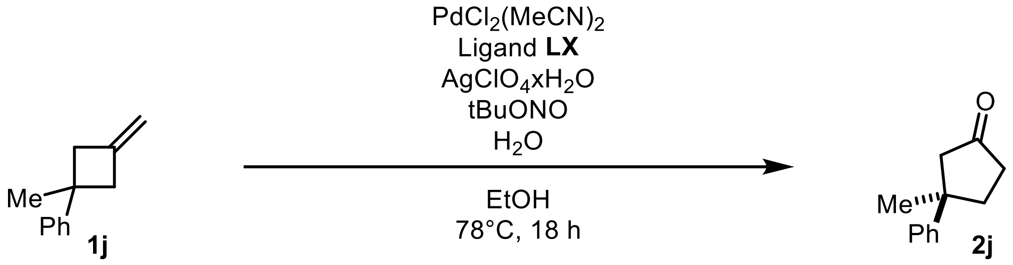

Entry	Ligand	NMR Yield[a]	Yield	*er*[b]
**1**	L1	83%	80%	63:37
**2**	L2	73%	n.d.	63:37
**3**	L3	86%	75%	60:40
**4**	L4	80%	80%	62:87
**5**	L5	60%	48%	62:38
**6**	L6	n.d.	77%	61:39
**7**	L7	26%	n.d.	56:44
**8**	L8	30%	29%	54:46
**9**	L9	n.d.	37%	50:50
**10**	L10	40%	n.d.	53:47
**11**	L11	n.d.	52%	50:50
**12**	L12	55%	55%	52:48
**13**	L13	47%	37%	50:50
**14**	L14	-	-	-

Reactions were run on 0.20 mmol scale in 2 mL solvent (0.1 M) using 10 mol% catalyst, 11 mol% ligand, 11 mol% $AgClO_4xH_2O$, 1.00 eq. *t*BuONO and 30.0 eq. water. [a] Determined *via* ^{1}H-NMR analysis using mesitylene as internal standard. [b] The enantiomeric ratio was determined *via* HPLC using a chiral stationary phase (detailed conditions see corresponding entry in chapter 5.3).

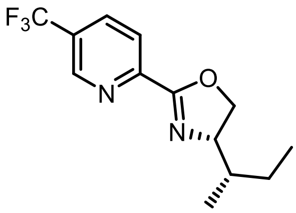

L1

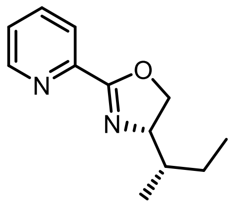

L2

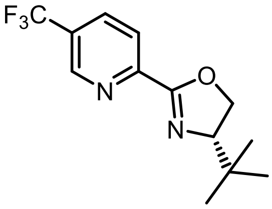

L3

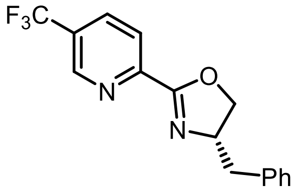

L4

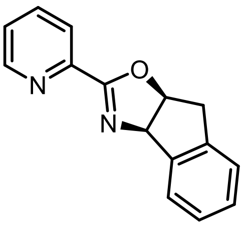

L5

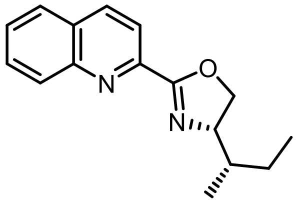

L6

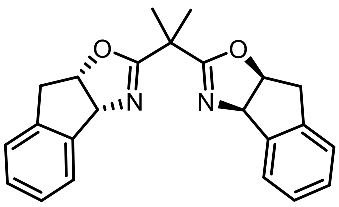

L7

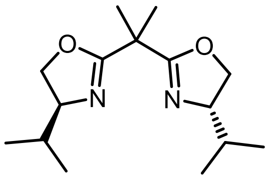

L8

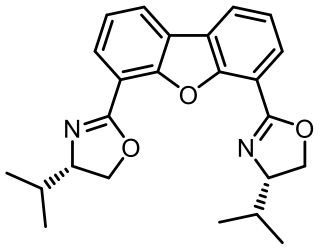

L9

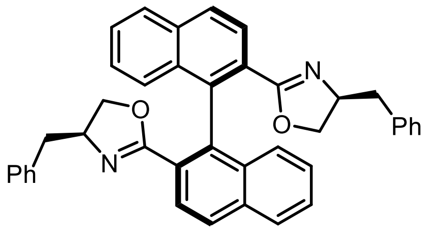

L10

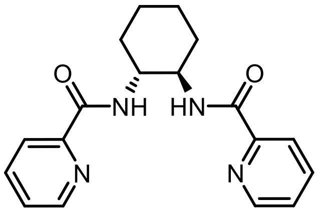

L11

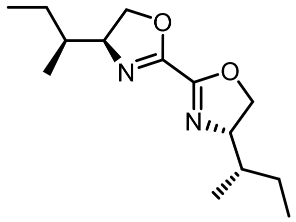

L12

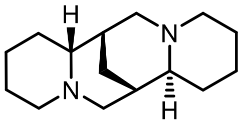

L13

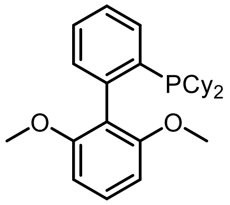

L14

Figure S1: Structure of ligands tested in this study.

4 Isotope labeling experiments

4.1 ^{18}O-Labeling experiments

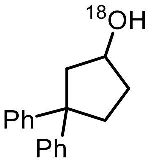

3,3-Diphenylcyclopentan-1-ol-^{18}O [4a]

A tube was charged with $PdCl_2(MeCN)_2$ (1.3 mg, 0.005 mmol, 0.05 eq.) and EtOH (1.00 ml, 0.1 M). $H_2{}^{18}O$ (60.1 mg, 54 µL, 3.00 mmol, 30.00 eq.) was added before the MCB **1a** (22.0 mg, 0.10 mmol, 1.00 eq.) and *t*BuONO (90% purity, 11.5 mg, 13 µL, 0.10 mmol, 1.00 eq.) were added with a micro-syringe. The reaction mixture was stirred for 2 h at 30 °C. $NaBH_4$ (189.2 mg, 5.00 mmol, 50.00 eq.) was added at 0 °C and the mixture was stirred for 16 h at rt. Water (5 mL) and CH_2Cl_2 (5 mL) were added, the layers were separated and the aq. layer was extracted with CH_2Cl_2 (3 x 10 mL). The combined org. layers were dried over $MgSO_4$, filtered and concentrated under reduced pressure. The desired product was obtained *via* FC (pentane:Et_2O; 8:1→2:1) as colorless oil (21.4 mg, 0.089 mmol, 89%).

^{1}H NMR (400 MHz, $CDCl_3$): δ = 7.39 – 7.22 (m, 8H), 7.23 – 7.10 (m, 2H), 4.46 (tdd, *J* = 7.4, 5.5, 3.8 Hz, 1H), 2.88 (ddd, *J* = 13.5, 7.0, 1.5 Hz, 1H), 2.54 (dt, *J* = 12.9, 8.5 Hz, 1H), 2.46 – 2.27 (m, 2H), 2.15 (dq, *J* = 13.8, 8.0 Hz, 1H), 1.72 (dddd, *J* = 13.7, 8.7, 5.1, 3.8 Hz, 1H). **^{13}C NMR (101 MHz, $CDCl_3$)**: δ = 149.0, 148.2, 128.4, 128.3, 127.0, 126.9, 125.9, 125.9, 72.9, 72.8, 55.2, 48.6, 37.1, 34.4. **HRMS (EI)**: Calculated for $C_{17}H_{18}{}^{18}O^+$ [M]$^+$: 240.1395, found: 240.1395; Oxygen-18: 1-fold: 78.21% (Figure 2). Spectroscopic data was in in agreement with that previously reported.[32]

WWU MÜNSTER

Organisch-Chemisches Institut
Abteilung Massenspektrometrie
Corrensstraße 40
48149 Münster

0251-83-33299

LabelChecker Results

Formula:	C17 H18 O
Mass (monoisotopic):	238.14
Difference Value:	0.000776
Error Sum:	0.028
Error (%):	1.240

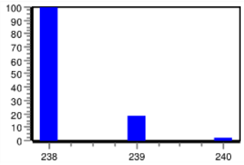

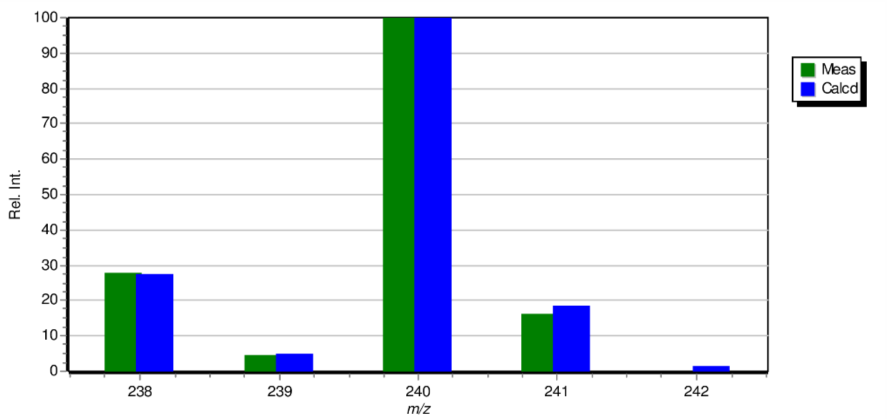

Oxygen-18: 0-fold (%): 27.86 21.79
Oxygen-18: 1-fold (%): 100.00 78.21
Label Atom Sum: 0.78 (78.21%)

Isotope List used for fitting data:

m/z	intensity
238.14	24333
239.13	4179
240.14	87783
241.14	14315

Citation of UMC software in scientific publications: The computer software UMC (Universal Mass Calculator) was used to (e.g. simulate isotope patterns, evaluate the degree of deuteration/labelling, evaluate elemental compositions). UMC Version 3.12.0.89, Dr. Matthias C. Letzel, WWU Münster, Org.-Chem. Institut, Germany. https://www.uni-muenster.de/Chemie.oc/ms/downloads.html

Figure 2: LabelChecker result for the ^{18}O-incorporation experiment.

4.2 ^{13}C-Labeling experiments

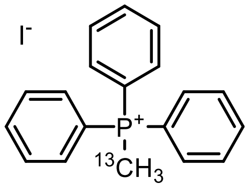

Methyl-^{13}C-triphenylphosphonium iodide [S31]

Triphenylphosphine (1.84 g, 7.00 mmol, 1.00 eq.) was suspended in dry PhMe (15 mL) and Iodo(^{13}C)methane (1.00 g, 437 µL, 7.00 mmol, 1.00 eq.) was added. Colorless precipitation occurred quickly after addition of the iodomethane. The mixture was stirred for 17 h at rt. The solids were collected, washed with hexane and dried under vacuum, affording the product as colorless solid (2.62 g, 6.47 mmol, 92%).

^{1}H NMR (400 MHz, $CDCl_3$): *δ* = 7.78 (ddt, *J*=8.8, 6.4, 1.7, 3H), 7.75 – 7.71 (m, 3H), 7.71 – 7.63 (m, 9H), 3.40 – 2.87 (m, 3H). **^{13}C NMR (101 MHz, $CDCl_3$)**: *δ* = 135.3 (d, *J* = 3.2 Hz, 3C), 133.4 (d, *J* = 10.8 Hz, 6C), 130.6 (d, *J* = 13.0 Hz, 6C), 119.0 (d, *J* = 89.2 Hz, 3C), 11.7 (d, *J* = 57.1 Hz). Spectroscopic data was in agreement with that previously reported.[33]

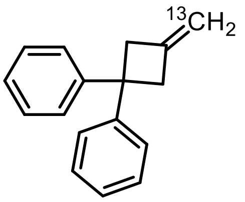

1-(^{13}C)Methylen-3,3-diphenylcyclobutan [^{13}C-1a]

Following modified[2] general procedure **A** using cyclobutanone **S17** 3,3-diphenylcyclobutan-1-one (121 mg, 544 µmol, 1.00 eq.), methyl-^{13}C-triphenylphosphonium iodide (298 mg, 735 µmol, 1.35 eq.) and *n*-BuLi (2.5 M in hexanes, 283 µL, 708 µmol, 2.50M, 1.30 eq.) the desired product was obtained *via* FC (pentane) as colorless oil (78 mg, 0.49 mmol, 62%).

IR (neat): $\tilde{\nu}$ = 3060 (w), 2917 (w), 1945 (w), 1876 (w), 1804 (w), 1743 (w), 1656 (w), 1595 (w), 1493 (m), 1446 (w), 1411 (w), 1313 (w), 1023 (w), 866 (m), 757 (m), 699 (s), 663 (m), 652 (m), 633 (m), 623 (m), 609 (m), 589 (s). **^{1}H NMR (400 MHz, $CDCl_3$)**: *δ* = 7.33–7.26 (m, 8H), 7.22–7.13 (m, 2H), 5.08 (p, *J* = 2.4 Hz, 1H), 4.69 (p, *J* = 2.4 Hz,

[2] The triphenylphosphine oxide was removed via filtration through a short silica plug with CH_2Cl_2.

1H), 3.45 (dt, *J* = 3.4, 2.4 Hz, 4H). **^{13}C NMR (101 MHz, CDCl$_3$)**: δ = 149.2, 144.4, 143.6, 128.4, 126.6, 125.9, 106.8, 46.2. **HRMS (APCI, CH$_3$COONH$_4$)**: Calculated for $C_{16}{}^{13}CH_{17}^+$ [M+H]$^+$: 222.1358, found: 222.1352.

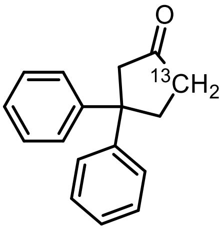

3,3-Diphenylcyclopentan-1-one-5-^{13}C [^{13}C-2a]

Following general procedure **E** using MCB **^{13}C-1a** (22.1 mg, 0.10 mmol, 1.00 eq.), PdCl$_2$(MeCN)$_2$ (1.3 mg, 5.0 µmol, 0.05 eq.), *t*BuONO (90% purity, 13.3 µL, 0.10 mmol, 1.00 eq.) and H_2O (54 µL, 3.00 mmol, 30.0 eq.) the desired product was obtained *via* FC (pentane:EtOAc; 95:5) as pale-yellow resin (22 mg, 93 µmol, 93%).

IR (neat): $\tilde{\nu}$ = 3058 (w), 2919 (w), 2363 (m), 2341 (m), 1596 (w), 1494 (m), 1446 (w), 1401 (w), 1252 (w), 1146 (m), 1034 (w), 771 (m), 755 (m), 697 (s), 653 (m), 623 (m), 596 (s). **^{1}H NMR (400 MHz, CDCl$_3$)**: δ = 7.40–7.29 (m, 8H), 7.29–7.22 (m, 2H), 3.07 (s, 2H), 2.79 (td, *J* = 7.5, 3.3 Hz, 2H), 2.52 (t, *J* = 7.6 Hz, 1H), 2.19 (t, *J* = 7.5 Hz, 1H). **^{13}C NMR (101 MHz, CDCl$_3$)**: δ = 217.5 (d, *J* = 36.7 Hz, 5), 146.7 (2C, 7, 13), 128.7 (4C, 8, 12, 14, 18), 126.8 (4C, 9, 11, 15, 17), 126.6 (2C, 10, 16), 52.0 (d, *J* = 14.7 Hz), 51.2 (d, *J* = 147.8 Hz), 36.7 (4), 35.5 (d, *J* = 35.1 Hz). **HRMS (ESI)**: Calculated for $C_{16}{}^{13}CH_{16}ONa^+$ [M+Na]$^+$: 260.1127, found 260.1127.

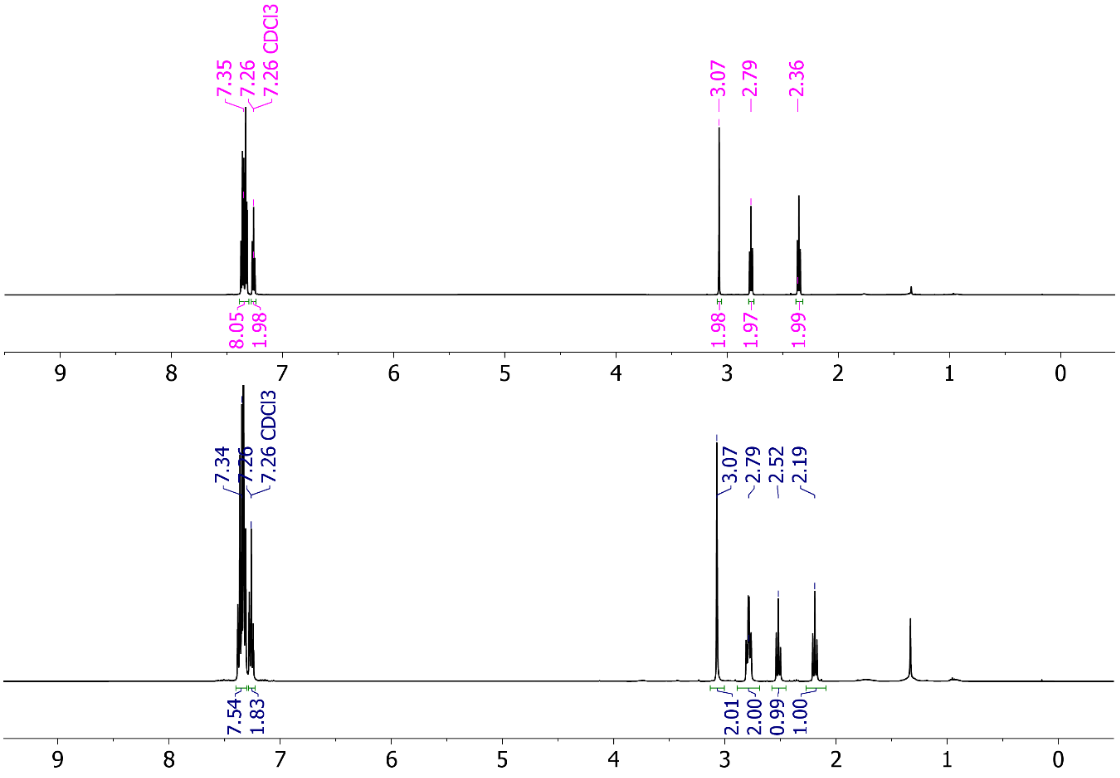

Figure 3: Comparison of the ^{1}H NMR spectra of **[2a]** (top) and **[^{13}C-2a]** (bottom).

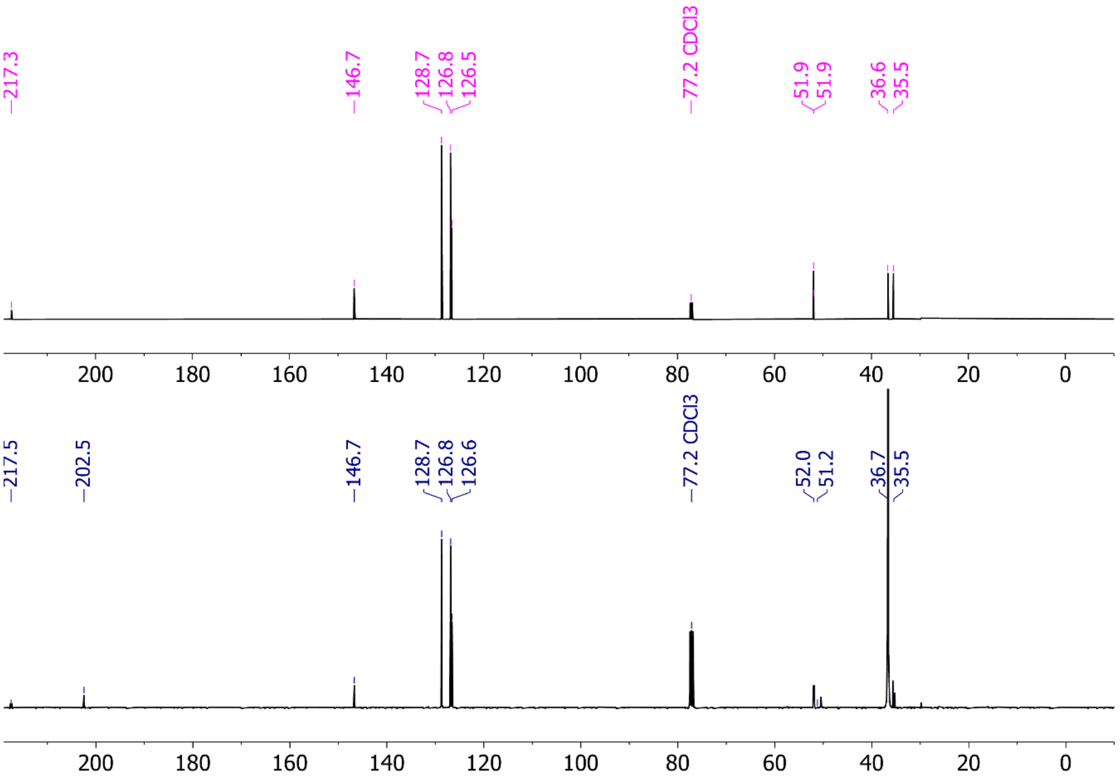

Figure 4: Comparison of the ^{13}C NMR spectra of **[2a]** (top) and **[^{13}C-2a]** (bottom).[3]

[3] The peak at 202.5 ppm in the bottom spectrum is probably caused by residually formed ^{13}C-labeled aldehyde **[^{13}C-3a]**.

5 Synthesis of cyclopentanones

5.1 General procedure E: Oxidative ring expansion

A tube was charged with $PdCl_2(MeCN)_2$ (3.9 mg, 0.015 mmol, 0.05 eq.) and EtOH (3.00 ml, 0.1 M with respect to the MCB). H_2O (162 mg, 162 µL, 30.0 mmol, 30.0 eq.) was added before the methylenecyclobutane and *t*BuONO (90% purity, 34.4 mg, 40 µL, 0.30 mmol, 1.00 eq.) were added with *via* micro-syringe. The reaction mixture was stirred for 3 h at 30 °C. The crude mixture was concentrated in vacuo. The NMR-yield was determined *via* ^{1}H NMR analysis of the crude reaction mixture using mesitylene (14 µL, 0.10 mmol) as internal standard. The product was obtained *via* FC with the conditions given in the corresponding entry.

5.2 Syntheses

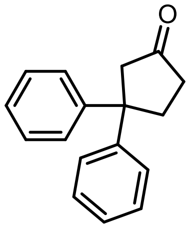

3,3-Diphenylcyclopentan-1-one [2a]

Following procedure **D** using (3-methylenecyclobutane-1,1-diyl)dibenzene (66.1 mg, 0.30 mmol, 1.00 eq.) the desired product was obtained *via* FC (pentane:Et_2O; 10:1) as colorless oil (61.0 mg, 0.258 mmol, 86%).

^{1}H NMR (599 MHz, CDCl$_3$): δ = 7.33 – 7.24 (m, 8H), 7.23 – 7.17 (m, 2H), 3.01 (s, 2H), 2.73 (t, J = 7.5 Hz, 2H), 2.30 (t, J = 7.5 Hz, 2H). **^{13}C NMR (151 MHz, CDCl$_3$)**: δ = 217.3, 146.7, 128.7, 126.8, 126.5, 51.9, 51.9, 36.6, 35.5. Spectroscopic data was in agreement with that previously reported.[34]

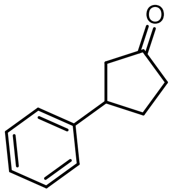

3-Phenylcyclopentan-1-one [2b]

Following procedure **E** using MCB **1b** (43.3 mg, 0.30 mmol, 1.00 eq.) the desired product was obtained *via* FC (pentane:Et_2O; 10:1) as colorless oil (44.2 mg, 0.276 mmol, 92%).

^{1}H NMR (400 MHz, $CDCl_3$): δ = 7.39 – 7.30 (m, 2H), 7.28 – 7.18 (m, 3H), 3.48 – 3.35 (m, 1H), 2.73 – 2.61 (m, 1H), 2.50 – 2.39 (m, 2H), 2.39 – 2.21 (m, 2H), 2.06 – 1.91 (m, 1H). **^{13}C NMR (101 MHz, $CDCl_3$)**: δ = 218.5, 143.2, 128.8, 126.9, 126.9, 45.9, 42.4, 39.0, 31.3. Spectroscopic data was in agreement with that previously reported.[35]

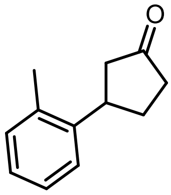

3-(*o*-Tolyl)cyclopentan-1-one [2c]

Following procedure **E** using MCB **1c** (47.5 mg, 0.30 mmol, 1.00 eq.) the desired product was obtained *via* FC (pentane:Et_2O; 10:1) as colorless oil (48.2 mg, 0.277 mmol, 92%).

^{1}H NMR (400 MHz, $CDCl_3$): δ = 7.24 – 7.12 (m, 4H), 3.61 (*app* tdd, $J \approx$ 10.3, 7.5, 6.0 Hz, 1H), 2.68 – 2.59 (m, 1H), 2.55 – 2.43 (m, 1H), 2.43 – 2.21 (m, 6H), 2.08 – 1.93 (m, 1H). **^{13}C NMR (101 MHz, $CDCl_3$)**: δ = 218.8, 141.1, 136.1, 130.8, 126.6, 126.6, 124.9, 45.5, 38.7, 38.5, 30.2, 19.8. Spectroscopic data was in agreement with that previously reported.[36]

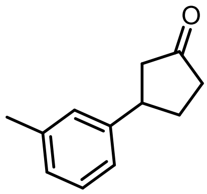

3-(*m*-Tolyl)cyclopentan-1-one [2d]

Following procedure **E** using MCB **1d** (47.5 mg, 0.30 mmol, 1.00 eq.) the desired product was obtained *via* FC (pentane:Et_2O; 10:1) as colorless oil (48.6 mg, 0.279 mmol, 93%).

^{1}H NMR (400 MHz, $CDCl_3$): δ = 7.25 – 7.20 (m, 1H), 7.10 – 7.03 (m, 3H), 3.45 – 3.32 (m, 1H), 2.72 – 2.60 (m, 1H), 2.53 – 2.23 (m, 7H), 2.06 – 1.91 (m, 1H). **^{13}C NMR (101 MHz, $CDCl_3$)**: δ = 218.7, 143.2, 138.4, 128.7, 127.7, 127.6, 123.8, 46.0, 42.3, 39.0, 31.3, 21.6. Spectroscopic data was in agreement with that previously reported.[36]

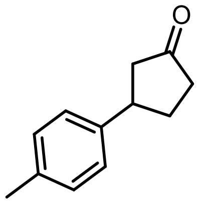

3-(*p*-Tolyl)cyclopentan-1-one [2e]

Following procedure **E** using MCB **1e** (47.5 mg, 0.30 mmol, 1.00 eq.) the desired product was obtained *via* FC (pentane:Et_2O; 10:1) as colorless oil (48.0 mg, 0.275 mmol, 92%).

^{1}H NMR (400 MHz, $CDCl_3$): δ = 7.16 (s, 4H), 3.39 (*app* tdd, $J \approx$ 11.0, 7.5, 6.1 Hz, 1H), 2.73 – 2.60 (m, 1H), 2.52 – 2.38 (m, 2H), 2.37 – 2.24 (m, 5H), 2.05 – 1.89 (m, 1H). **^{13}C NMR (101 MHz, $CDCl_3$)**: δ = 218.6, 140.1, 136.3, 129.4, 126.6, 45.9, 41.9, 38.9, 31.3, 21.0. Spectroscopic data was in agreement with that previously reported.[37]

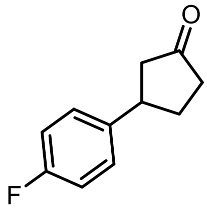

3-(4-Fluorophenyl)cyclopentan-1-one [2f]

Following procedure **E** using MCB **1f** (48.7 mg, 0.30 mmol, 1.00 eq.) the desired product was obtained *via* FC (pentane:Et_2O; 10:1) as colorless oil (48.0 mg, 0.269 mmol, 90%).

^{1}H NMR (400 MHz, CDCl$_3$): *δ* = 7.25 – 7.16 (m, 2H), 7.08 – 6.97 (m, 2H), 3.47 – 3.33 (m, 1H), 2.72 – 2.60 (m, 1H), 2.53 – 2.36 (m, 2H), 2.36 – 2.24 (m, 2H), 2.03 – 1.87 (m, 1H). **^{13}C NMR (101 MHz, CDCl$_3$)**: *δ* = 218.1, 161.6 (d, *J* = 244.8 Hz), 138.7 (d, *J* = 3.2 Hz), 128.2 (d, *J* = 7.9 Hz), 115.5 (d, *J* = 21.2 Hz), 45.9, 41.5, 38.9, 31.3. **^{19}F NMR (377 MHz, CDCl$_3$)**: *δ* = −116.3. Spectroscopic data was in agreement with that previously reported.[36]

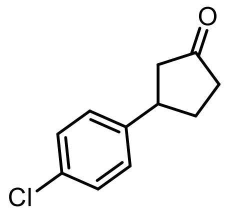

3-(4-Chlorophenyl)cyclopentan-1-one [2g]

Following procedure **E** using MCB **1g** (53.6 mg, 0.30 mmol, 1.00 eq.) the desired product was obtained *via* FC (pentane:Et$_2$O; 10:1) as colorless oil (55.0 mg, 0.283 mmol, 94%).

^{1}H NMR (400 MHz, CDCl$_3$): *δ* = 7.36 – 7.30 (m, 2H), 7.24 – 7.16 (m, 2H), 3.48 – 3.35 (m, 1H), 2.74 – 2.62 (m, 1H), 2.54 – 2.41 (m, 2H), 2.38 – 2.25 (m, 2H), 2.05 – 1.90 (m, 1H). **^{13}C NMR (101 MHz, CDCl$_3$)**: *δ* = 217.8, 141.6, 132.6, 128.9, 128.2, 45.8, 41.8, 38.9, 31.3. Spectroscopic data was in agreement with that previously reported.[36]

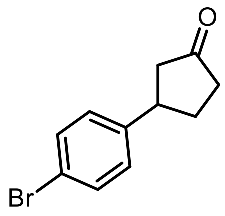

3-(4-Bromophenyl)cyclopentan-1-one [2h]

Following procedure **E** using MCB **1h** (66.9 mg, 0.30 mmol, 1.00 eq.) the desired product was obtained *via* FC (pentane:Et$_2$O; 10:1) as colorless oil (67.0 mg, 0.280 mmol, 93%).

^{1}H NMR (400 MHz, CDCl$_3$): *δ* = 7.48 – 7.41 (m, 2H), 7.17 – 7.08 (m, 2H), 3.37 (*app* tdd, *J* ≈ 11.1, 7.4, 6.0 Hz, 1H), 2.70 – 2.58 (m, 1H), 2.44 (*app* ddddd, *J* ≈ 16.4, 10.2, 8.2, 3.8, 1.9 Hz, 2H), 2.35 – 2.21 (m, 2H), 2.01 – 1.86 (m, 1H). **^{13}C NMR (101 MHz, CDCl$_3$)**: *δ* = 217.8, 142.2, 131.9, 128.6, 120.6, 45.7, 41.8, 38.9, 31.2. Spectroscopic data was in agreement with that previously reported.[35]

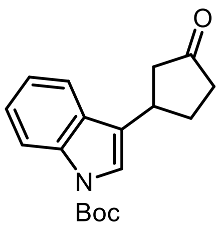

tert-Butyl 3-(3-oxocyclopentyl)-1*H*-indole-1-carboxylate [2i]

Following procedure **E** using MCB **1i** (85.0 mg, 0.30 mmol, 1.00 eq.) the desired product was obtained *via* FC (pentane:Et_2O; 10:1) as colorless oil (81.1 mg, 0.271 mmol, 90%).

IR (neat): $\tilde{\nu}$ = 3053 (w), 2978 (w), 2933 (w), 1725 (s), 1609 (w), 1569 (w), 1476 (m), 1451 (s), 1369 (s), 1339 (m), 1308 (s), 1251 (s), 1220 (m), 1150 (s), 1042 (s), 1059 (m), 1018 (s), 977 (m), 938 (m), 907 (w), 856 (m), 836 (m), 816 (w), 764 (s), 743 (s). **^{1}H NMR (599 MHz, $CDCl_3$)**: *δ* = 8.14 (s, 1H), 7.55 (dt, *J* = 7.8, 1.0 Hz, 1H), 7.38 (br s, 1H), 7.34 (ddd, *J* = 8.4, 7.2, 1.3 Hz, 1H), 7.28 – 7.24 (m, 1H), 3.63 (tddd, *J* = 9.4, 7.5, 6.2, 1.2 Hz, 1H), 2.80 – 2.70 (m, 1H), 2.57 – 2.50 (m, 1H), 2.49 – 2.43 (m, 1H), 2.43 – 2.30 (m, 2H), 2.12 (dtd, *J* = 12.6, 9.5, 8.3 Hz, 1H), 1.67 (s, 9H). **^{13}C NMR (151 MHz, $CDCl_3$)**: *δ* = 218.4, 149.9, 136.0, 129.9, 124.8, 123.1, 122.7, 121.4, 119.3, 115.7, 83.91, 44.8, 38.3, 33.5, 29.4, 28.4. **HRMS (ESI)**: Calculated for $C_{18}H_{21}NO_3Na^+$ $[M+Na]^+$: 322.1414, found: 322.1414.

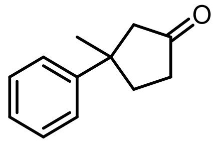

3-Methyl-3-phenylcyclopentan-1-one [2j]

Following procedure **E** using MCB **1j** (47.5 mg, 0.30 mmol, 1.00 eq.) the desired product was obtained *via* FC (pentane:Et_2O; 10:1) as colorless oil (48.3 mg, 0.277 mmol, 92%).

^{1}H NMR (400 MHz, $CDCl_3$): *δ* = 7.40 – 7.18 (m, 5H), 2.66 (d, *J* = 17.6 Hz, 1H), 2.48 (d, *J* = 17.7 Hz, 1H), 2.49 – 2.23 (m, 4H), 1.39 (s, 3H). **^{13}C NMR (101 MHz, $CDCl_3$)**: *δ* = 218.8, 148.6, 128.7, 126.5, 125.6, 52.4, 44.0, 36.9, 35.9, 29.6. Spectroscopic data was in agreement with that previously reported.[38]

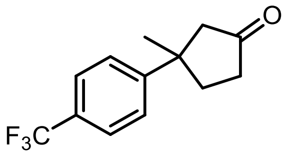

3-Methyl-3-(4-(trifluoromethyl)phenyl)cyclopentan-1-one [2k]

Following procedure **E** using MCB **1k** (67.9 mg, 0.30 mmol, 1.00 eq.) the desired product was obtained *via* FC (pentane:Et_2O; 10:1) as colorless oil (68.6 mg, 0.283 mmol, 94%).

IR (neat): $\tilde{\nu}$ = 2963 (w), 1742 (s), 1613 (m), 1455 (w), 1409 (m), 1380 (w), 1325 (s), 1160 (s), 1109 (s), 1081 (s), 1066 (s), 1015 (s), 981 (w), 955 (w), 900 (w), 839 (s), 814 (w), 778 (w), 746 (w), 726 (w), 712 (w), 667 (m). **^{1}H NMR (400 MHz, $CDCl_3$)**: δ = 7.60 (d, J = 8.2 Hz, 2H), 7.41 (d, J = 8.1 Hz, 2H), 2.64 (d, J = 17.5 Hz, 1H), 2.51 (d, J = 17.4 Hz, 1H), 2.49 – 2.42 (m, 1H), 2.41 – 2.33 (m, 1H), 2.33 – 2.26 (m, 2H), 1.40 (s, 3H). **^{13}C NMR (151 MHz, $CDCl_3$)**: δ = 217.6, 152.7, 128.9 (q, J = 32.7 Hz), 126.0, 125.7 (q, J = 3.8 Hz), 124.2 (q, J = 271.9 Hz), 52.1, 44.08, 36.7, 35.7, 29.4. **^{19}F NMR (564 MHz, $CDCl_3$)**: δ = −62.50. **HRMS (EI)**: Calculated for $C_{13}H_{13}F_3O^+$ $[M]^+$: 242.0913, found: 242.0913.

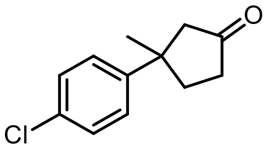

3-(4-Chlorophenyl)-3-methylcyclopentan-1-one [2l]

Following procedure **E** using MCB **1l** (57.8 mg, 0.30 mmol, 1.00 eq.) the desired product was obtained *via* FC (pentane:Et_2O; 10:1) as colorless resin (59.9 mg, 0.287 mmol, 96%).

IR (neat): $\tilde{\nu}$ = 3459 (w), 3051 (w), 3032 (w), 2966 (m), 2931 (w), 2895 (m), 2876 (w), 2898 (w), 1736 (s), 1652 (m), 1495 (m), 1481 (m), 1446 (m), 1403 (m), 1380 (m), 1920 (m), 1286 (m), 1268 (m), 1228 (m), 1201 (m), 1191 (m), 1157 (s), 1139 (m), 1103 (m), 1089 (s), 1011 (s), 998 (m), 984 (m), 961 (m), 891 (m), 858 (m), 824 (s), 761 (m), 731 (m), 720 (m), 694 (m). **^{1}H NMR (400 MHz, $CDCl_3$)**: δ = 7.35 – 7.27 (m, 2H), 7.27 – 7.18 (m, 2H), 2.61 (d, J = 17.6 Hz, 1H), 2.48 – 2.30 (m, 2 H), 2.47 (d, J = 17.6 Hz, 1H), 2.30 – 2.20 (m, 2H), 1.38 (s, 3H). **^{13}C NMR (101 MHz, $CDCl_3$)**: δ = 217.9, 147.0, 132.1, 128.7, 126.9, 52.1, 43.5, 36.6, 35.8, 29.3. **HRMS (EI)**: Calculated for $C_{12}H_{13}ClO^+$ $[M]^+$: 208.0649, found: 208.0649.

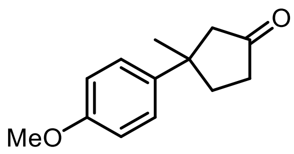

3-Methyl-3-(4-(trifluoromethyl)phenyl)cyclopentan-1-one [2m]

Following procedure **E** using MCB **1m** (56.5 mg, 0.30 mmol, 1.00 eq.) the desired product was obtained *via* FC (pentane:Et_2O; 7.5:1) as colorless oil (51.9 mg, 0.254 mmol, 85%).

IR (neat): $\tilde{\nu}$ = 3036 (w), 3957 (m), 2837 (w),2253 (w), 1738 (s), 1611 (m), 1581 (w), 1513 (s), 1464 (m), 1405 (w), 1378 (w), 1299 (m), 1248 (s), 1182 (s), 1157 (m), 1111 (w), 1080 (w), 1032 (s), 992 (w), 911 (m), 861 (w), 829 (s), 811 (m), 792 (m), 729 (s), 671 (w). **^{1}H NMR (599 MHz, $CDCl_3$)**: δ = 7.24 – 7.18 (m, 2H), 6.90 – 6.85 (m, 2H), 3.79 (s, 3H), 2.62 (d, J = 17.5 Hz, 1H), 2.44 – 2.28 (m, 2H), 2.43 (d, J = 17.3 Hz, 1H), 2.32 – 2.18 (m, 2H), 1.36 (s, 3H). **^{13}C NMR (151 MHz, $CDCl_3$)**: δ = 218.8, 158.0, 140.6, 126.6, 114.0, 55.4, 52.6, 43.3, 36.9, 36.2, 29.6. **HRMS (EI)**: Calculated for $C_{13}H_{16}O_2^+$ $[M]^+$: 204.1145, found: 204.1147.

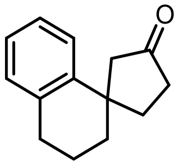

3',4'-Dihydro-2'*H*-spiro[cyclopentane-1,1'-naphthalen]-3-one [2n]

Following procedure **E** using MCB **1n** (55.3 mg, 0.30 mmol, 1.00 eq.) the desired product was obtained *via* FC (pentane:Et_2O; 10:1) as colorless oil (55.1 mg, 0.275 mmol, 92%).

IR (neat): $\tilde{\nu}$ = 3060 (w), 3015 (w), 2928 (m), 2861 (m), 1739 (s), 1491 (m), 1449 (m), 1405 (m), 1294 (w), 1278 (w), 1244 (w), 1200 (w), 1146 (m), 122 (w), 1064 (w), 987 (w), 919 (w), 883 (w), 810 (w), 786 (w), 761 (m), 733 (m). **^{1}H NMR (500 MHz, $CDCl_3$)**: δ = 7.30 (dd, J = 7.9, 1.3 Hz, 1H), 7.22 – 7.17 (m, 1H), 7.14 (td, J = 7.3, 1.4 Hz, 1H), 7.12 – 7.09 (m, 1H), 2.85 (t, J = 5.9 Hz, 2H), 2.69 – 2.62 (m, 1H), 2.54 – 2.29 (m, 4H), 2.15 (dddd, J = 13.0, 8.7, 4.1, 1.8 Hz, 1H), 1.96 – 1.85 (m, 1H), 1.85 – 1.75 (m, 3H). **^{13}C NMR (126 MHz, $CDCl_3$)**: δ = 219.3, 142.3, 137.3, 129.5, 126.4, 126.19, 126.18, 54.7, 42.9, 37.7, 36.8, 35.9, 30.3, 19.8. **HRMS (EI)**: Calculated for $C_{14}H_{16}O^+$ $[M]^+$: 200.1196, found: 200.1196.

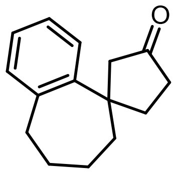

6,7,8,9-Tetrahydrospiro[benzo[7]annulene-5,1'-cyclopentan]-3'-one [2o]

Following procedure **E** using MCB **1o** (59.5 mg, 0.30 mmol, 1.00 eq.) the desired product was obtained *via* FC (pentane:Et_2O; 10:1) as colorless oil (57.9 mg, 0.270 mmol, 90%).

IR (neat): $\tilde{\nu}$ = 3057 (w), 2920 (m), 2854 (w), 1740 (s), 1599 (w), 1488 (m), 1446 (m), 1404 (m), 1355 (w), 1325 (w), 1278 (w), 1249 (m), 1156 (m), 1108 (w), 1090 (w), 1057 (w), 1042 (m), 976 (w), 954 (w), 924 (w), 887 (m), 819 (w), 759 (s), 746 (s). **^{1}H NMR (599 MHz, $CDCl_3$)**: δ = 7.19 – 7.10 (m, 4H), 3.05 – 2.95 (m, 1H), 2.87 (ddd, J = 14.9, 8.5, 2.0 Hz, 1H), 2.77 (d, J = 17.3 Hz, 1H), 2.56 (d, J = 17.4 Hz, 1H), 2.42 – 2.36 (m, 2H), 2.36 – 2.21 (m, 2H), 1.98 – 1.84 (m, 2H), 1.84 – 1.71 (m, 3H), 1.63 – 1.54 (m, 1H). **^{13}C NMR (151 MHz, $CDCl_3$)**: δ = 218.6, 145.4, 142.8, 131.6, 126.9, 126.4, 125.5, 52.5, 48.3, 38.2, 37.4, 36.3, 32.9, 28.0, 27.4. **HRMS (EI)**: Calculated for $C_{15}H_{18}O^+$ [M]$^+$: 214.1352, found: 214.1350.

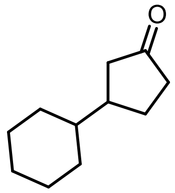

3-Cyclohexylcyclopentan-1-one [2p]

Following procedure **E** using MCB **1p** (45.1 mg, 0.30 mmol, 1.00 eq.) the desired product was obtained *via* FC (pentane:Et_2O; 15:1) as colorless oil (45.0 mg, 0.271 mmol, 90%).

^{1}H NMR (400 MHz, $CDCl_3$): δ = 2.43 – 2.23 (m, 2H), 2.22 – 1.99 (m, 2H), 1.96 – 1.78 (m, 3H), 1.78 – 1.56 (m, 4H), 1.55 – 1.38 (m, 1H), 1.30 – 1.07 (m, 2H), 1.02 – 0.86 (m, 2H). **^{13}C NMR (101 MHz, $CDCl_3$)**: δ = 220.2, 43.7, 43.6, 43.5, 39.2, 32.1, 30.9, 27.7, 26.6, 26.31, 26.28. Spectroscopic data was in agreement with that previously reported.[39]

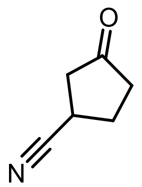

3-Oxocyclopentane-1-carbonitrile [2q]

Following general procedure **E** using MCB **1q** (27.9 mg, 0.30 mmol, 1.00 eq.) the desired product was obtained *via* FC (CH_2Cl_2) as a colorless oil (11.0 mg, 0.101 mmol, 34%). *Note:* The product is fairly volatile.

^{1}H NMR (600 MHz, $CDCl_3$): δ = 3.22 – 3.15 (m, 1H), 2.64 – 2.58 (m, 1H), 2.54 – 2.40 (m, 3H), 2.32 – 2.22 (m, 2H). **^{13}C NMR (151 MHz, $CDCl_3$)**: δ = 212.8, 120.9, 41.5, 36.8, 27.5, 25.7. Spectroscopic data was in agreement with that previously reported.[40]

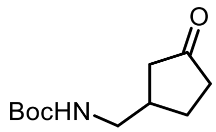

tert-Butyl ((3-oxocyclopentyl)methyl)carbamate [2r]

Following procedure **E** using MCB **1r** (59.2 mg, 0.30 mmol, 1.00 eq.) the desired product was obtained *via* FC (pentane:Et_2O: 1:1) as a yellow oil (56.3 mg, 0.264 mmol, 88%).

^{1}H NMR (400 MHz, $CDCl_3$): δ = 4.65 (br s, 1H), 3.20 (s, 2H), 2.46 – 2.26 (m, 3H), 2.25 – 2.07 (m, 2H), 1.97 – 1.84 (m, 1H), 1.68 – 1.52 (m, 1H), 1.44 (s, 9H). **^{13}C NMR (101 MHz, $CDCl_3$)**: δ = 218.6, 156.1, 44.8, 42.8, 38.2, 37.8, 28.5, 26.9. Spectroscopic data was in agreement with that previously reported.[41]

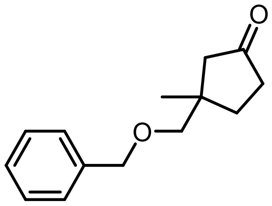

3-((Benzyloxy)methyl)-3-methylcyclopentan-1-one [2s]

Following general procedure **E** using MCB **1s** (51 mg, 0.18 mmol, 1.00 eq.), $PdCl_2(MeCN)_2$ (2.3 mg, 9.0 µmol, 0.05 eq.), *t*BuONO (90% purity, 24 µL, 0.18 mmol, 1.00 eq.) and H_2O (100 µL, 5.42 mmol, 30.0 eq.) the desired product was obtained *via* FC (CH_2Cl_2) as a colorless oil (33 mg, 0.15 mmol, 84%).

IR (neat): $\tilde{\nu}$ = 2960 (w), 2875 (m), 2329 (w), 1739 (s), 1497 (w), 1454 (m), 1404 (w), 1363 (w), 1258 (w), 1205 (w), 1173 (w), 1099 (s), 1032 (w), 912 (w), 873 (w), 740 (m), 698 (s), 615 (s), 586 (s). **^{1}H NMR (400 MHz, $CDCl_3$)**: δ = 7.39–7.26 (m, 5H), 4.52 (s, 2H), 3.34–3.27 (m, 2H), 2.41–2.22 (m, 3H), 2.10–1.94 (m, 2H), 1.78–1.66 (m, 1H), 1.11 (s, 3H). **^{13}C NMR (101 MHz, $CDCl_3$)**: δ = 219.8, 138.5, 128.5, 127.7, 127.5, 77.9, 73.4, 49.4, 40.7, 37.4, 32.7, 24.2. **HRMS (ESI)**: Calculated for $C_{14}H_{19}O_2^+$ $[M+H]^+$: 219.1380, found 219.1375.

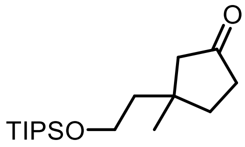

3-Methyl-3-(2-((triisopropylsilyl)oxy)ethyl)cyclopentan-1-one [2t]

Following general procedure **E** using MCB **1t** (85 mg, 0.30 mmol, 1.00 eq.) the desired product was obtained *via* FC (CH_2Cl_2) as a colorless oil (69 mg, 0.23 mmol, 77%).[4]

IR (neat): $\tilde{\nu}$ = 3492 (br), 2944 (s), 2868 (s), 2245 (w), 1744 (s), 1463 (m), 1403 (w), 1382 (w), 1255 (w), 1165 (m), 1098 (s), 1013 (m), 997 (m), 912 (m), 882 (s), 811 (m), 746 (m), 677 (s), 641 (s), 607 (s). **^{1}H NMR (400 MHz, $CDCl_3$)**: δ = 3.84–3.79 (m, 1H), 3.79–3.73 (m, 1H), 2.32–2.25 (m, 2H), 2.24–2.12 (m, 1H), 2.12–2.02 (m, 1H), 1.95–1.85 (m, 1H), 1.82–1.74 (m, 1H), 1.69 (t, J = 6.7 Hz, 2H), 1.08 (s, 3H), 1.07–1.03 (m, 21H). **^{13}C NMR (101 MHz, $CDCl_3$)**: δ = 220.3, 60.4, 52.9, 44.3, 38.6, 36.7, 36.0, 25.3, 18.2, 17.8 (TIPSOH), 12.4 (TIPSOH), 12.1. **HRMS (ESI)**: Calculated for $C_{17}H_{34}O_2SiNa^+$ $[M+Na]^+$: 321.2220, found 321.2218.

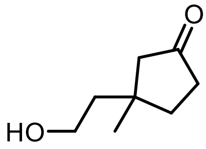

3-(2-Hydroxyethyl)-3-methylcyclopentan-1-one [2u]

Following general procedure **E** using MCB **1u** (40 mg, 0.29 mmol, 1.00 eq.) the desired product was obtained *via* FC (CH_2Cl_2:MeOH; 95:5) as a mixture with its hemiketal[5] (colorless oil, 40 mg, 0.28 mmol, 97%).

[4] Deprotection was partly observed, resulting in minor impurities with TIPSOH.

[5] 66:34 ketone:hemiketal according to ^{1}H NMR.

IR (neat): $\tilde{\nu}$ = 3451 (br), 2952 (m), 2359 (w), 1737 (s), 1461 (w), 1405 (m), 1378 (w), 1324 (w), 1248 (w), 1168 (m), 1125 (m), 1064 (m), 1028 (m), 993 (w), 924 (w), 876 (w), 774 (w), 736 (m). **^{1}H NMR (400 MHz, $CDCl_3$)**: δ = 3.82 – 3.68 (m, 2H), 2.32 – 2.24 (m, 2H), 2.21 – 2.10 (m, 1H), 2.10 – 2.00 (m, 1H), 1.91 – 1.74 (m, 2H), 1.71 (t, *J* = 7.2 Hz, 2H), 1.07 (s, 3H). **^{13}C NMR (101 MHz, $CDCl_3$)**: δ = 219.9, 59.7, 52.5, 44.0, 38.4, 36.5, 35.7, 25.2. **HRMS (ESI)**: Calculated for $C_8H_{15}O_2^+$ $[M+H]^+$: 143.1067, found 143.1070.

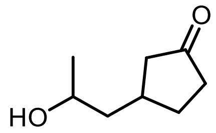

3-(2-Hydroxypropyl)cyclopentan-1-one [2v]

Following general procedure **E** using MCB **1v** (38 mg, 0.30 mmol, 1.00 eq.) the desired product was obtained *via* FC (CH_2Cl_2:MeOH; 99:1) as colorless oil (mixture of diastereomers, *dr* = 50:50 according to ^{13}C NMR; 27 mg, 0.19 mmol, 64%).

IR (neat): $\tilde{\nu}$ = 3505 (br), 2963 (m), 2917 (m), 2335 (w), 1736 (s), 1461 (w), 1405 (m), 1371 (w), 1309 (w), 1277 (w), 1237 (w), 1160 (m), 1056 (w), 1017 (w), 981 (w), 938 (w), 869 (w), 840 (w), 650 (m), 637 (m), 617 (m), 609 (s), 603 (s), 593 (m), 585 (m). **^{1}H NMR (400 MHz, $CDCl_3$)**: δ = 3.95–3.77 (m, 1H), 2.48–2.23 (m, 3H), 2.23–2.06 (m, 2H), 1.88 – 1.70 (m, 2H), 1.70–1.56 (m, 1H), 1.56–1.42 (m, 2H), 1.21 (d, *J* = 6.2 Hz, 3H). **^{13}C NMR (101 MHz, $CDCl_3$)**: δ = 219.9, 219.8, 66.7, 66.5, 45.7, 45.2, 45.1, 45.1, 38.7, 38.5, 34.2, 34.1, 30.1, 29.6, 24.4, 24.3. **HRMS (ESI)**: Calculated for $C_8H_{15}O_2^+$ $[M+H]^+$: 143.1067, found 143.1067.

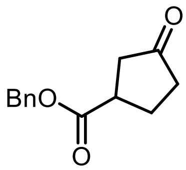

Benzyl 3-oxocyclopentane-1-carboxylate [2w]

Following procedure **E** using MCB **1w** (60.6 mg, 0.30 mmol, 1.00 eq.) the desired product was obtained *via* FC (pentane:Et_2O; 10:1) as colorless oil (59.6 mg, 0.273 mmol, 91%).

^{1}H NMR (400 MHz, $CDCl_3$): δ = 7.42 – 7.30 (m, 5H), 5.16 (s, 2H), 3.25 – 3.09 (m, 1H), 2.60 – 2.42 (m, 2H), 2.43 – 2.27 (m, 2H), 2.27 – 2.07 (m, 2H). **^{13}C NMR (101 MHz,**

$CDCl_3$): *δ* = 216.5, 174.2, 135.8, 128.8, 128.6, 128.3, 66.9, 41.2, 41.1, 37.6, 26.7. Spectroscopic data was in agreement with that previously reported.[42]

5.3 Unsuccessful substrates

Submission of 1-bromo-4-(2-methylenecyclopropyl)benzene [**S28**] and (4-methylene-cyclohexyl)benzene [**S29**] to the optimized conditions only yielded unreacted starting material.[6] Conversion towards the corresponding ketone or aldehyde products was not observed.

5.4 Stereochemical analysis

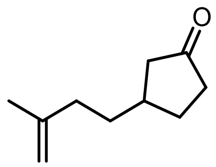

3-(3-Methylbut-3-en-1-yl)cyclopentan-1-one [2x]

Following procedure **E** using MCB **1x** (40.9 mg, 0.30 mmol, 1.00 eq.) the desired product was obtained *via* FC (pentane:Et_2O; 25:1) as colorless oil (41.6 mg, 0.273 mmol, 91%).

IR (neat): $\tilde{\nu}$ = 3074 (w), 2963 (m), 2925 (m), 1740 (s), 1649 (m), 1450 (m) 1405 (m), 1375 (w), 1240 (w), 886 (m), 727 (w). **^{1}H NMR (599 MHz, $CDCl_3$)**: *δ* = 4.74 – 4.65 (m, 2H), 2.44 – 2.36 (m, 1H), 2.34 – 2.26 (m, 1H), 2.21 – 2.10 (m, 3H), 2.06 (td, *J* = 7.9, 3.0 Hz, 2H), 1.85 – 1.77 (m, 1H), 1.73 (s, 3H), 1.65 – 1.53 (m, 2H), 1.55 – 1.47 (m, 1H). **^{13}C NMR (151 MHz, $CDCl_3$)**: *δ* = 219.8, 145.5, 110.3, 45.3, 38.7, 36.9, 36.1, 33.7, 29.6, 22.5. **HRMS (EI)**: Calculated for $C_{10}H_{16}O^+$ $[M]^+$: 152.1196, found: 152.1194.

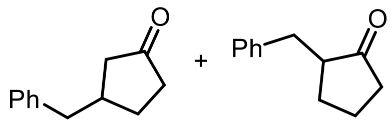

3-Benzylcyclopentan-1-one [6], 2-benzylcyclopentan-1-one [7]

Following general procedure **E** using MCB **5** (57 mg, 0.30 mmol, 1.00 eq.), two products were obtained *via* FC (CH_2Cl_2) as pale-yellow oils: 3-benzylcyclopentan-1-

[6] 58% and 78%, respectively, according to ^{1}H NMR analysis of the crude reaction mixture.

one (**6,** 16 mg, 92 µmol, 31%) and 2-benzylcyclopentan-1-one (**7,** 19 mg, 0.11 mmol, 36%).[7]

Major regioisomer [6]: ^{1}H NMR (400 MHz, $CDCl_3$): δ = 7.34–7.27 (m, 2H), 7.25–7.14 (m, 3H), 2.79–2.69 (m, 2H), 2.55–2.41 (m, 1H), 2.39–2.24 (m, 2H), 2.20–2.05 (m, 2H), 1.96–1.87 (m, 1H), 1.70–1.56 (m, 1H). **^{13}C NMR (101 MHz, $CDCl_3$)**: δ = 219.4, 140.1, 128.9, 128.6, 126.4, 45.1, 41.6, 39.0, 38.5, 29.2. Spectroscopic data was in agreement with that previously reported.[43]

Minor regioisomer [7]: ^{1}H NMR (400 MHz, $CDCl_3$): δ = 7.31–7.25 (m, 2H), 7.23–7.15 (m, 3H), 3.15 (dd, J = 13.8, 4.1 Hz, 1H), 2.54 (dd, J = 13.8, 9.5 Hz, 1H), 2.41–2.29 (m, 2H), 2.19–2.03 (m, 2H), 2.02–1.89 (m, 1H), 1.81–1.66 (m, 1H), 1.62 – 1.50 (m, 1H). **^{13}C NMR (101 MHz, $CDCl_3$)**: δ = 220.4, 140.1, 129.0, 128.6, 126.3, 51.2, 38.4, 35.7, 29.3, 20.7. Spectroscopic data was in agreement with that previously reported.[44]

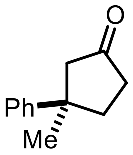

(+)-3-Methyl-3-phenylcyclopentan-1-one [(+)-2j]

A tube was charged with $PdCl_2(MeCN)_2$ (5.2 mg, 0.020 mmol, 0.10 eq.) and ligand **L1** (6.0 mg, 0.022 mmol, 0.11 eq.). Then, CH_2Cl_2 (3 mL) was added and the mixture was heated to reflux until a clear solution had formed (about 5 min.). The solvent was removed *in vacuo* and the residue dried under vacuum. Dry EtOH (2 mL) was added and the suspension was heated to 60 °C. Silver perchlorate hydrate (5.0 mg, 0.022 mmol, 0.11 eq.) was added. Water (0.11 mL, 6.0 mmol, 30.0 eq.), MCB **1j** (32 mg, 0.20 mmol, 1.00 eq.) and *t*BuONO (90% purity, 27 µL, 0.20 mmol, 1.00 eq.) were added successively. The tube was sealed and heated to 78 °C under stirring for 18 h. After cooling to rt the mixture was filtered through a whatman glas fibre filter and the solvent was removed *in vacuo*. The desired product was obtained *via* FC (hexane:Et_2O; 100:0→90:10) as a colorless oil (28 mg, 0.16 mmol, 80%).

^{1}H NMR (600 MHz, $CDCl_3$): δ = 7.41–7.35 (m, 2H), 7.33–7.30 (m, 2H), 7.29–7.24 (m, 1H), 2.68 (d, J = 17.6 Hz, 1H), 2.51 (d, J = 17.6 Hz, 1H), 2.48–2.27 (m, 4H), 1.41 (s, 3H). **^{13}C NMR (151 MHz, $CDCl_3$)**: δ = 218.8, 148.6, 128.7, 126.4, 125.5, 52.3, 43.9,

[7] Regioselectivitiy: 62:38 – **6**:**7** according to ^{1}H NMR analysis of the crude reaction mixture.

36.8, 35.8, 29.5. **Optical Rotation**: $[\alpha]_D^{25}$ = +5.2 (*c* = 1.00, $CHCl_3$) for an enantiomerically enriched sample of 63:37 *er*, the major enantiomer is (*R*)-configured, assigned in analogy to literature ($[\alpha]_D$ = +23.0° (*c* = 0.85, $CHCl_3$), 99.5:0.5 *er* in favor of the (*R*)-enantiomer).[45] The enantiomeric purity was established by HPLC analysis using a chiral column (Lux® i-Cellulose-5 column, 22 °C, 1 mL/min, 95:5 hexane:isopropanol, 210 nm, t_R(major) = 11.4 min, t_R(minor) = 12.5 min). Spectroscopic data was in agreement with that previously reported.[45,46]

[*rac*-2j]

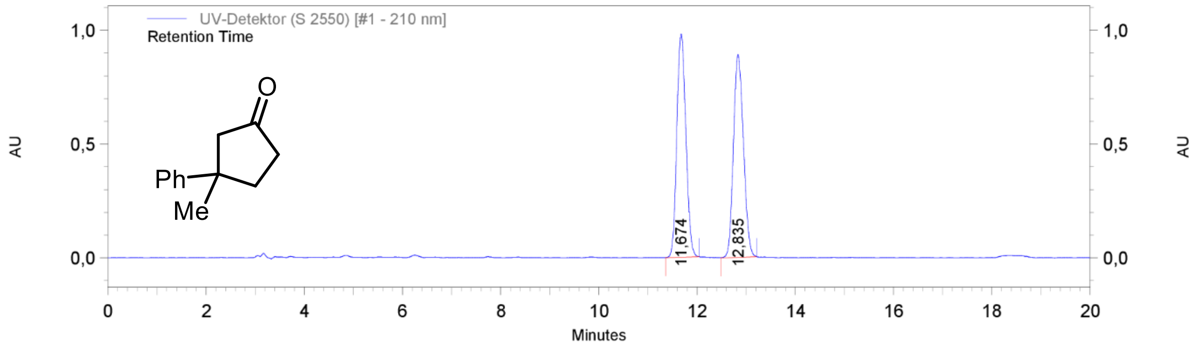

UV-Detektor (S 2550) [#1 - 210 nm] Results

Retention Time	Area	Area %
11,674	12558275	49,91
12,835	12601237	50,09

[(+)-2j]

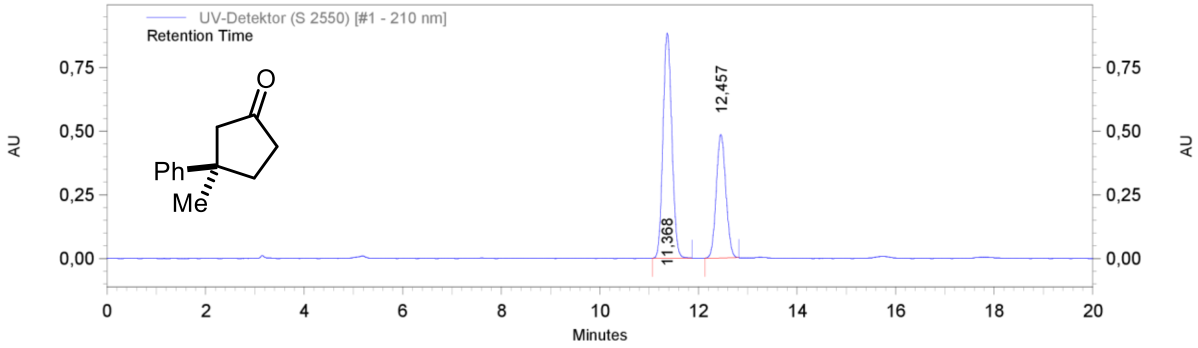

UV-Detektor (S 2550) [#1 - 210 nm] Results

Retention Time	Area	Area %
11,368	11048499	62,60
12,457	6600172	37,40

6 References

[1] A. V. Chernykh, D. S. Radchenko, A. V. Chernykh, I. S. Kondratov, N. A. Tolmachova, O. P. Datsenko, M. A. Kurkunov, S. X. Zozulya, Y. P. Kheylik, K. Bartels et al., *Eur. J. Org. Chem.* **2015**, 6466.

[2] A. V. Malkov, F. Friscourt, M. Bell, M. E. Swarbrick, P. Kocovský, *J. Org. Chem.* **2008**, *73*, 3996.

[3] Z. Du, M. J. Haglund, L. A. Pratt, K. L. Erickson, *J. Org. Chem.* **1998**, *63*, 8880.

[4] M. Nanko, Y. Inaba, K. Sekine, K. Mikami, *Helv. Chim. Acta* **2021**, *104*.

[5] J. Huang, G. Hu, S. An, D. Chen, M. Li, P. Li, *J. Org. Chem.* **2019**, *84*, 9758.

[6] M. Lux, M. Klussmann, *Org. Lett.* **2020**, *22*, 3697.

[7] A. Horn, U. Kazmaier, *Org. Lett.* **2019**, *21*, 4595.

[8] J. Sietmann, M. Ong, C. Mück-Lichtenfeld, C. G. Daniliuc, J. M. Wahl, *Angew. Chem.* **2021**, *133*, 9805.

[9] M. Zhang, J. Gao, J. Zhao, T. Qiu, Z. Li, Z. Guo, C. Liu, Y. Liu, *Eur. J. Org. Chem.* **2021**, *2021*, 6111.

[10] T. Matsuda, I. Yuihara, *Chem. Commun.* **2015**, *51*, 7393.

[11] X. Wu, G. Ding, W. Lu, L. Yang, J. Wang, Y. Zhang, X. Xie, Z. Zhang, *Org. Lett.* **2021**, *23*, 1434.

[12] Y. Zhou, C. Rao, Q. Song, *Org. Lett.* **2016**, *18*, 4000.

[13] Y. Chen, J. Du, Z. Zuo, *Chem* **2020**, *6*, 266.

[14] Y. Takahashi, H. Ohaku, S. Morishima, T. Suzuki, H. Ikeda, T. Miyashi, *J. Chem. Soc., Perkin Trans. 1* **1996**, 319.

[15] E. W. Della, P. E. Pigou, C. H. Schiesser, D. K. Taylor, *J. Org. Chem.* **1991**, *56*, 4659.

[16] J. B. Aggen, A. A. Goldblum, D. J. Hildebrandt, T. R. Kane, P. Dozzo, M. J. Gliedt, H. E. Moser (Achaogen Inc.), WO2010042850A1, **2010**.

[17] M. F. Brown, A. E. Fenwick, M. E. Flanagan, A. Gonzales, T. A. Johnson, N. Kaila, M. J. Mitton-Fry, J. W. Strohbach, R. E. Tenbrink, J. D. Trzupek et al. (Pfizer Inc.), US2014243312A1, **2014**.

[18] S. J. Cowling, J. W. Goodby, *Chem. Commun.* **2006**, 4107.

[19] A. Malashchuk, A. V. Chernykh, M. Y. Perebyinis, I. V. Komarov, O. O. Grygorenko, *Eur. J. Org. Chem.* **2021**, *2021*, 6570.

[20] H. Qin, W. Cai, S. Wang, T. Guo, G. Li, H. Lu, *Angew. Chem. Int. Ed.* **2021**, *60*, 20678.

[21] A. Tsoukaki, E. Skolia, I. Triandafillidi, C. G. Kokotos, *Eur. J. Org. Chem.* **2022**, e202200463.

[22] A. B. Lutjen, M. A. Quirk, A. M. Barbera, E. M. Kolonko, *Bioorg. Med. Chem.* **2018**, *26*, 5291.

[23] C. Bolm, K. Weickhardt, M. Zehnder, T. Ranff, *Chem. Ber.* **1991**, *124*, 1173.

[24] E. W. Werner, T.-S. Mei, A. J. Burckle, M. S. Sigman, *Science* **2012**, *338*, 1455.

[25] C. Zhang, C. B. Santiago, J. M. Crawford, M. S. Sigman, *J. Am. Chem. Soc.* **2015**, *137*, 15668.

[26] A. J. Davenport, D. L. Davies, J. Fawcett, S. A. Garratt, D. R. Russell, *J. Chem. Soc., Dalton Trans.* **2000**, 4432.

[27] B. Su, T.-G. Zhou, X.-W. Li, X.-R. Shao, P.-L. Xu, W.-L. Wu, J. F. Hartwig, Z.-J. Shi, *Angew. Chem. Int. Ed.* **2017**, *56*, 1092.

[28] A. Cornejo, J. M. Fraile, J. I. García, M. J. Gil, V. Martínez-Merino, J. A. Mayoral, E. Pires, I. Villalba, *Synlett* **2005**, 2321.

[29] a) D. A. Evans, G. S. Peterson, J. S. Johnson, D. M. Barnes, K. R. Campos, K. A. Woerpel, *J. Org. Chem.* **1998**, *63*, 4541; b) A. Teichert, A. Pfaltz, *Angew. Chem. Int. Ed.* **2008**, *47*, 3360.

[30] Y. Uozumi, H. Kyota, E. Kishi, K. Kitayama, T. Hayashi, *Tetrahedron: Asymmetry* **1996**, *7*, 1603.

[31] X. Wei, W. Shu, A. García-Domínguez, E. Merino, C. Nevado, *J. Am. Chem. Soc.* **2020**, *142*, 13515.

[32] J. W. Wilt, R. A. Dabek, K. C. Welzel, *J. Org. Chem.* **1972**, *37*, 425.

[33] D. C. Braddock, J. Clarke, H. S. Rzepa, *Chem. Commun.* **2013**, *49*, 11176.

[34] D. P. Provencal, J. W. Leahy, *J. Org. Chem.* **1994**, *59*, 5496.

[35] T. Pecchioli, M. Christmann, *Org. Lett.* **2018**, *20*, 5256.

[36] Q. Lang, H. Yang, G. Gu, Q. Feng, J. Wen, X. Zhang, *Chin. J. Chem.* **2021**, *39*, 933.

[37] X. Feng, Y. Wang, B. Wei, J. Yang, H. Du, *Org. Lett.* **2011**, *13*, 3300.

[38] H. Wang, Y. Li, R. Zhang, K. Jin, D. Zhao, C. Duan, *J. Org. Chem.* **2012**, *77*, 4849.

[39] Q. An, Z. Wang, Y. Chen, X. Wang, K. Zhang, H. Pan, W. Liu, Z. Zuo, *J. Am. Chem. Soc.* **2020**, *142*, 6216.

[40] K. Kim, S. Lee, S. H. Hong, *Org. Lett.* **2021**, *23*, 5501.
[41] K. Miyazawa, T. Koike, M. Akita, *Adv. Synth. Catal.* **2014**, *356*, 2749.
[42] M. Nagatomo, K. Hagiwara, K. Masuda, M. Koshimizu, T. Kawamata, Y. Matsui, D. Urabe, M. Inoue, *Chem. Eur. J.* **2016**, *22*, 222.
[43] R. J. Phipps, L. McMurray, S. Ritter, H. A. Duong, M. J. Gaunt, *J. Am. Chem. Soc.* **2012**, *134*, 10773.
[44] X.-J. Dai, H. Wang, C.-J. Li, *Angew. Chem. Int. Ed.* **2017**, *56*, 6302.
[45] C. Hawner, D. Müller, L. Gremaud, A. Felouat, S. Woodward, A. Alexakis, *Angew. Chem. Int. Ed.* **2010**, *49*, 7769.
[46] R. C. Gadwood, *J. Org. Chem.* **1983**, *48*, 2098.

7 ^{1}H, ^{13}C and ^{19}F NMR spectra

[S4], ^{1}H, $CDCl_3$, 400 MHz

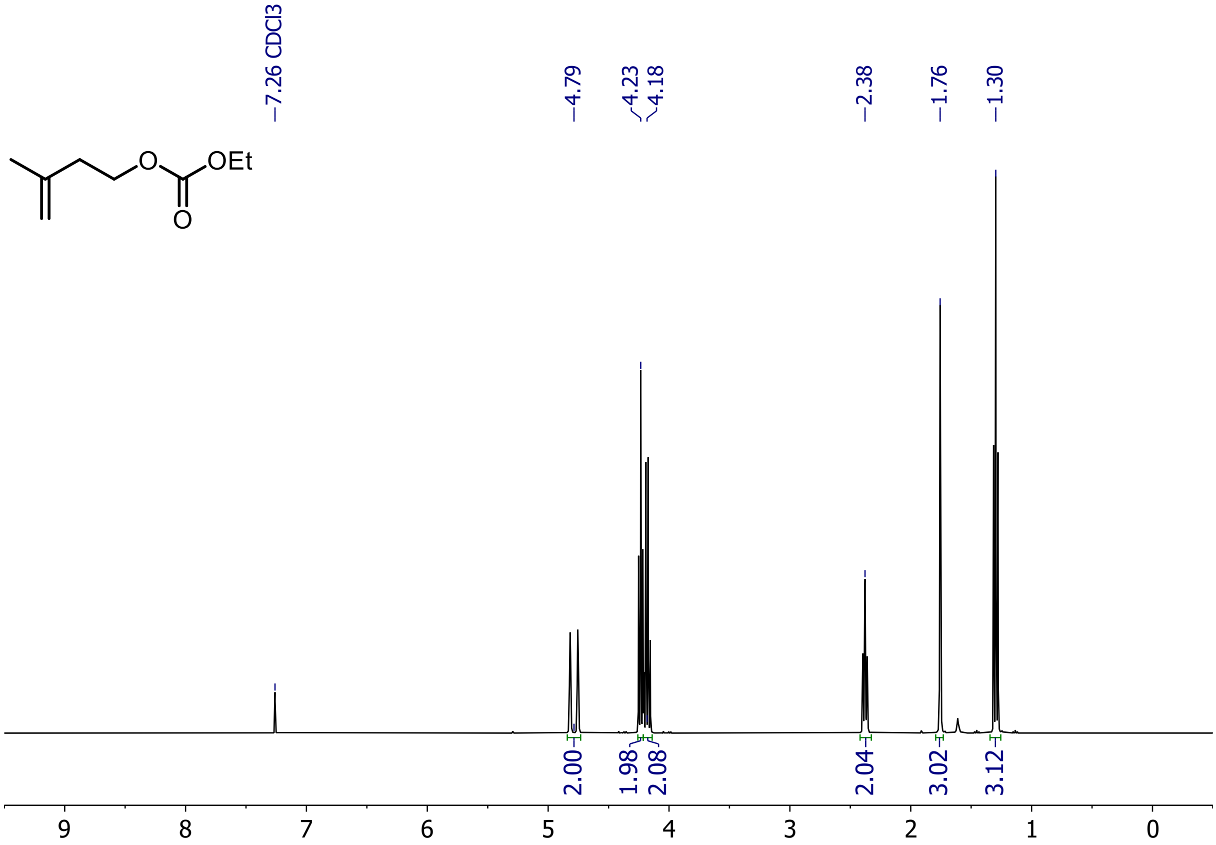

[S4], ^{13}C, $CDCl_3$, 101 MHz

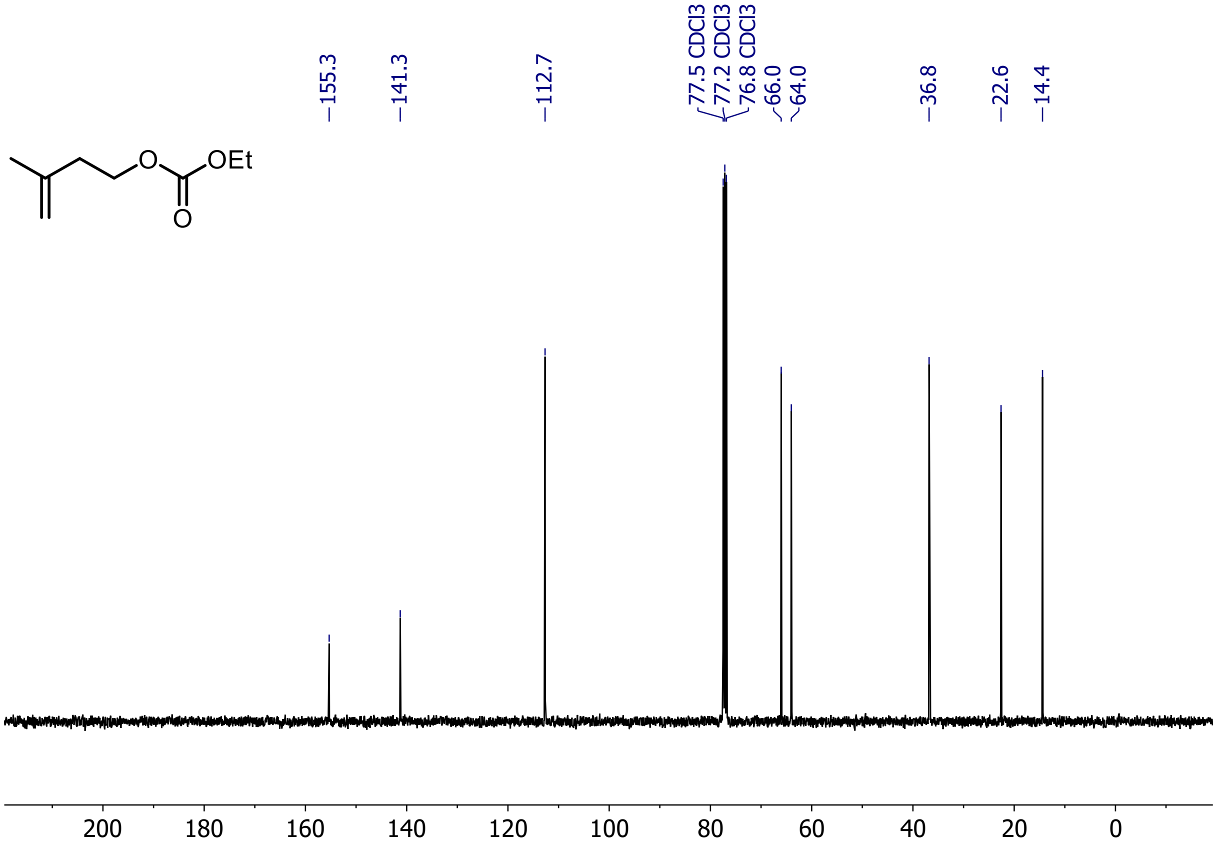

[S5], ^{1}H, $CDCl_3$, 400 MHz

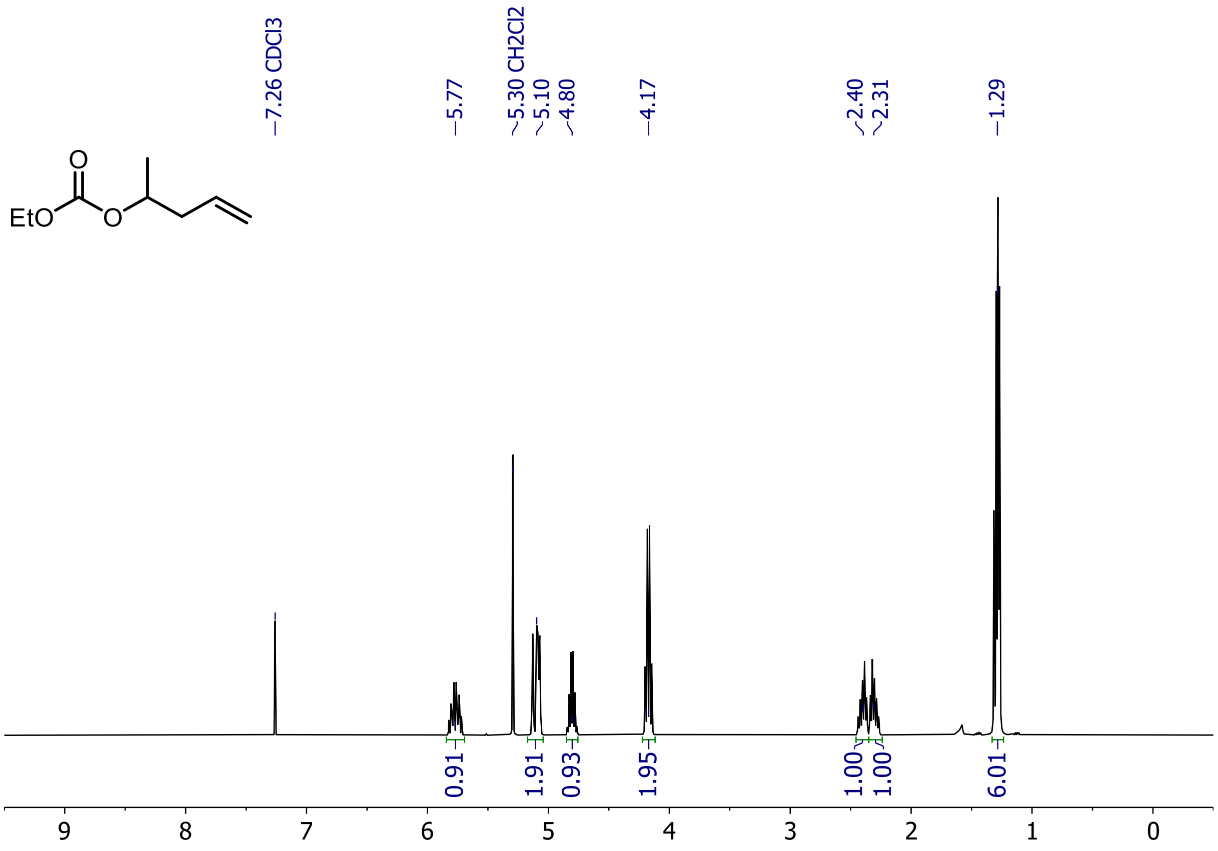

[S5], ^{13}C, $CDCl_3$, 101 MHz

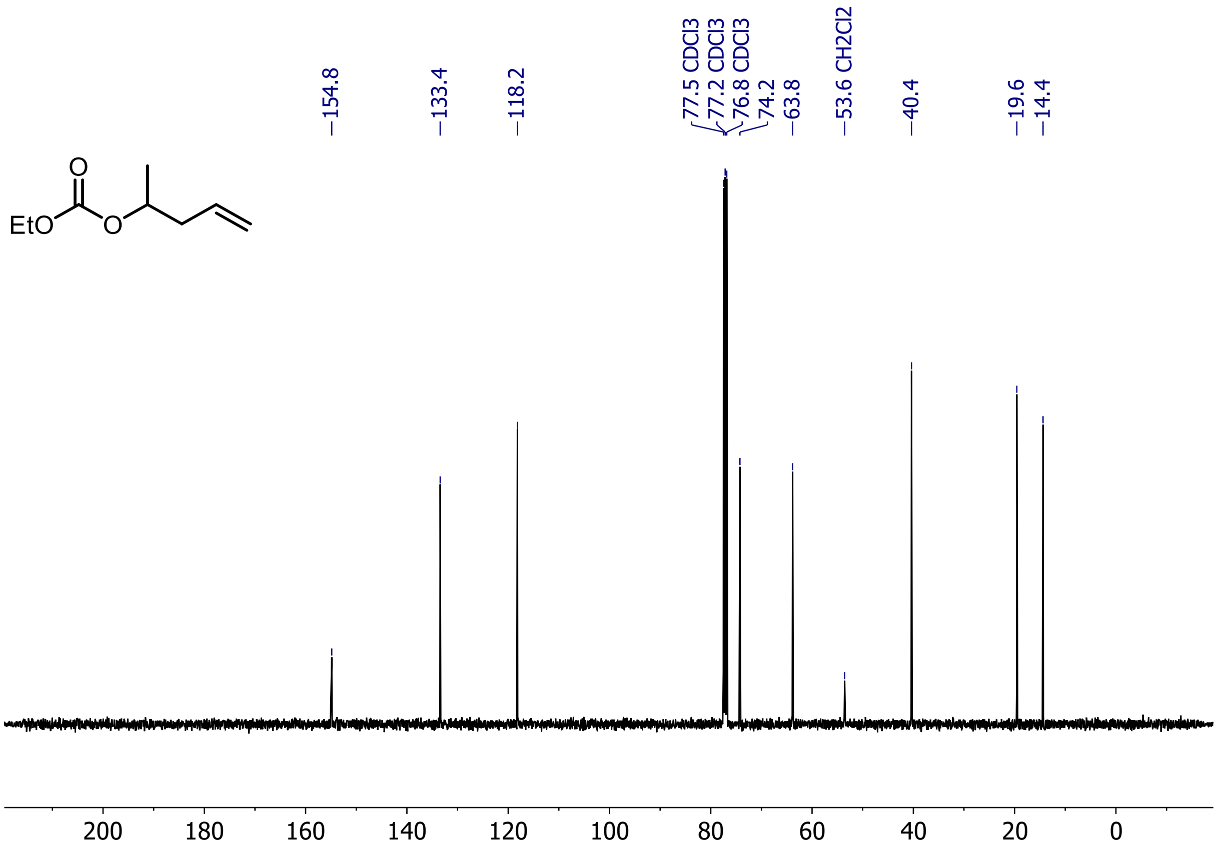

[S18], ^{1}H, $CDCl_3$, 599 MHz

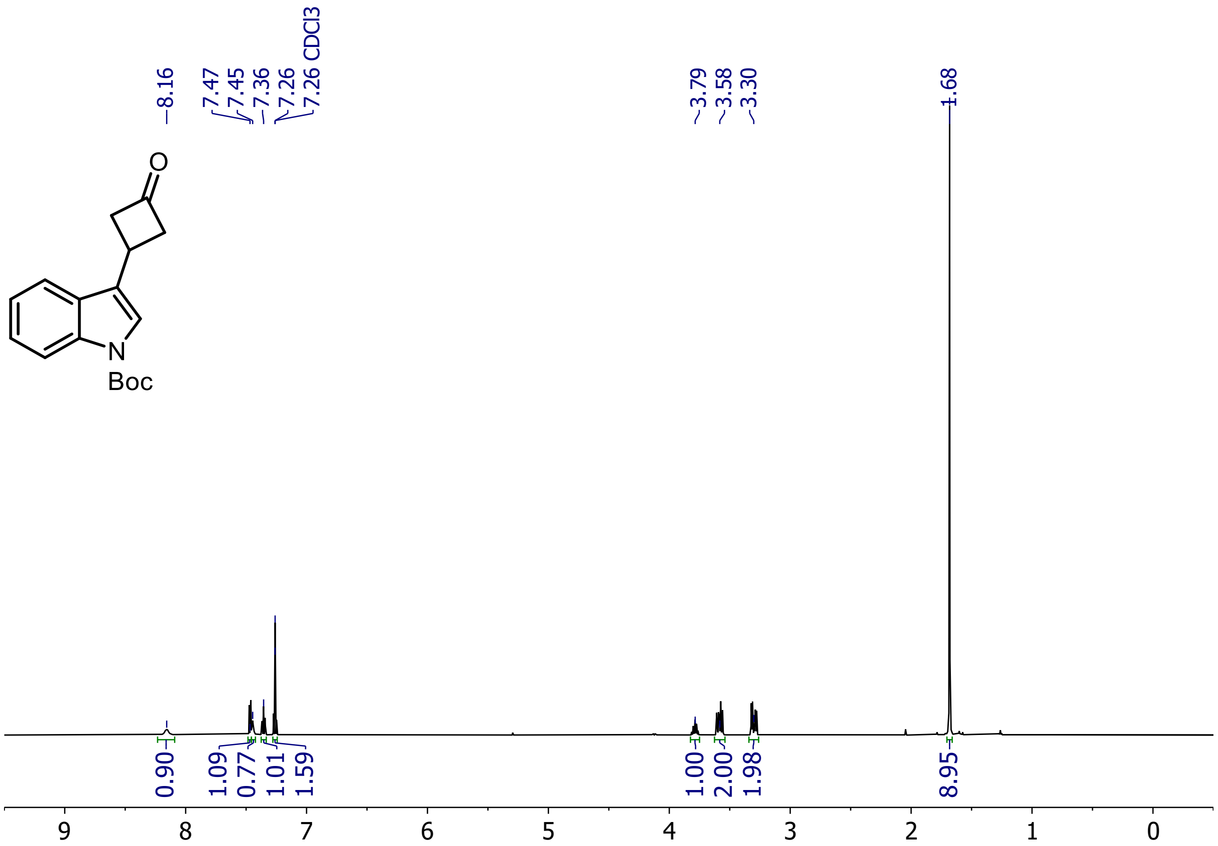

[S18], ^{13}C, $CDCl_3$, 151 MHz

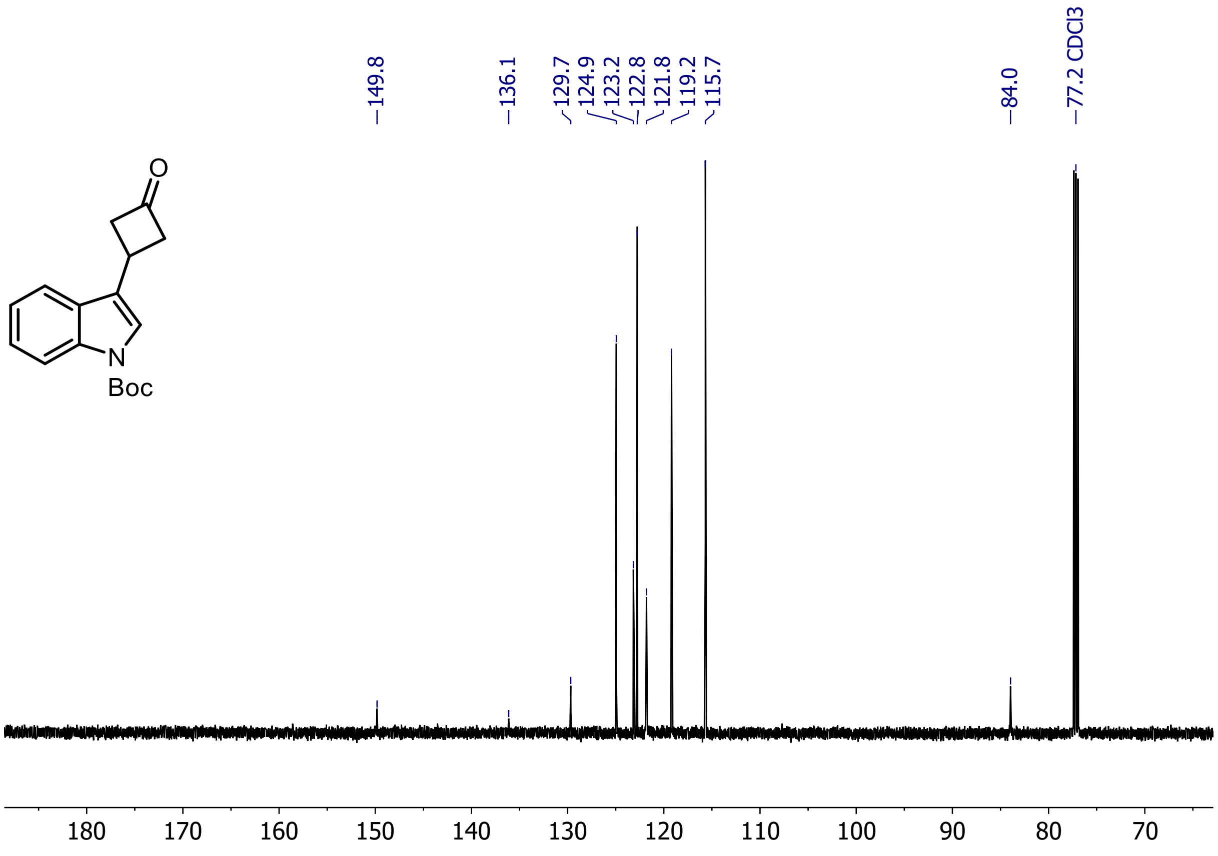

[S19], ^{1}H, $CDCl_3$, 599 MHz

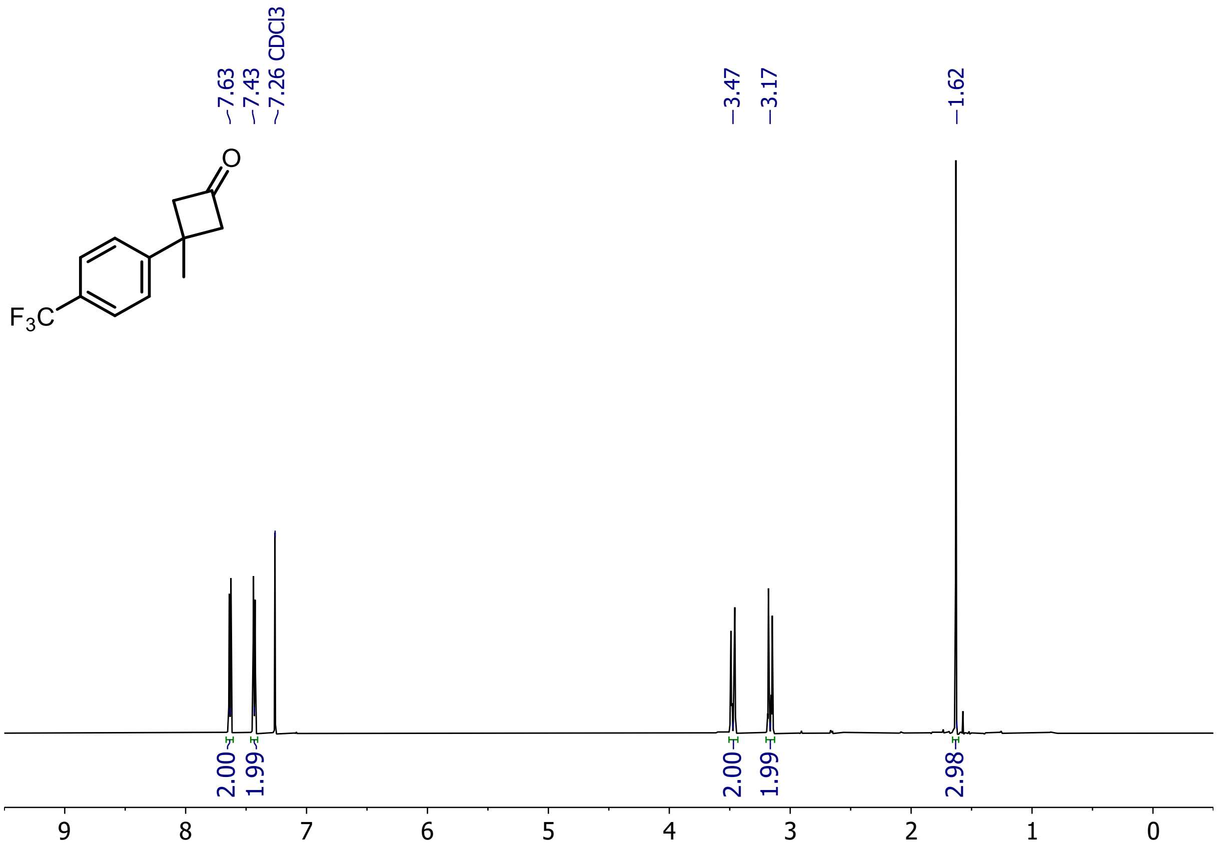

[S19], ^{13}C, $CDCl_3$, 151 MHz

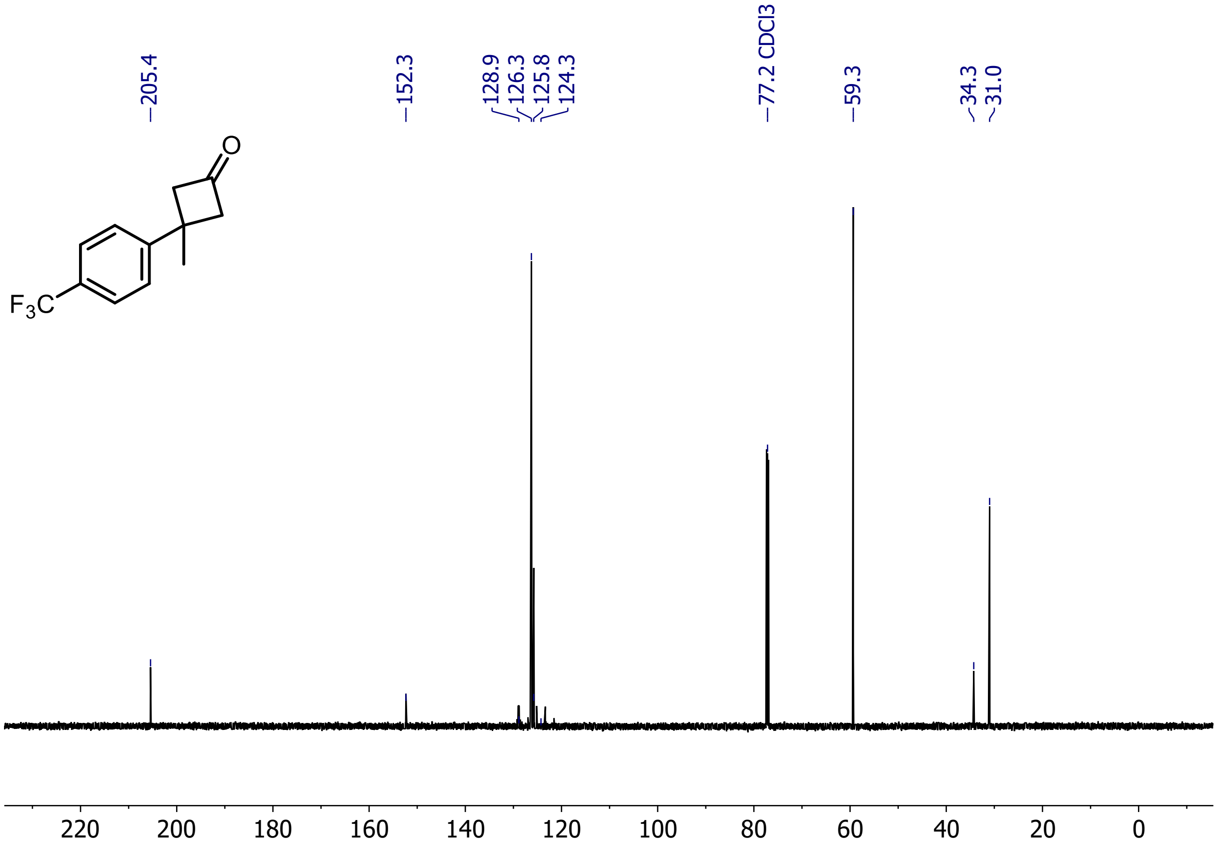

[S19], ^{19}F, $CDCl_3$, 377 MHz

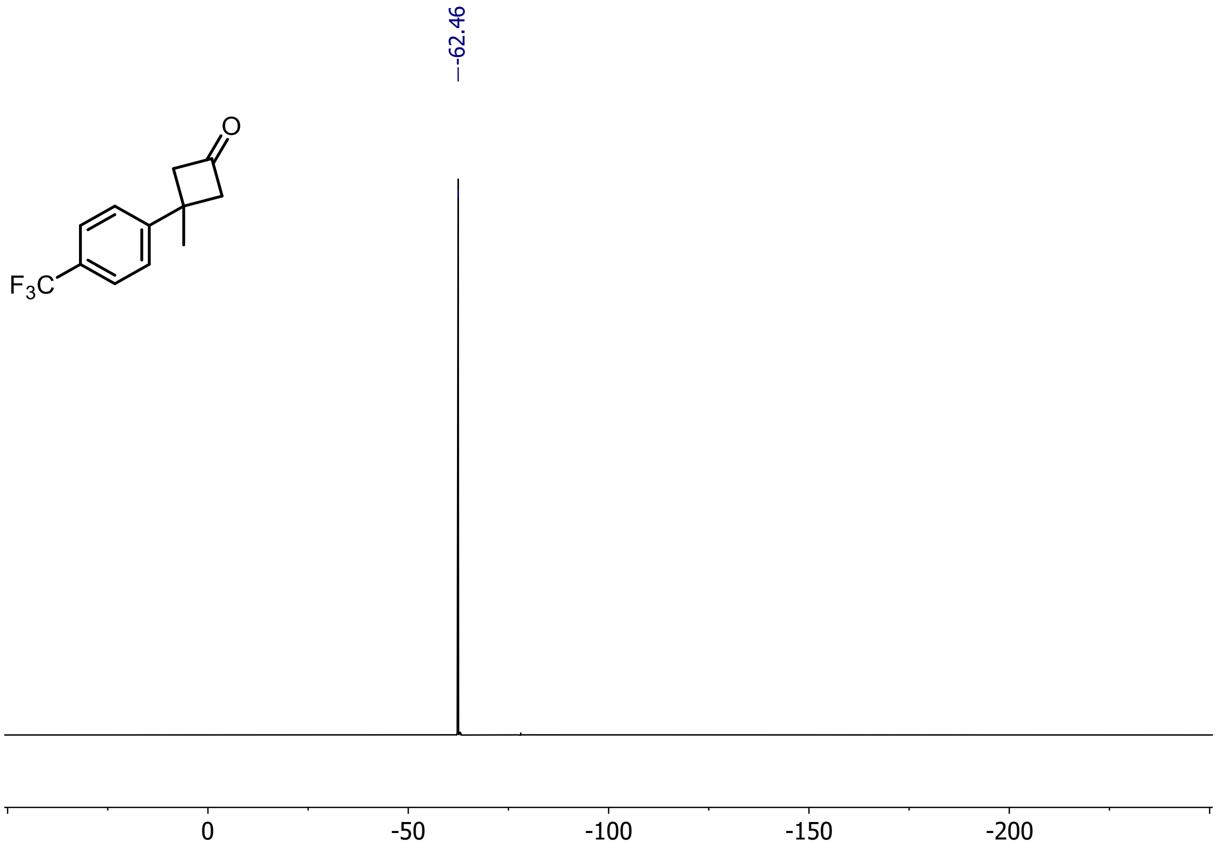

[S21], ^{1}H, $CDCl_3$, 599 MHz

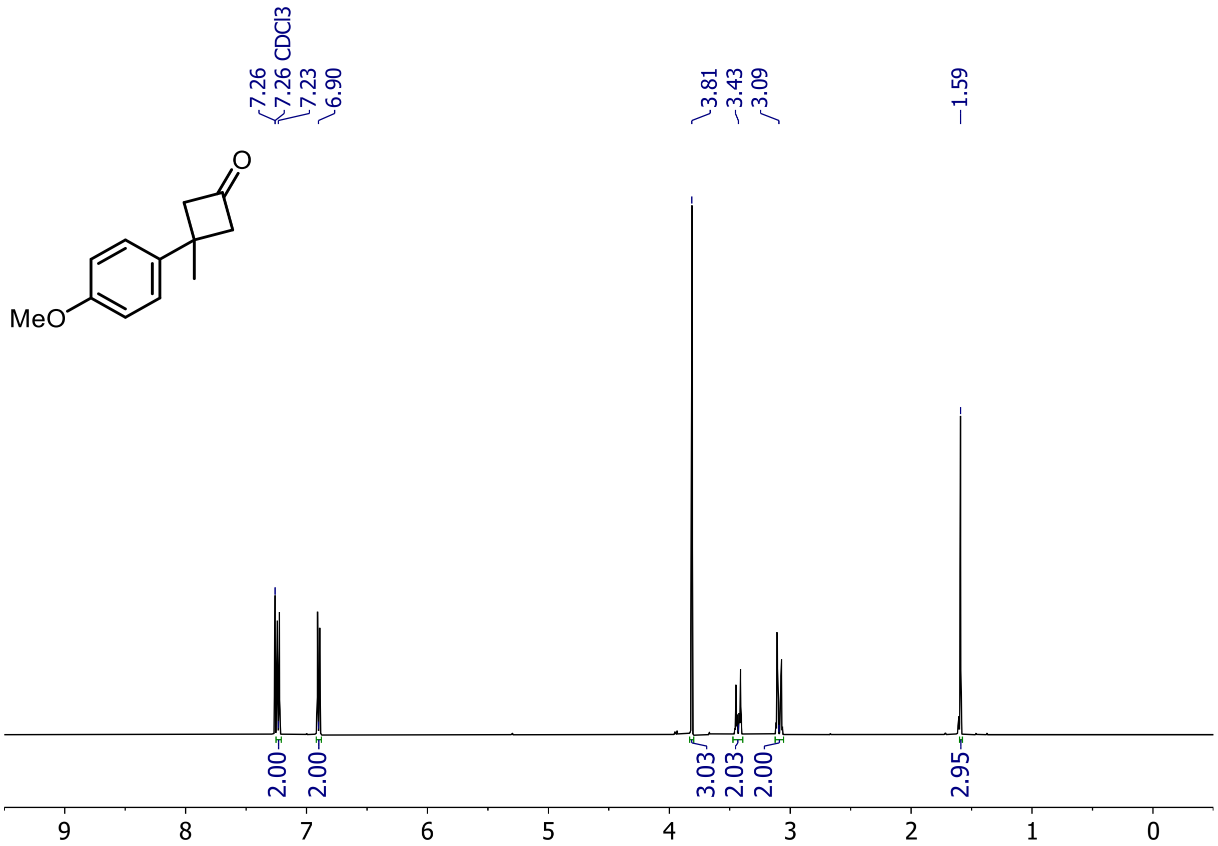

[S21], ^{13}C, $CDCl_3$, 151 MHz

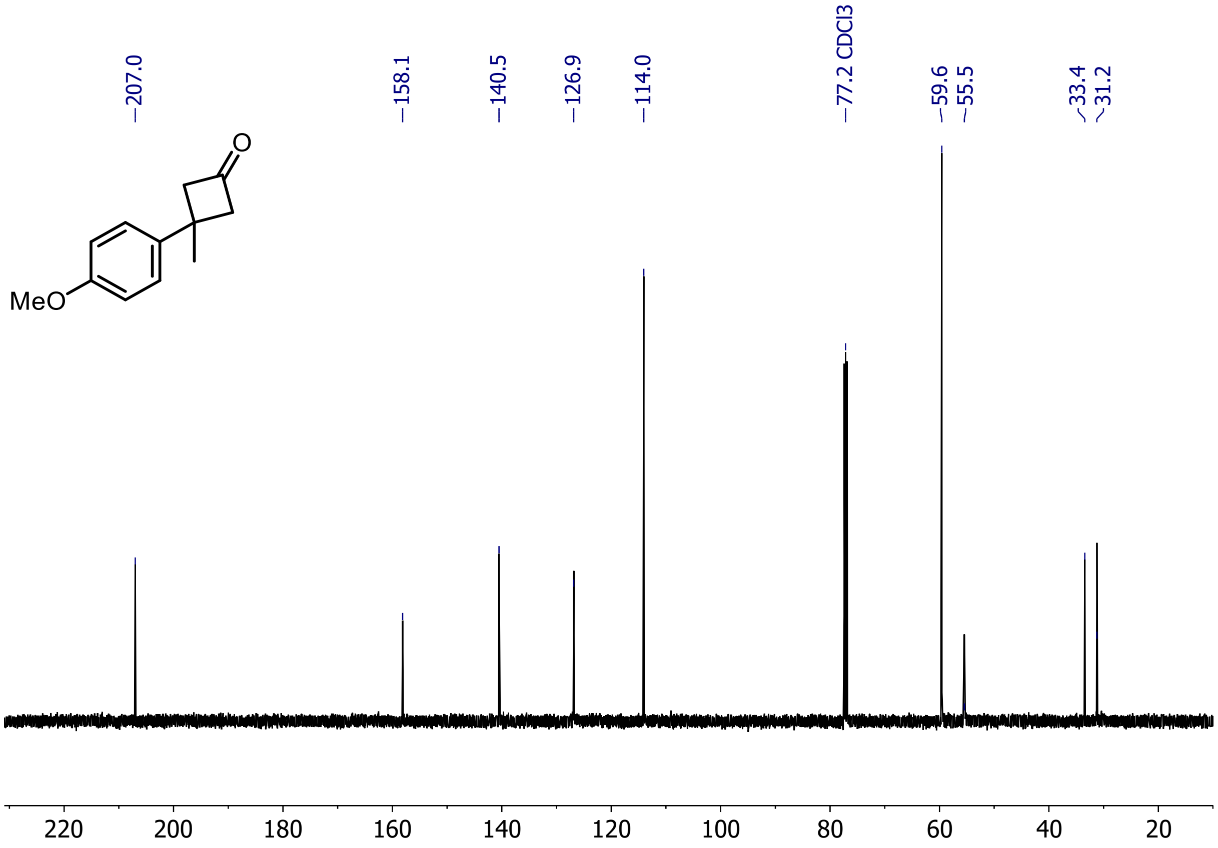

[S22], ^1H, $CDCl_3$, 400 MHz

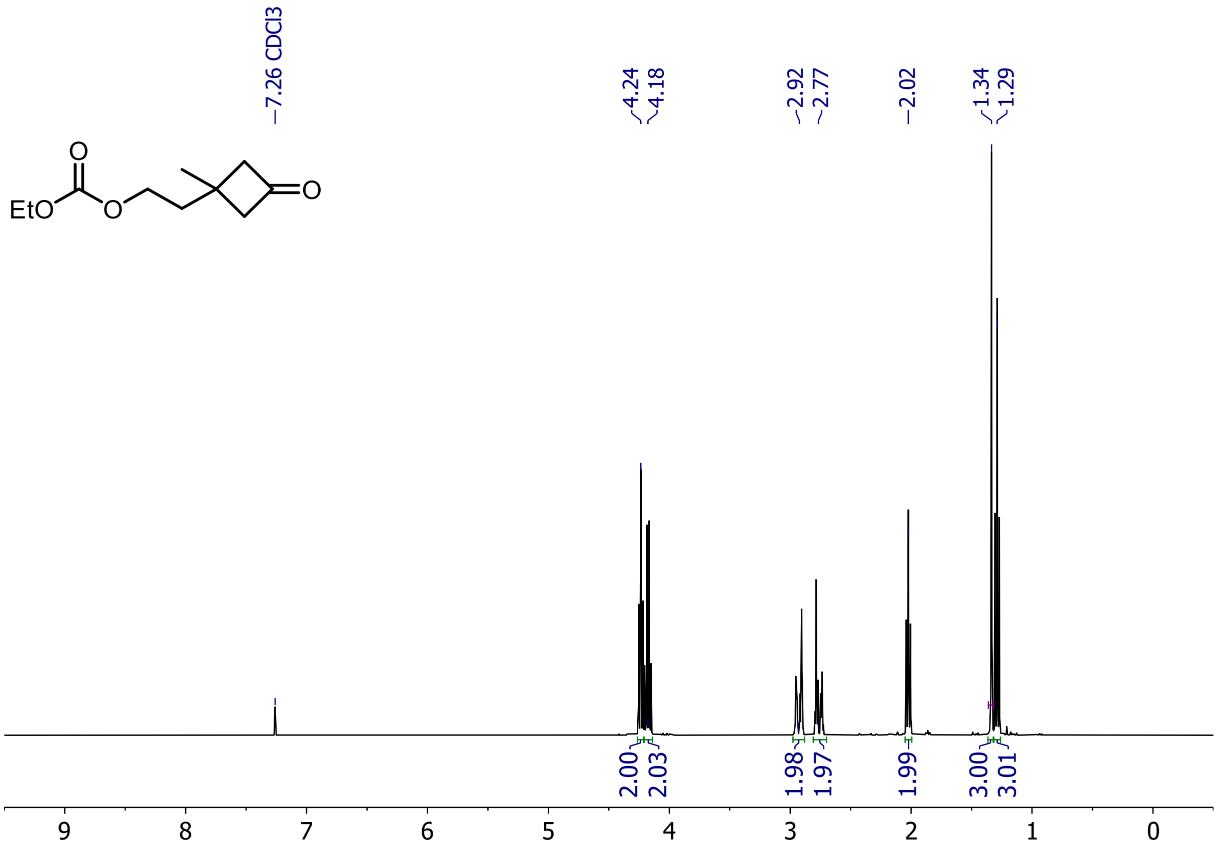

[S22], ^{13}C, $CDCl_3$, 101 MHz

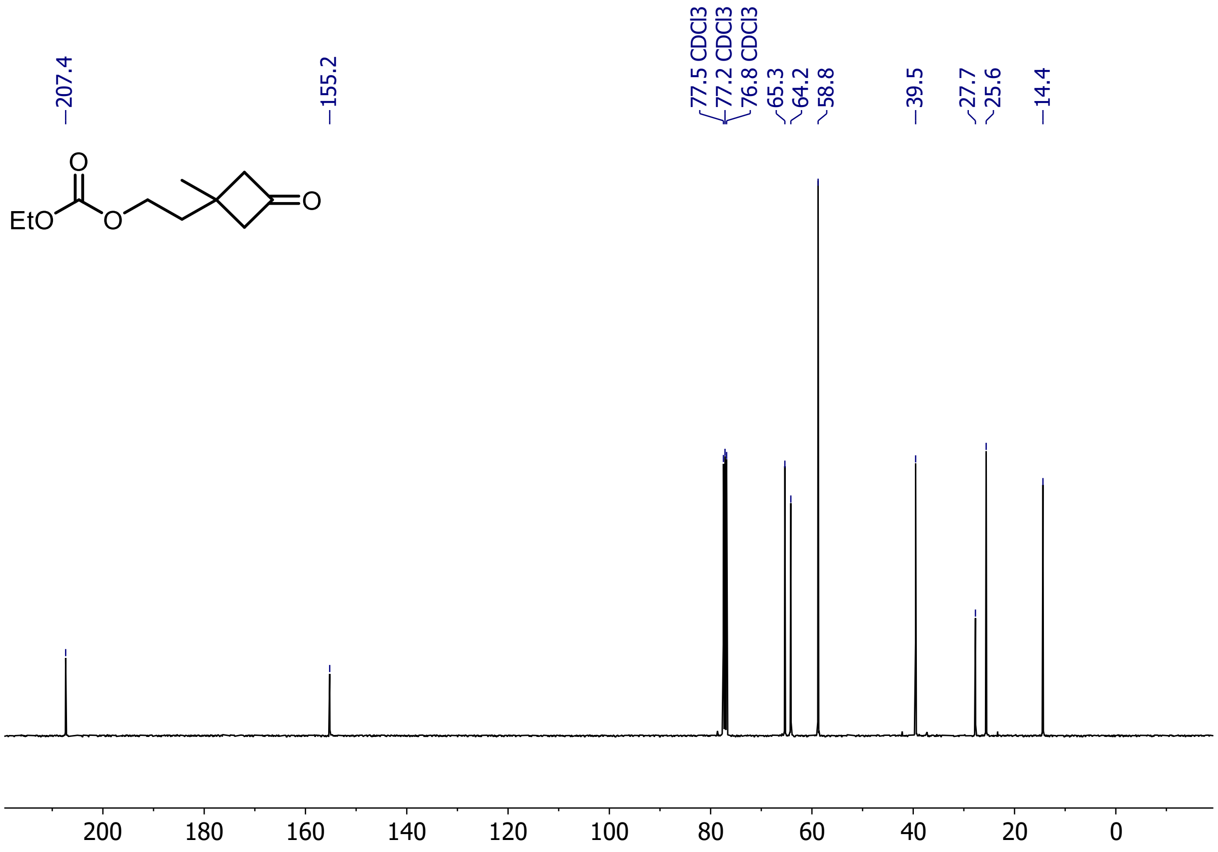

[S23], ^{1}H, $CDCl_3$, 400 MHz

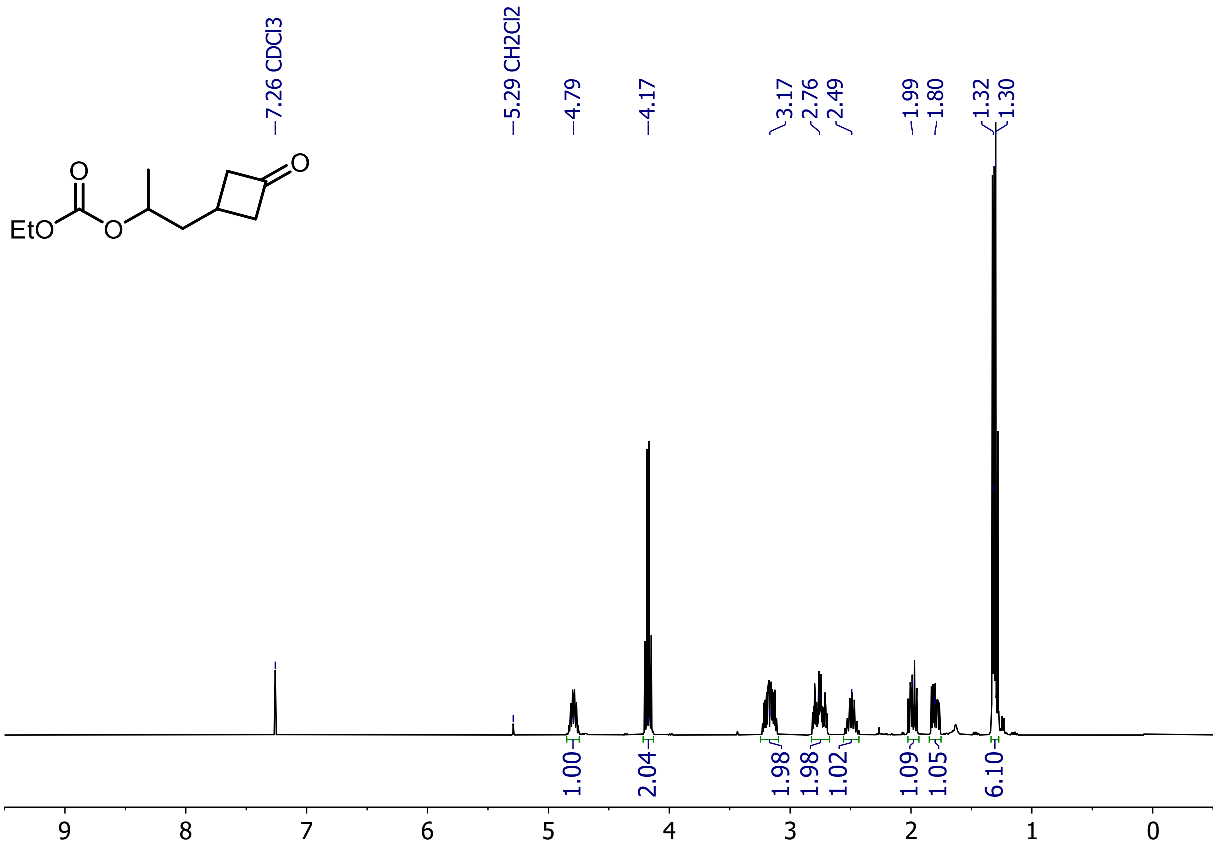

[S23], ^{13}C, $CDCl_3$, 101 MHz

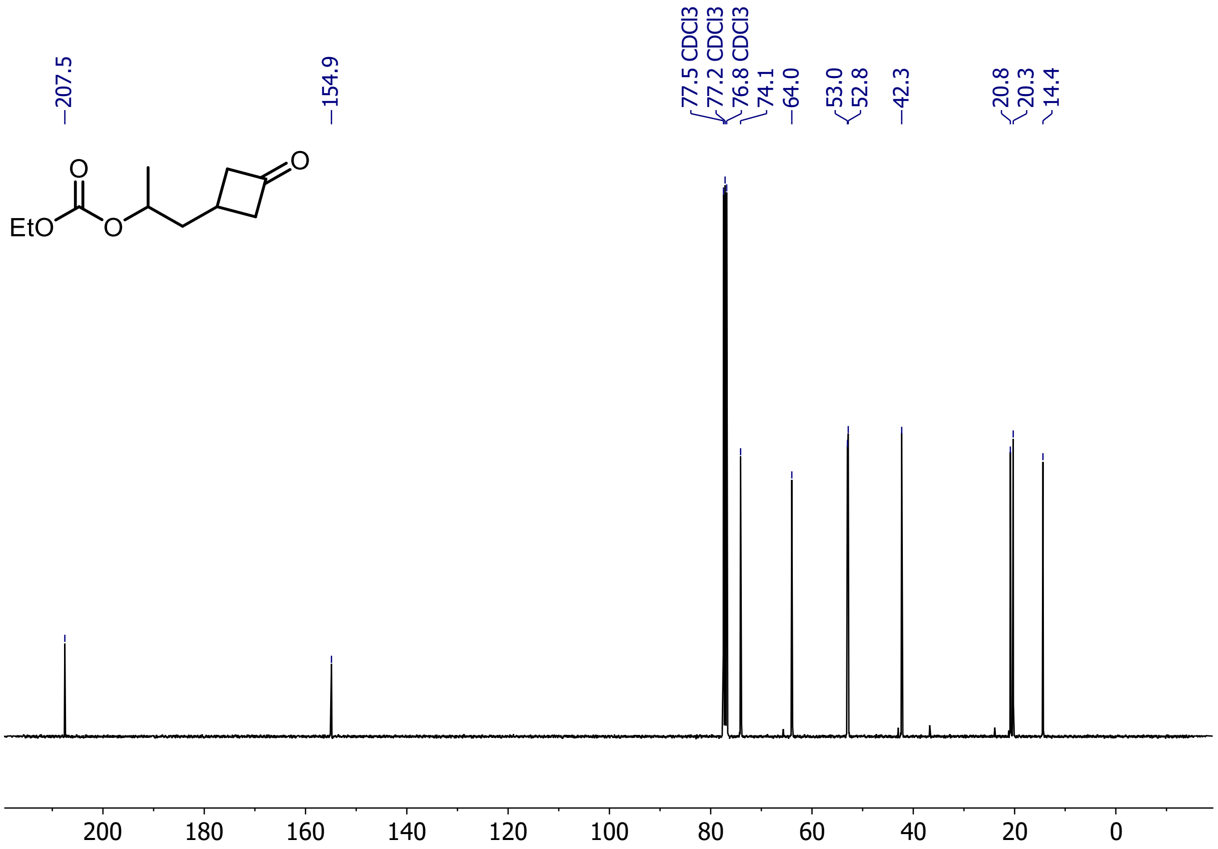

[S24], ^{1}H, $CDCl_3$, 599 MHz

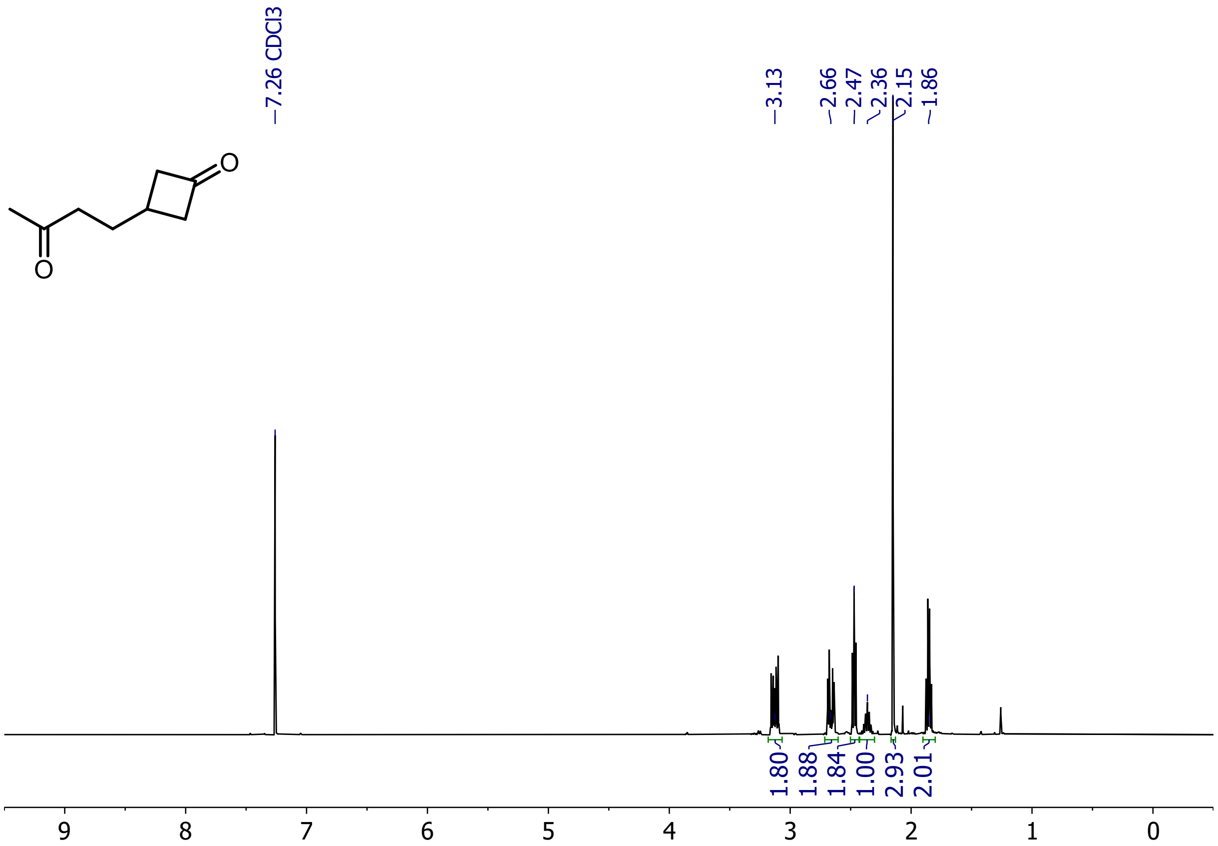

[S24], ^{13}C, $CDCl_3$, 151 MHz

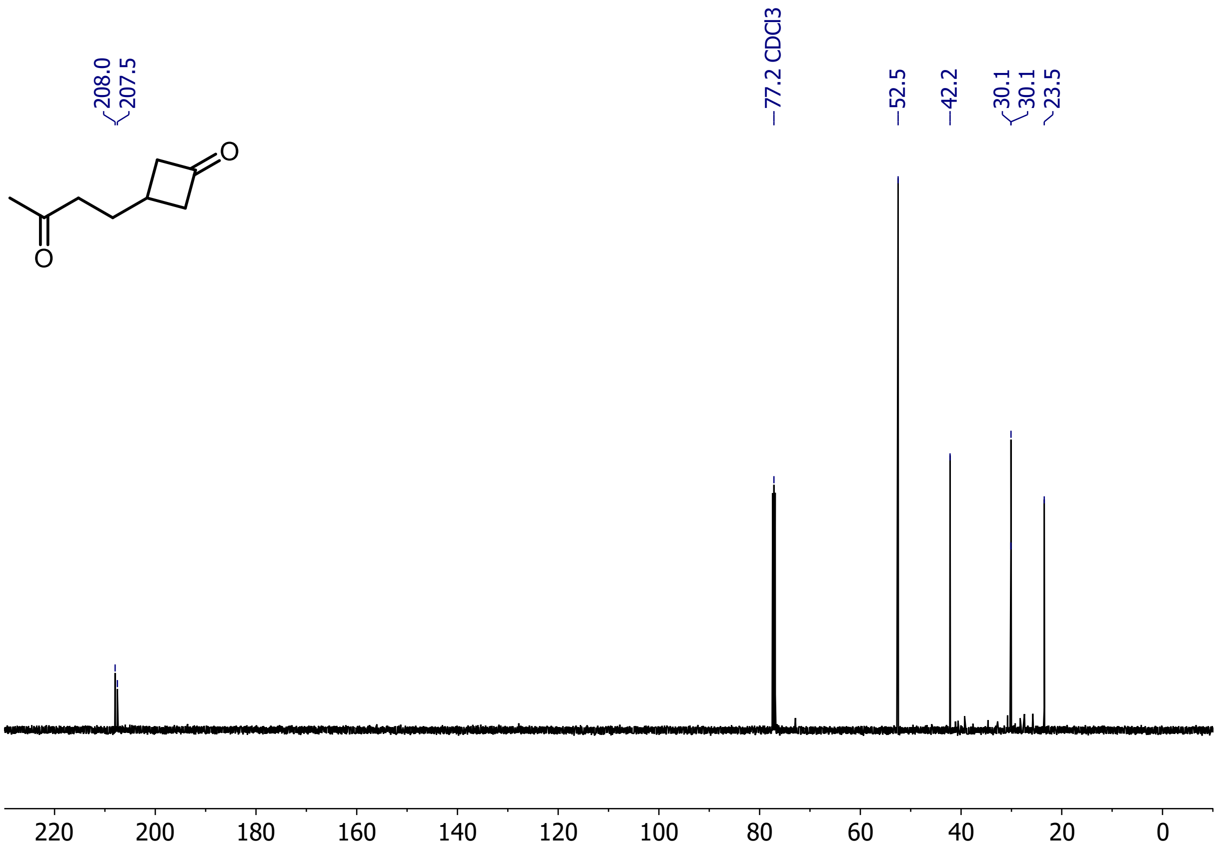

[1a], ^{1}H, $CDCl_3$, 400 MHz

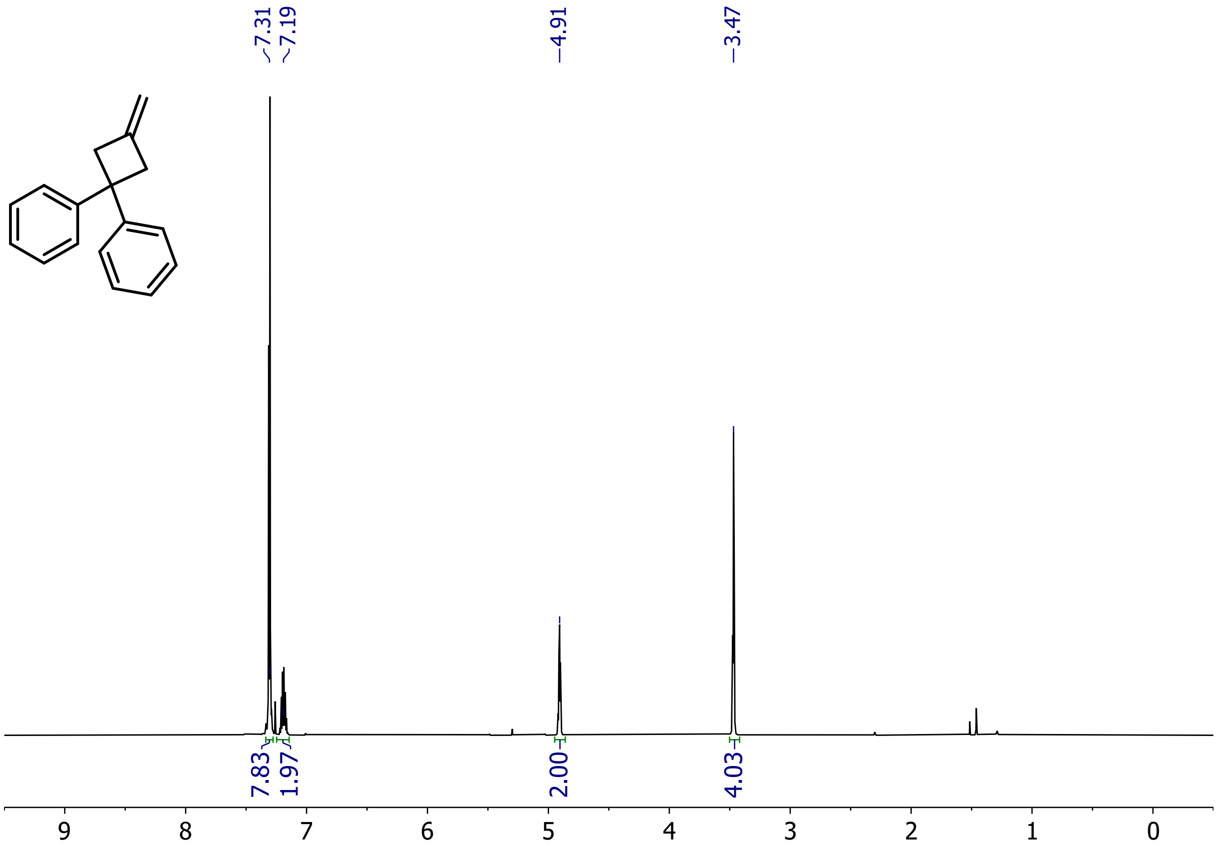

[1a], ^{13}C, $CDCl_3$, 101 MHz

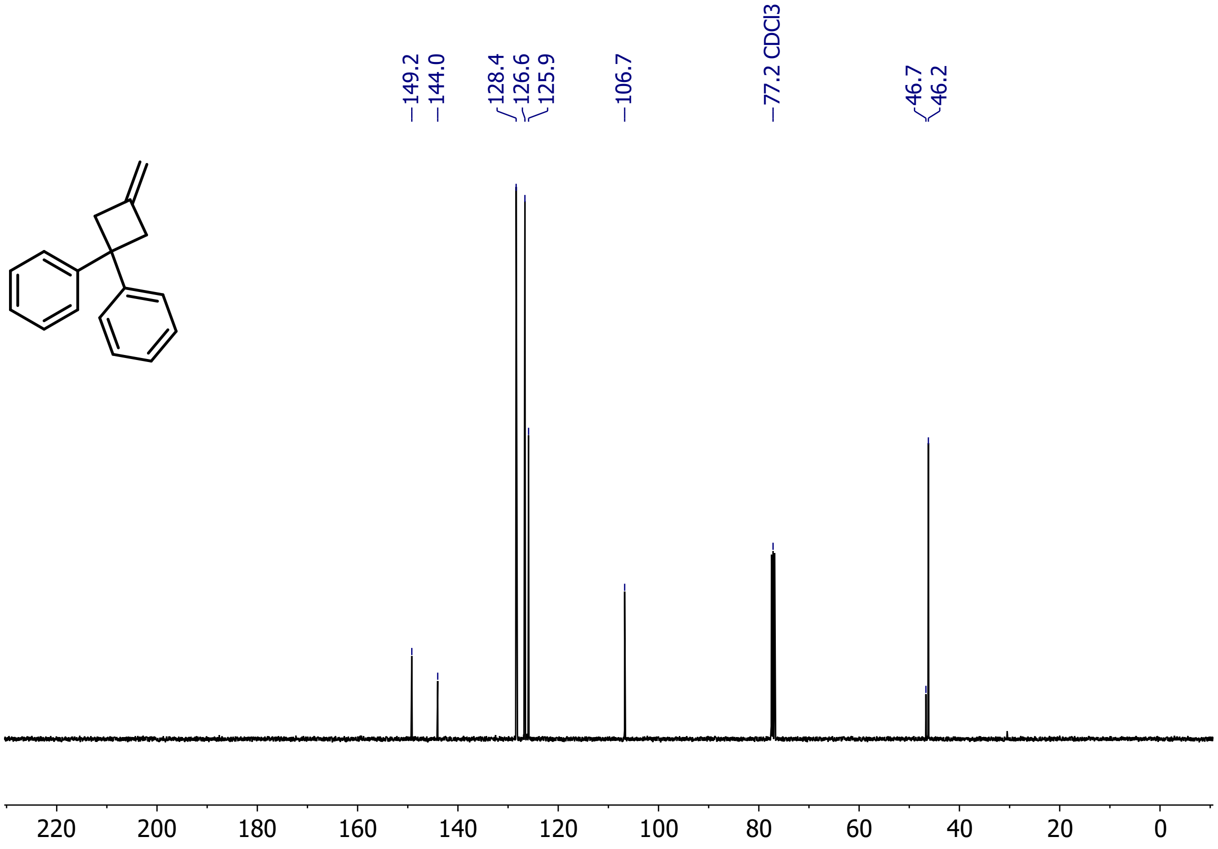

[1b], ^{1}H, $CDCl_3$, 400 MHz

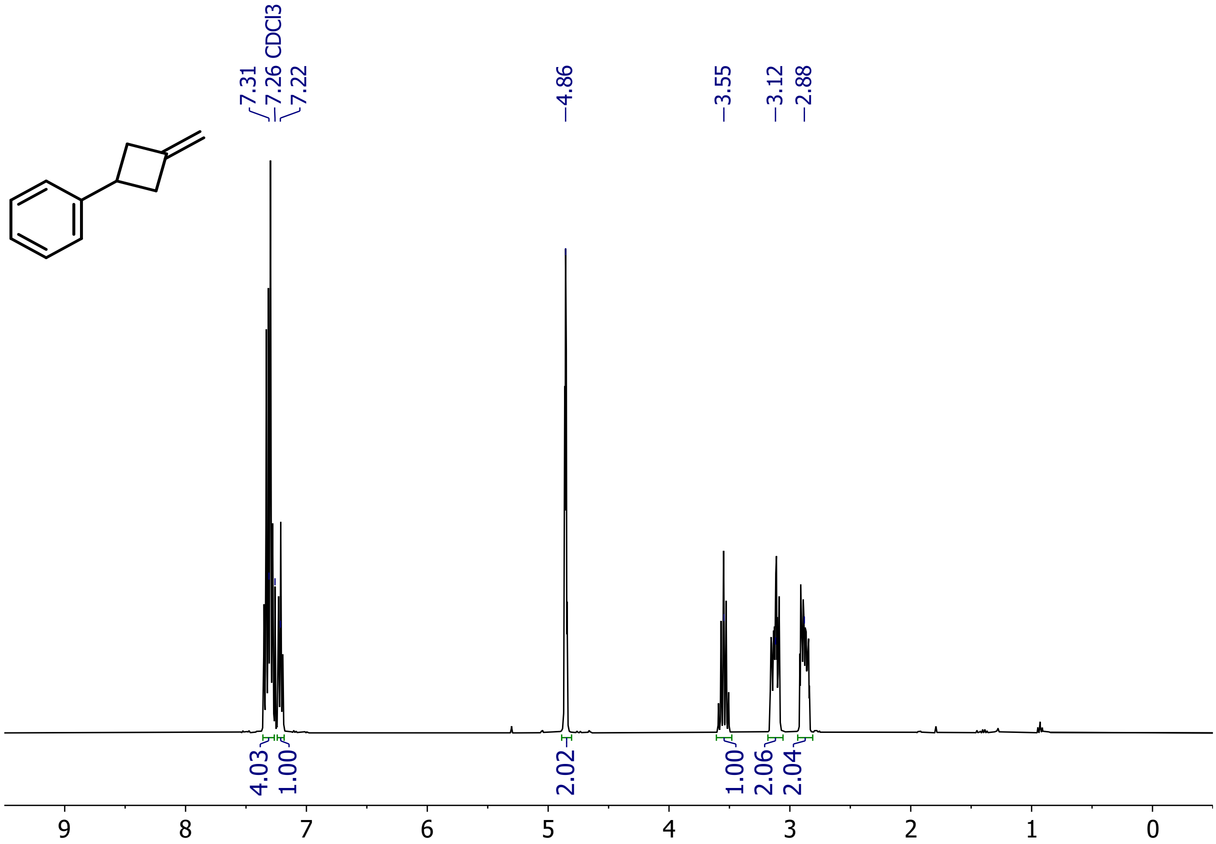

[1b], ^{13}C, $CDCl_3$, 101 MHz

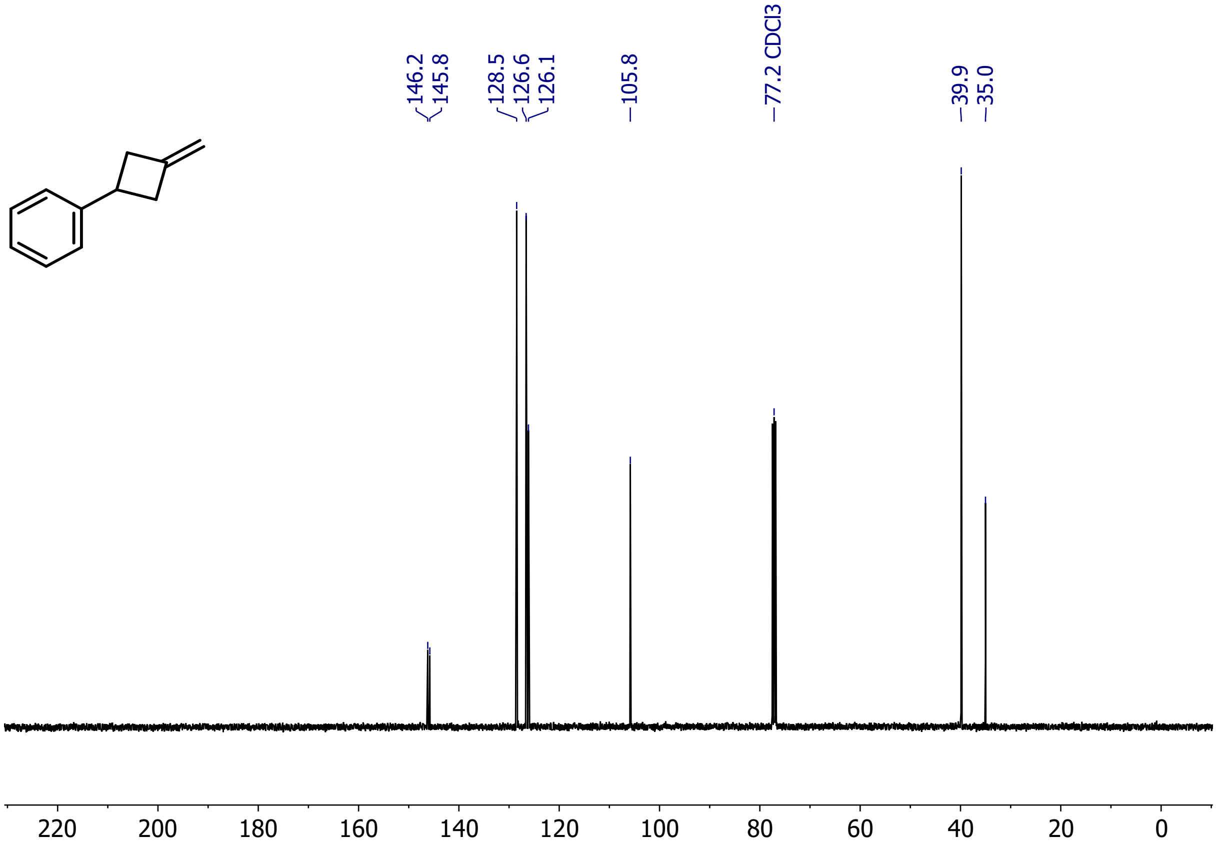

[1c], ^{1}H, $CDCl_3$, 599 MHz

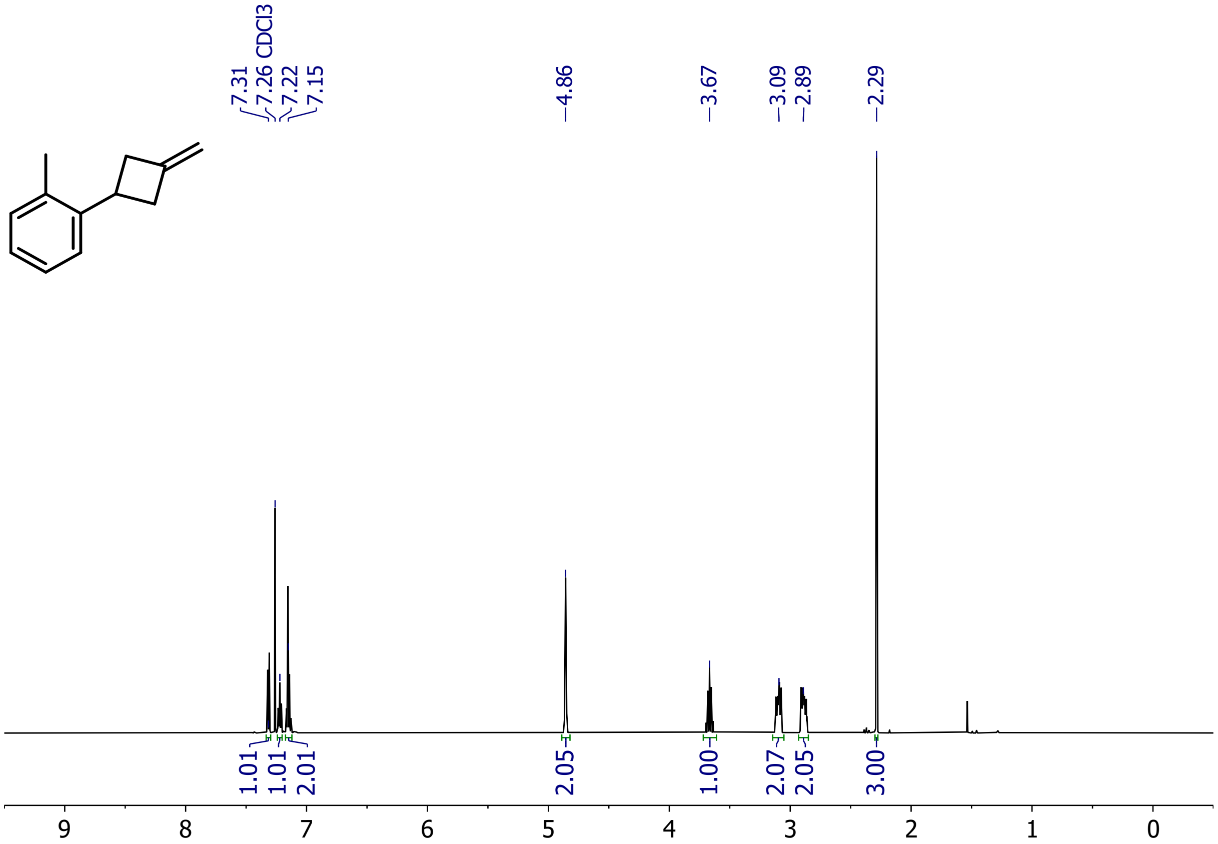

[1c], ^{13}C, $CDCl_3$, 151 MHz

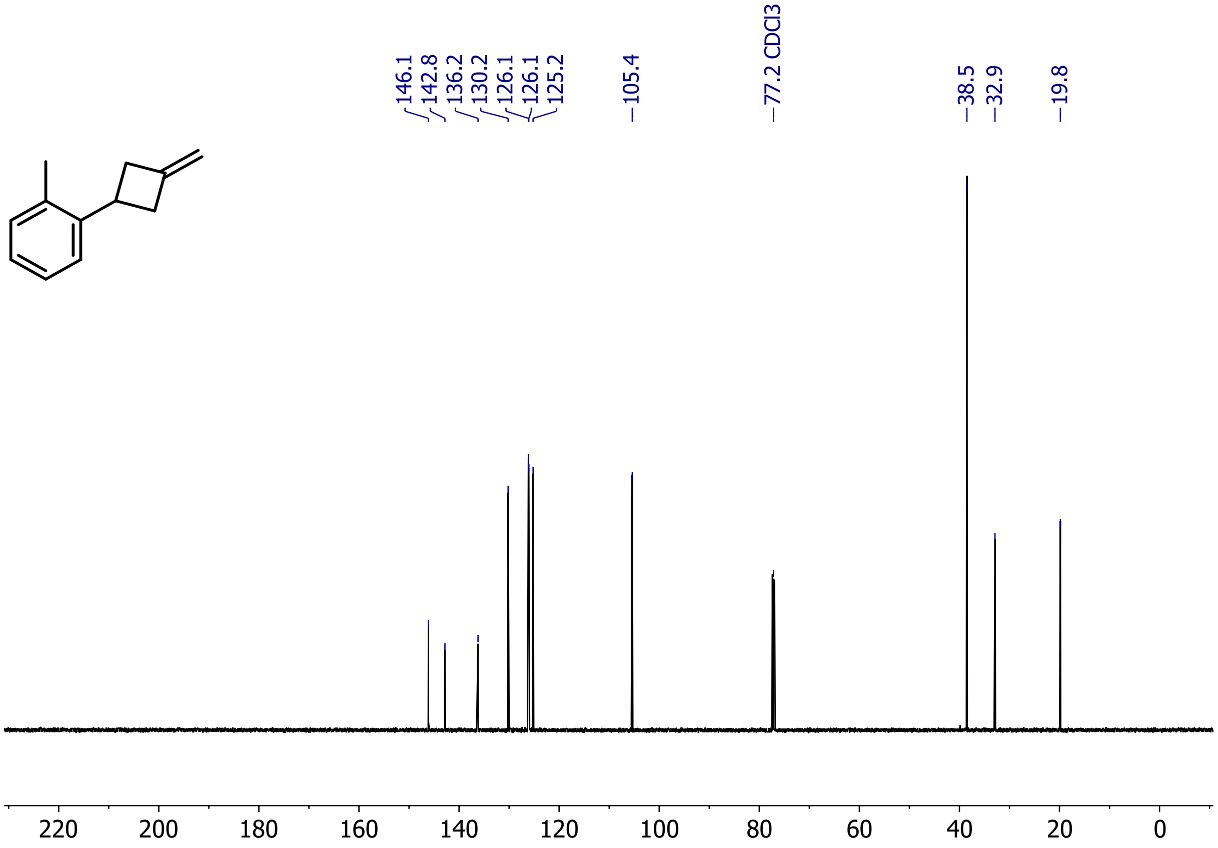

[1d], ^{1}H, $CDCl_3$, 599 MHz

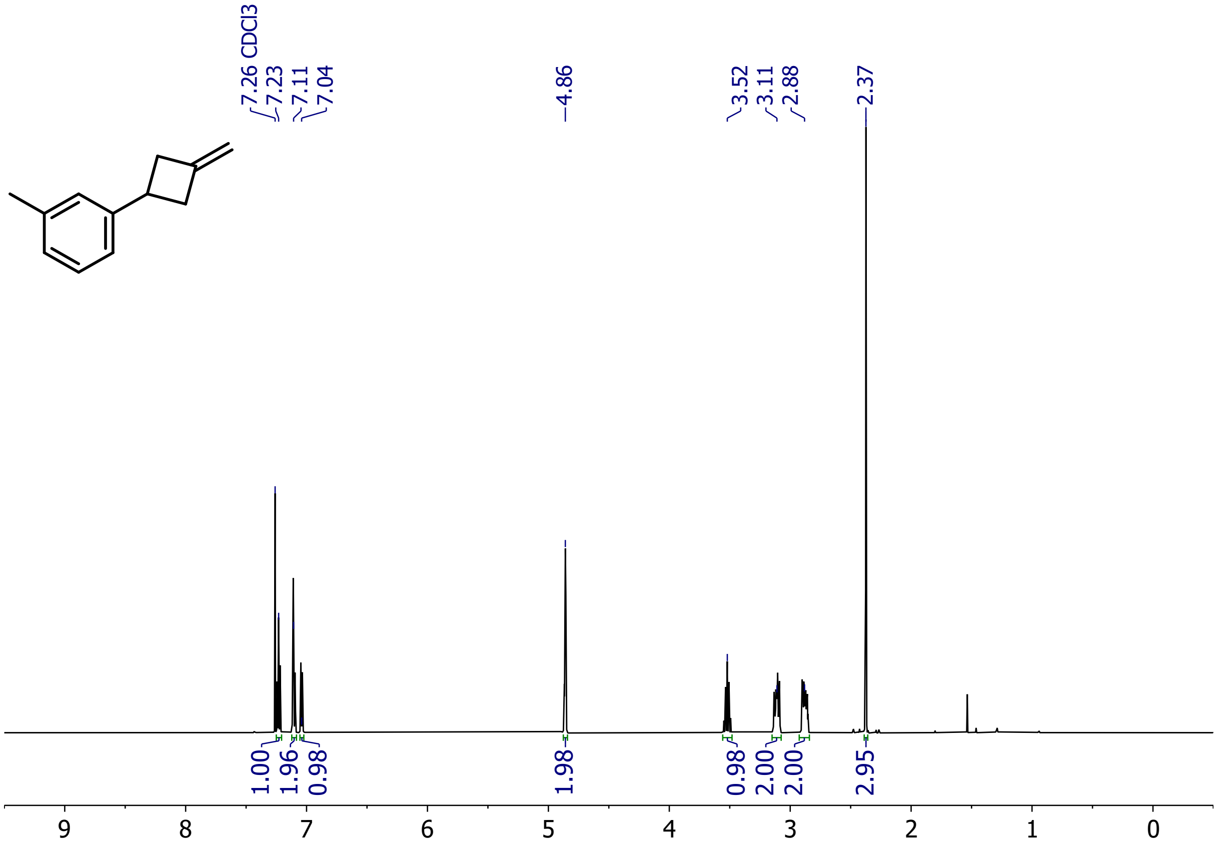

[1d], ^{13}C, $CDCl_3$, 151 MHz

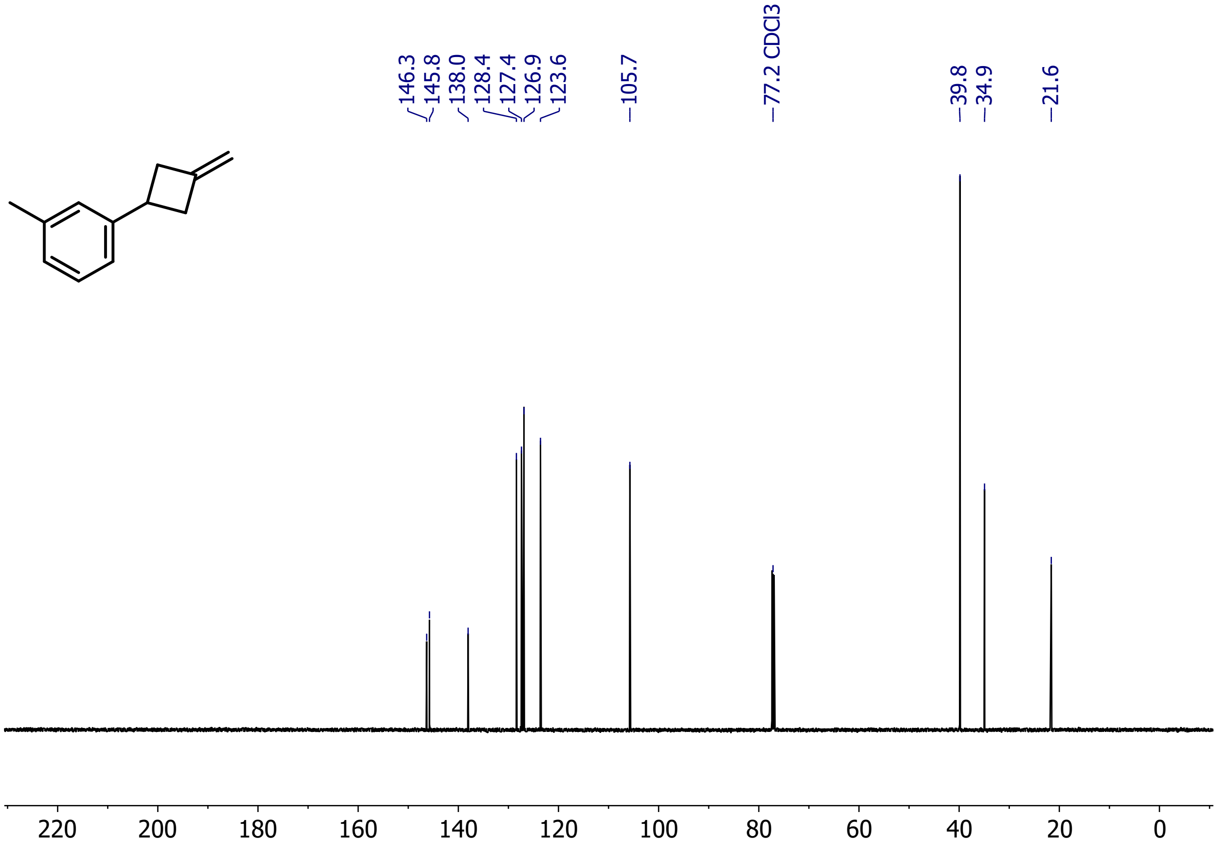

[1e], ^1H, $CDCl_3$, 599 MHz

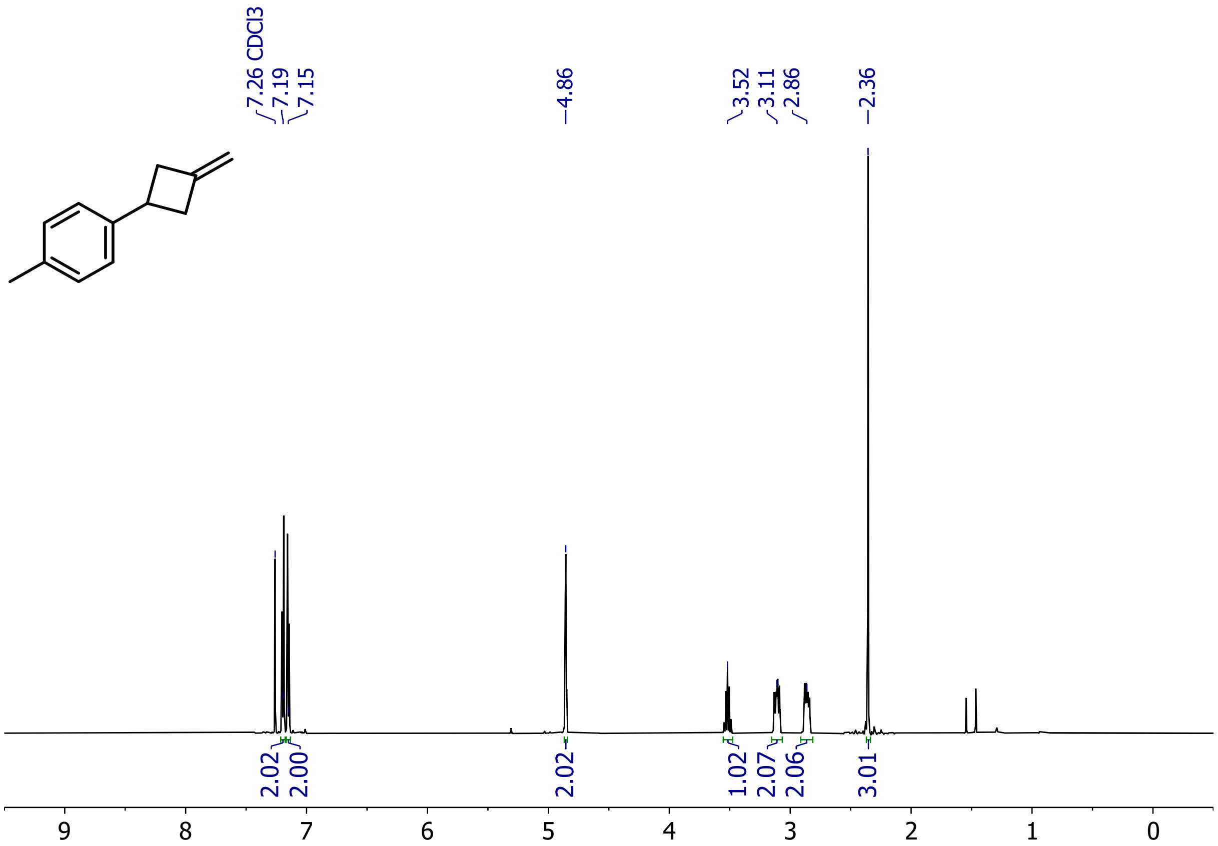

[1e], ^{13}C, $CDCl_3$, 151 MHz

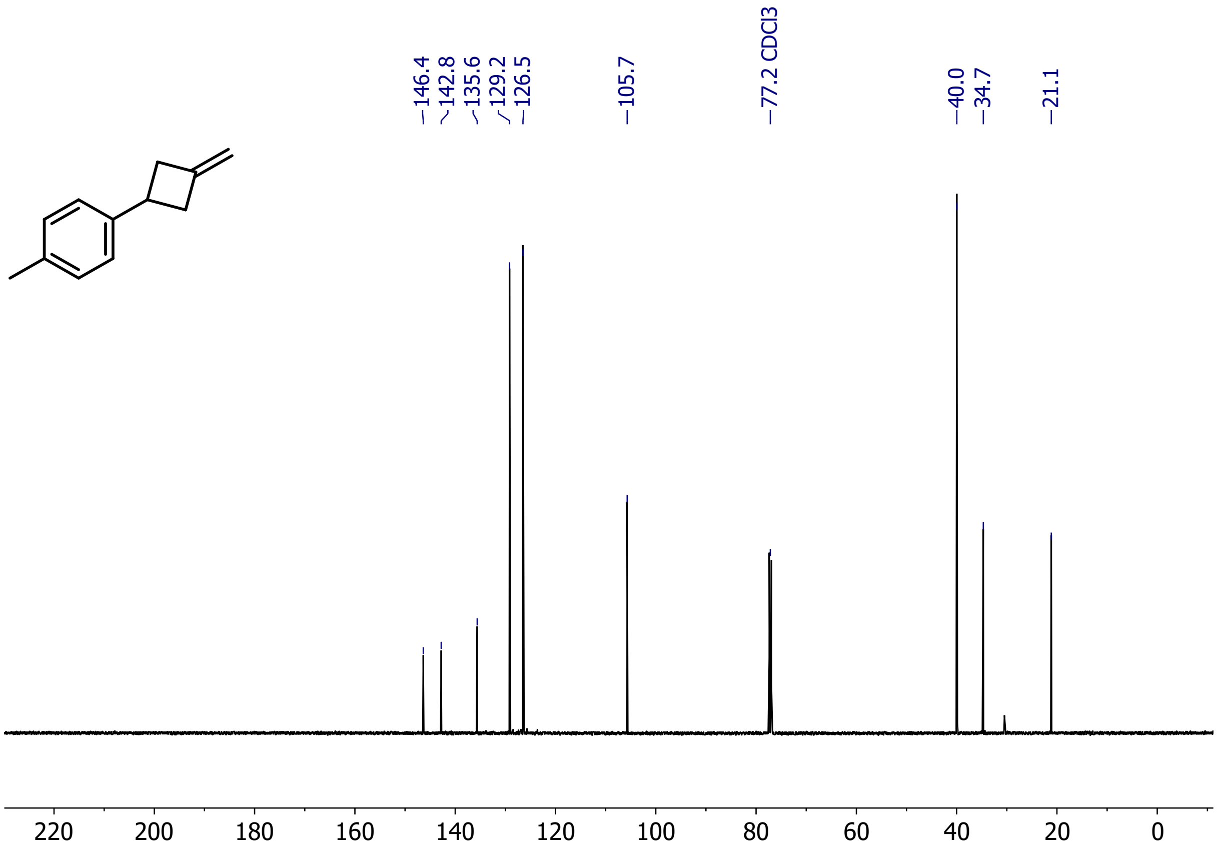

[1f], ^{1}H, $CDCl_3$, 599 MHz

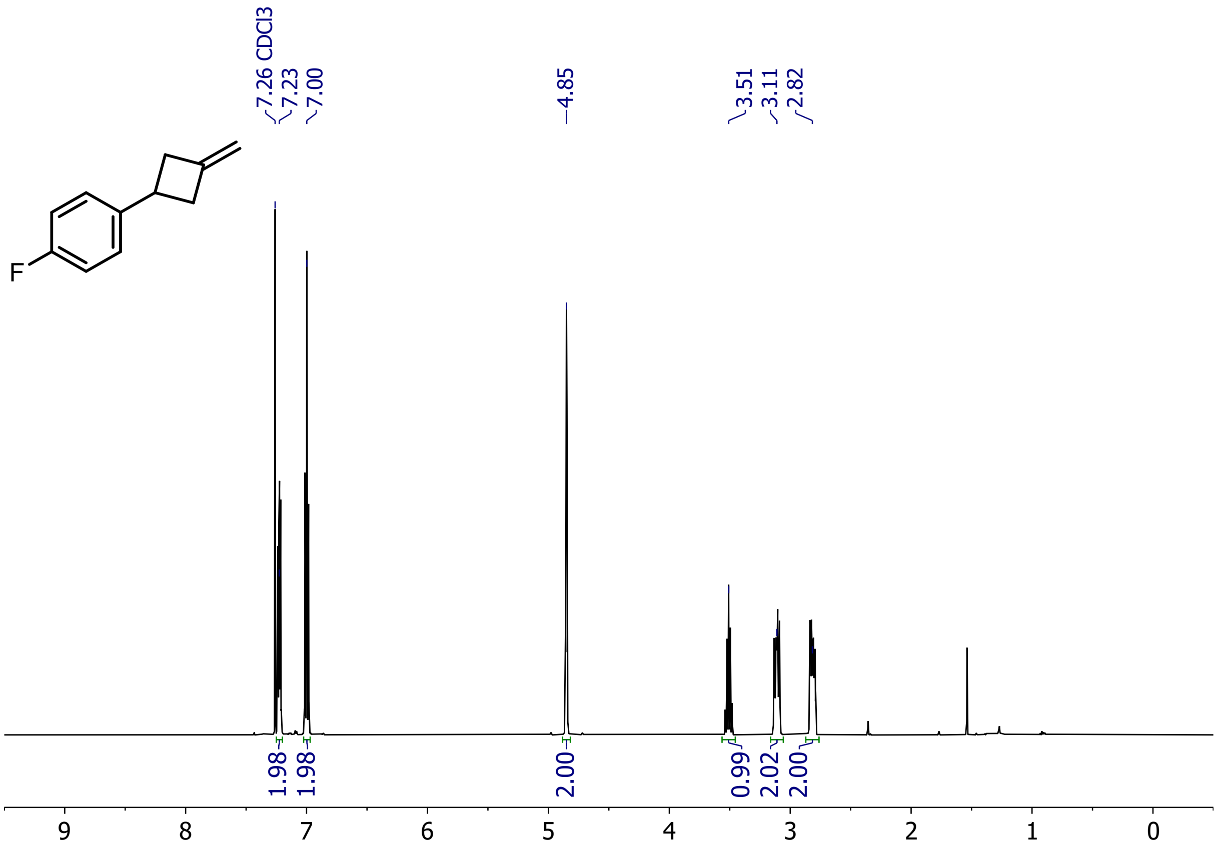

[1f], ^{13}C, $CDCl_3$, 151 MHz

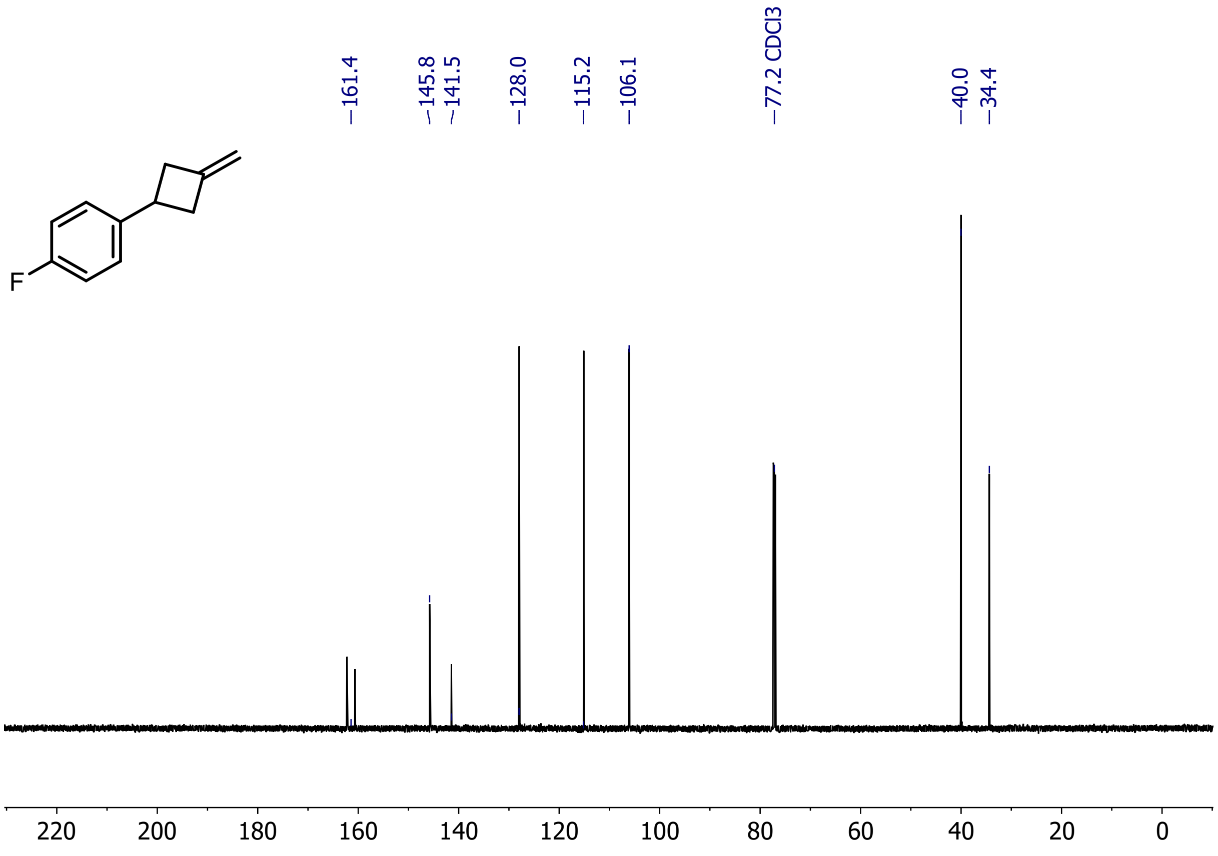

[1f], ^{19}F, $CDCl_3$, 564 MHz

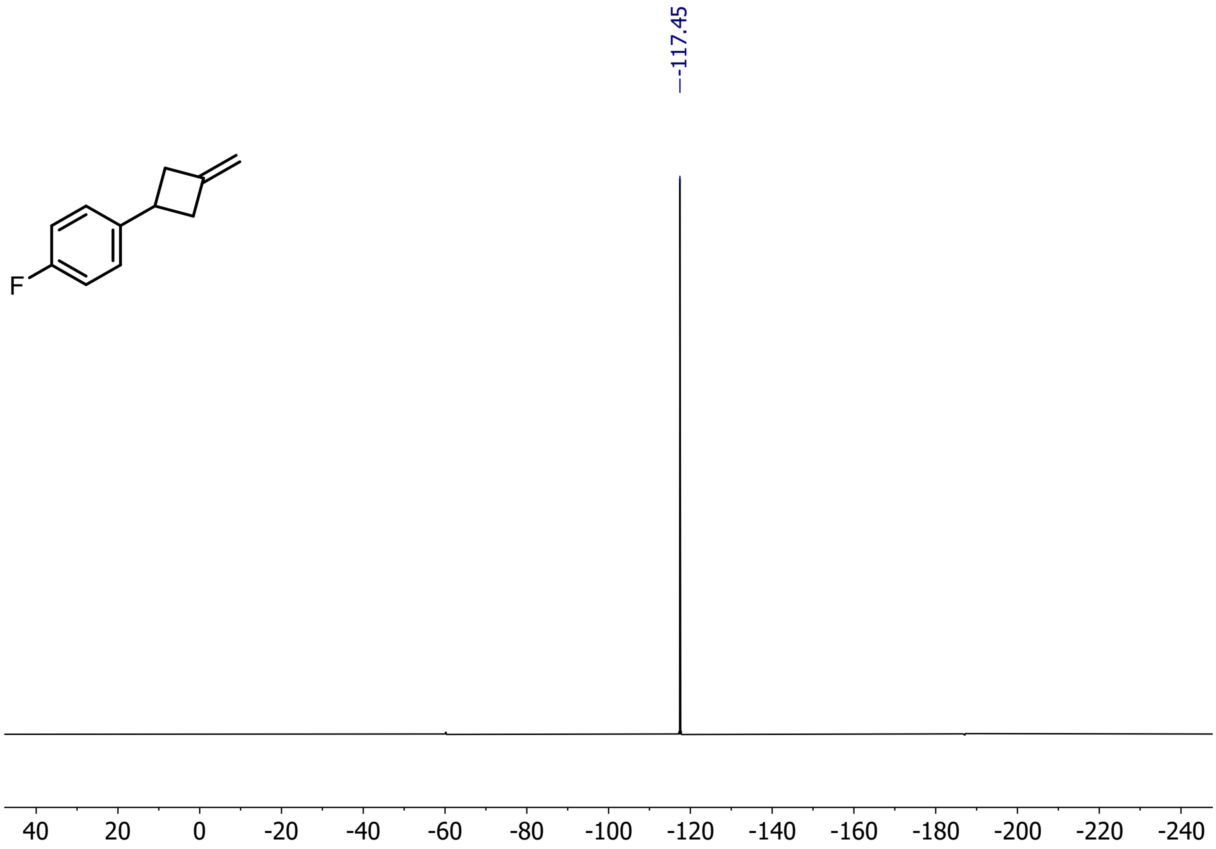

[1g], ^{1}H, $CDCl_3$, 599 MHz

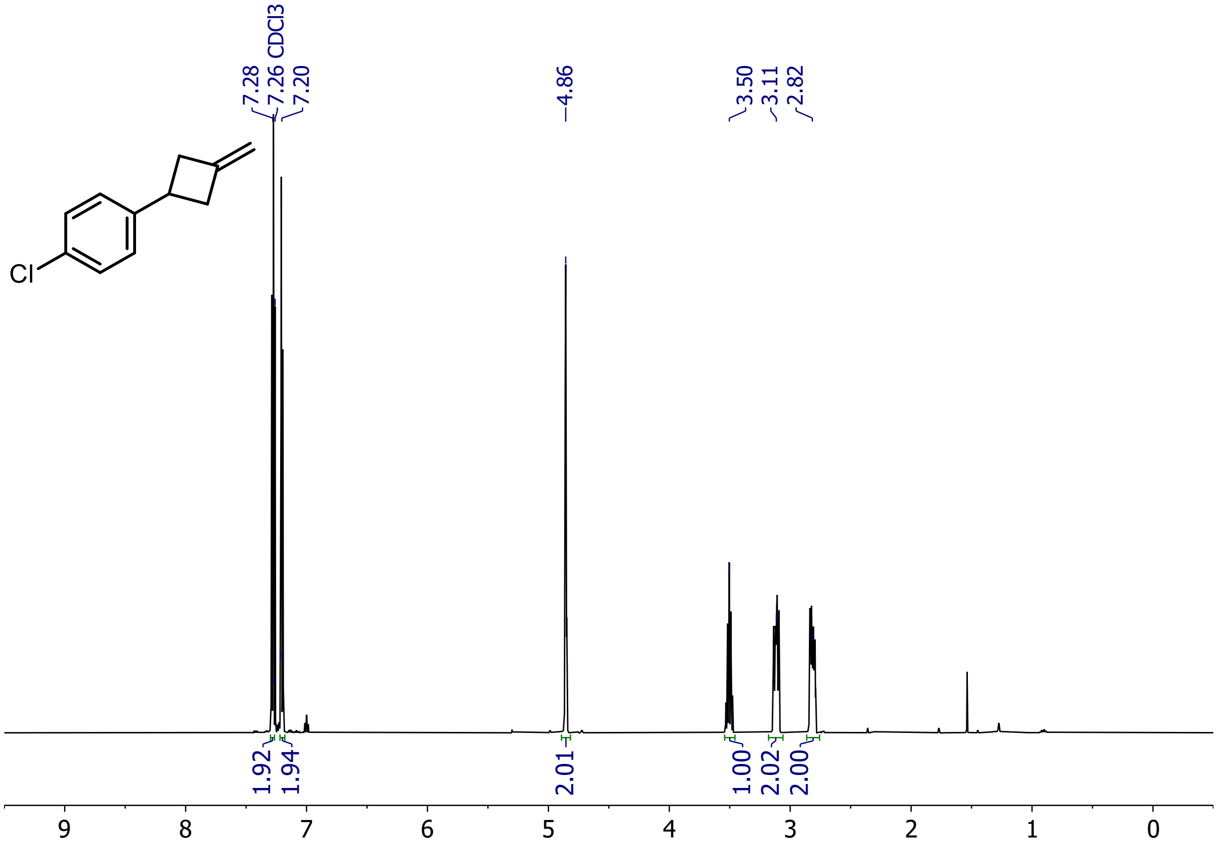

[1g], ^{13}C, $CDCl_3$, 151 MHz

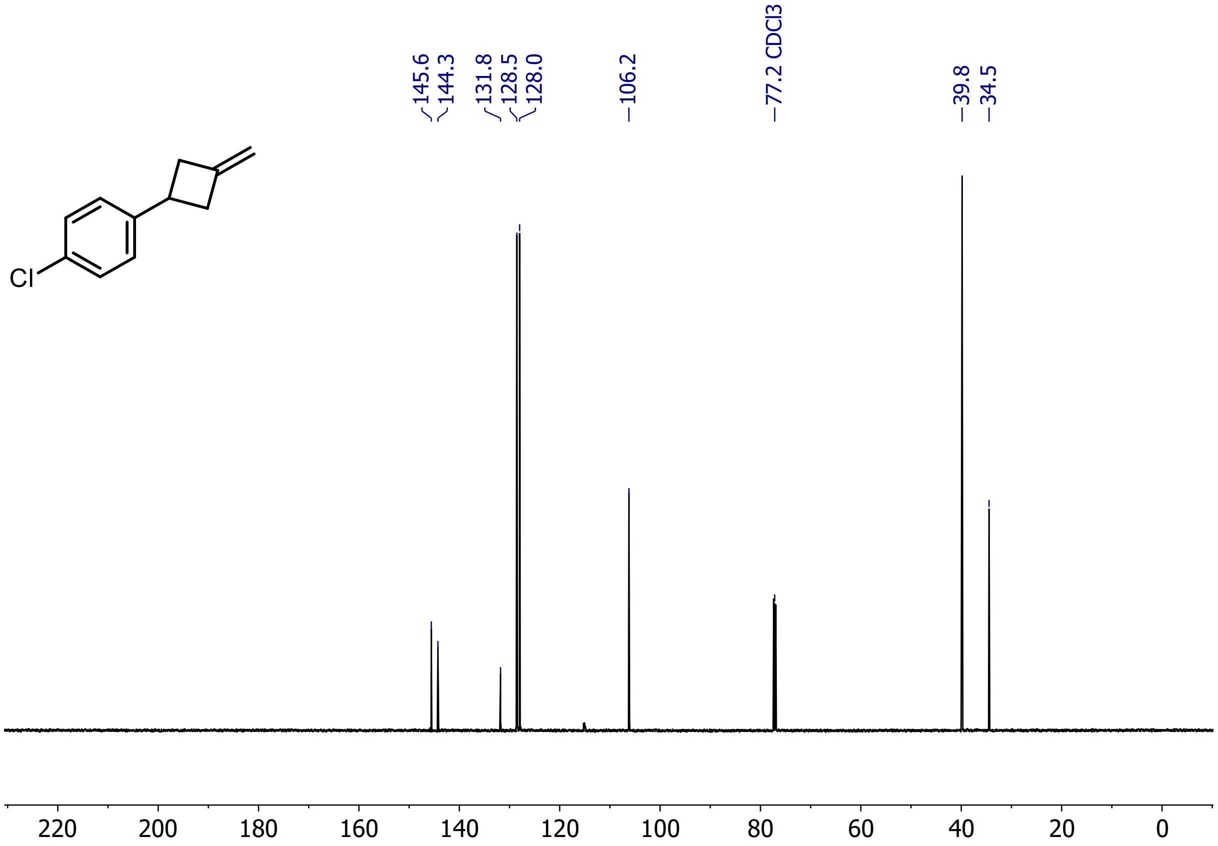

[1h], ^{1}H, $CDCl_3$, 599 MHz

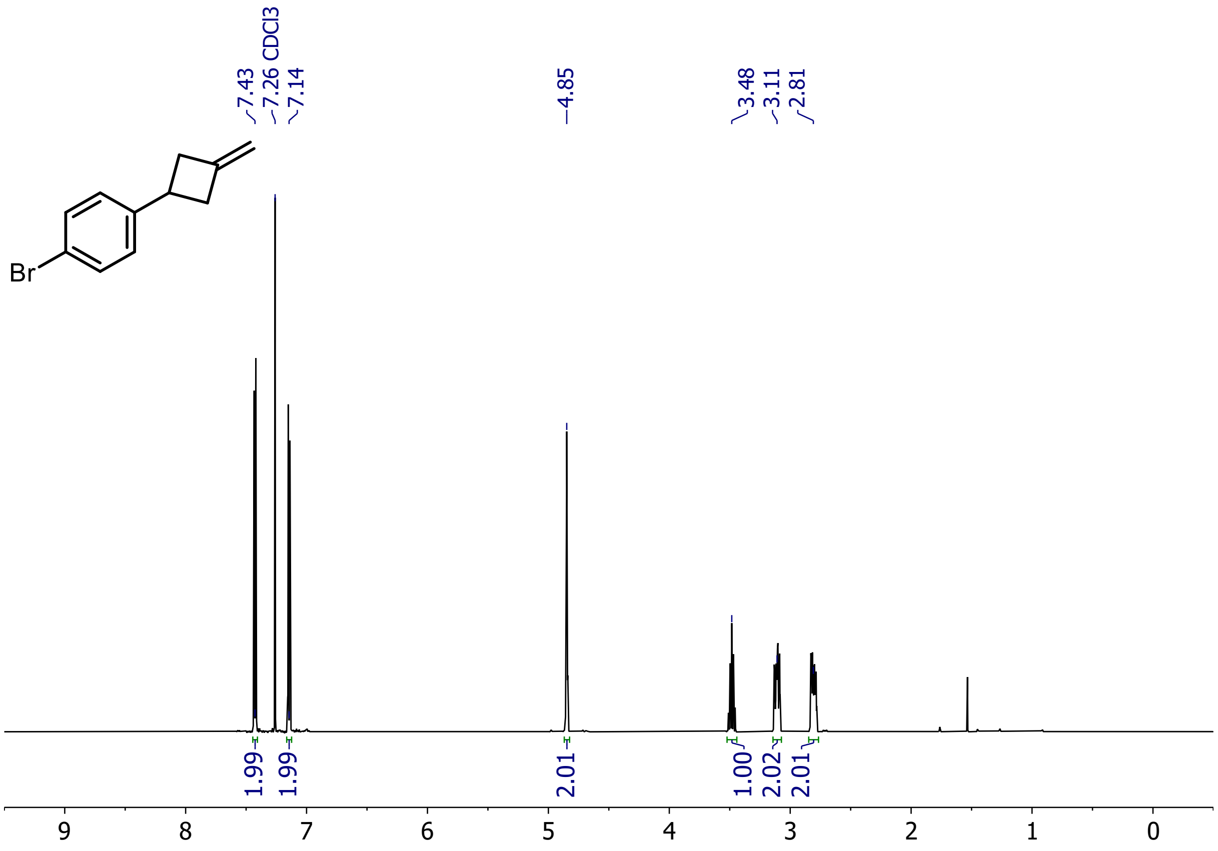

[1h], ^{13}C, $CDCl_3$, 151 MHz

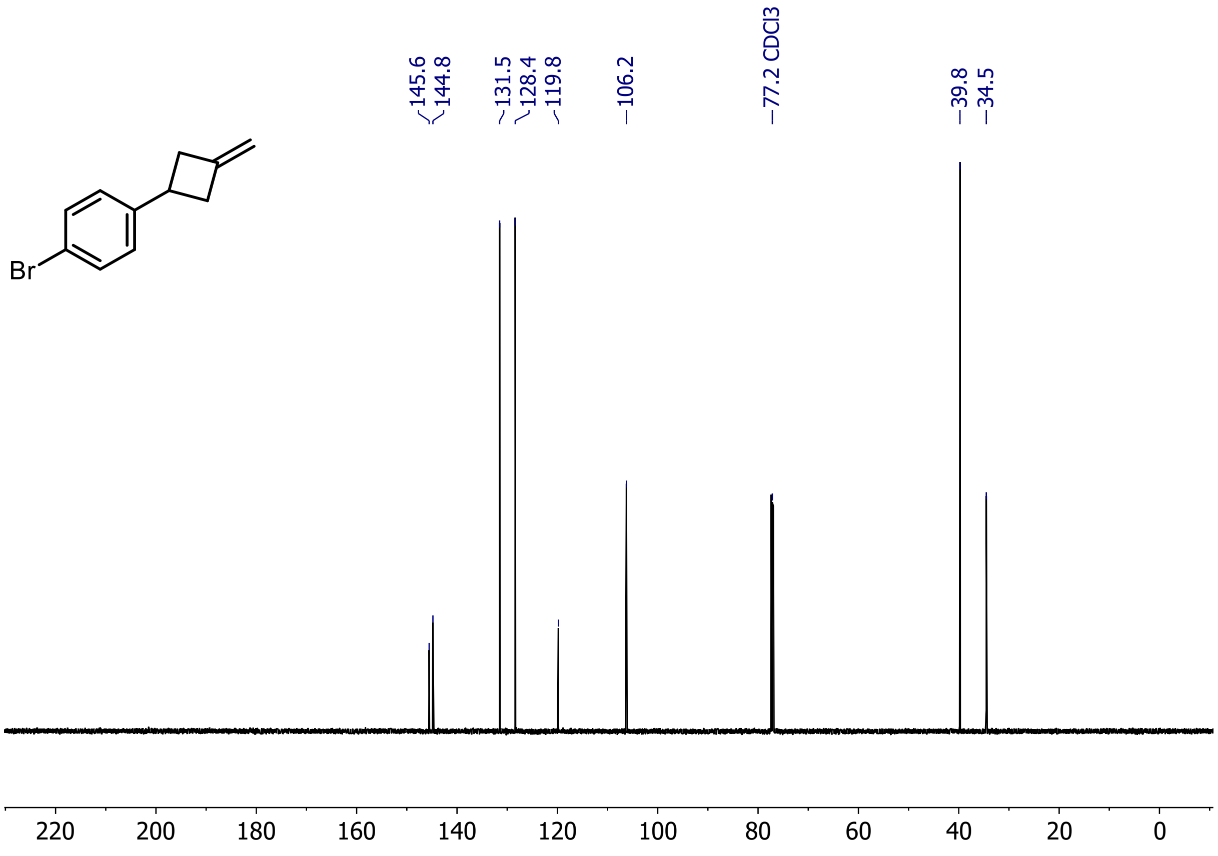

[1i], ^{1}H, $CDCl_3$, 599 MHz

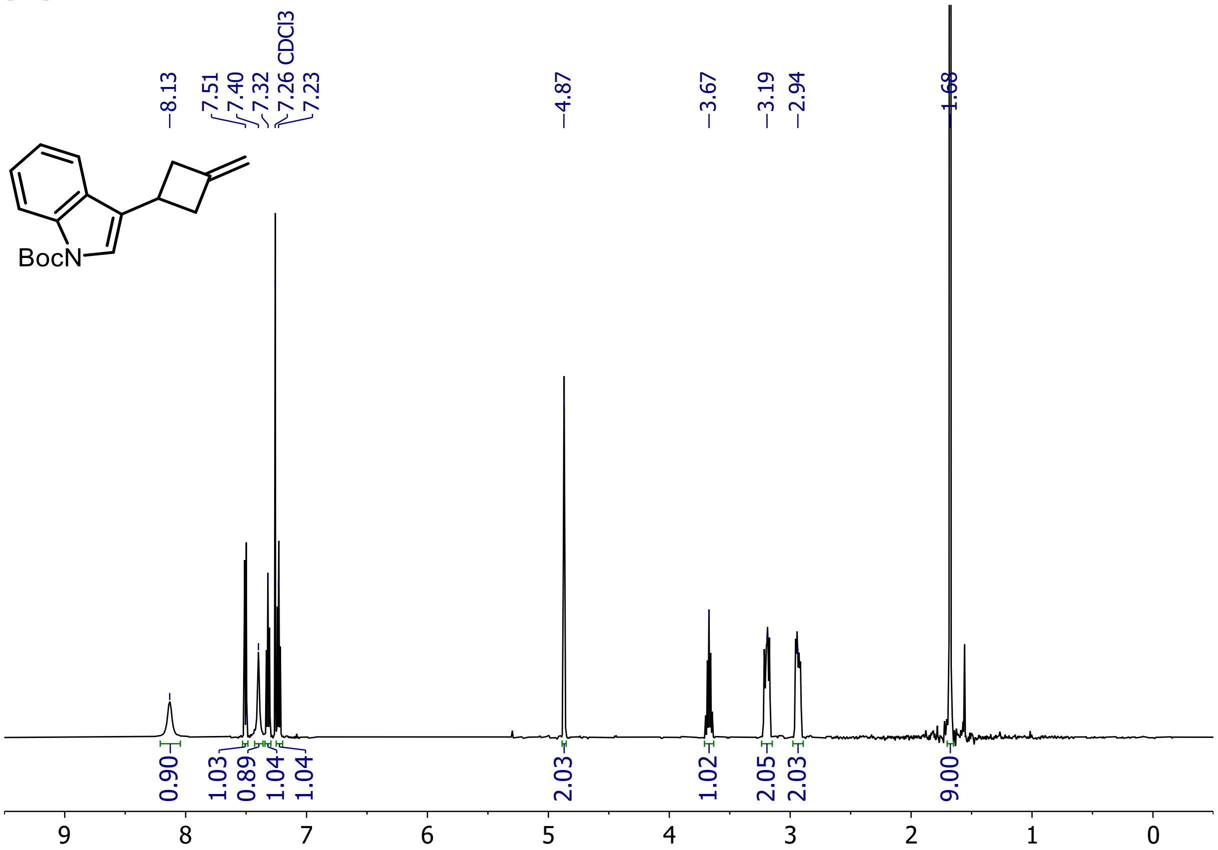

[1i], ^{13}C, $CDCl_3$, 151 MHz

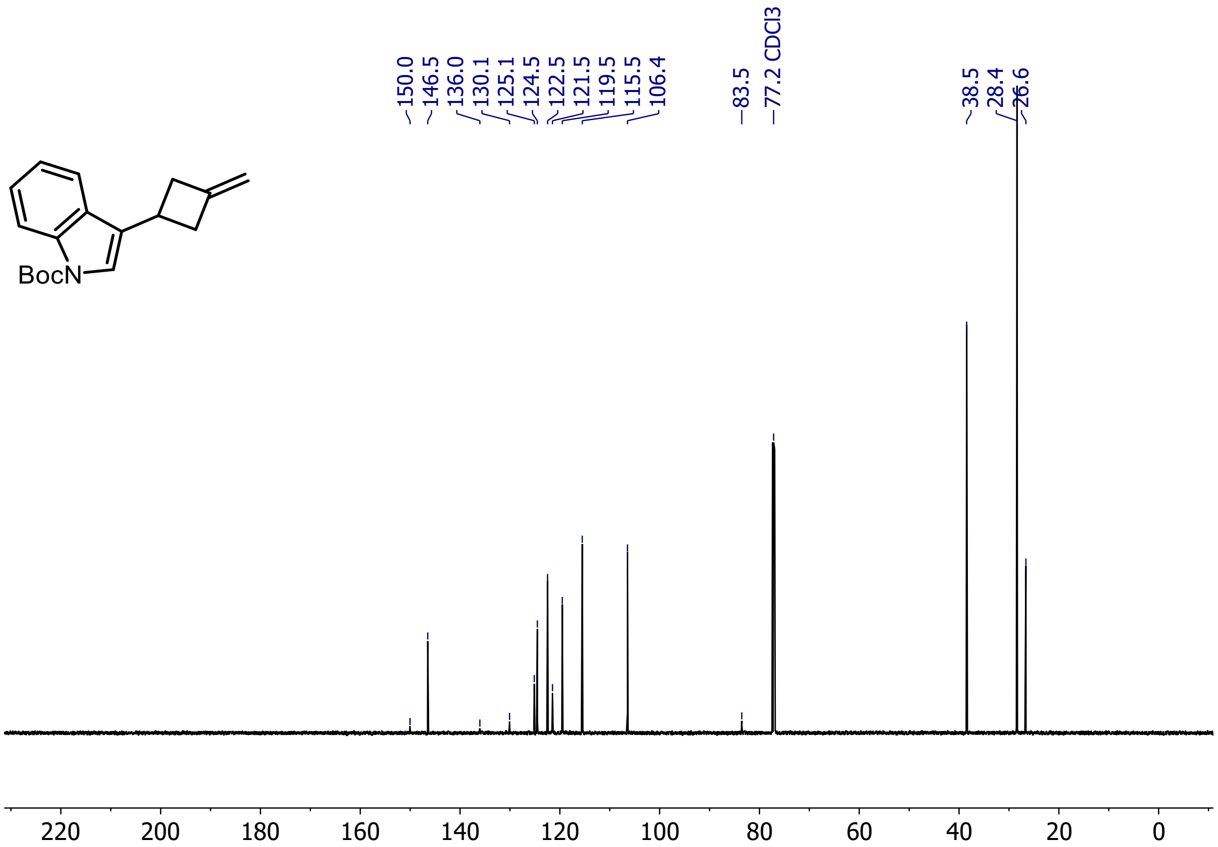

[1j], ^{1}H, $CDCl_3$, 400 MHz

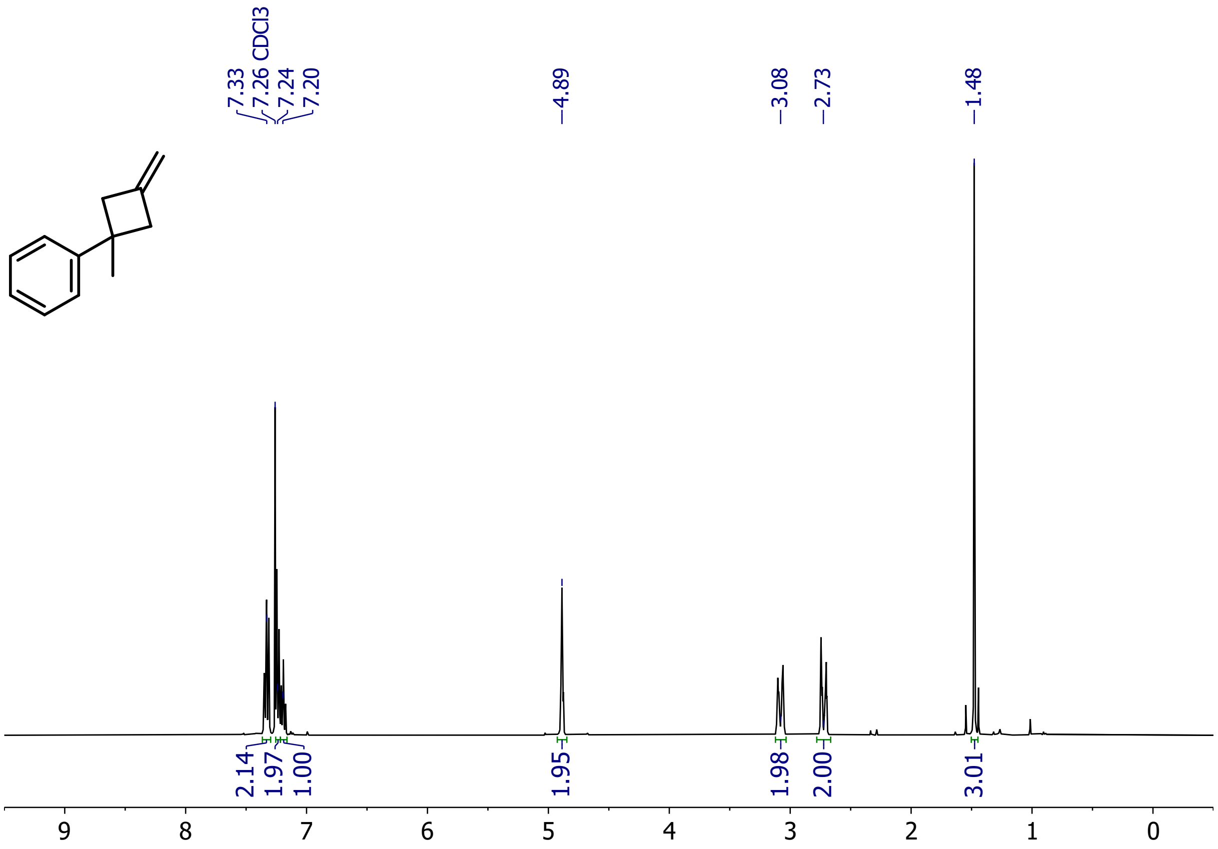

[1j], ^{13}C, $CDCl_3$, 101 MHz

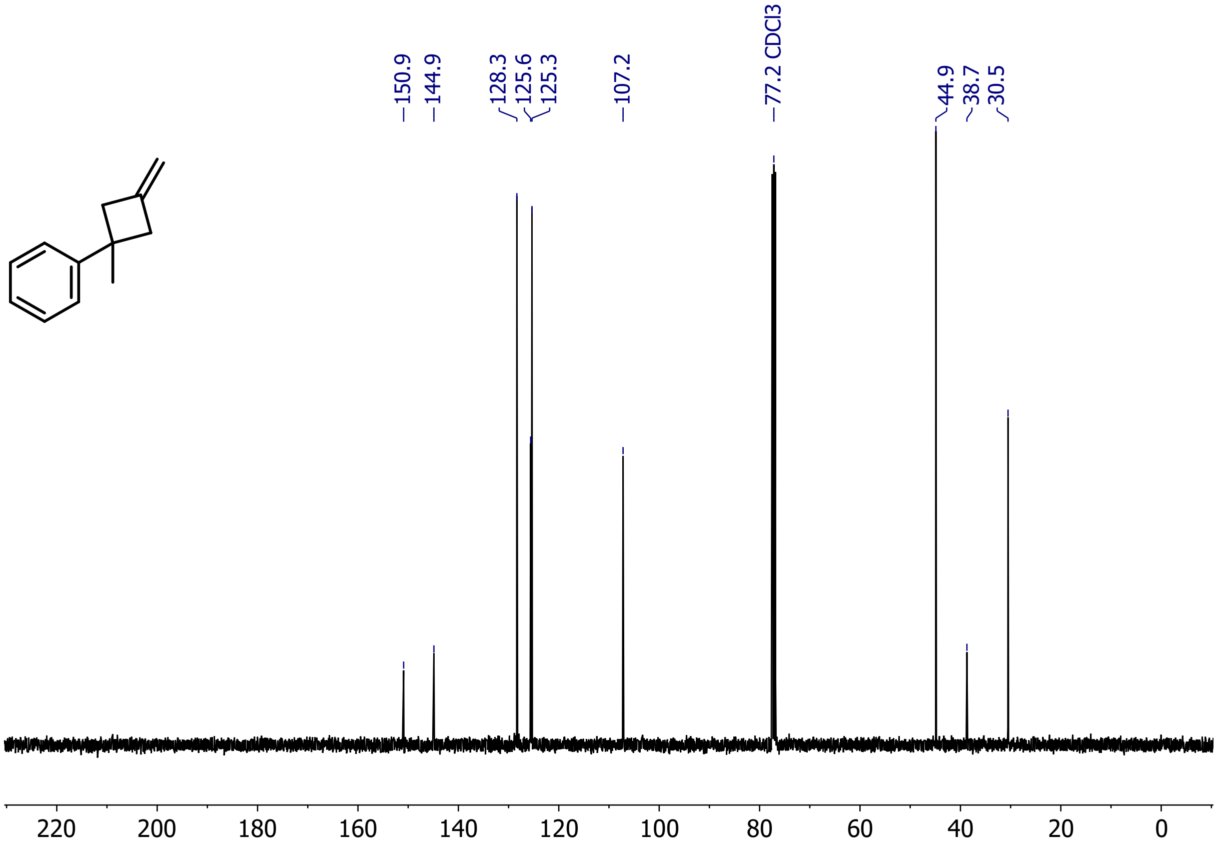

[1k], ^{1}H, $CDCl_3$, 599 MHz

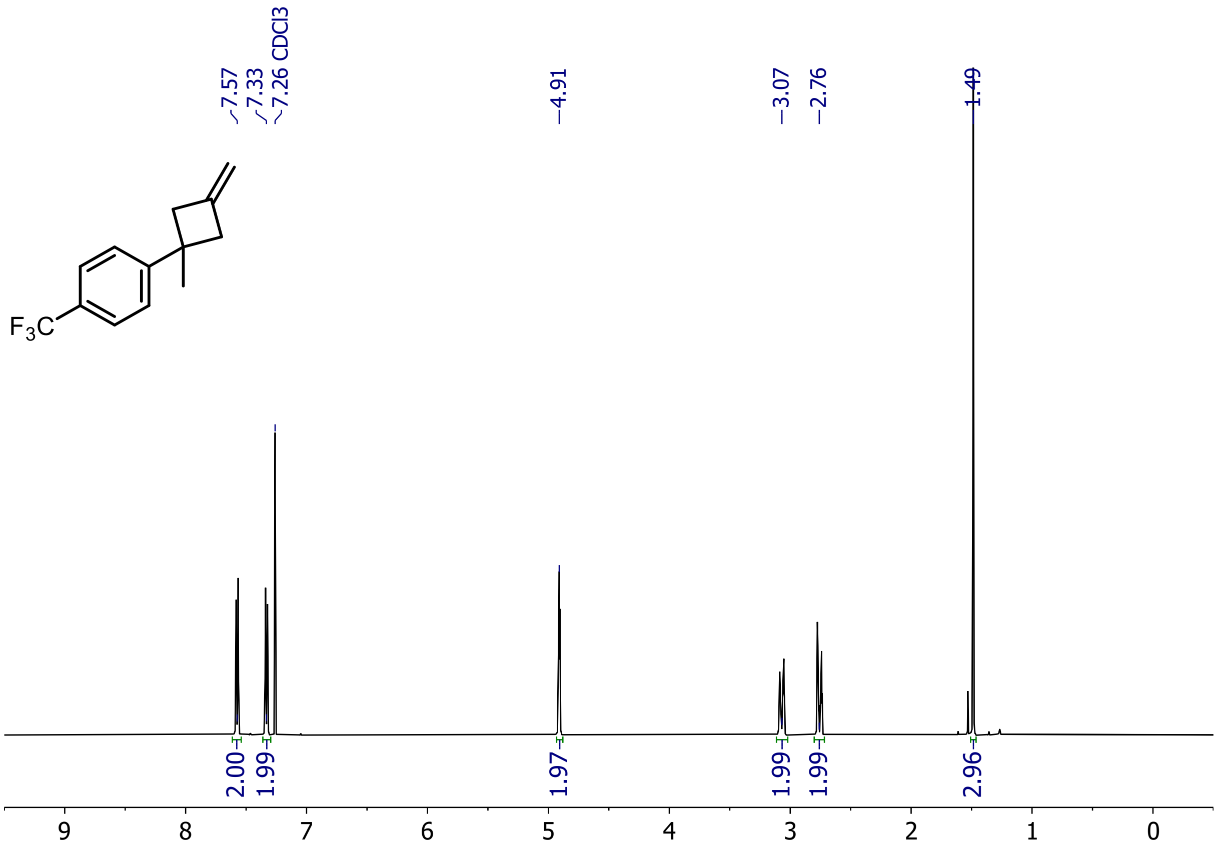

[1k], ^{13}C, $CDCl_3$, 151 MHz

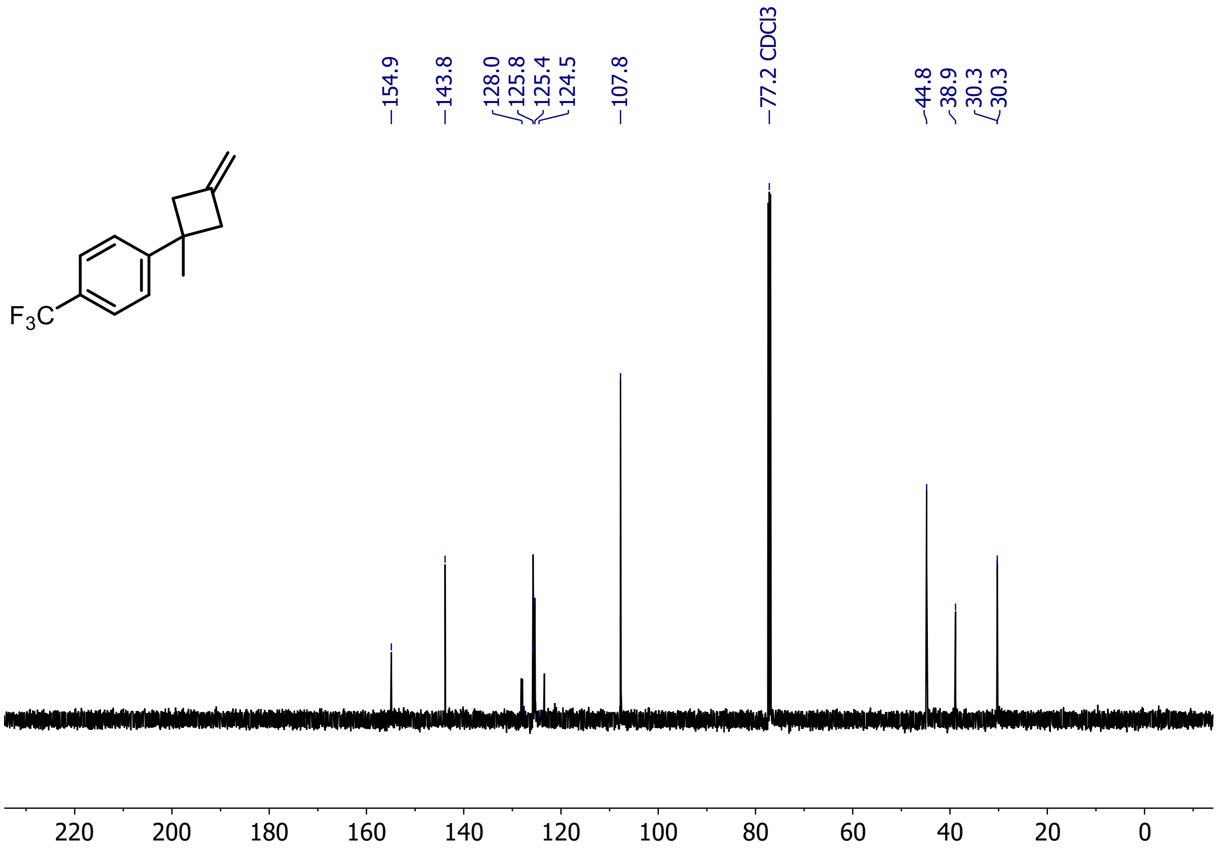

[1k], ^{19}F, $CDCl_3$, 377 MHz

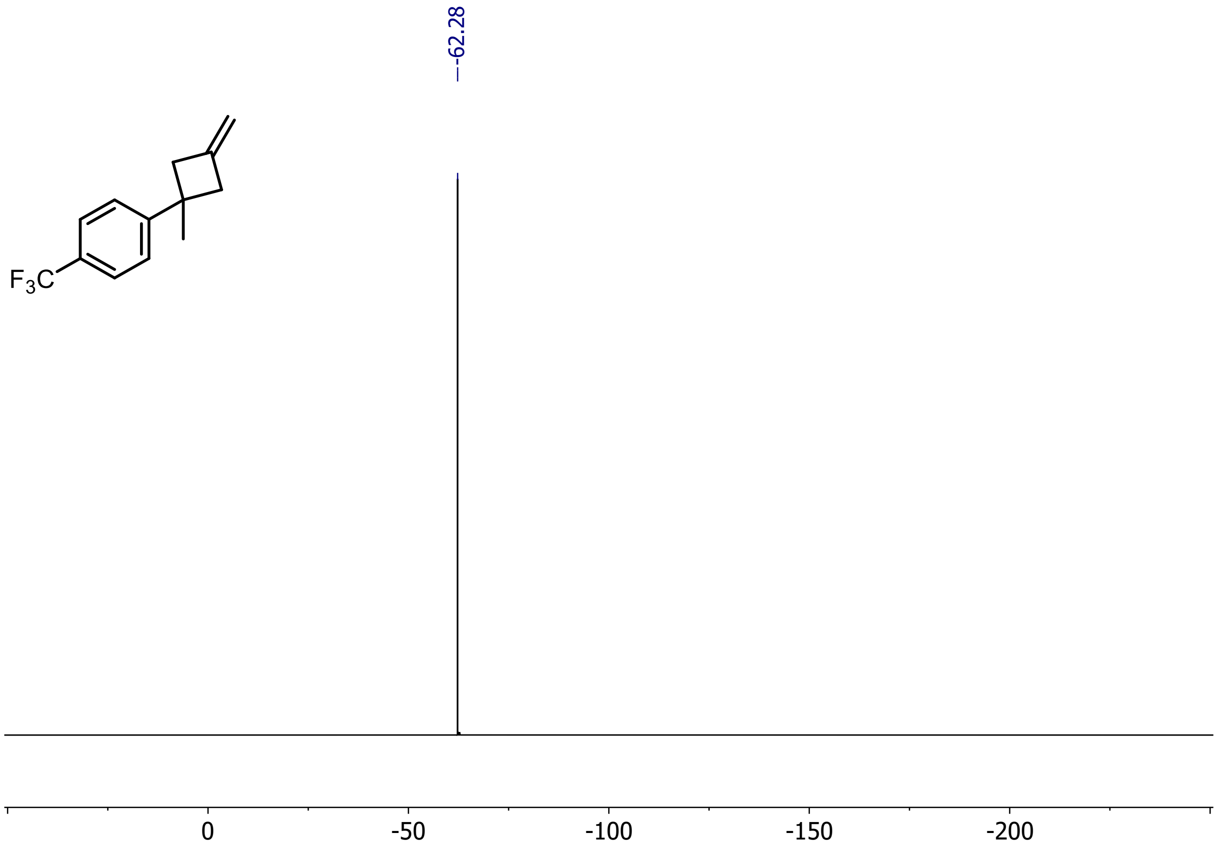

[1l], ^{1}H, $CDCl_3$, 599 MHz

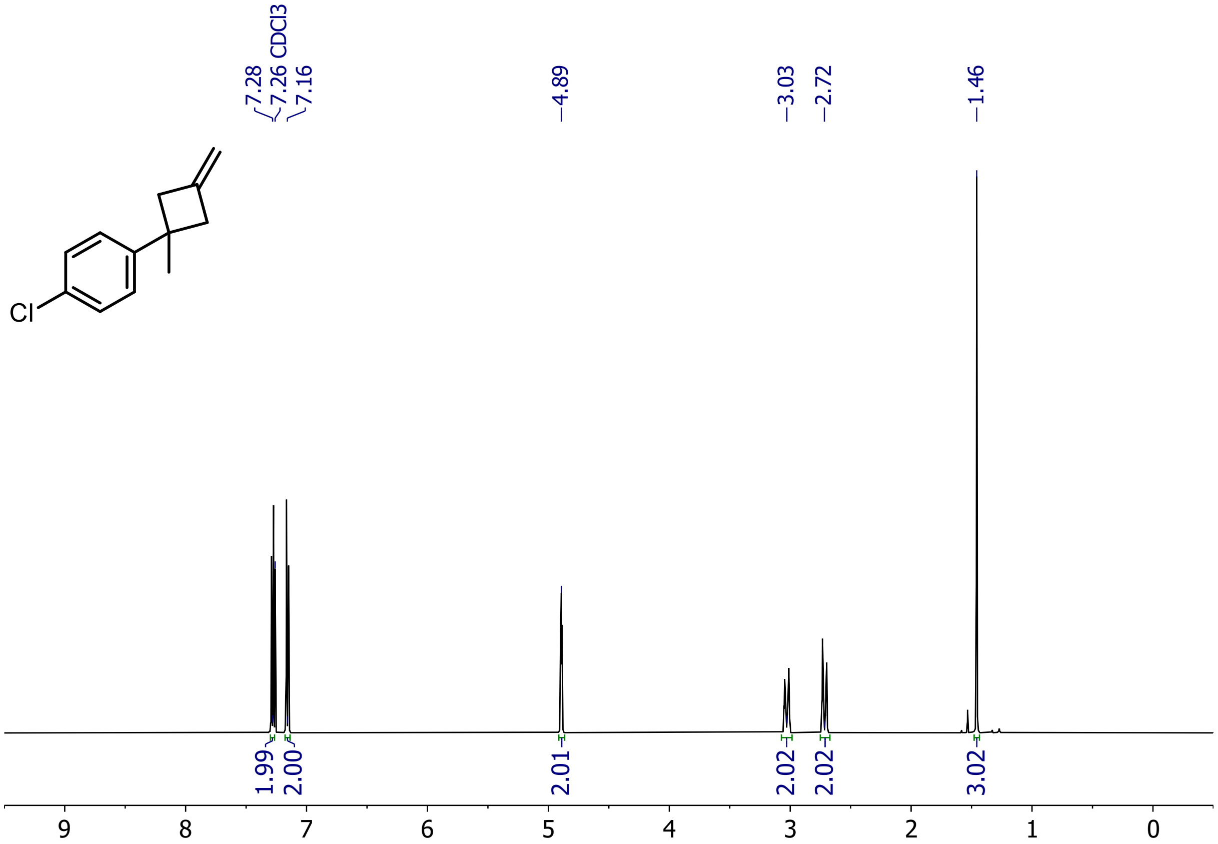

[1l], ^{13}C, $CDCl_3$, 151 MHz

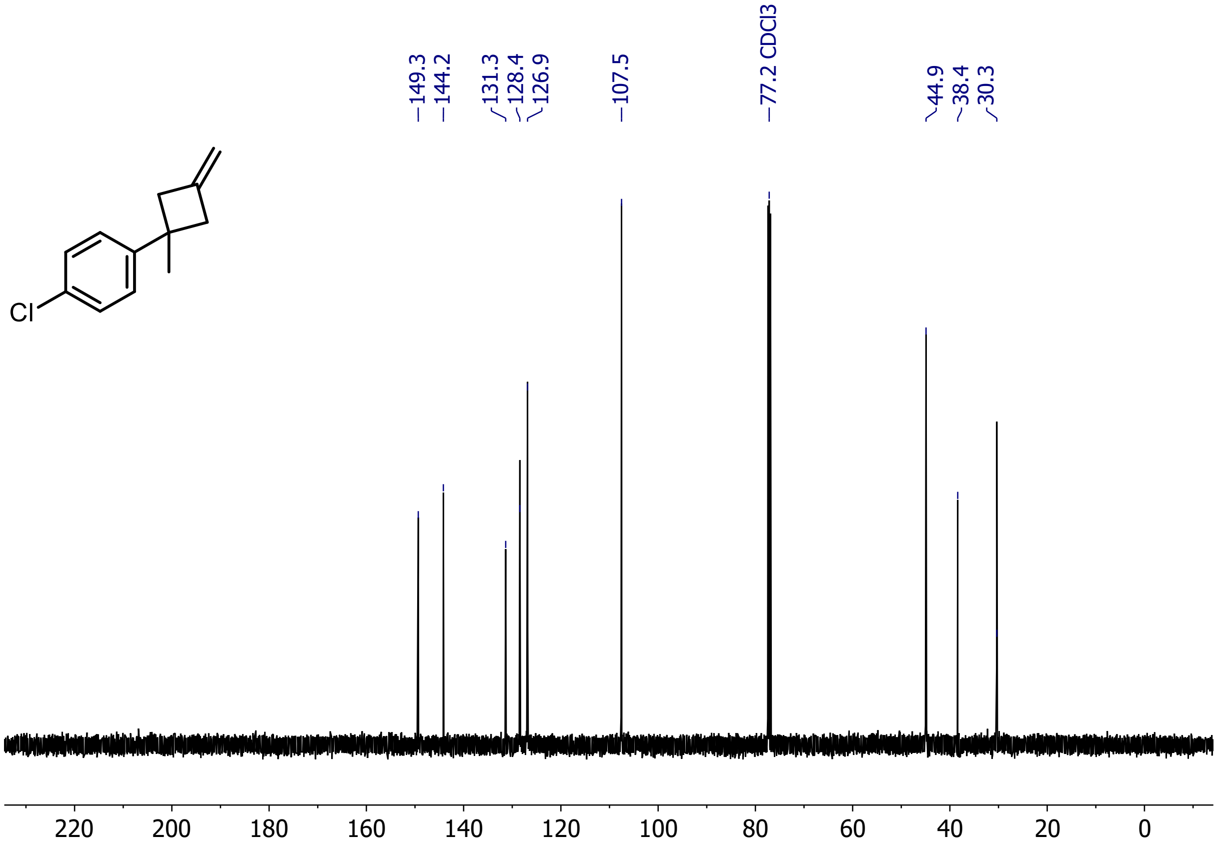

[1m], ^{1}H, $CDCl_3$, 599 MHz

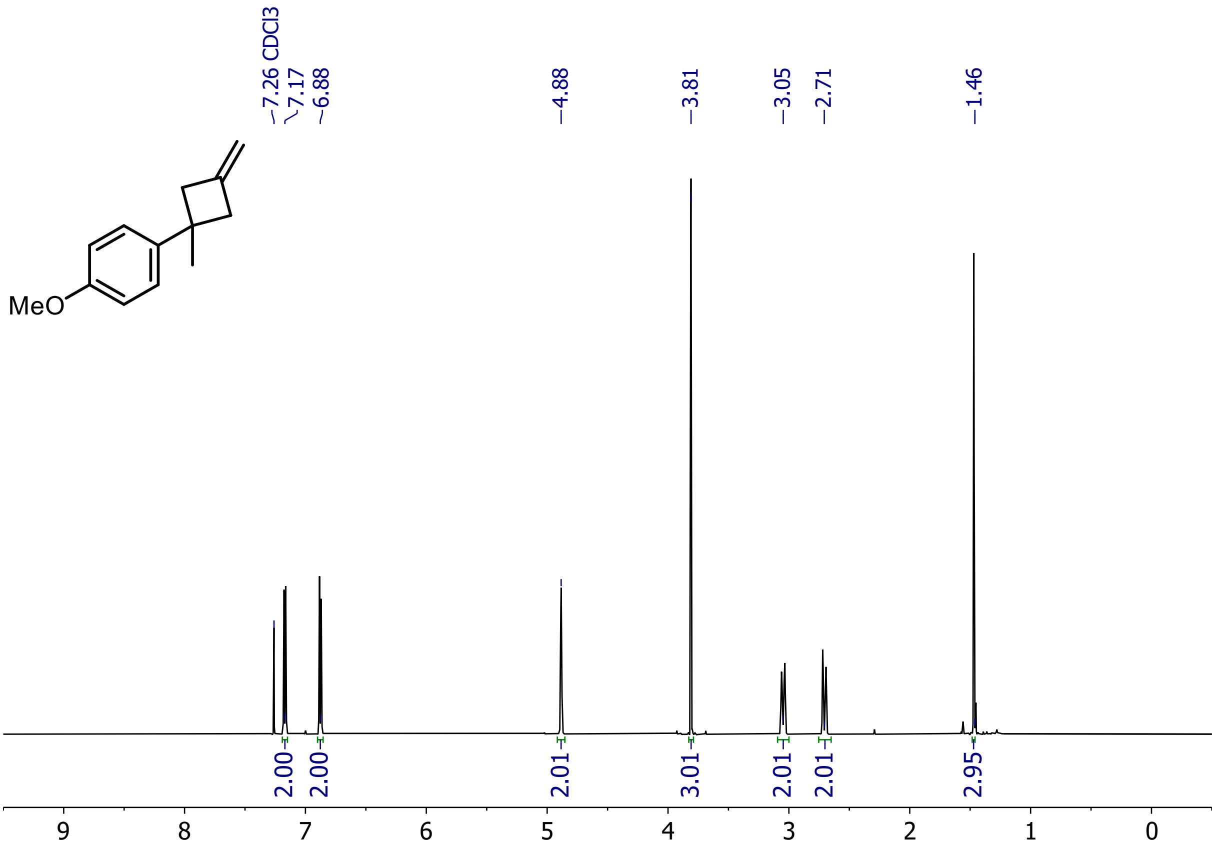

[1m], ^{13}C, $CDCl_3$, 151 MHz

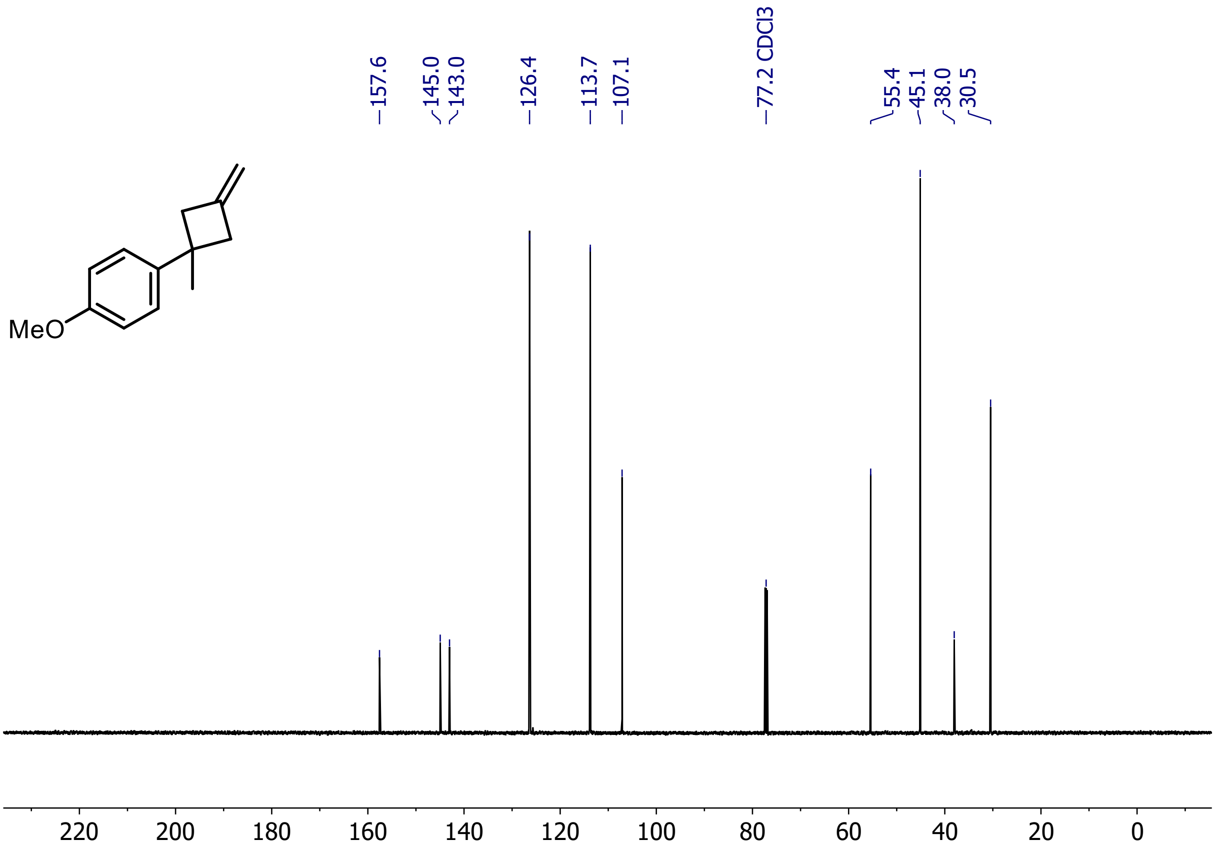

[1n], ^{1}H, $CDCl_3$, 599 MHz

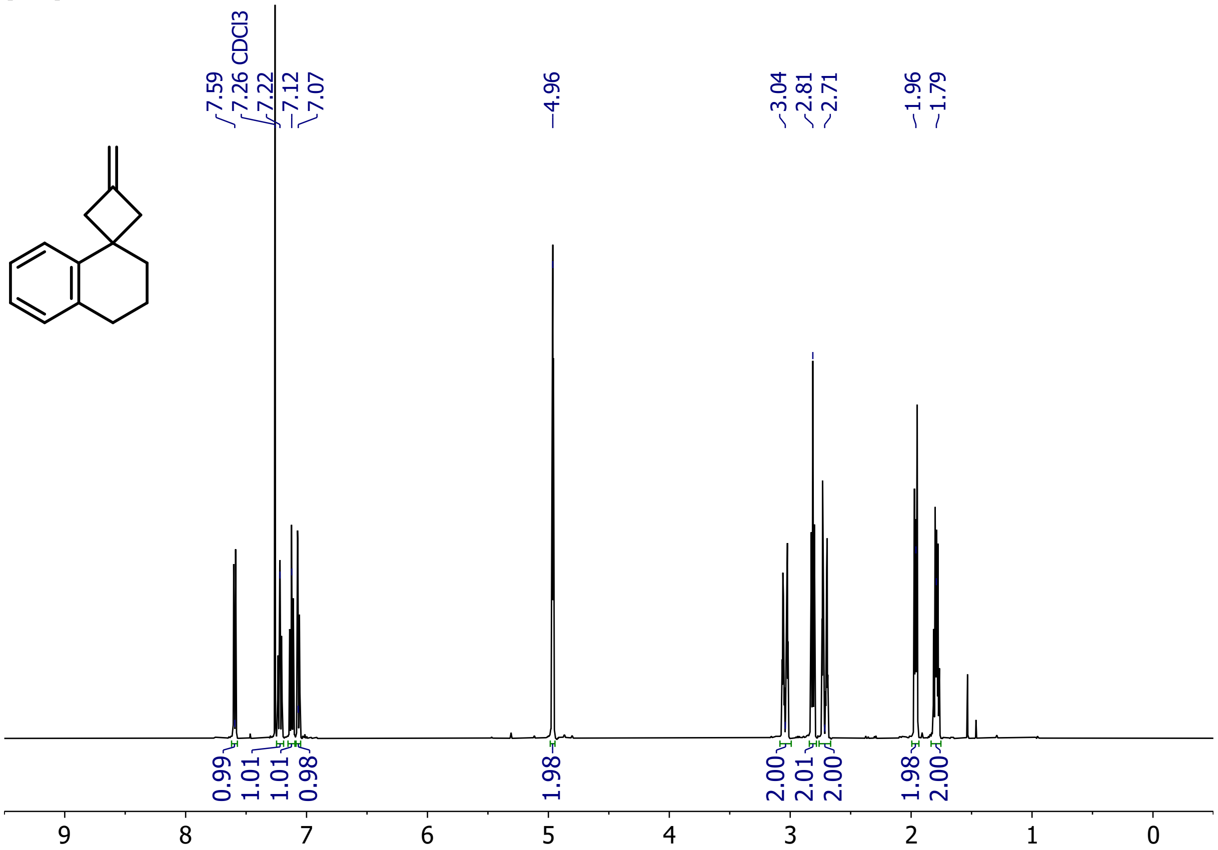

[1n], ^{13}C, $CDCl_3$, 151 MHz

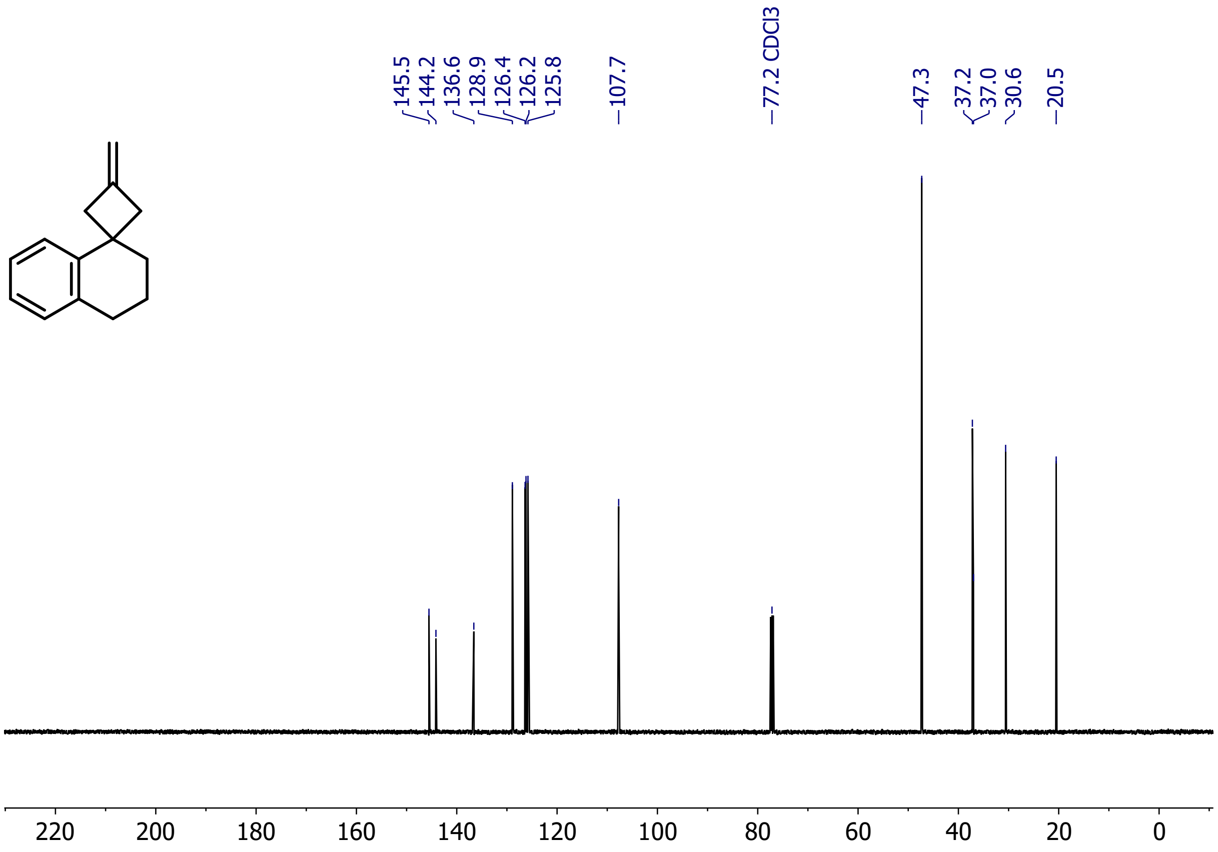

[1o], ^{1}H, $CDCl_3$, 599 MHz

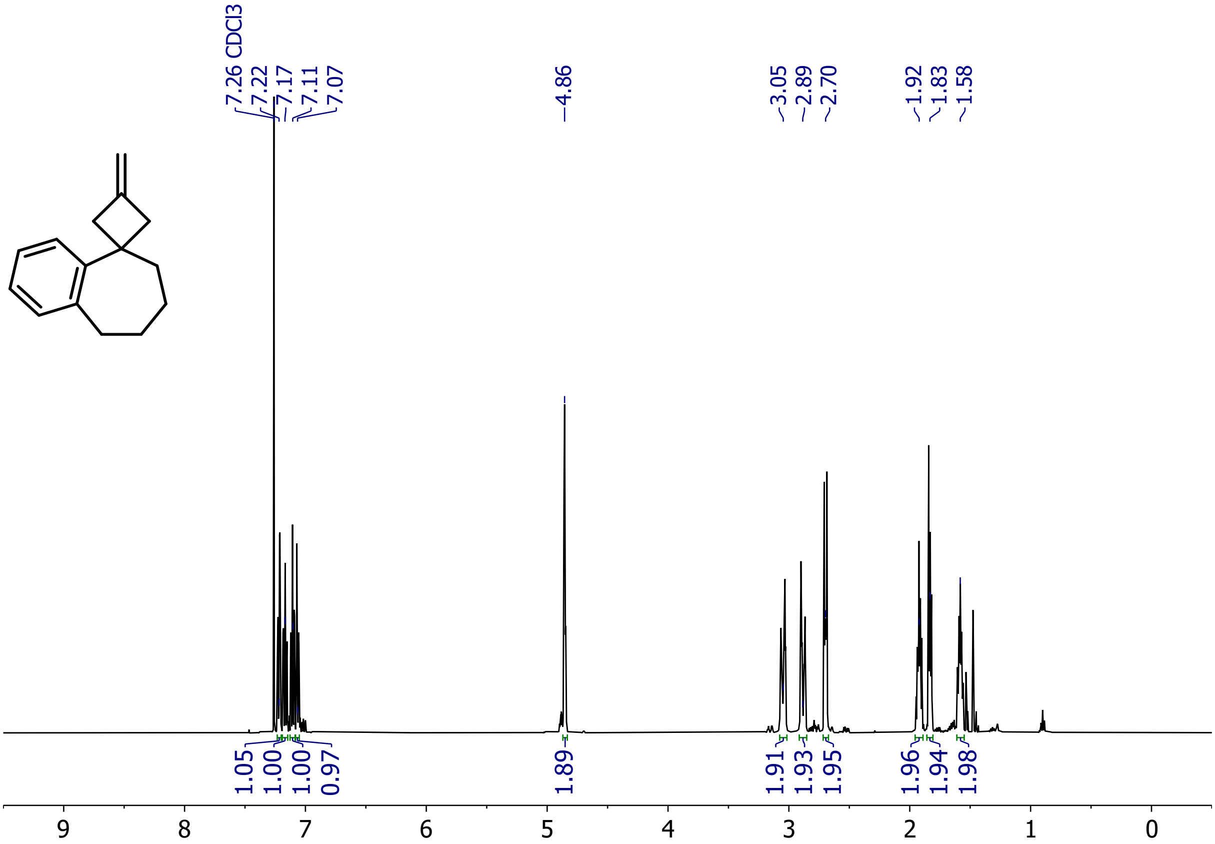

[1o], ^{13}C, $CDCl_3$, 151 MHz

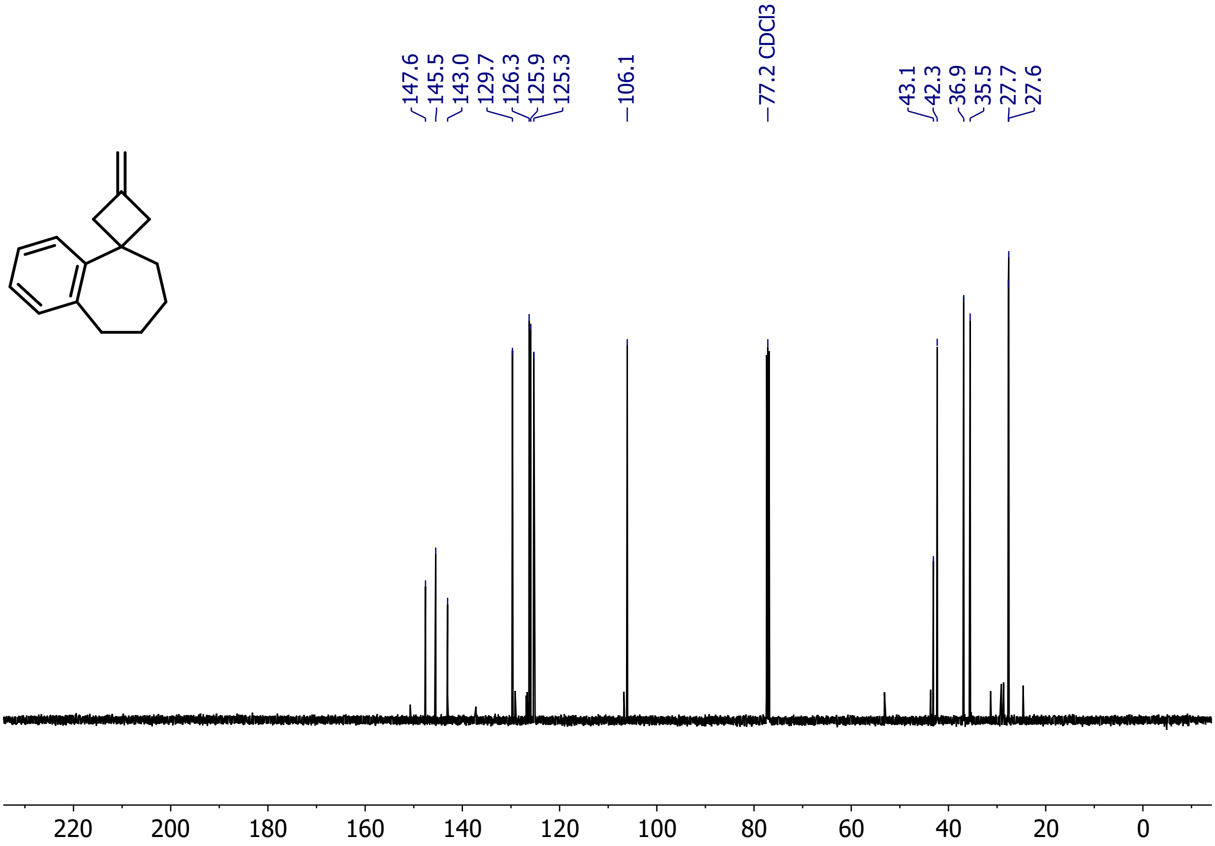

[1p], ^{1}H, $CDCl_3$, 599 MHz

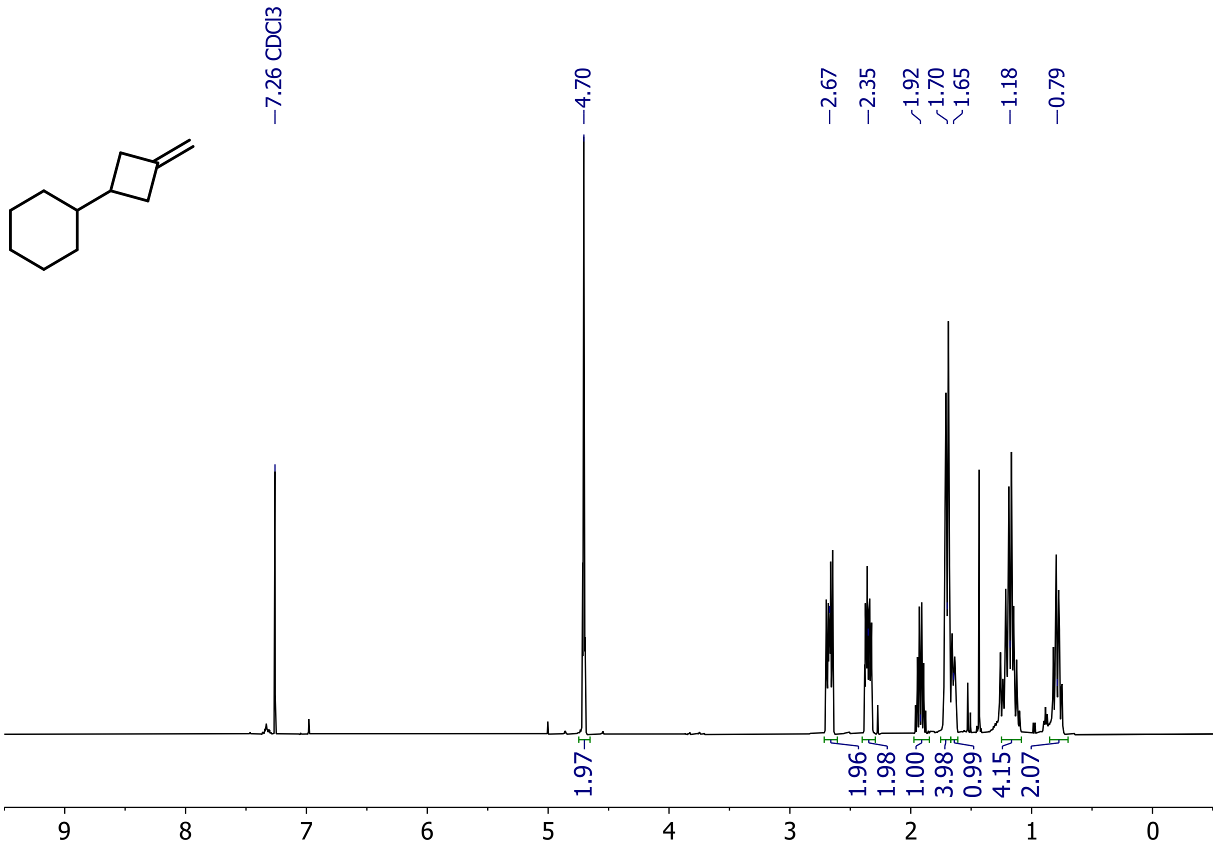

[1p], ^{13}C, $CDCl_3$, 151 MHz

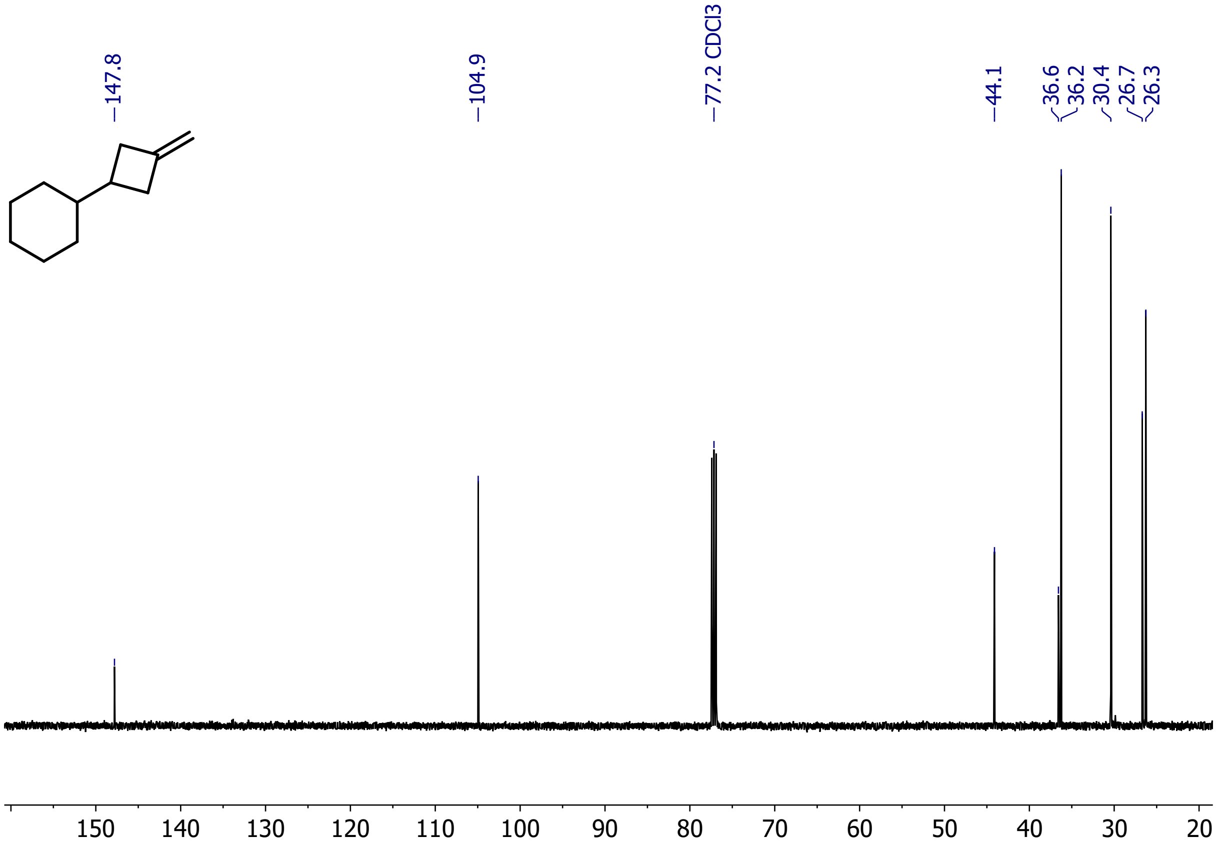

[1r], ^{1}H, $CDCl_3$, 400 MHz

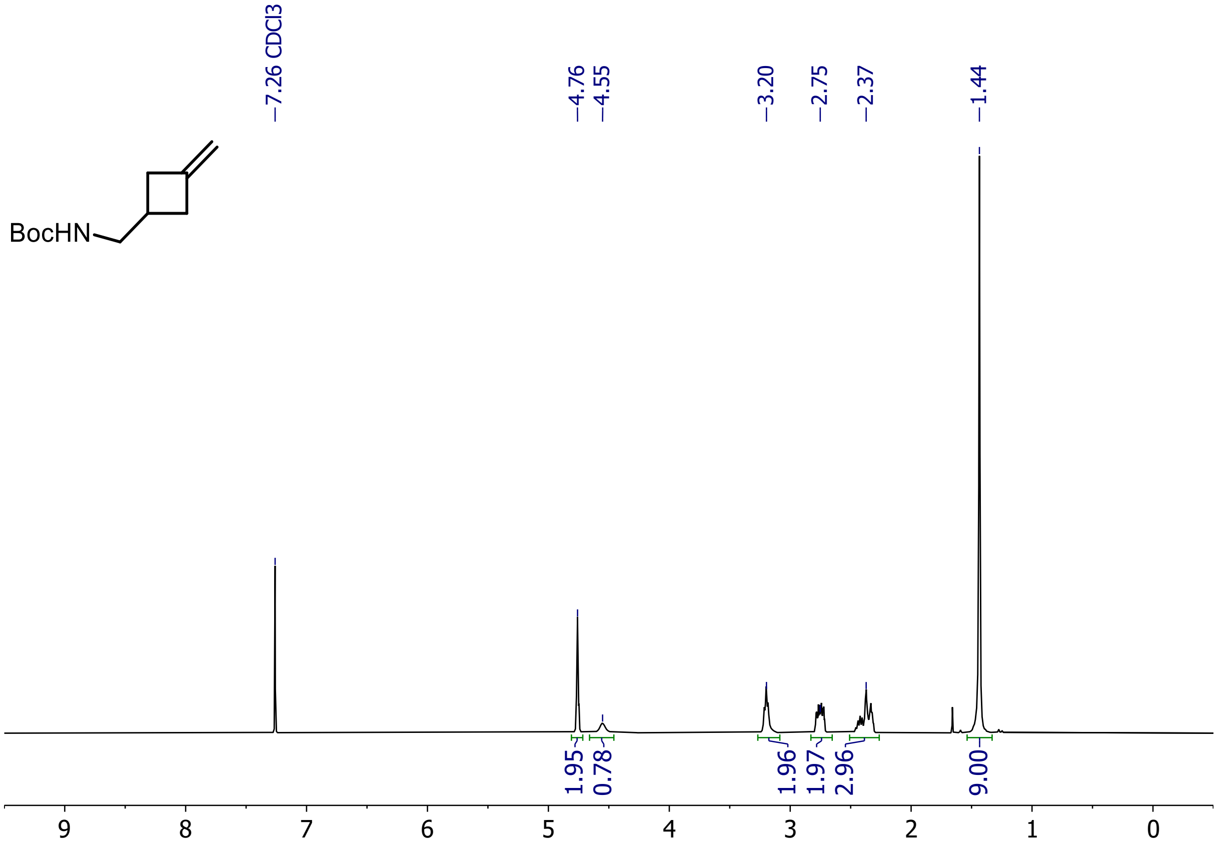

[1r], ^{13}C, $CDCl_3$, 101 MHz

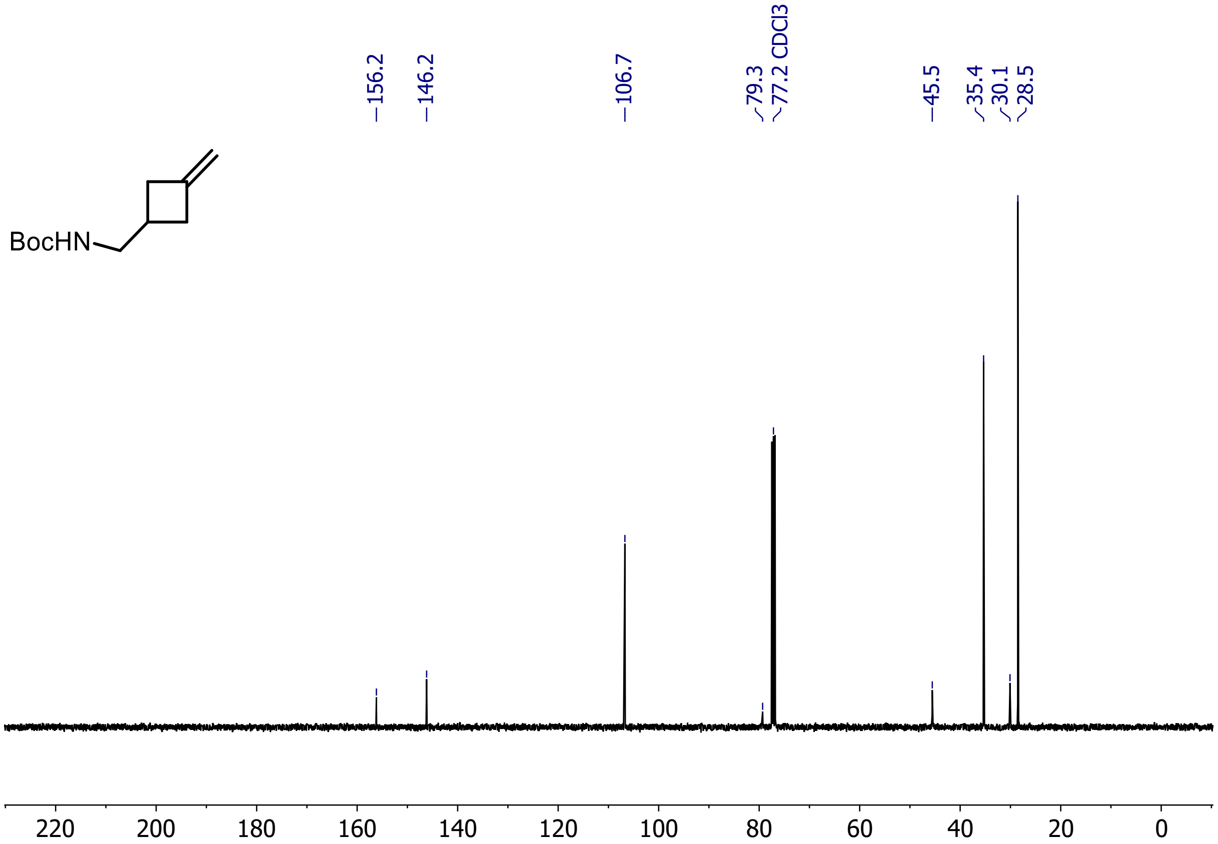

[S27], ^1H, $CDCl_3$, 400 MHz

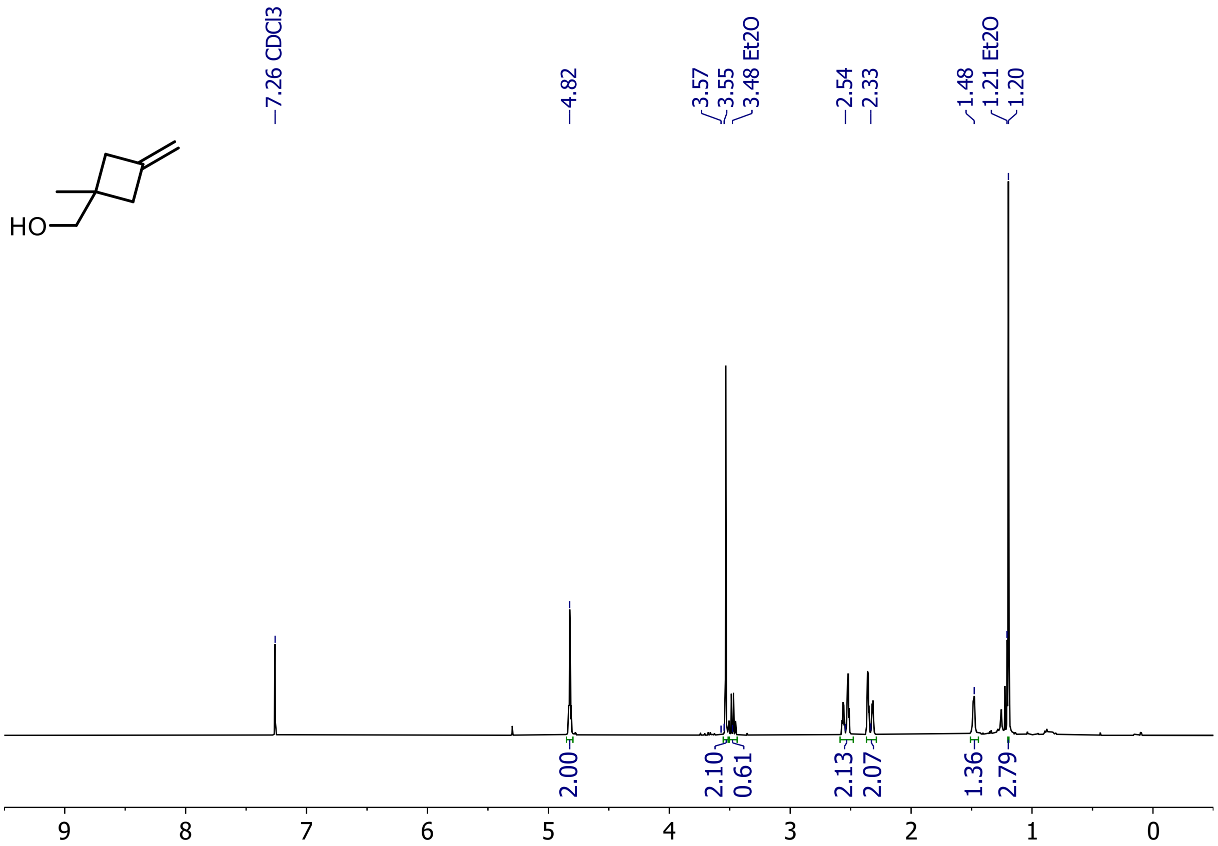

[S27], ^{13}C, $CDCl_3$, 101 MHz

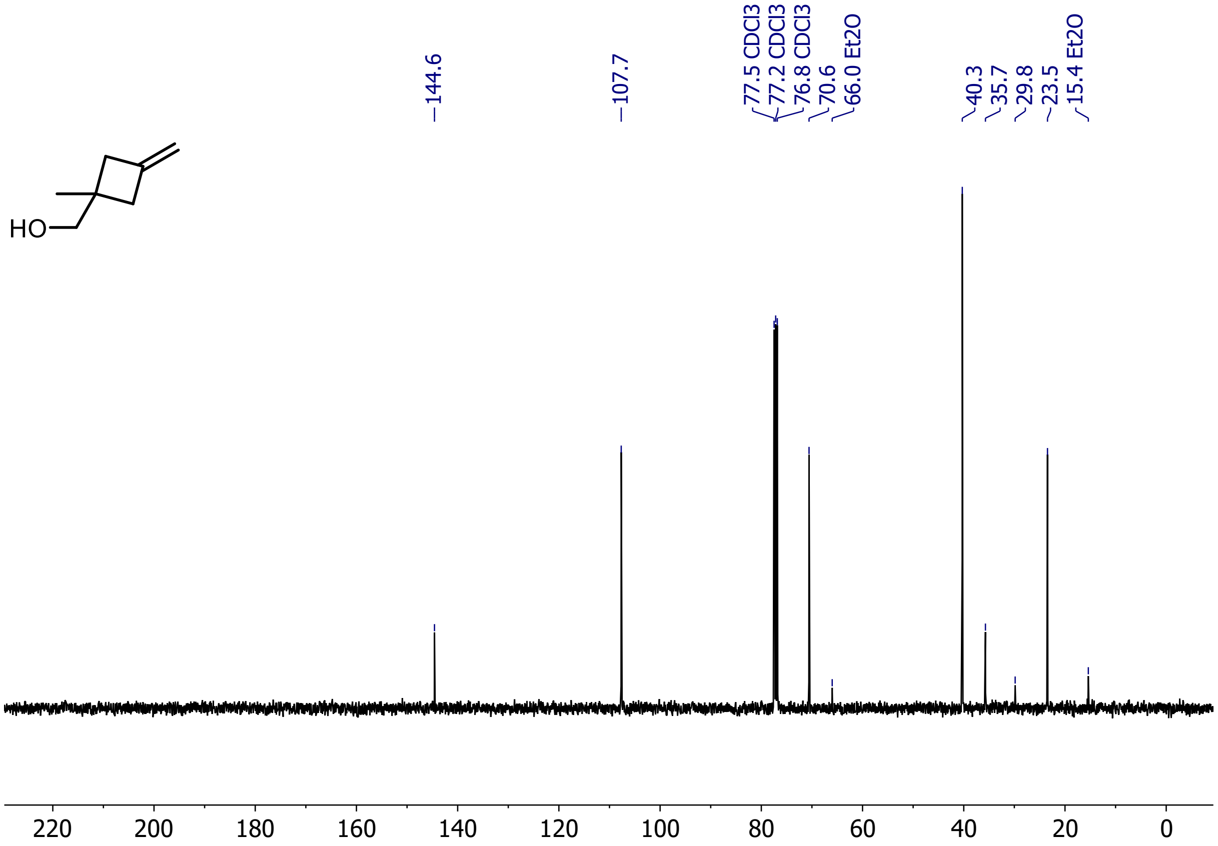

[1s], ^{1}H, $CDCl_3$, 400 MHz

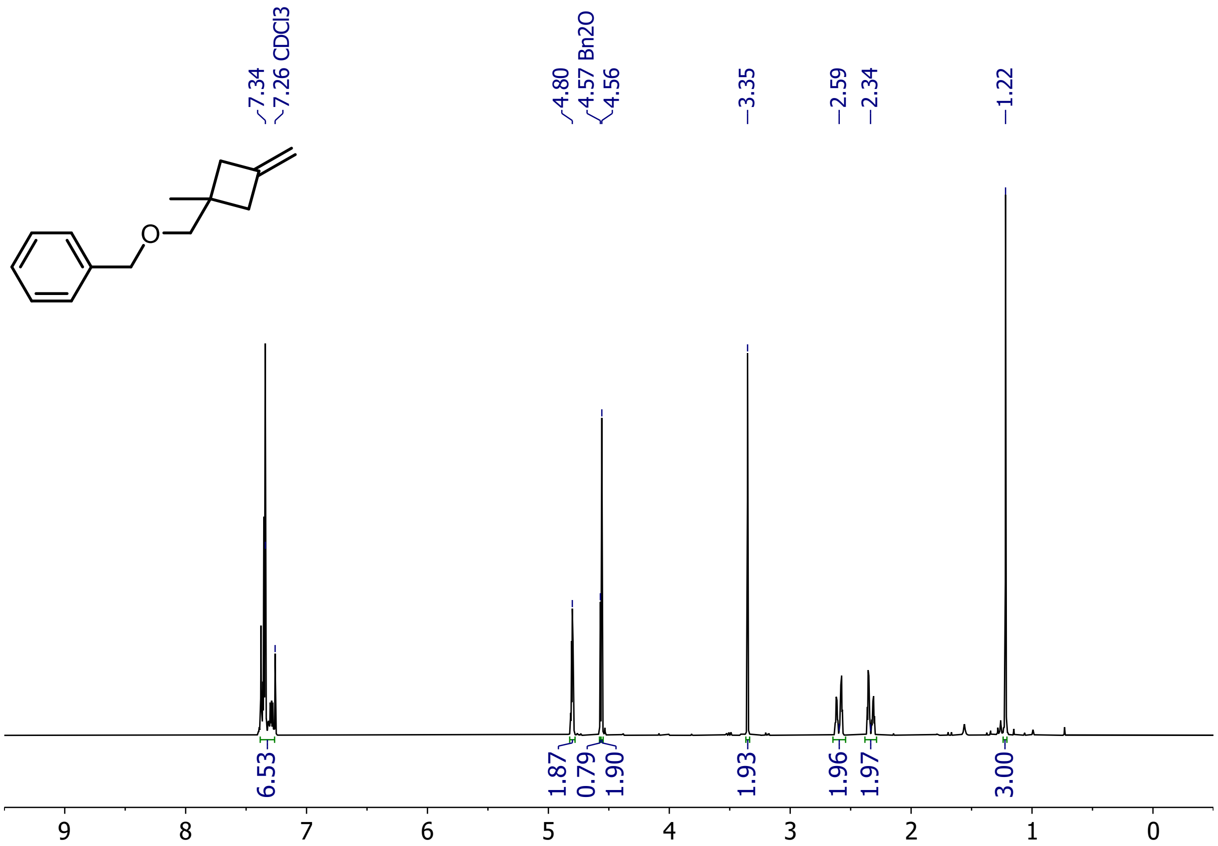

[1s], ^{13}C, $CDCl_3$, 101 MHz

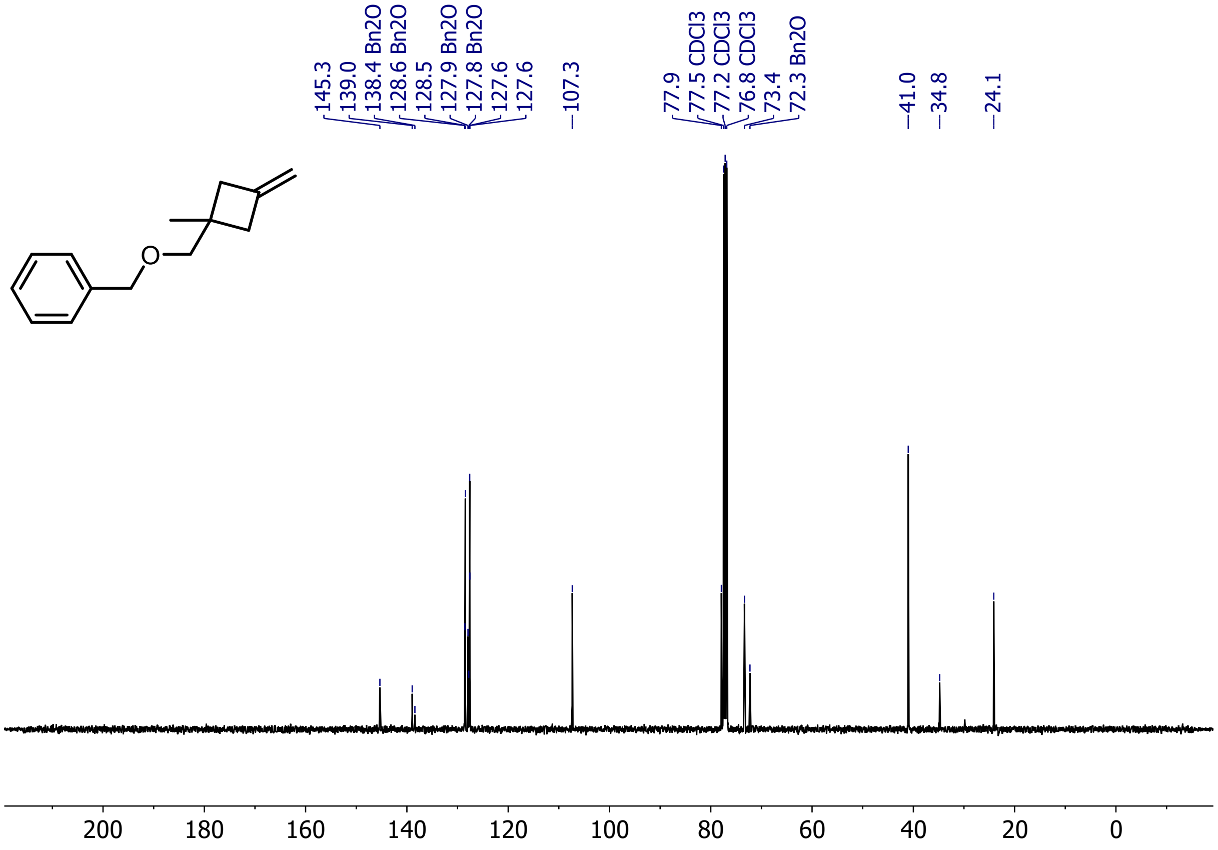

[1t], ^{1}H, $CDCl_3$, 400 MHz

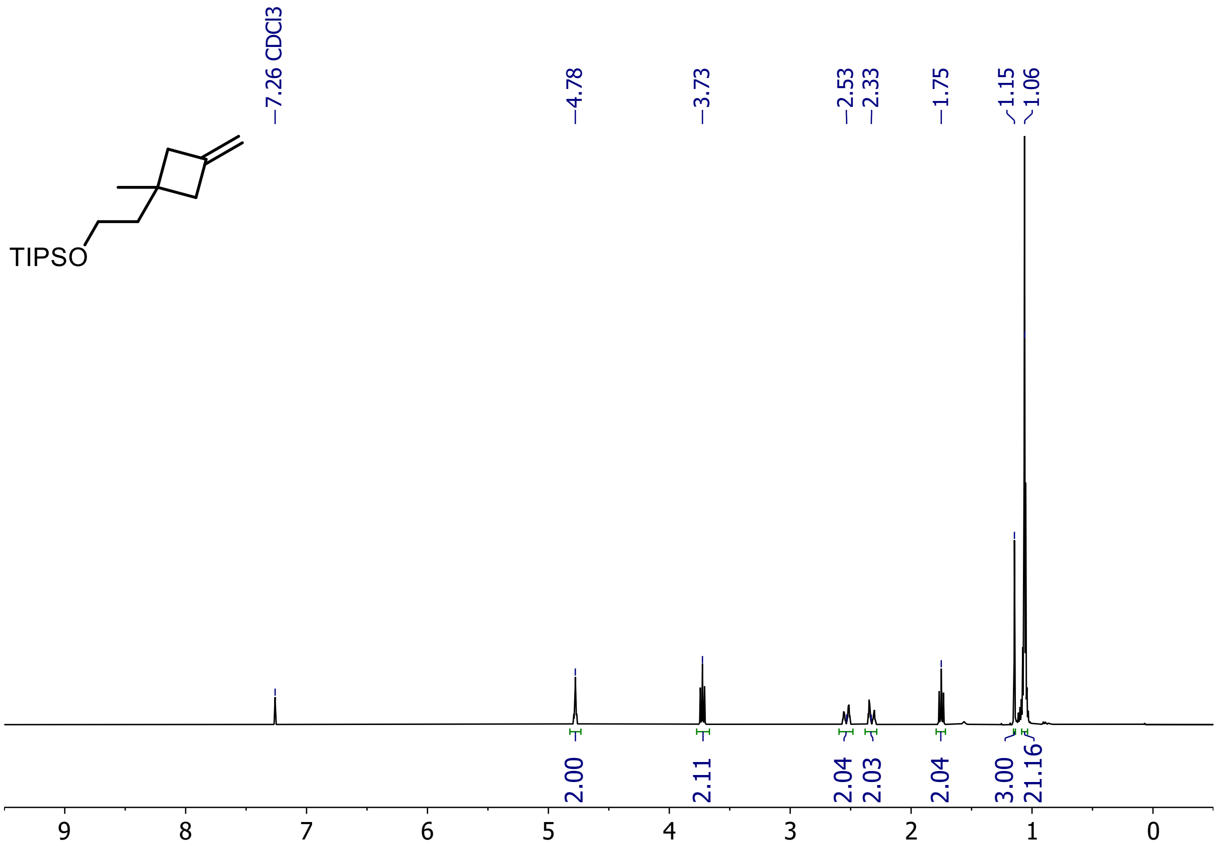

[1t], ^{13}C, $CDCl_3$, 101 MHz

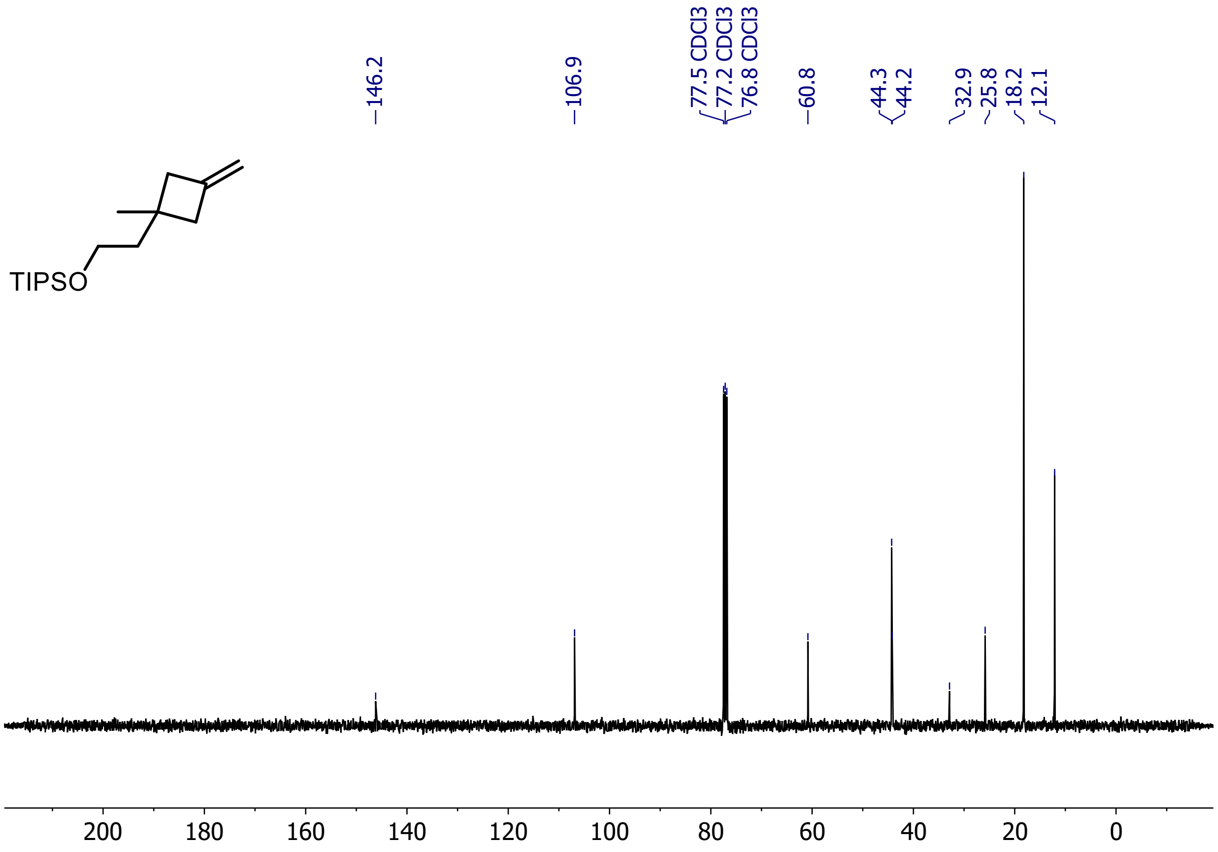

[1u], ^{1}H, $CDCl_3$, 400 MHz

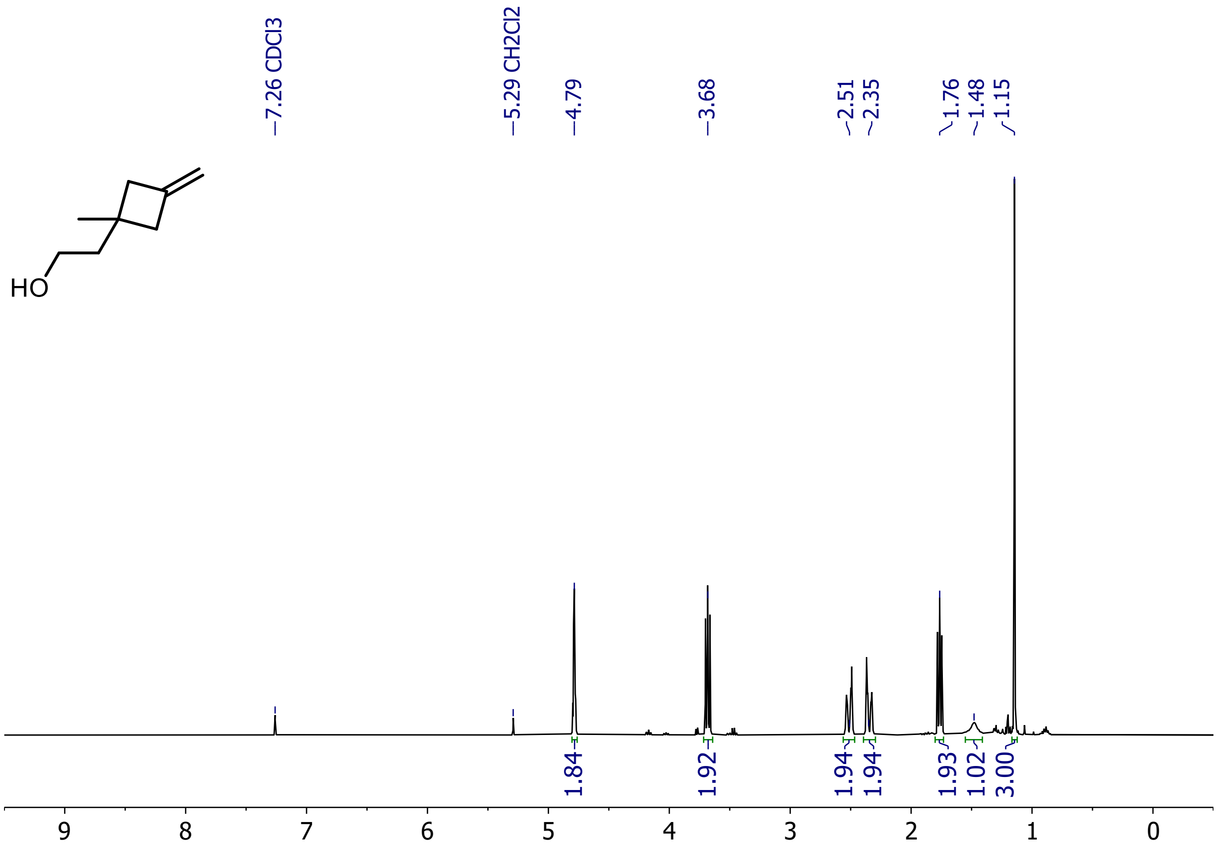

[1u], ^{13}C, $CDCl_3$, 101 MHz

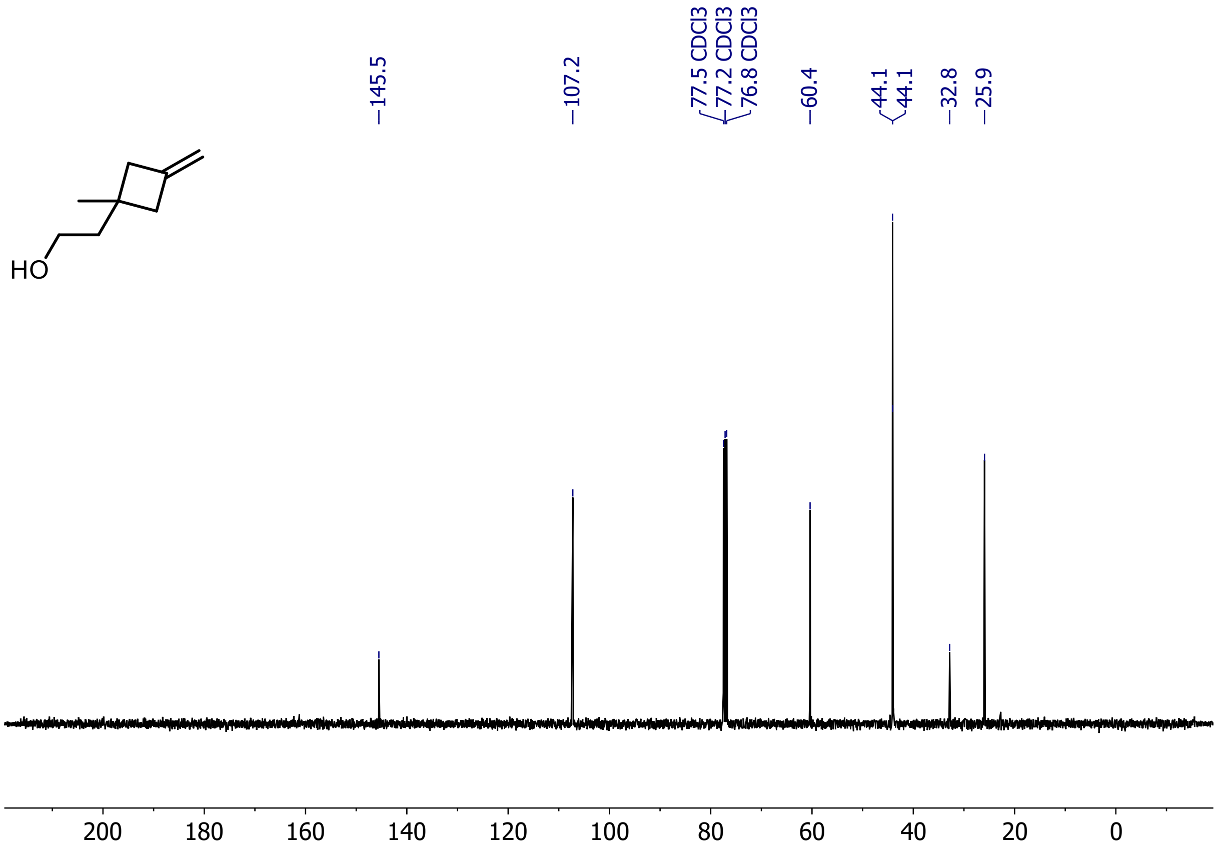

[1v], ^{1}H, $CDCl_3$, 400 MHz

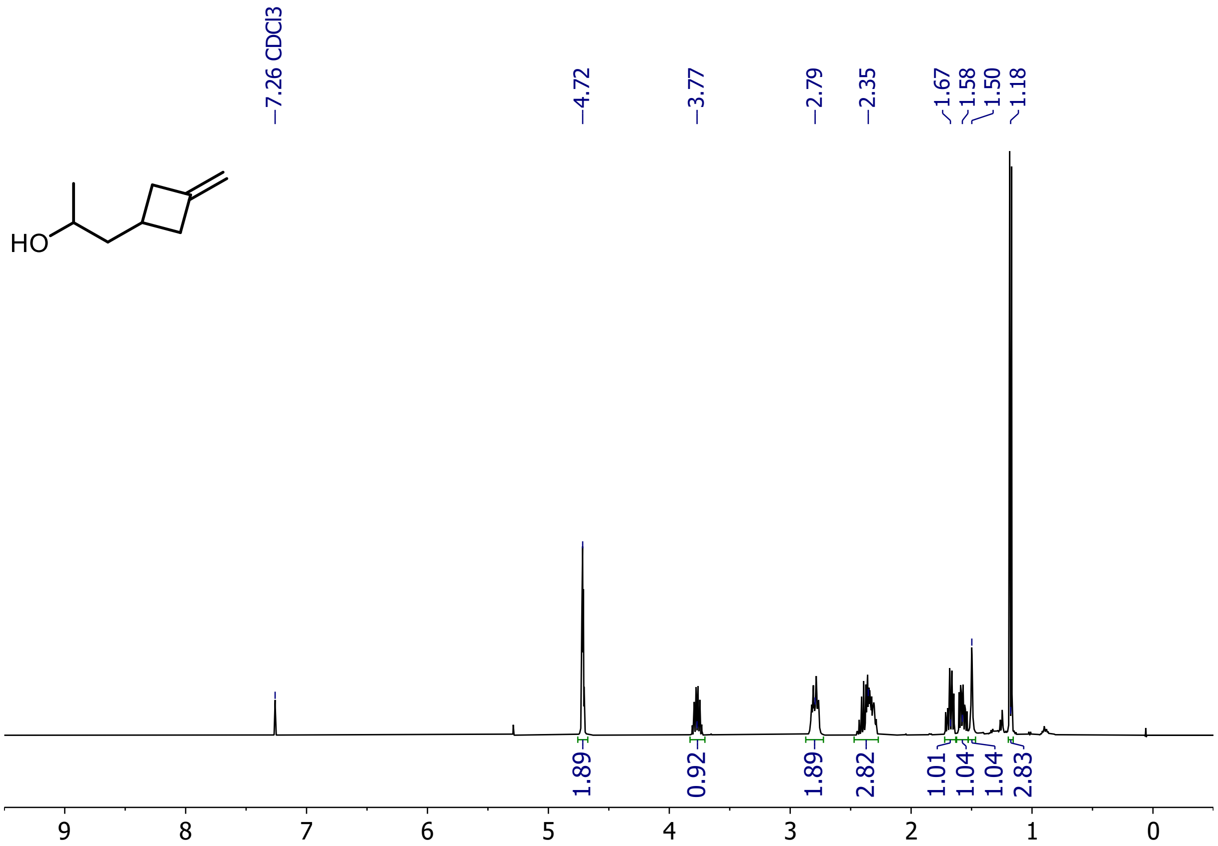

[1v], ^{13}C, $CDCl_3$, 101 MHz

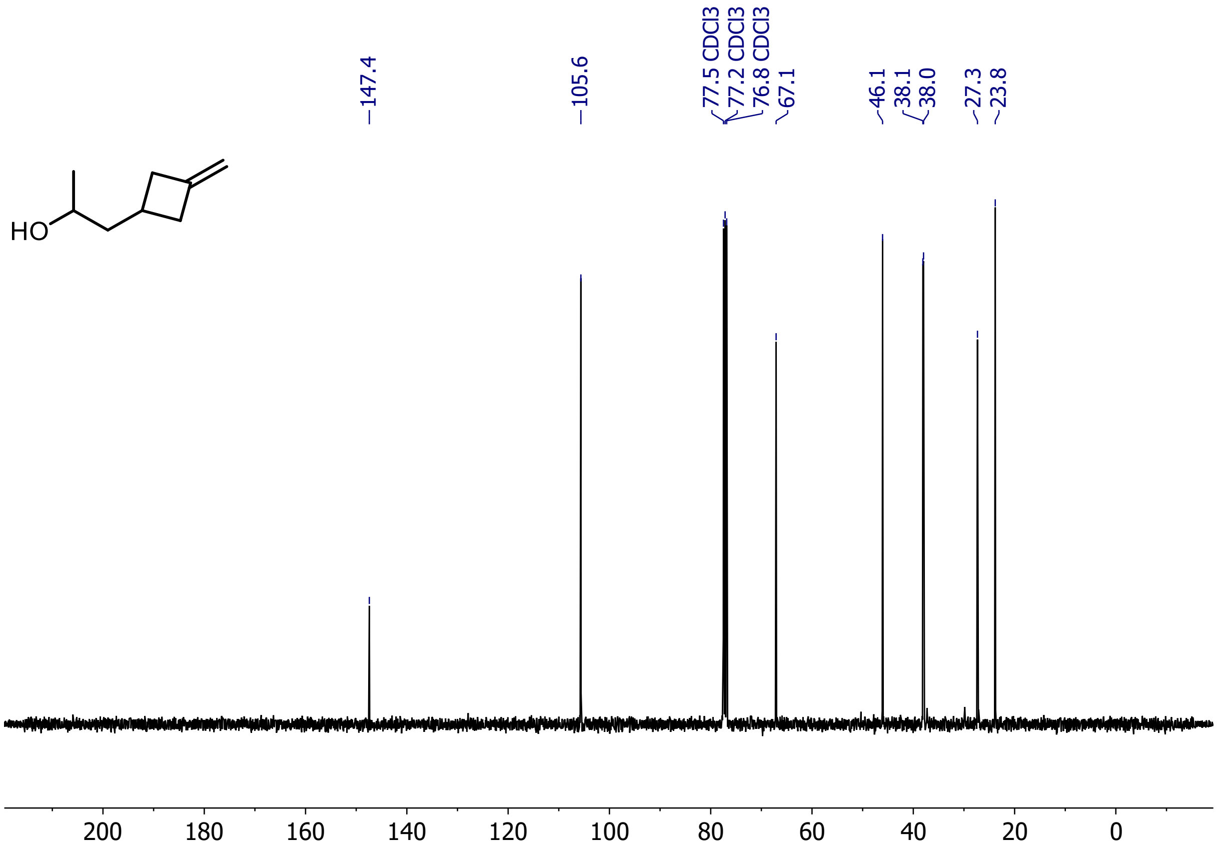

[1w], ^{1}H, $CDCl_3$, 400 MHz

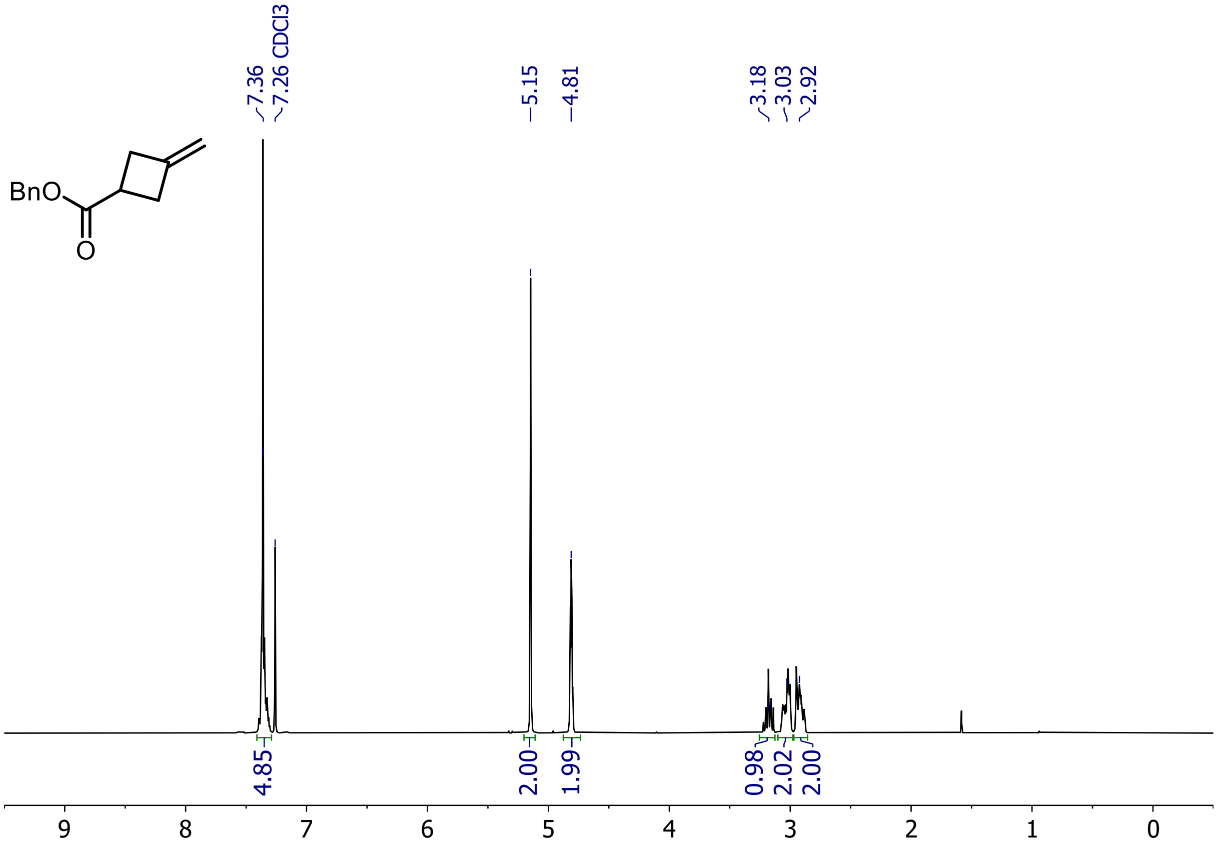

[1w], ^{13}C, $CDCl_3$, 101 MHz

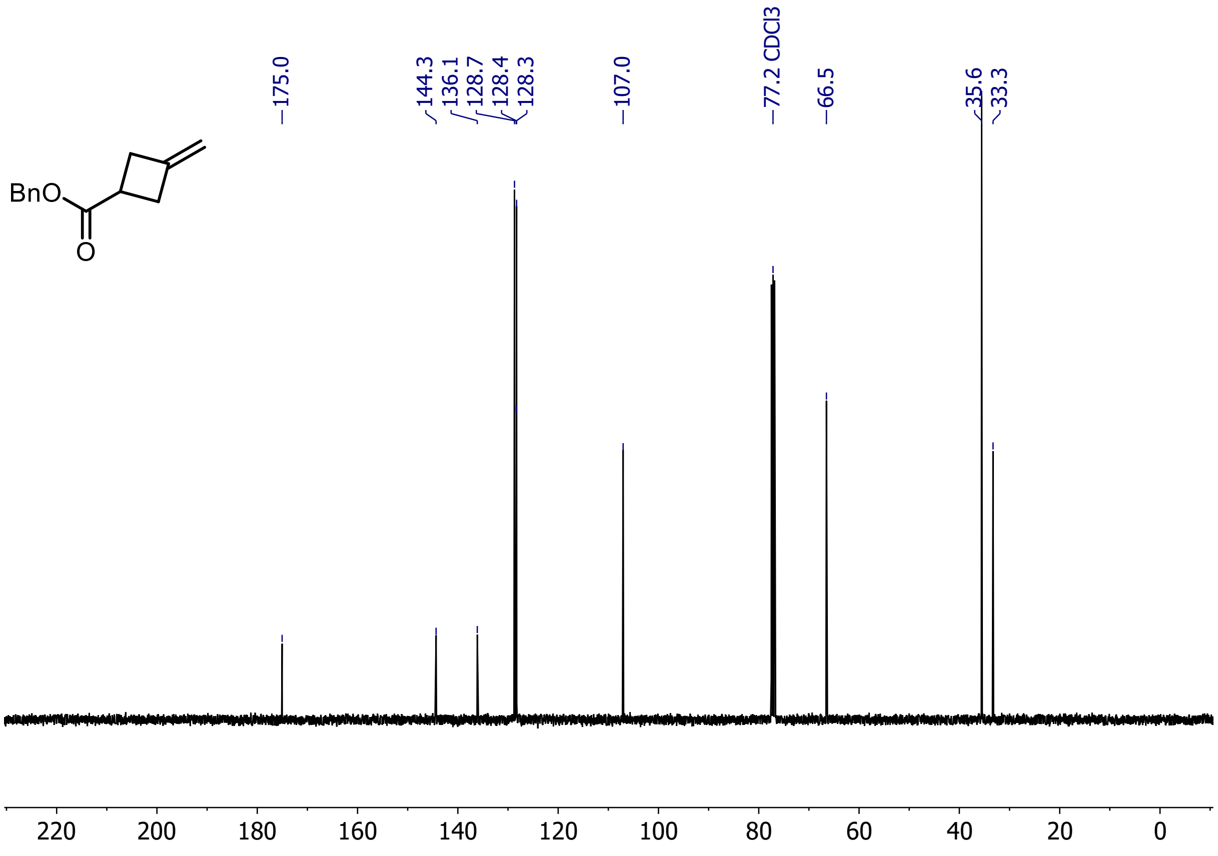

[1x], ^{1}H, $CDCl_3$, 599 MHz

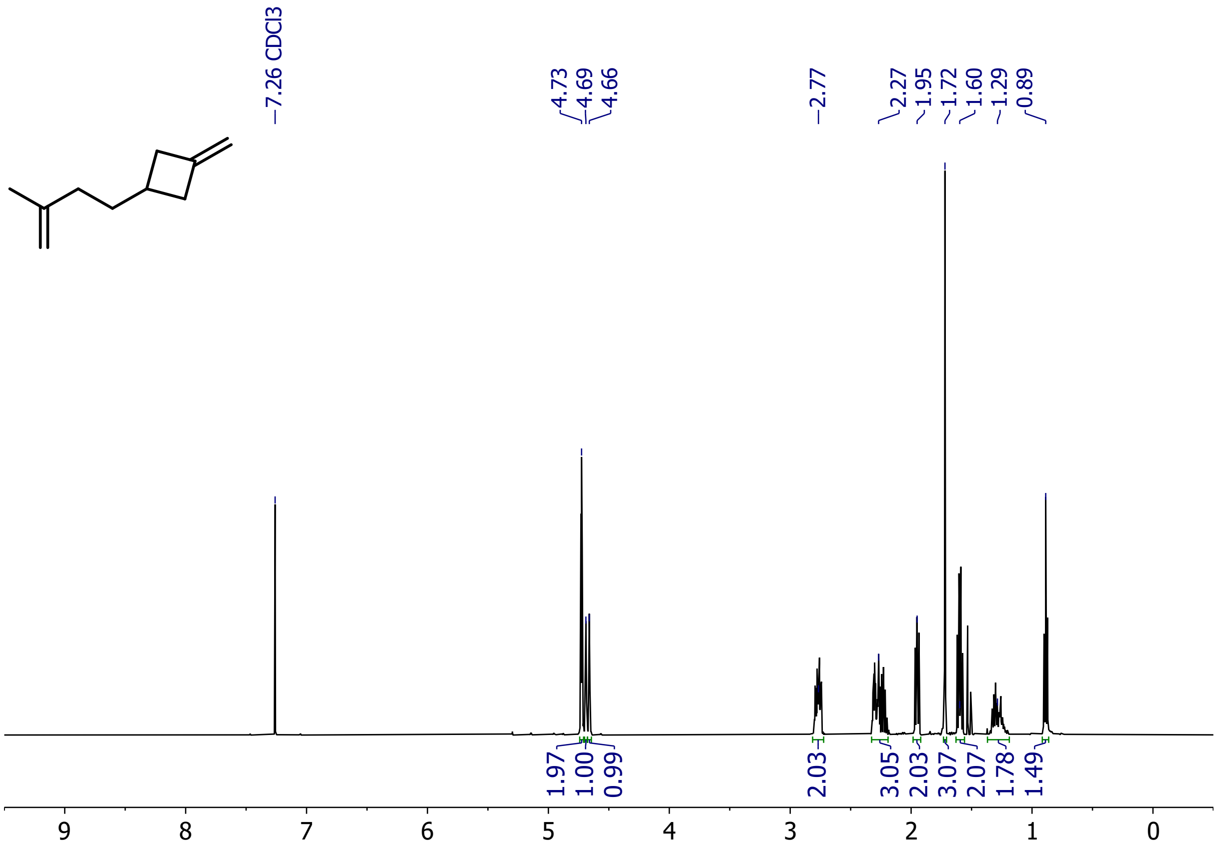

[1x], ^{13}C, $CDCl_3$, 151 MHz

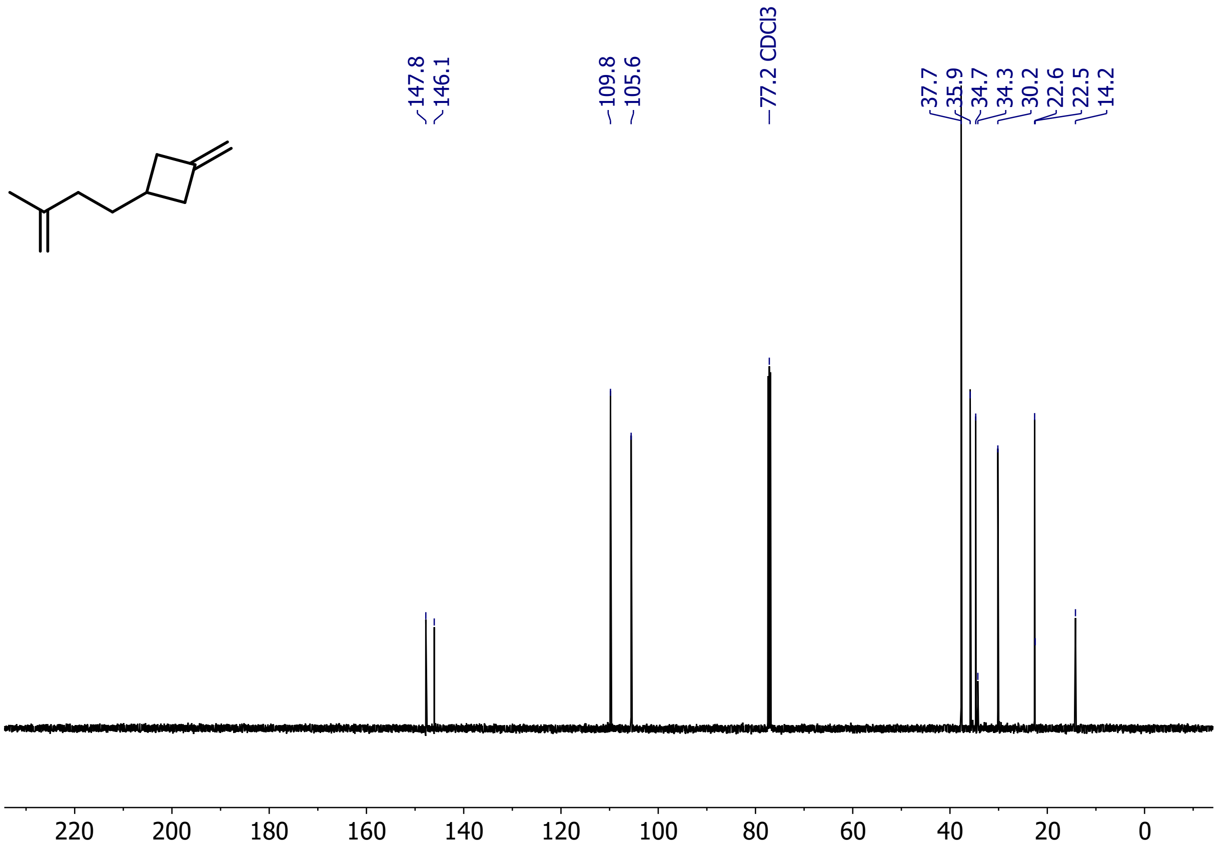

[5], ^{1}H, $CDCl_3$, 400 MHz

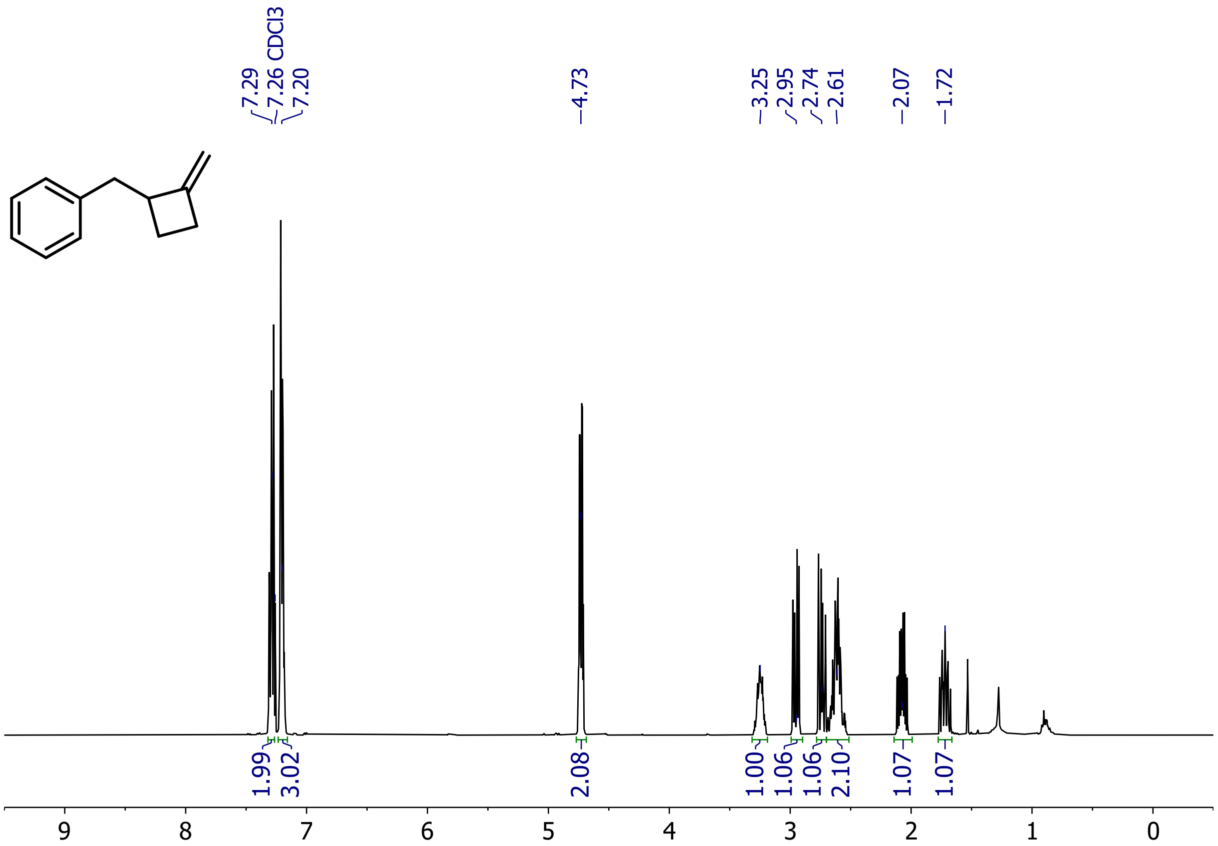

[5], ^{13}C, $CDCl_3$, 101 MHz

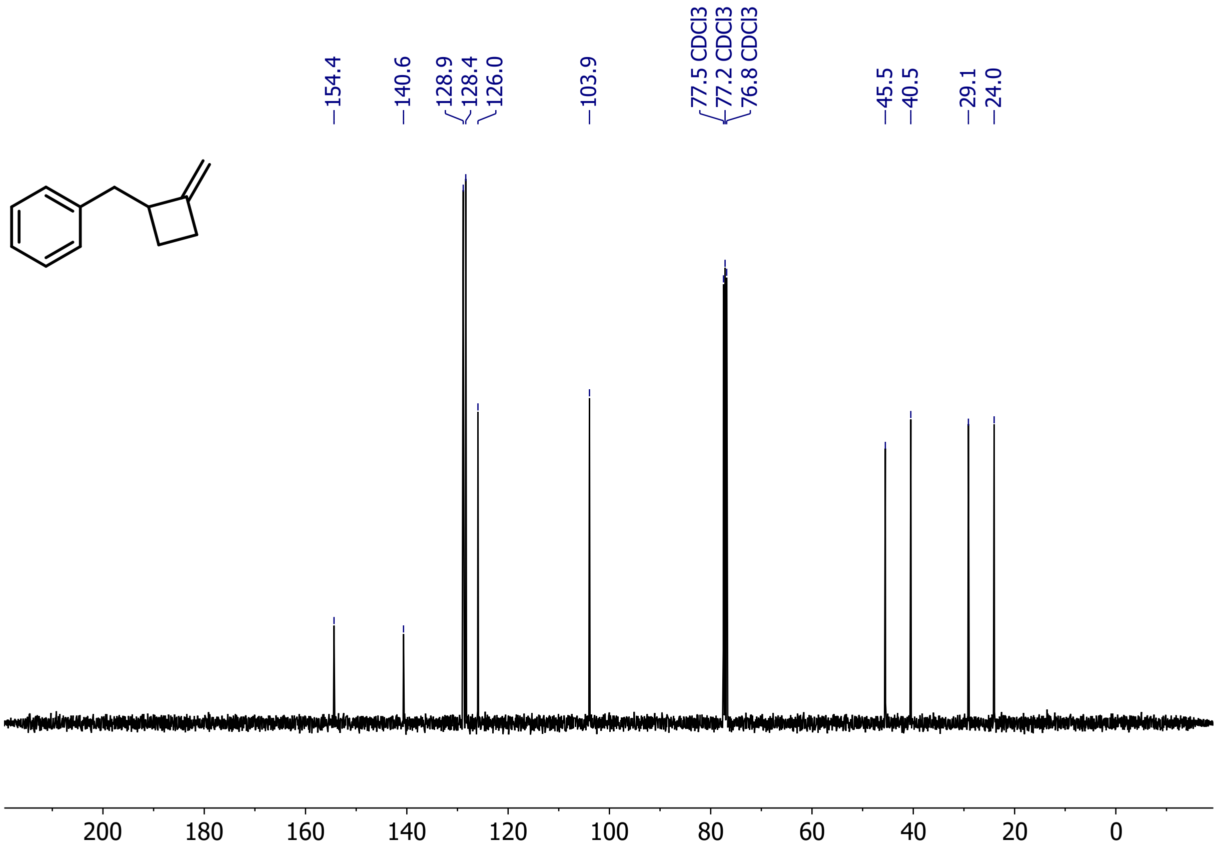

[S30], ^{1}H, $CDCl_3$, 400 MHz

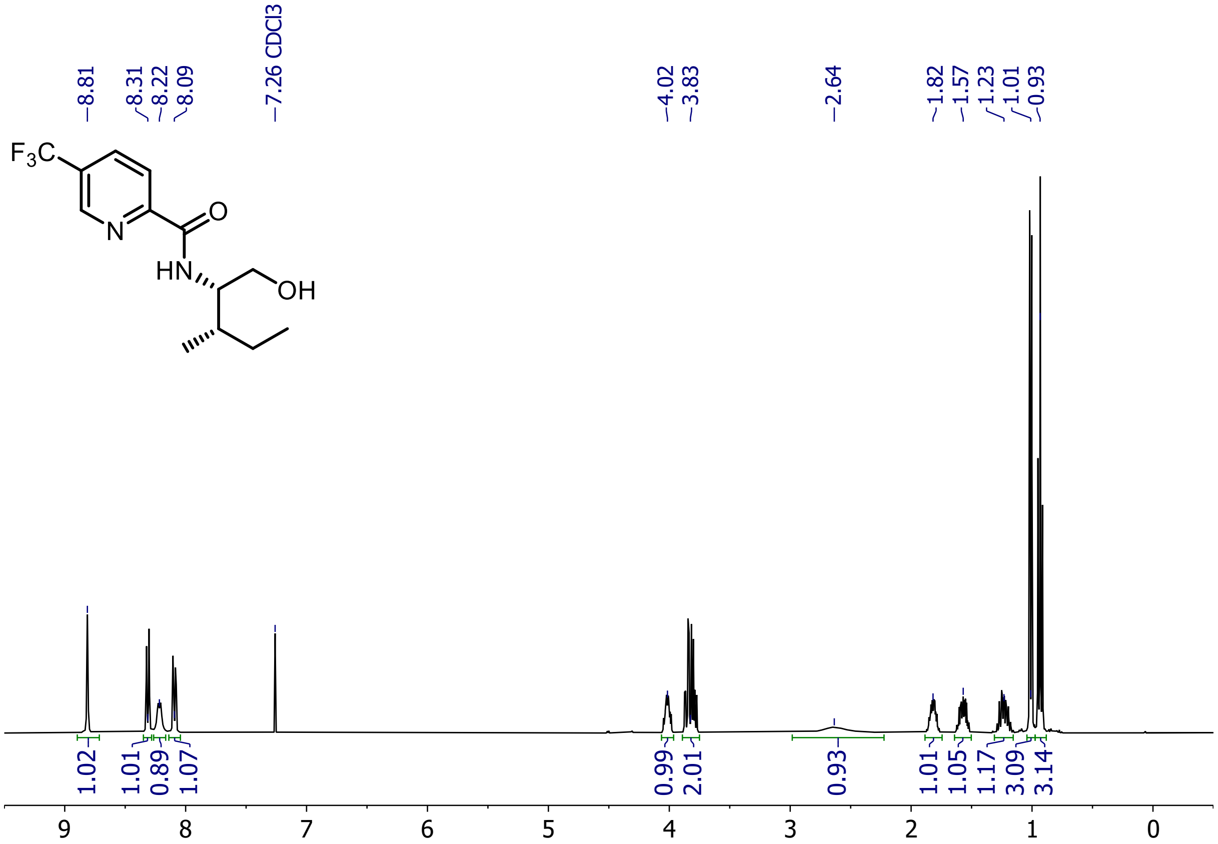

[S30], ^{13}C, $CDCl_3$, 101 MHz

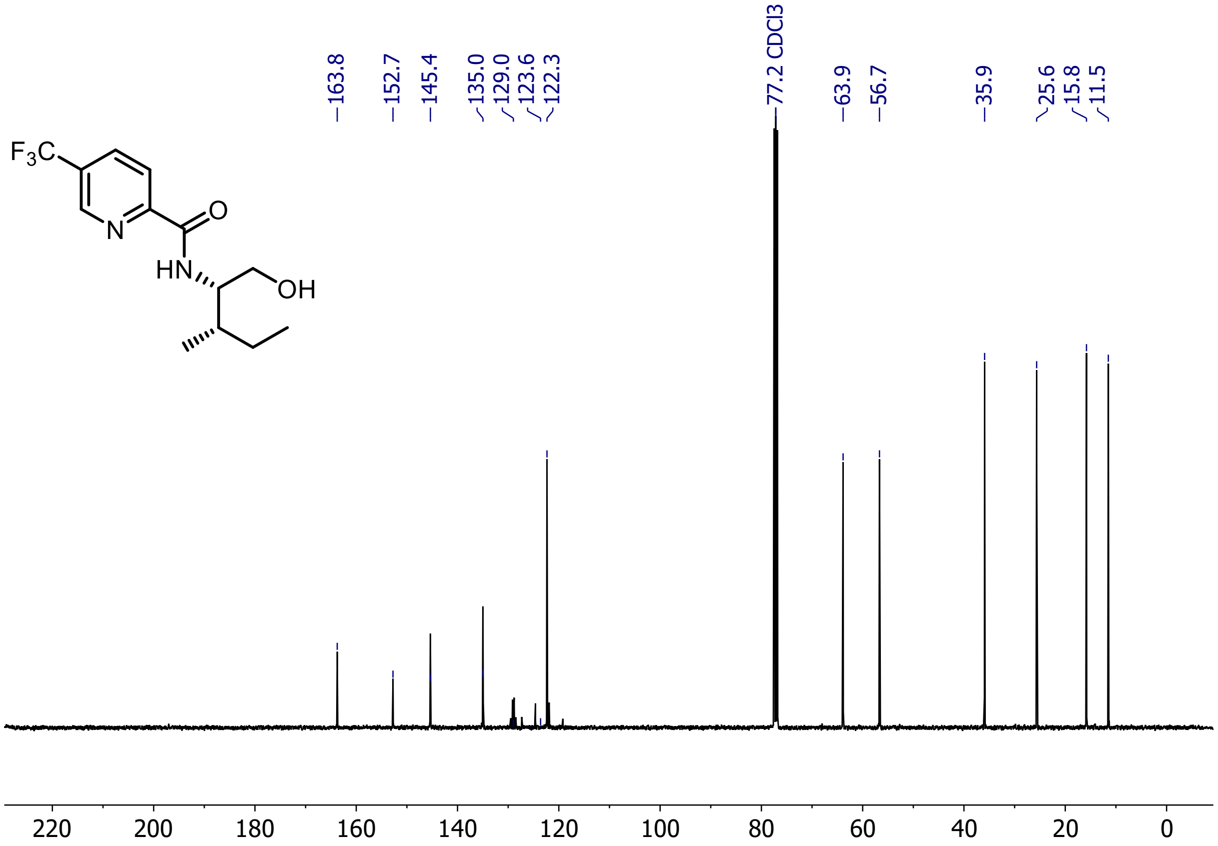

[S30], ^{19}F, $CDCl_3$, 282 MHz

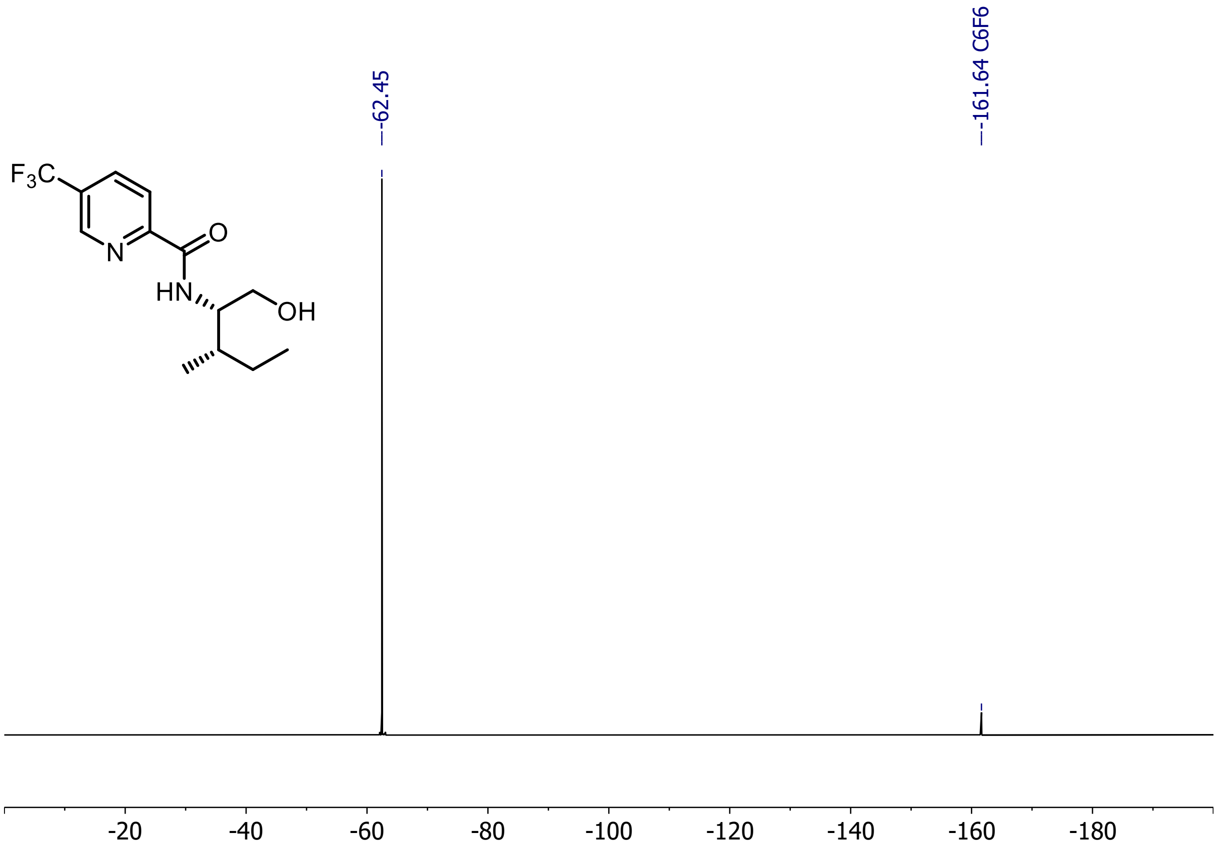

[L1], ^{1}H, $CDCl_3$, 400 MHz

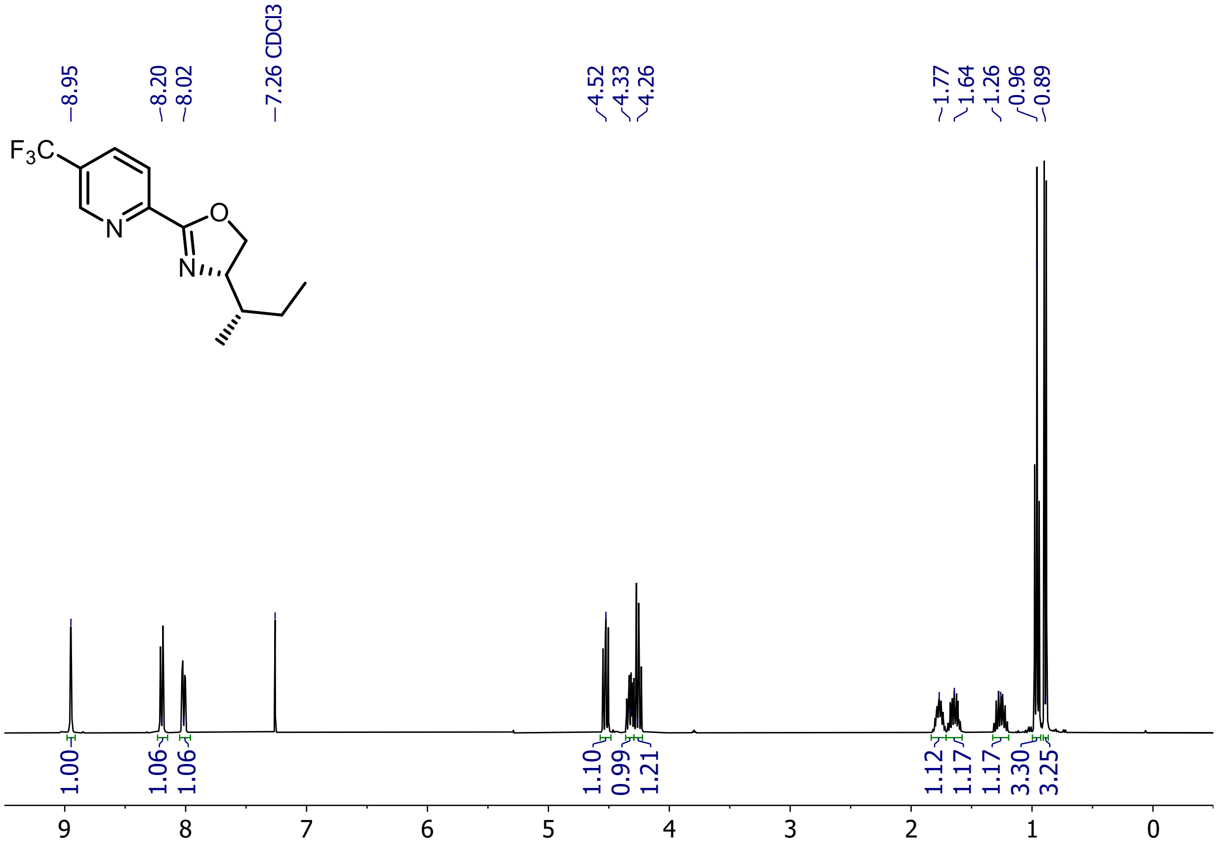

[L1], ^{13}C, $CDCl_3$, 101 MHz

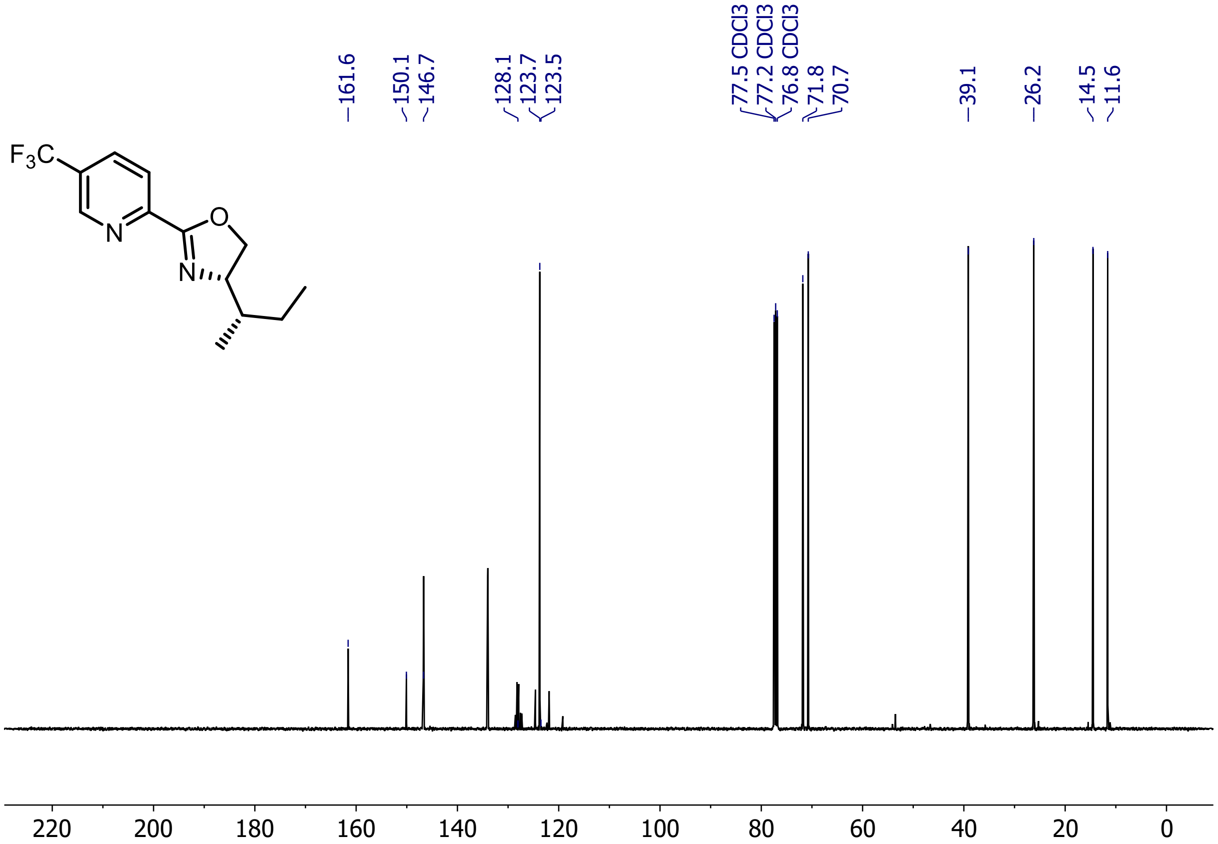

[L1], ^{19}F, $CDCl_3$, 282 MHz

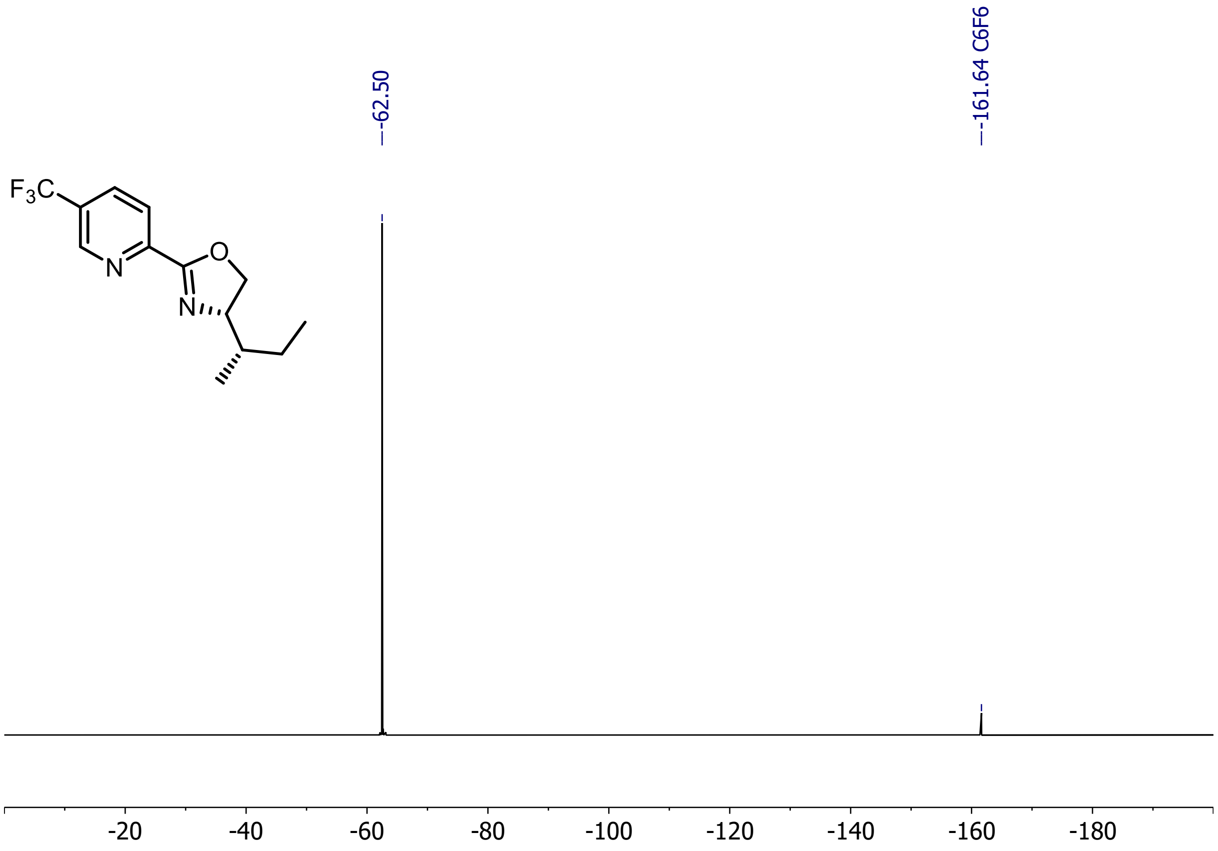

[4a], ^{1}H, $CDCl_3$, 400 MHz

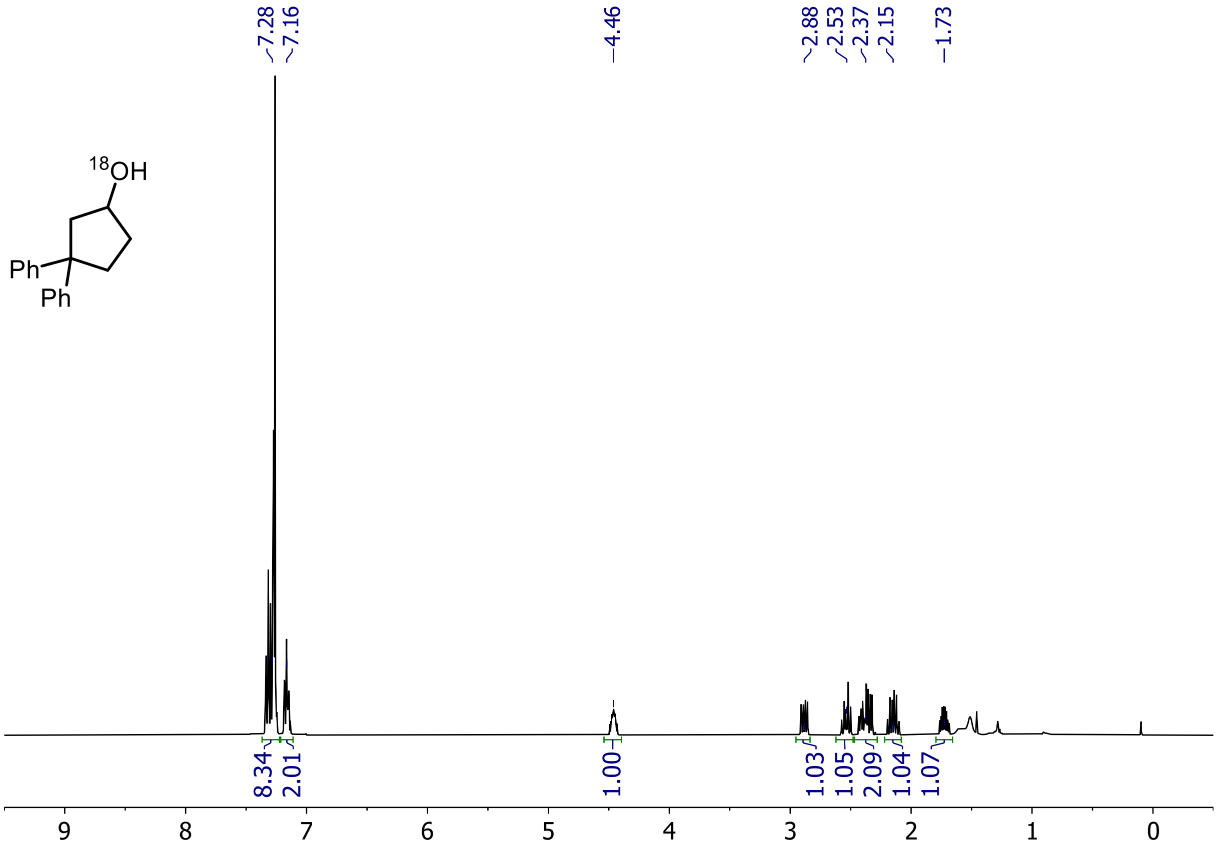

[4a], ^{13}C, $CDCl_3$, 101 MHz

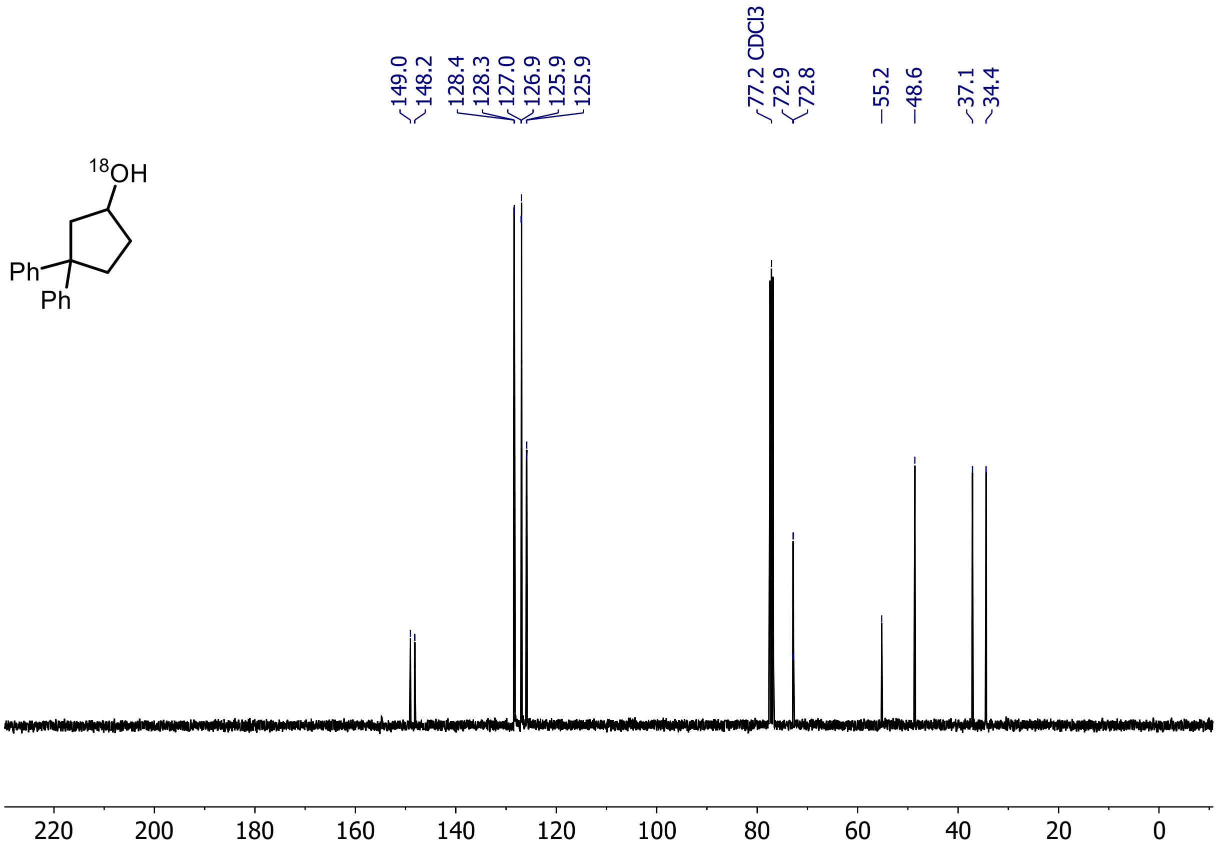

[13C-1a], ^{1}H, $CDCl_3$, 400 MHz

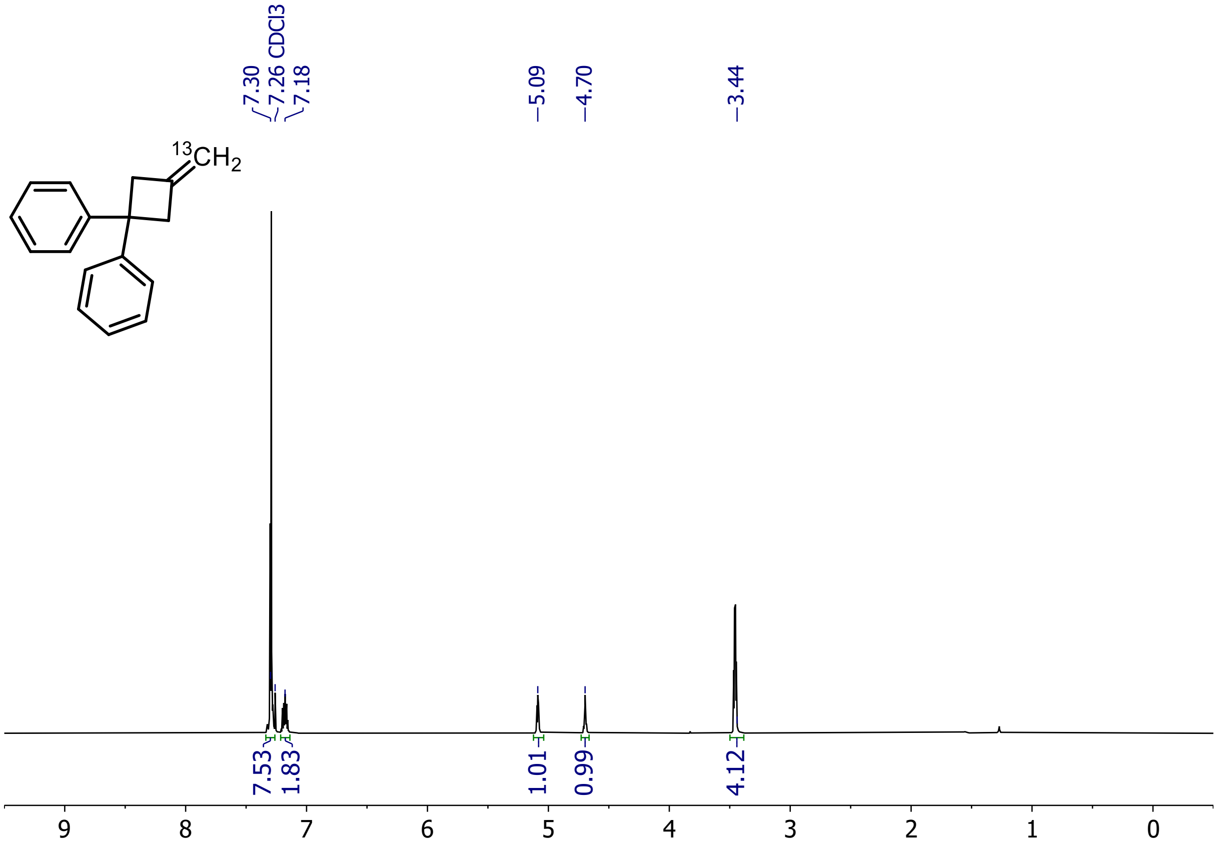

[13C-1a], ^{13}C, $CDCl_3$, 101 MHz

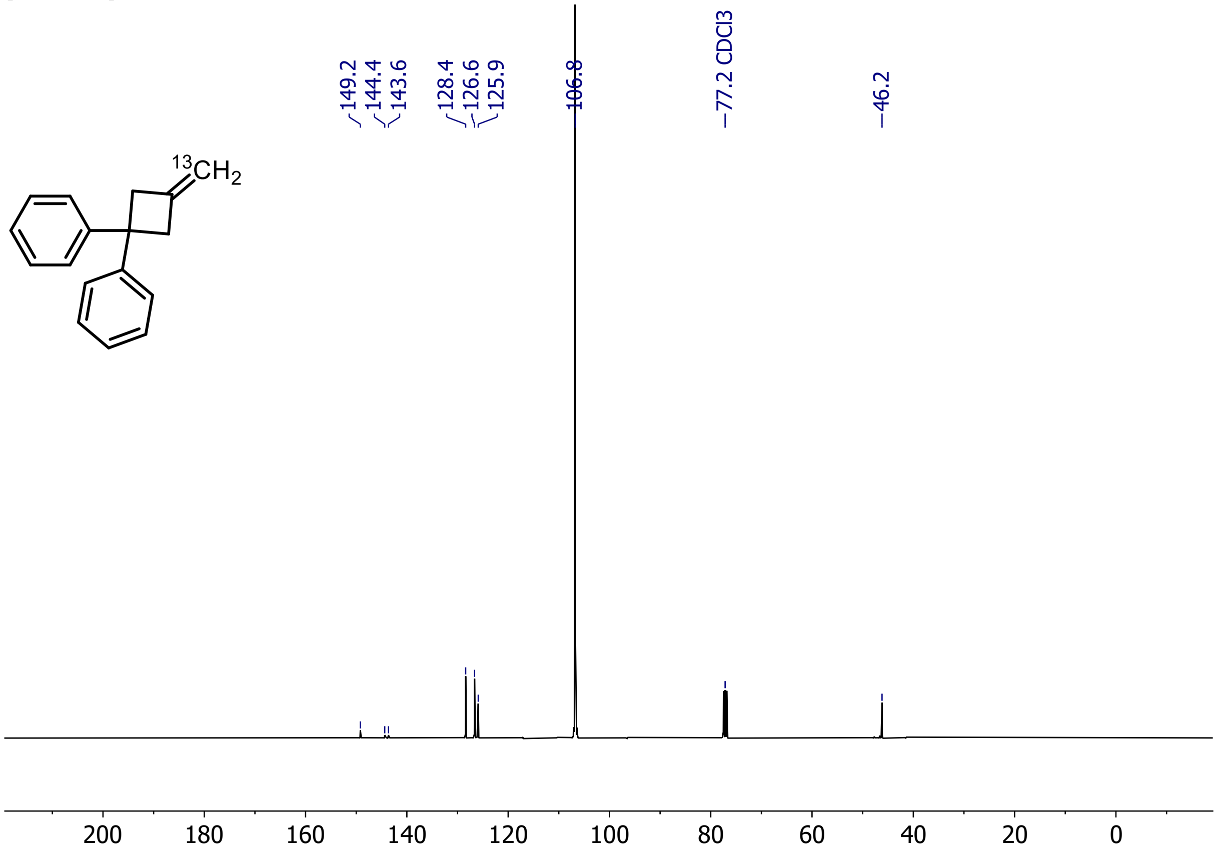

[^{13}C-2a], ^{1}H, $CDCl_3$, 400 MHz

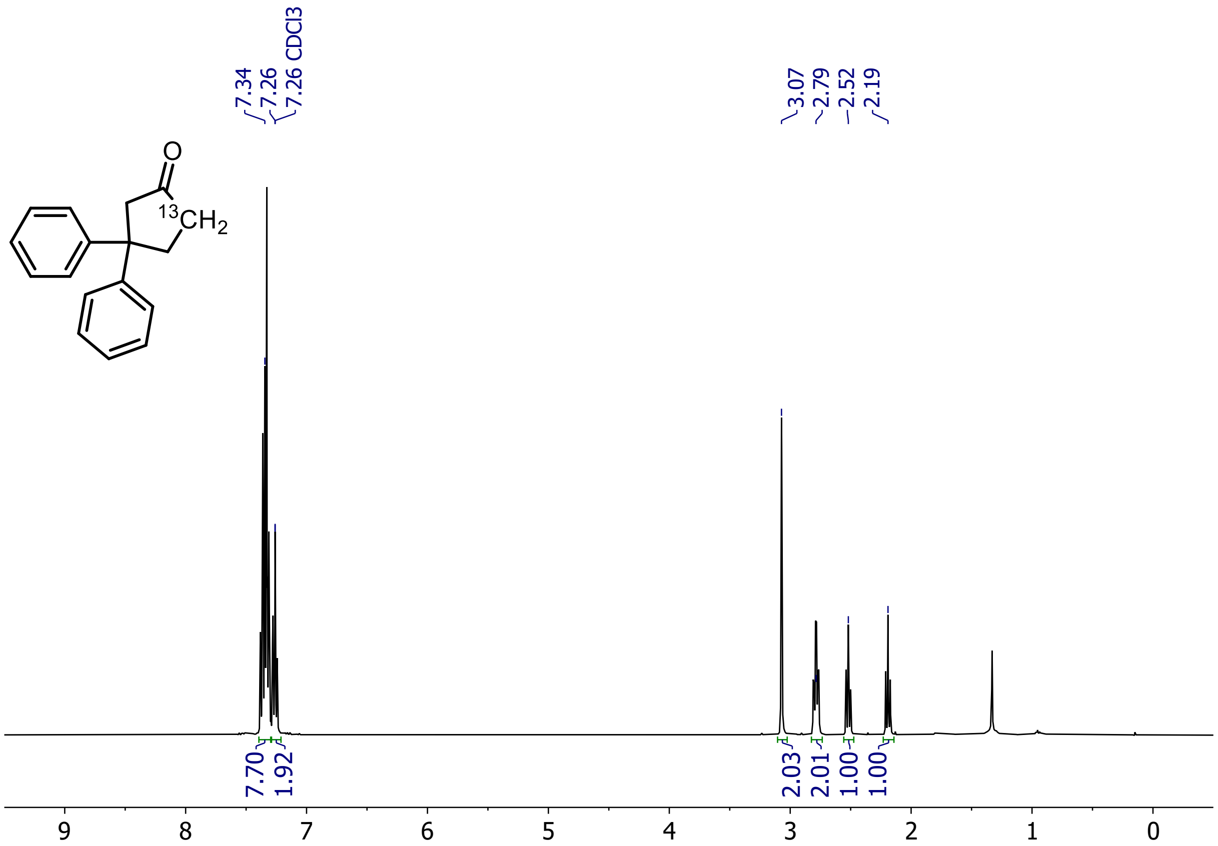

[^{13}C-2a], ^{13}C, $CDCl_3$, 101 MHz

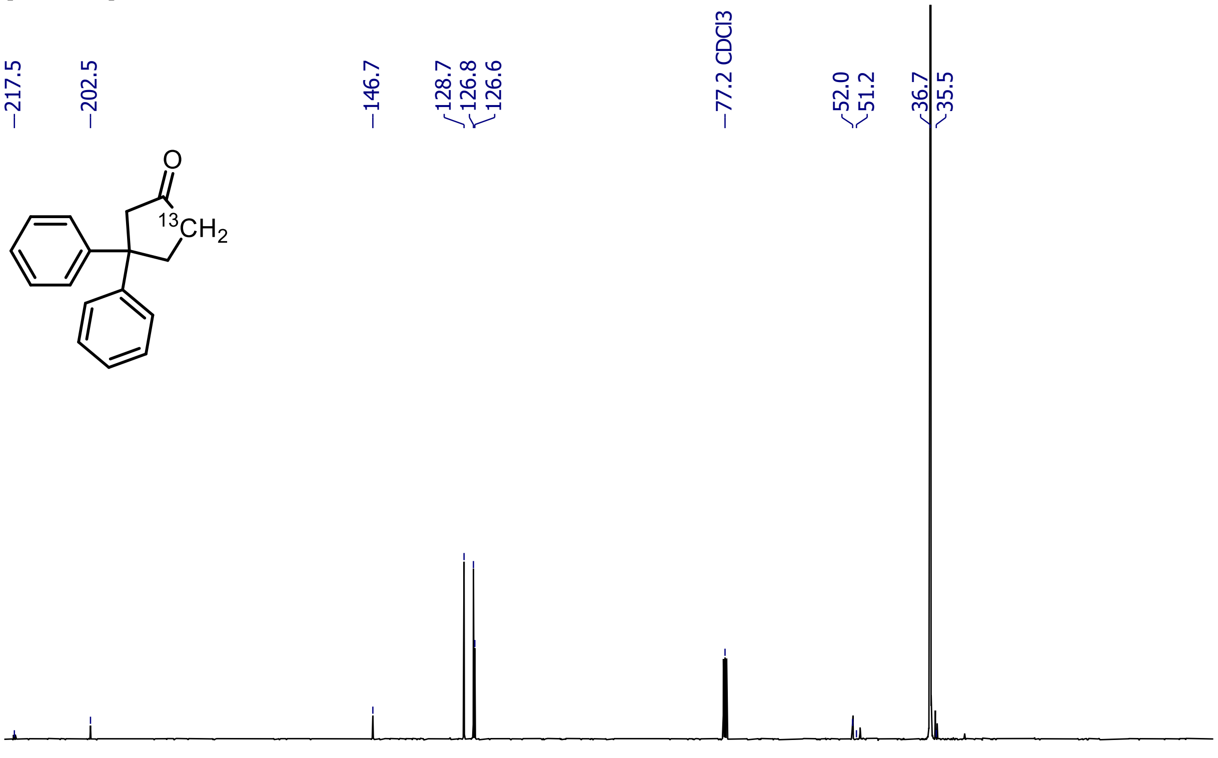

[2a], ^{1}H, $CDCl_3$, 599 MHz

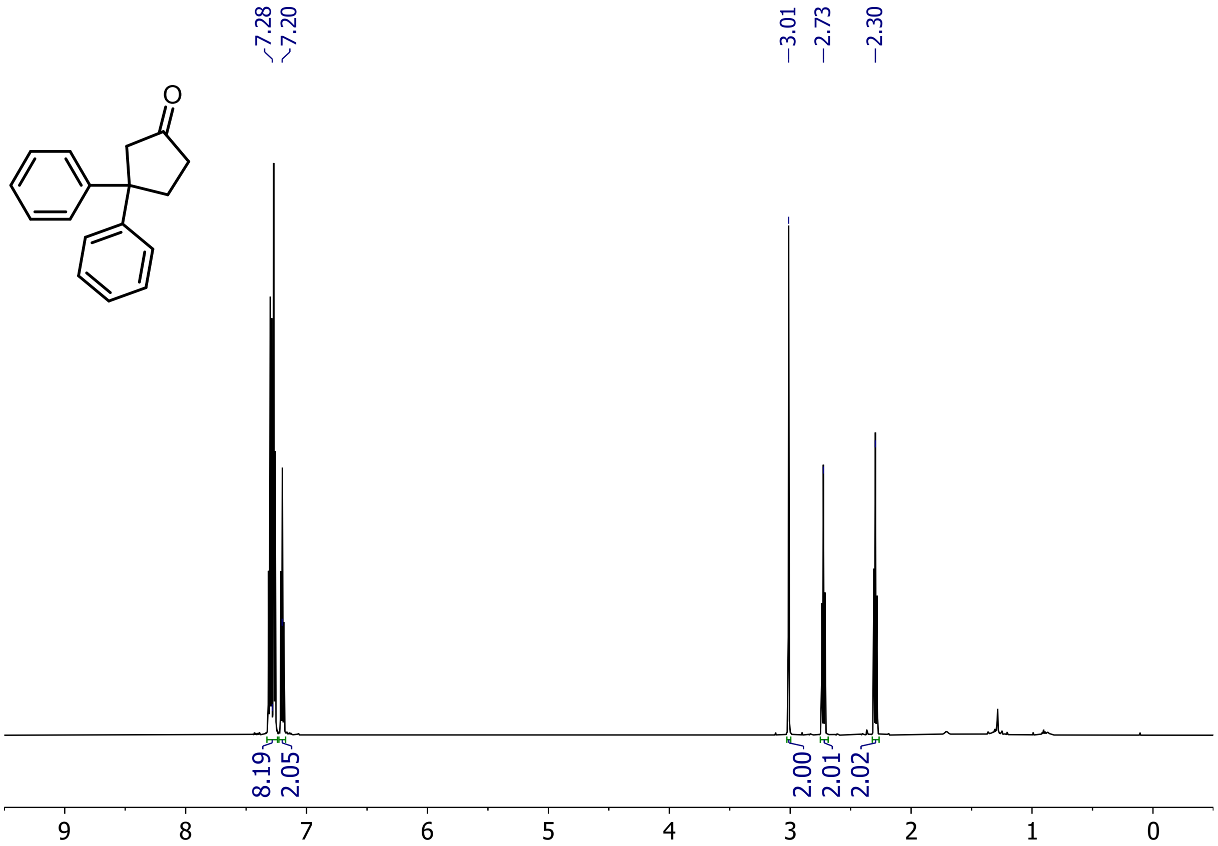

[2a], ^{13}C, $CDCl_3$, 151 MHz

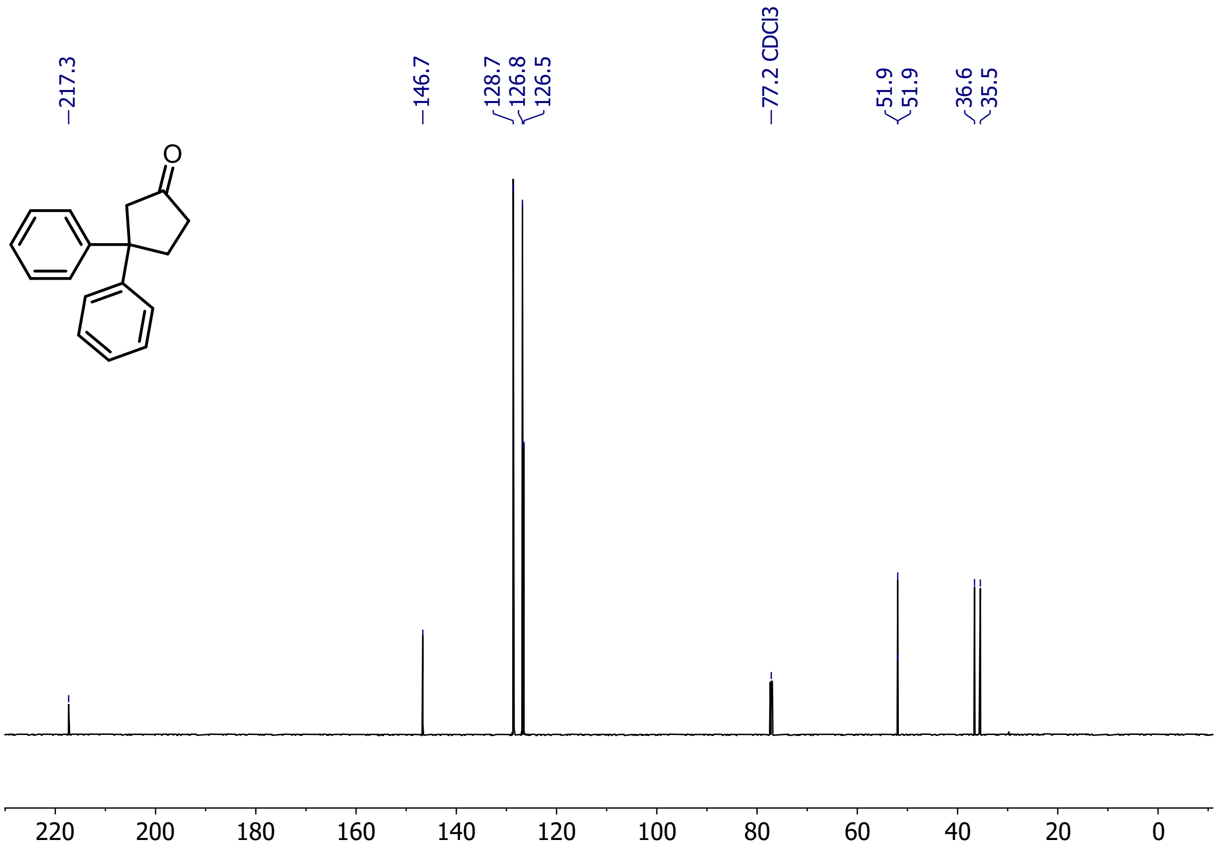

[2b], ^{1}H, $CDCl_3$, 400 MHz

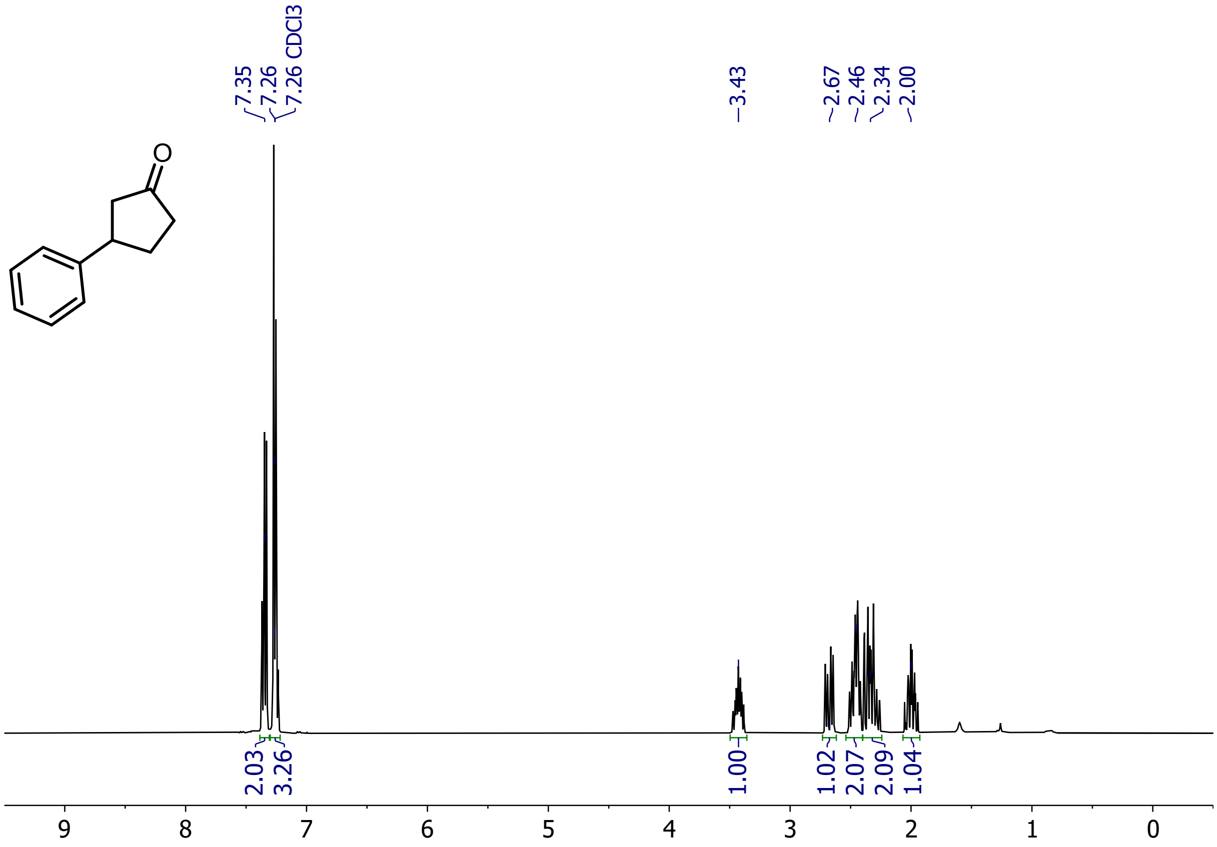

[2b], ^{13}C, $CDCl_3$, 101 MHz

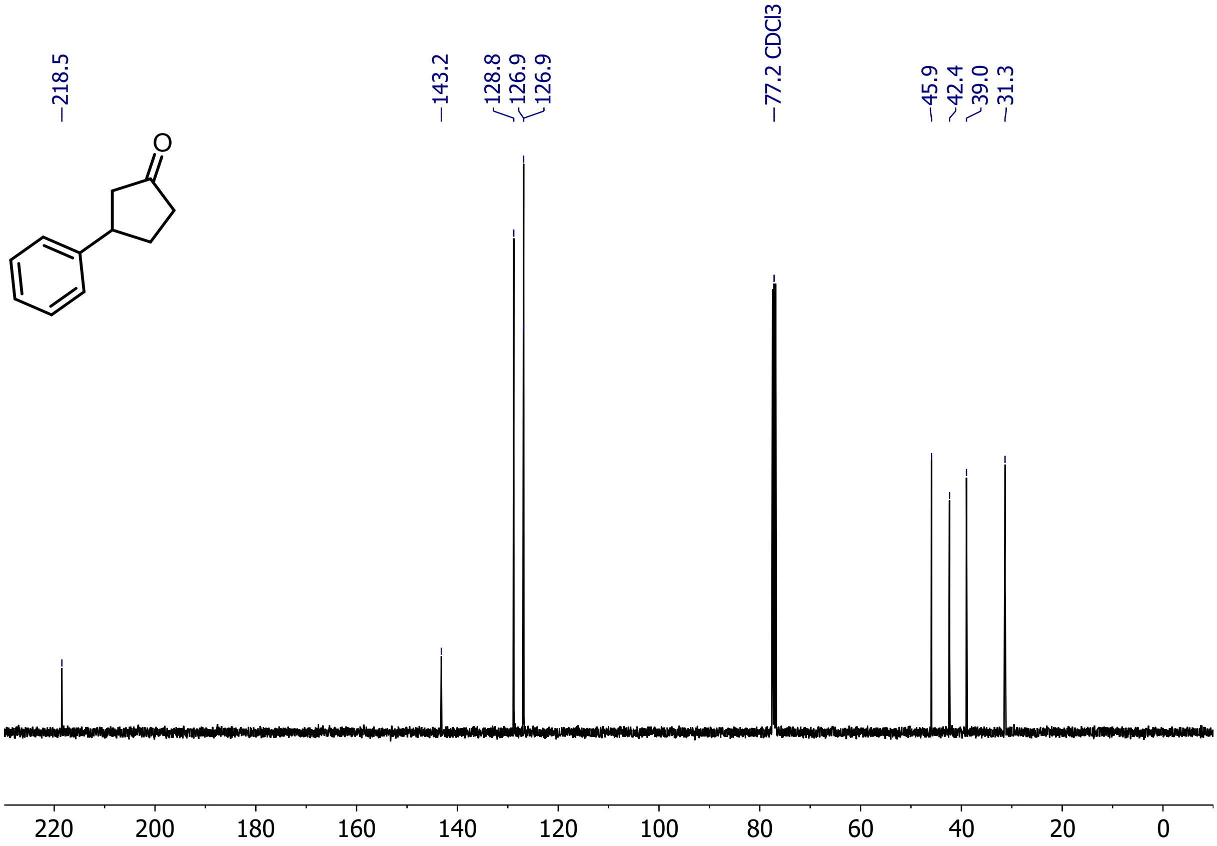

[2c], ^{1}H, $CDCl_3$, 400 MHz

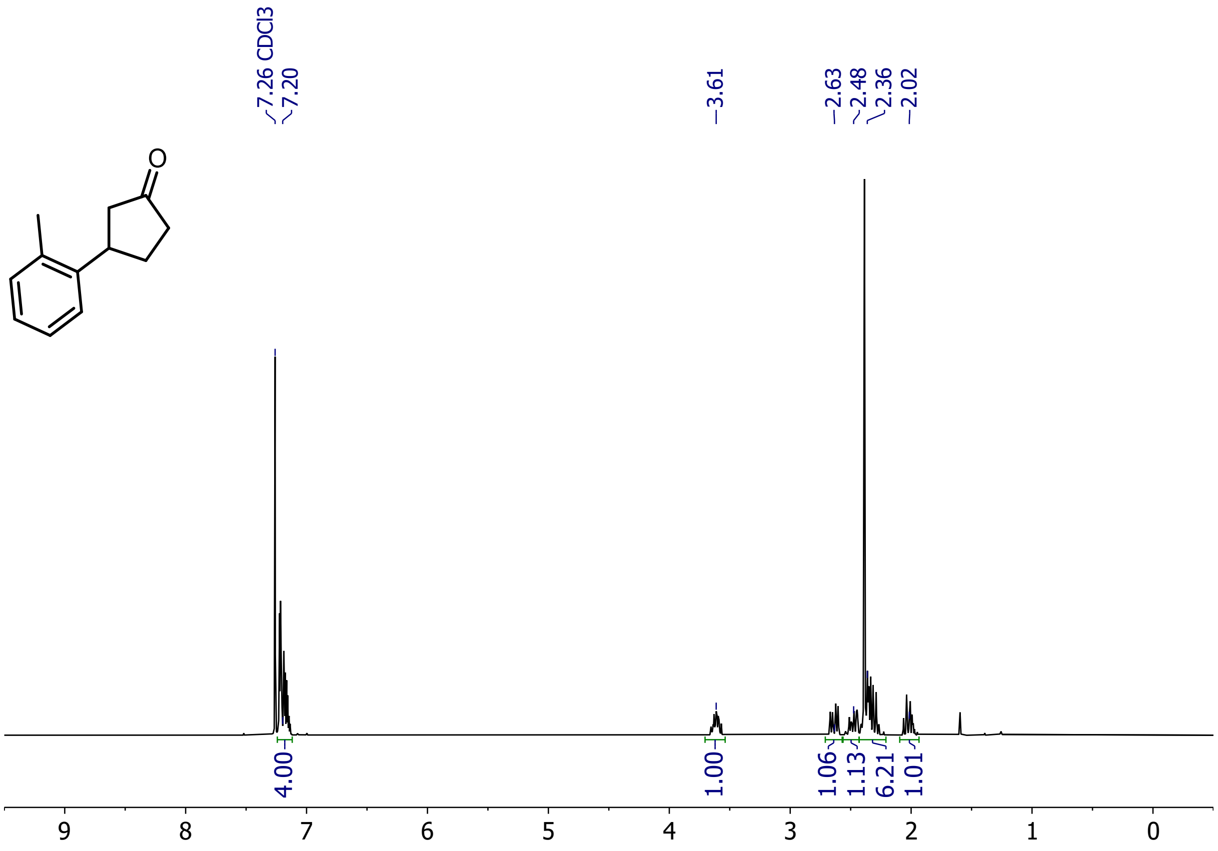

[2c], ^{13}C, $CDCl_3$, 101 MHz

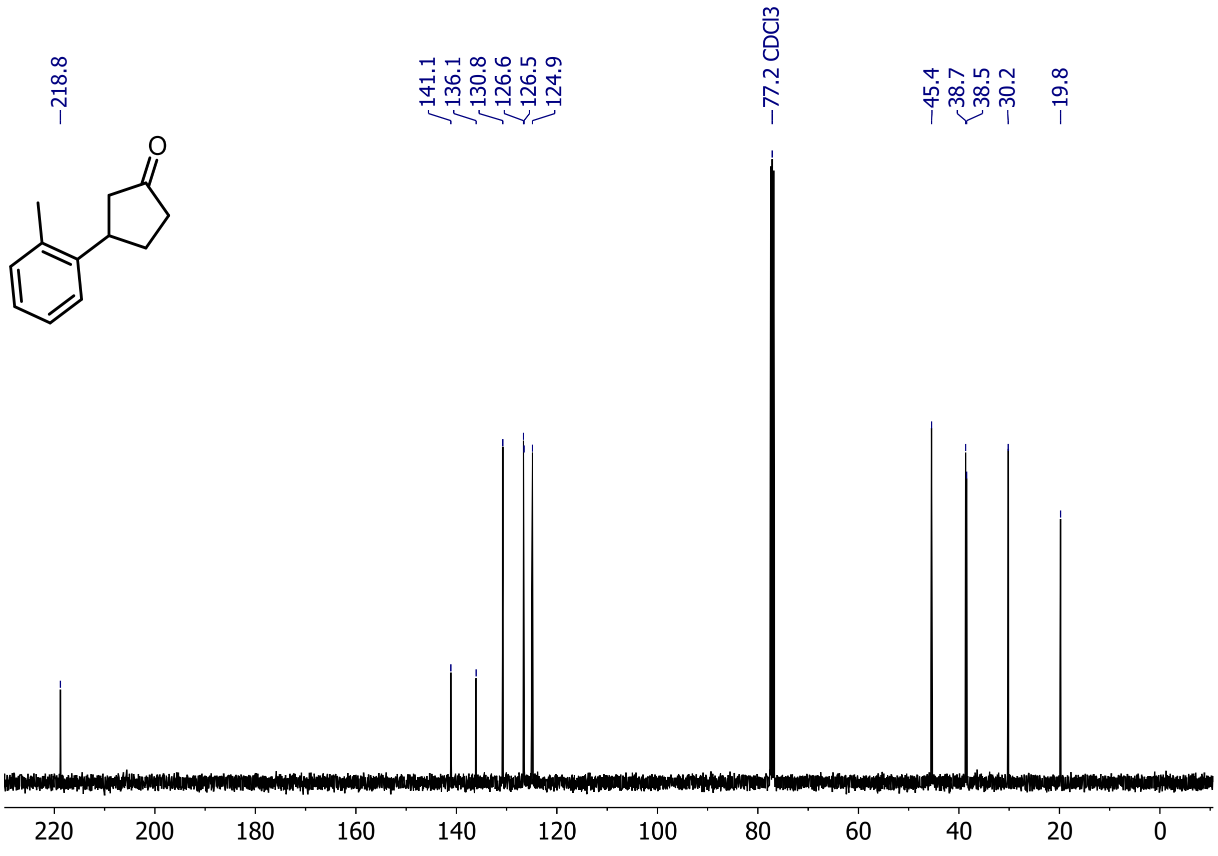

[2d], ^{1}H, $CDCl_3$, 400 MHz

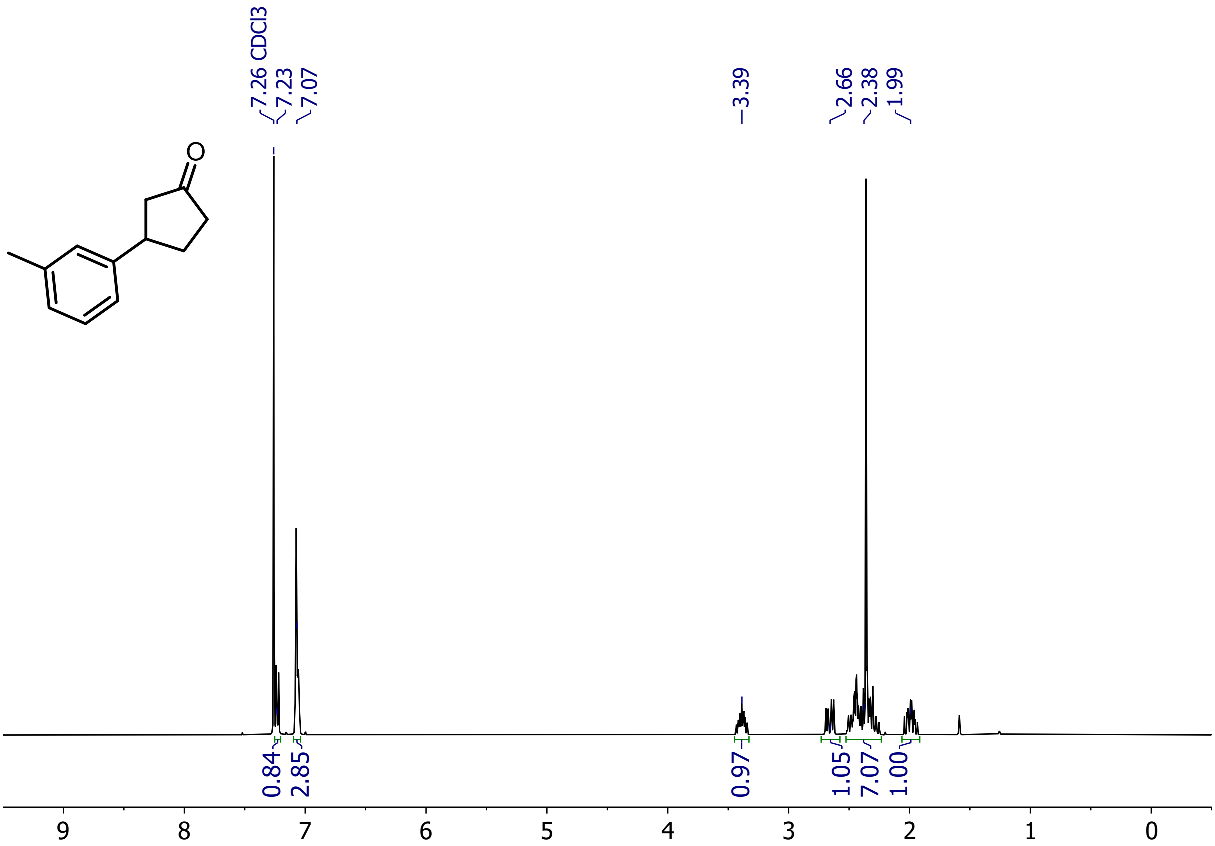

[2d], ^{13}C, $CDCl_3$, 101 MHz

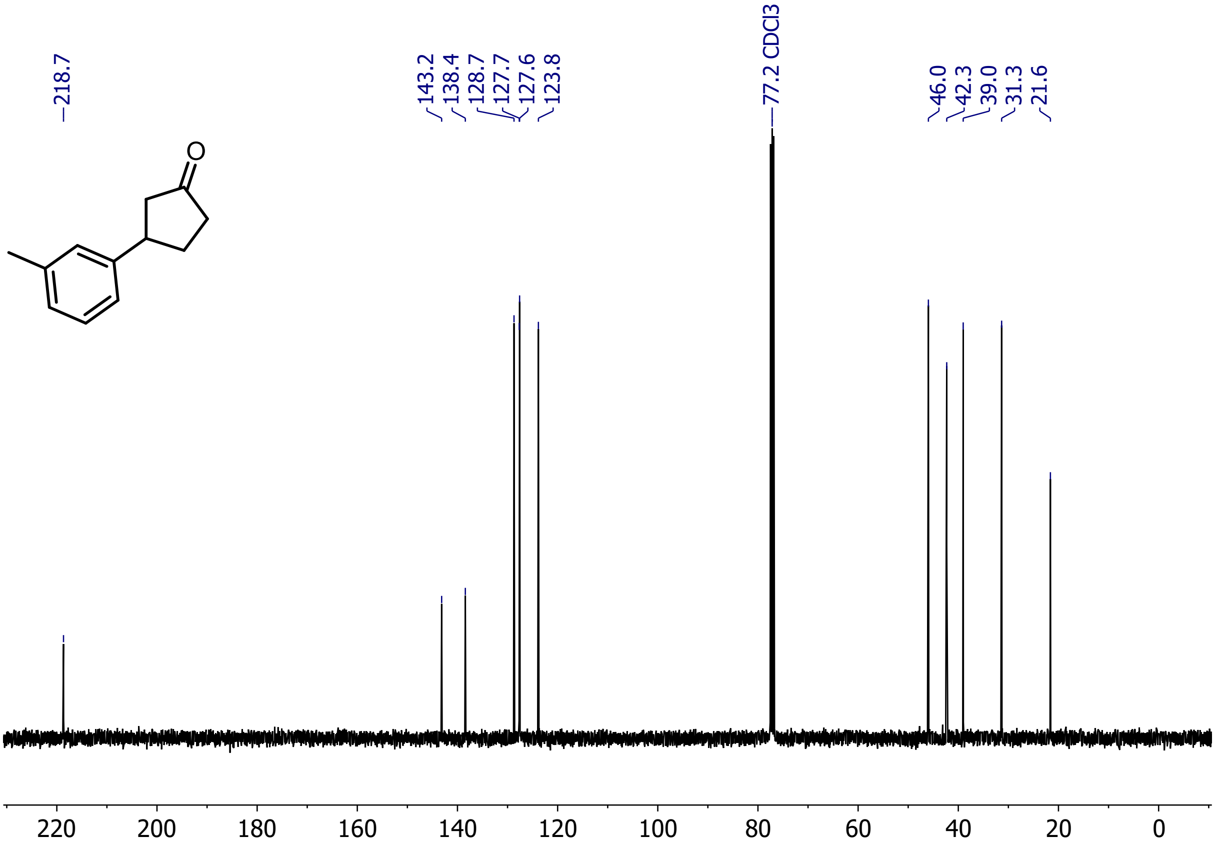

[2e], ^{1}H, $CDCl_3$, 400 MHz

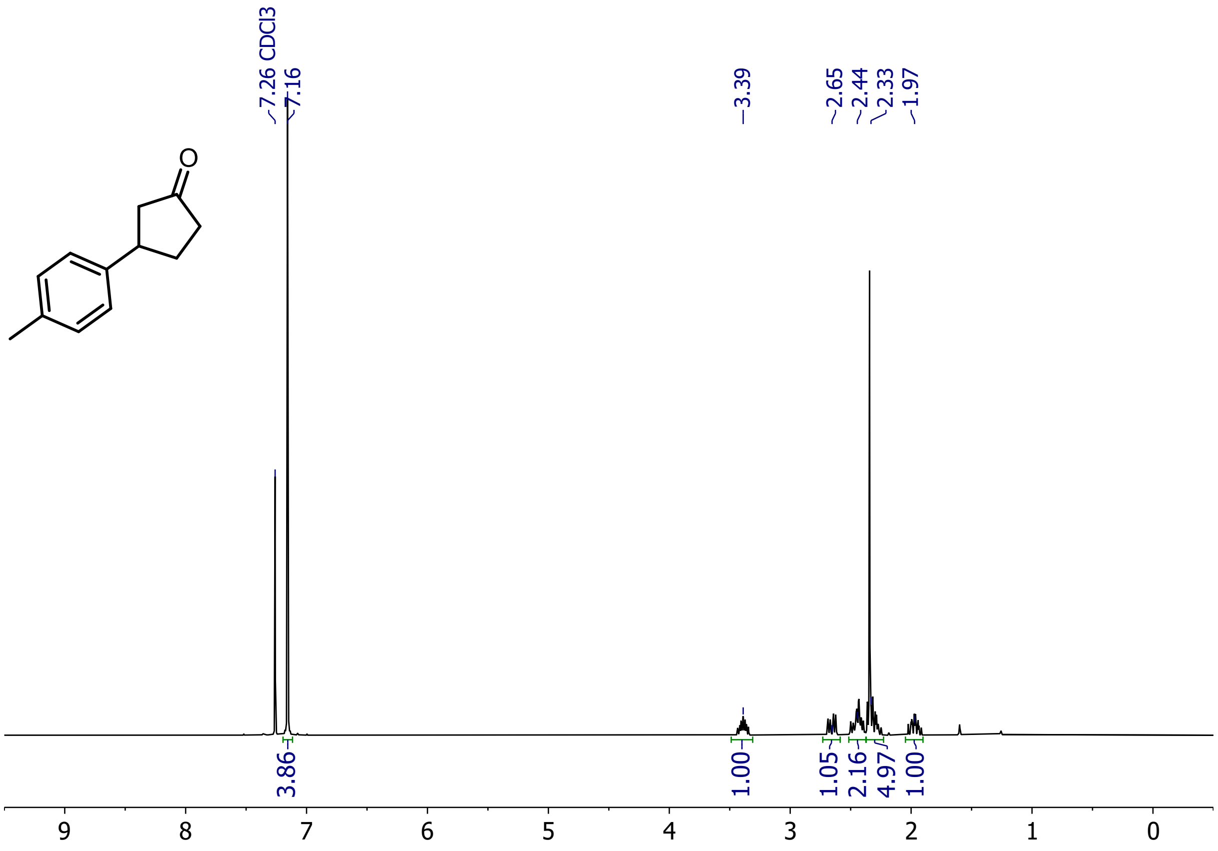

[2e], ^{13}C, $CDCl_3$, 101 MHz

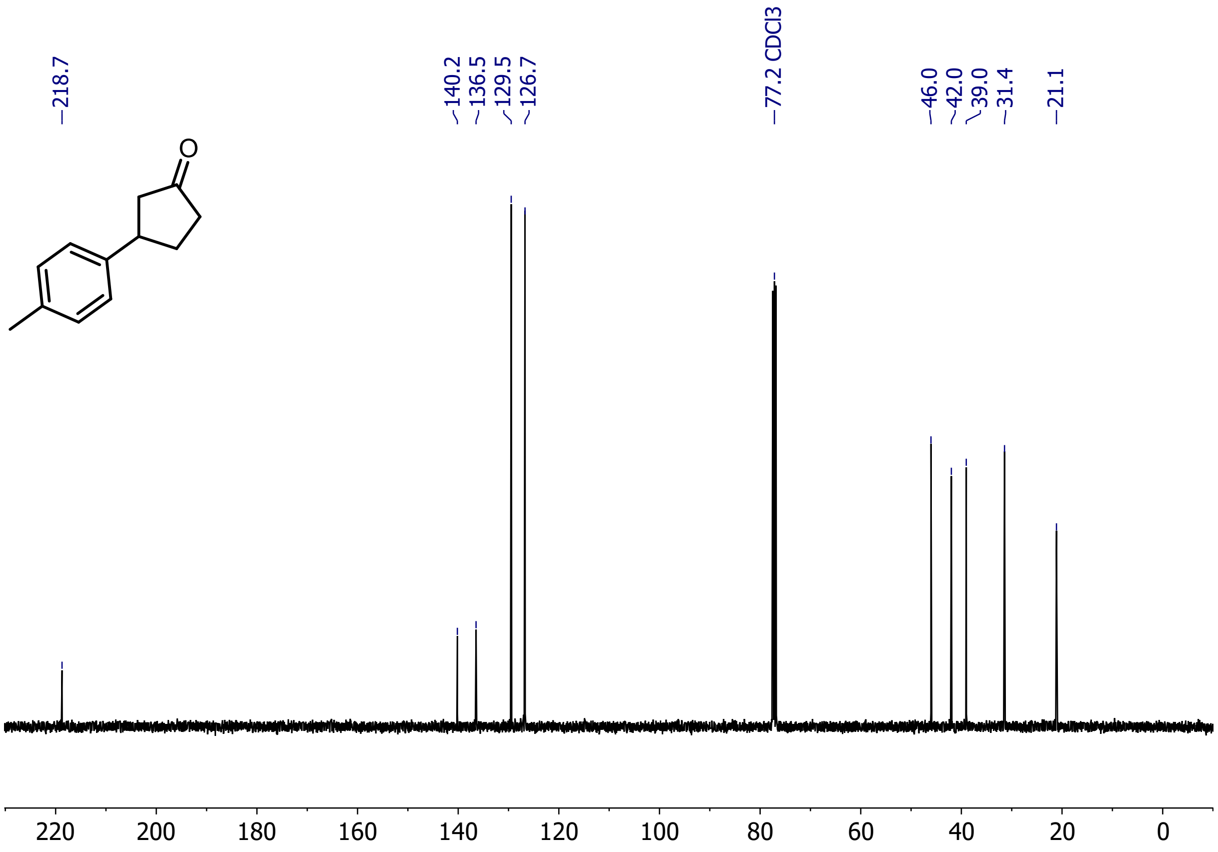

[2f], ^{1}H, $CDCl_3$, 400 MHz

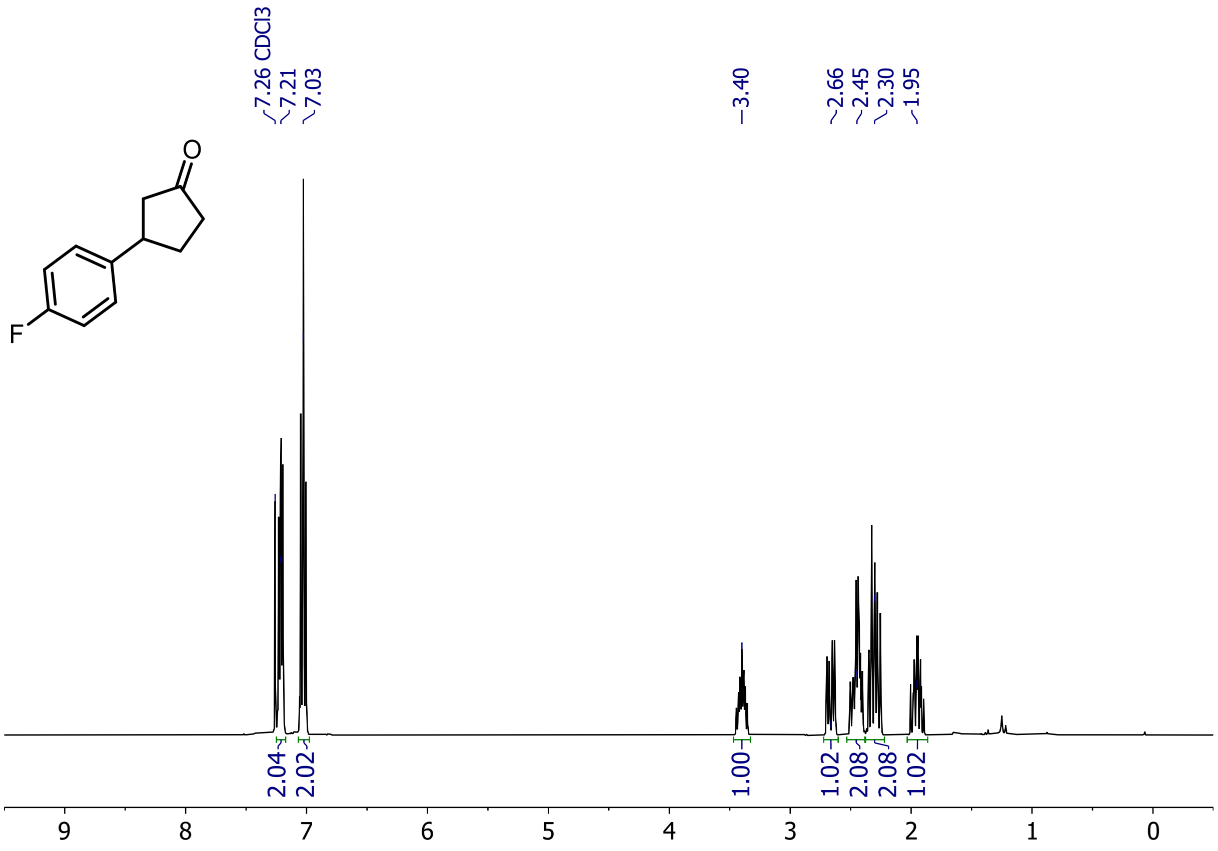

[2f], ^{13}C, $CDCl_3$, 101 MHz

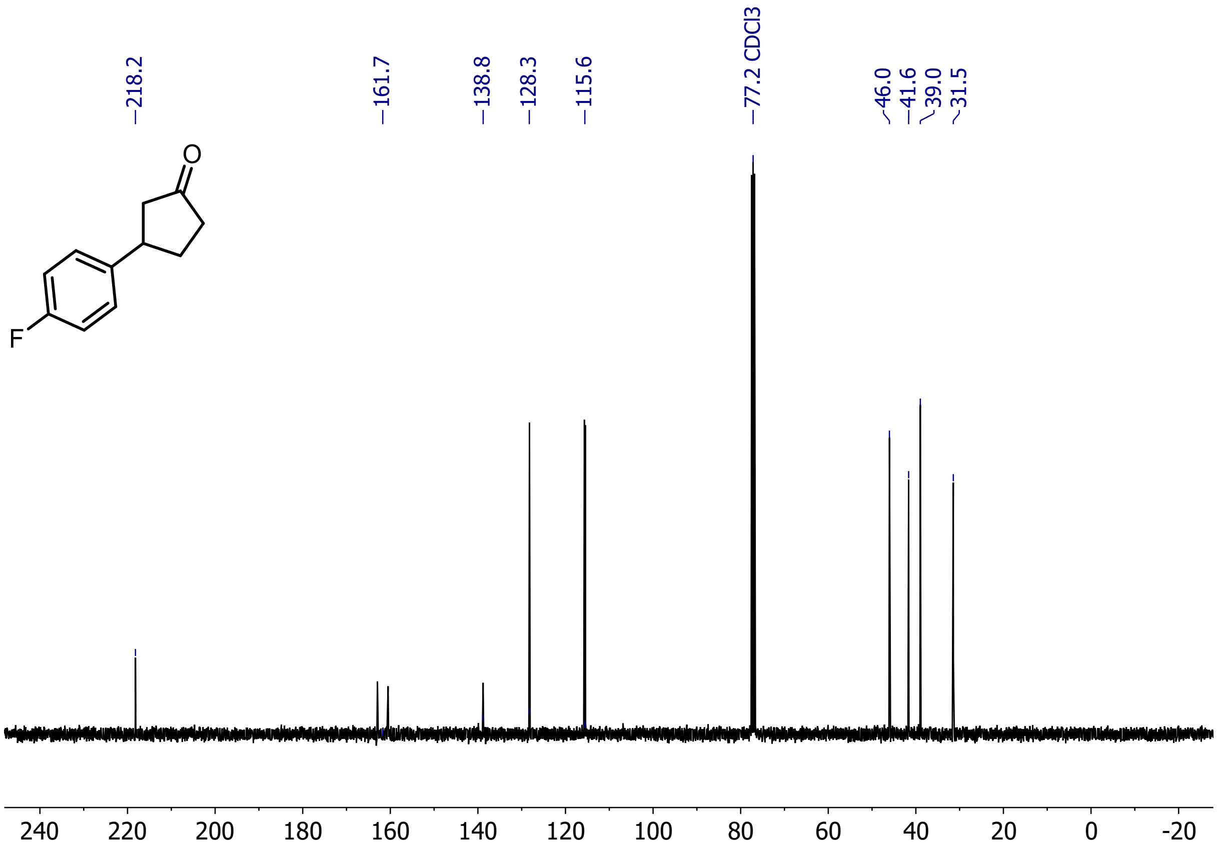

[2f], ^{19}F, $CDCl_3$, 377 MHz

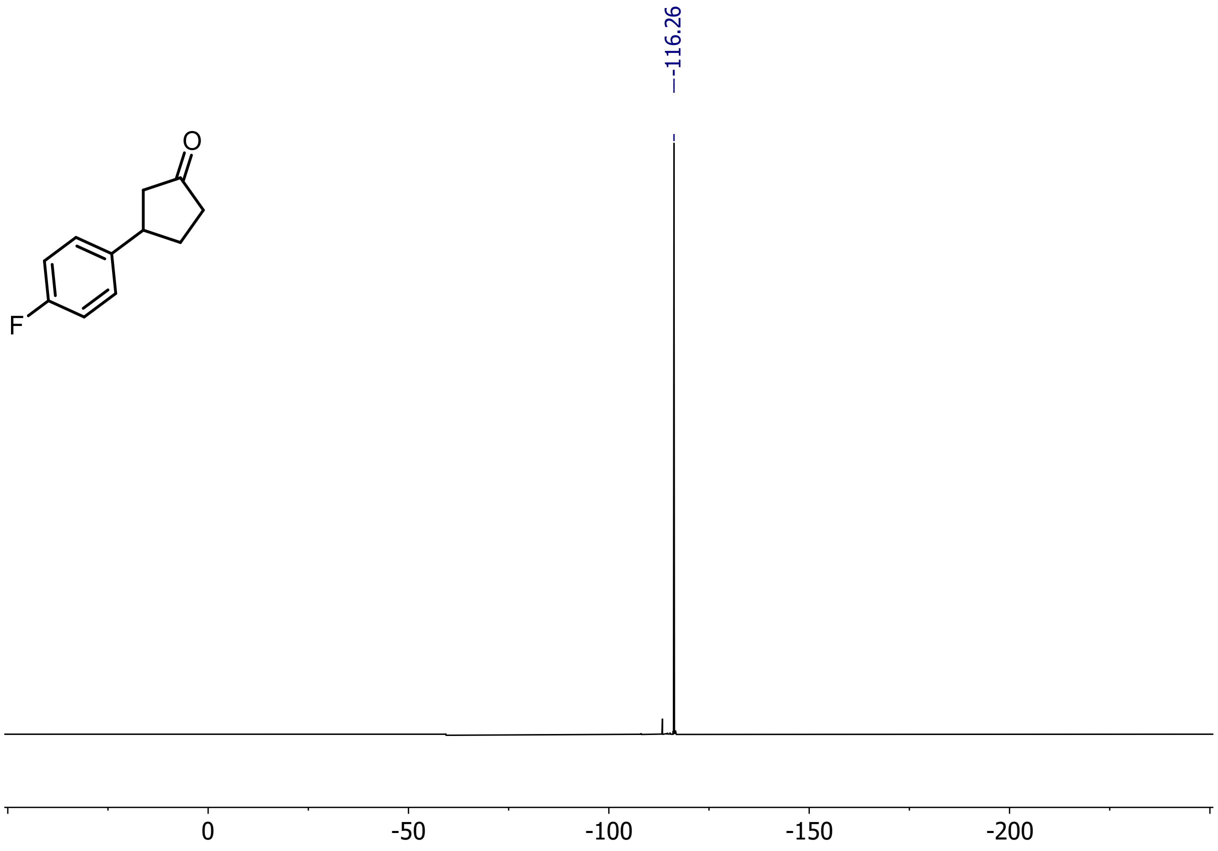

[2g], ^{1}H, $CDCl_3$, 400 MHz

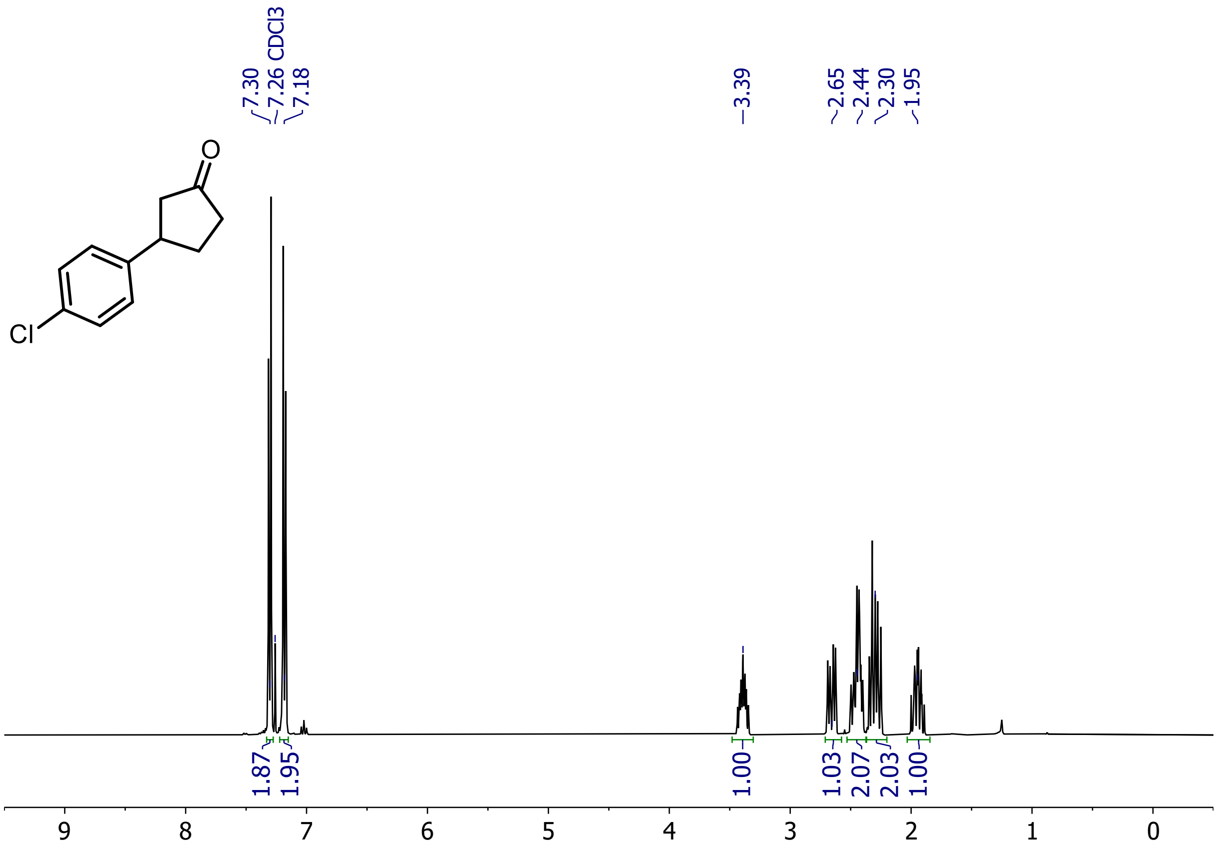

[2g], ^{13}C, $CDCl_3$, 101 MHz

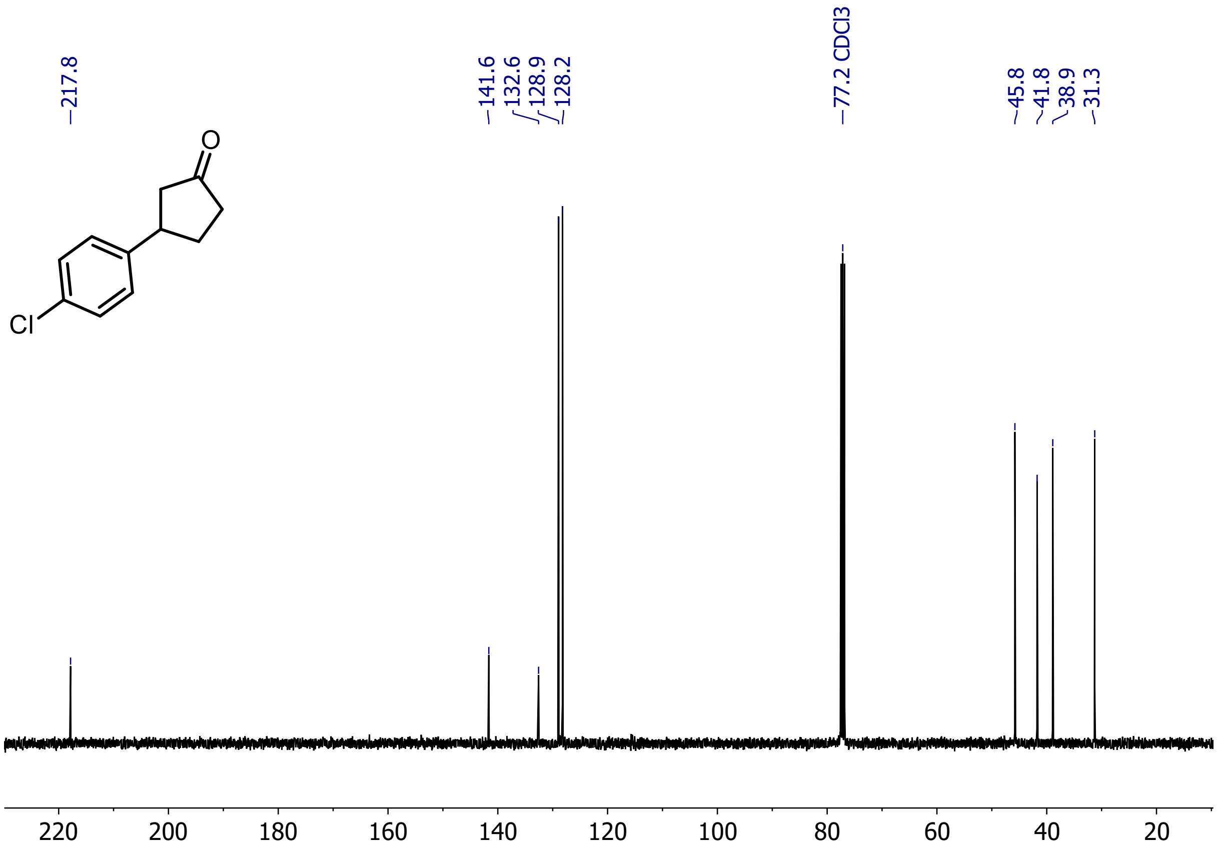

[2h], ^{1}H, $CDCl_3$, 400 MHz

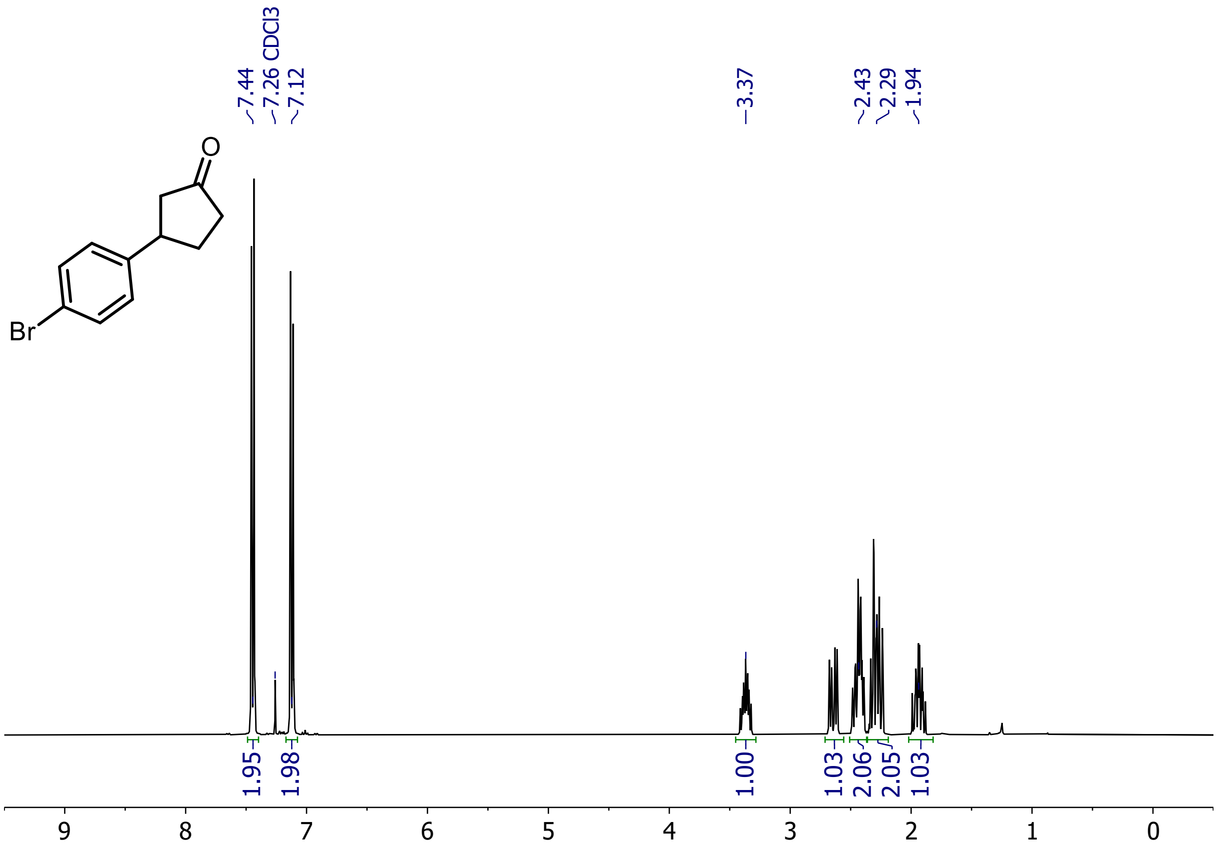

[2h], ^{13}C, $CDCl_3$, 101 MHz

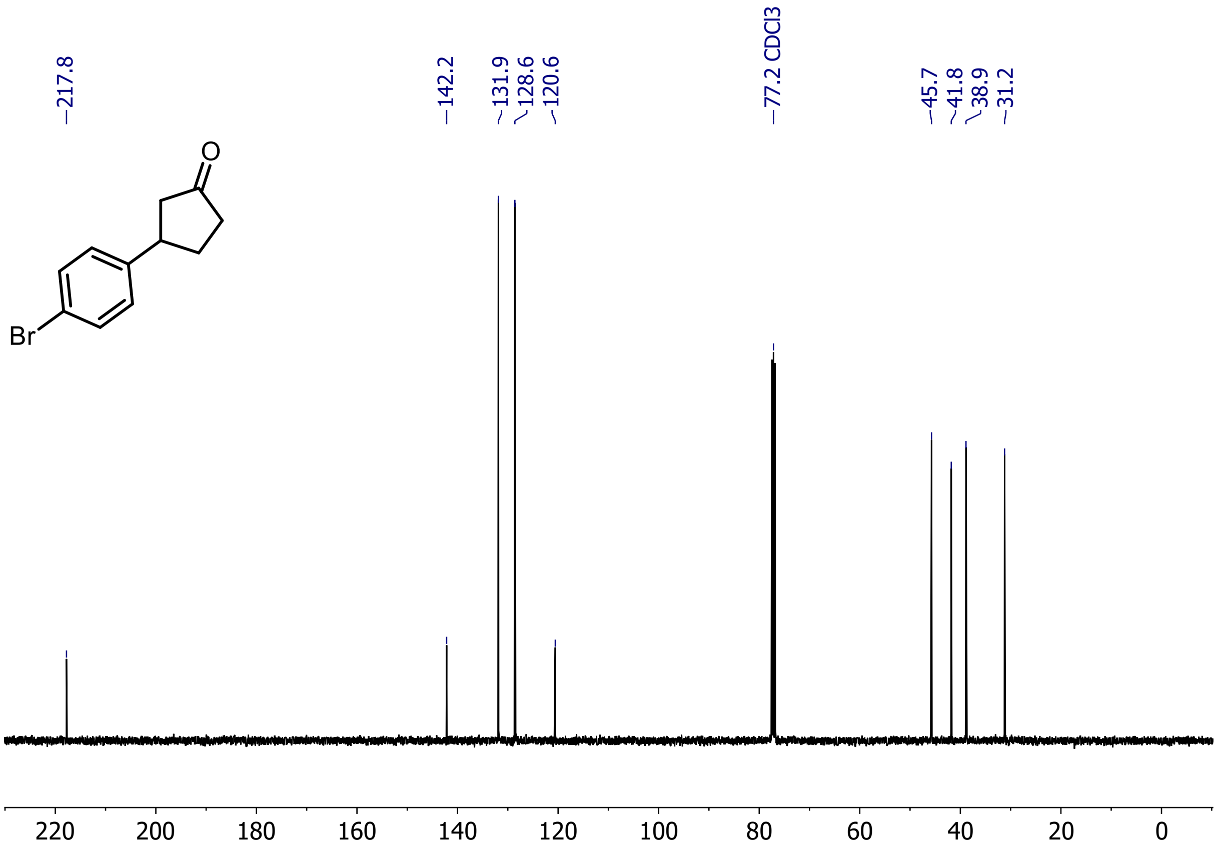

[2i], ^{1}H, $CDCl_3$, 599 MHz

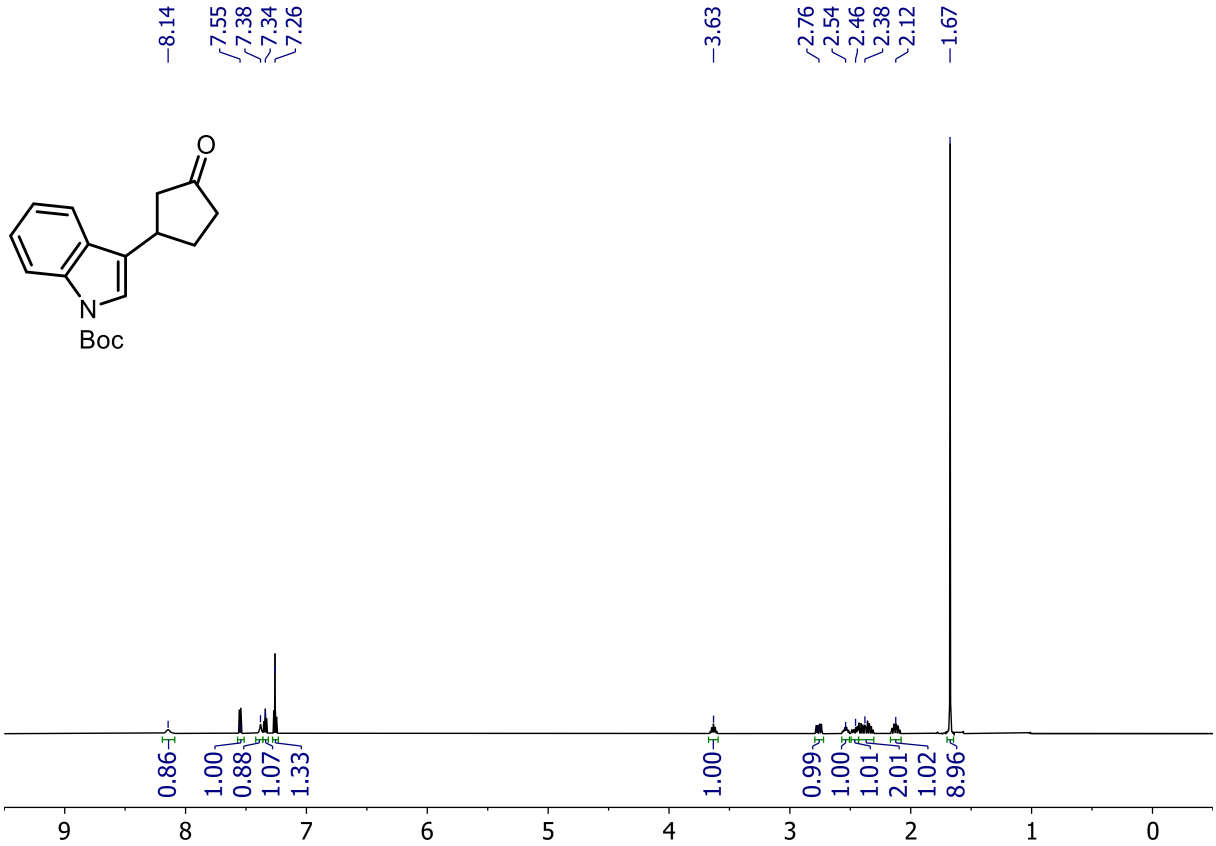

[2i], ^{13}C, $CDCl_3$, 151 MHz

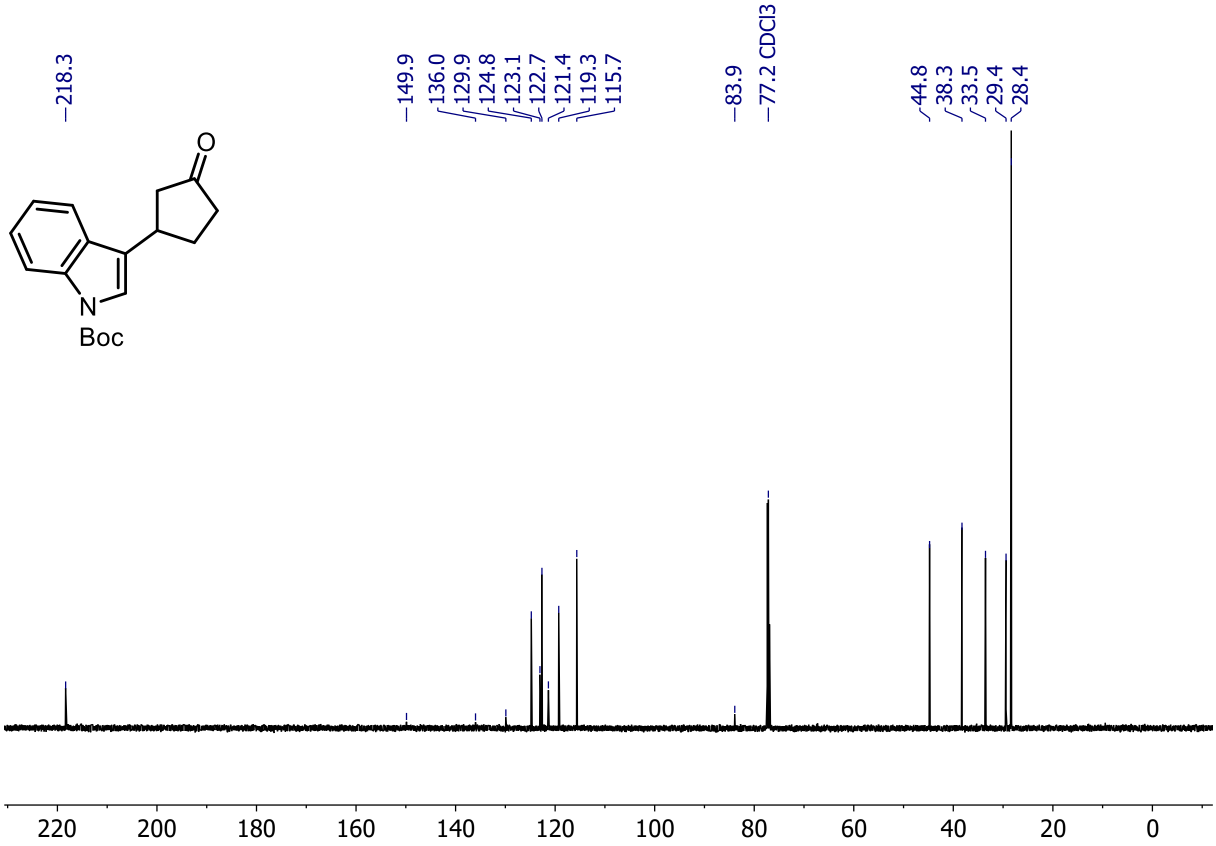

[2j], ^{1}H, $CDCl_3$, 400 MHz

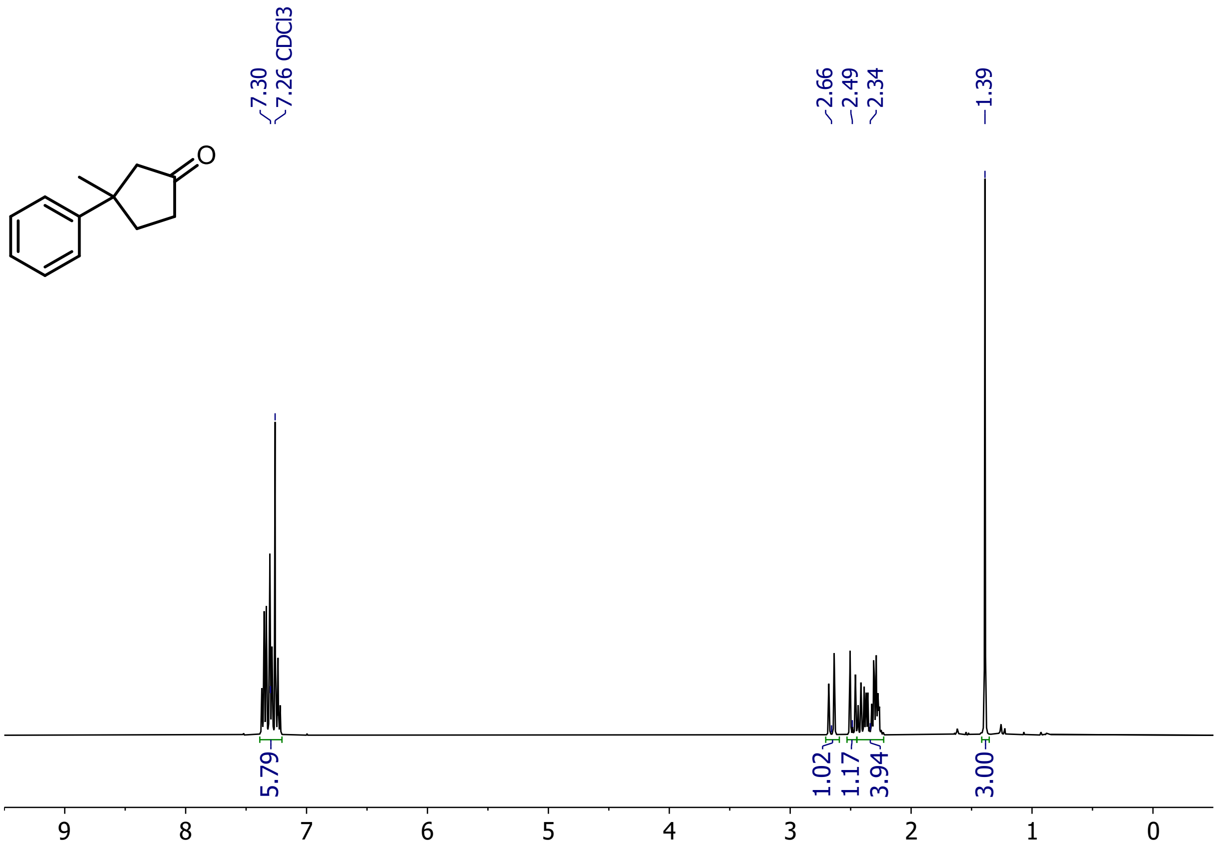

[2j], ^{13}C, $CDCl_3$, 101 MHz

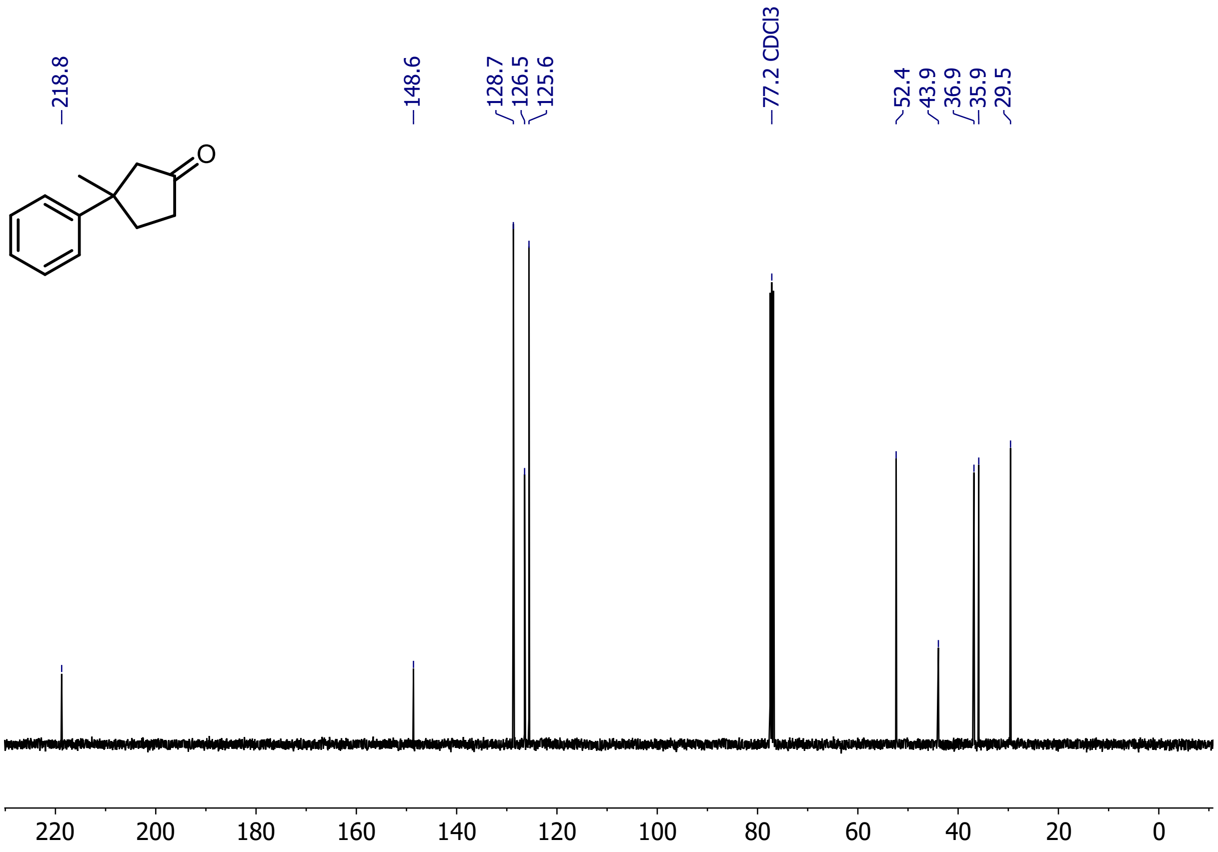

[2k], ^{1}H, $CDCl_3$, 599 MHz

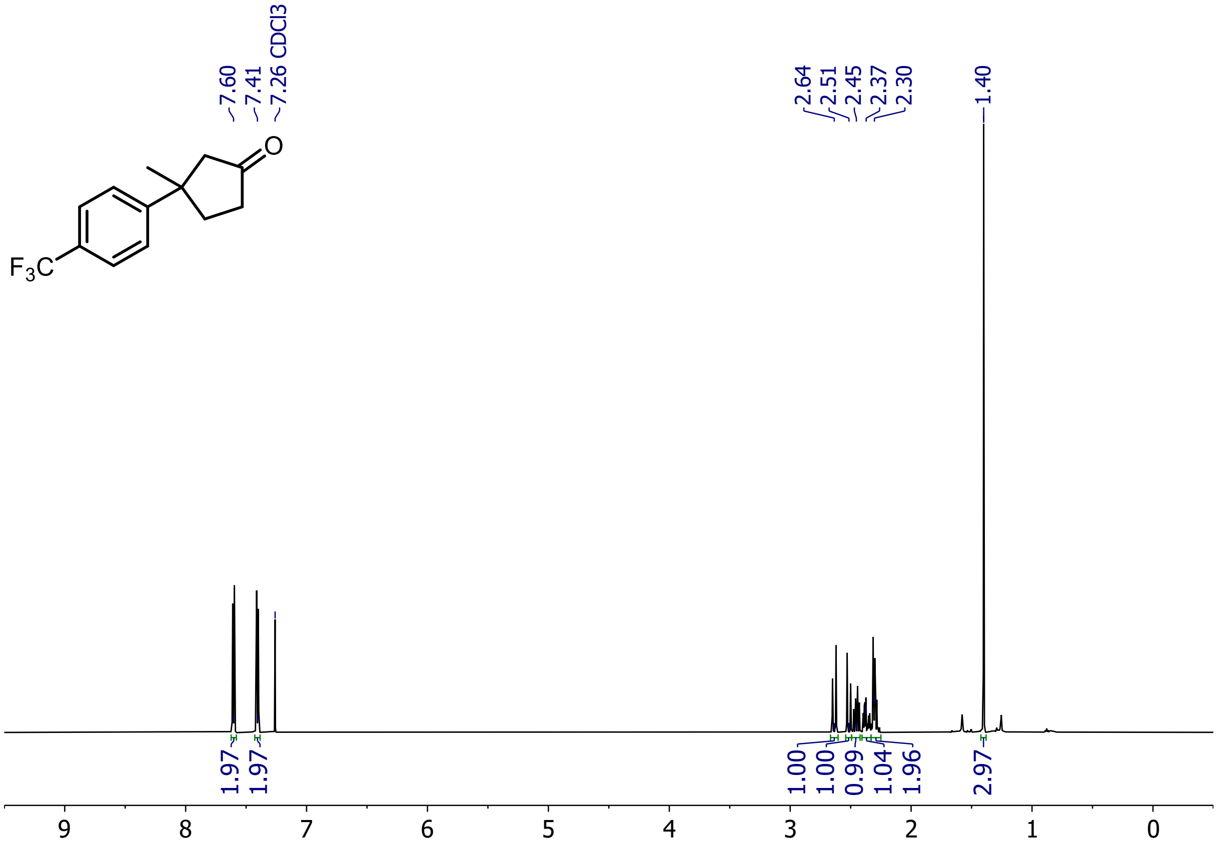

[2k], ^{13}C, $CDCl_3$, 151 MHz

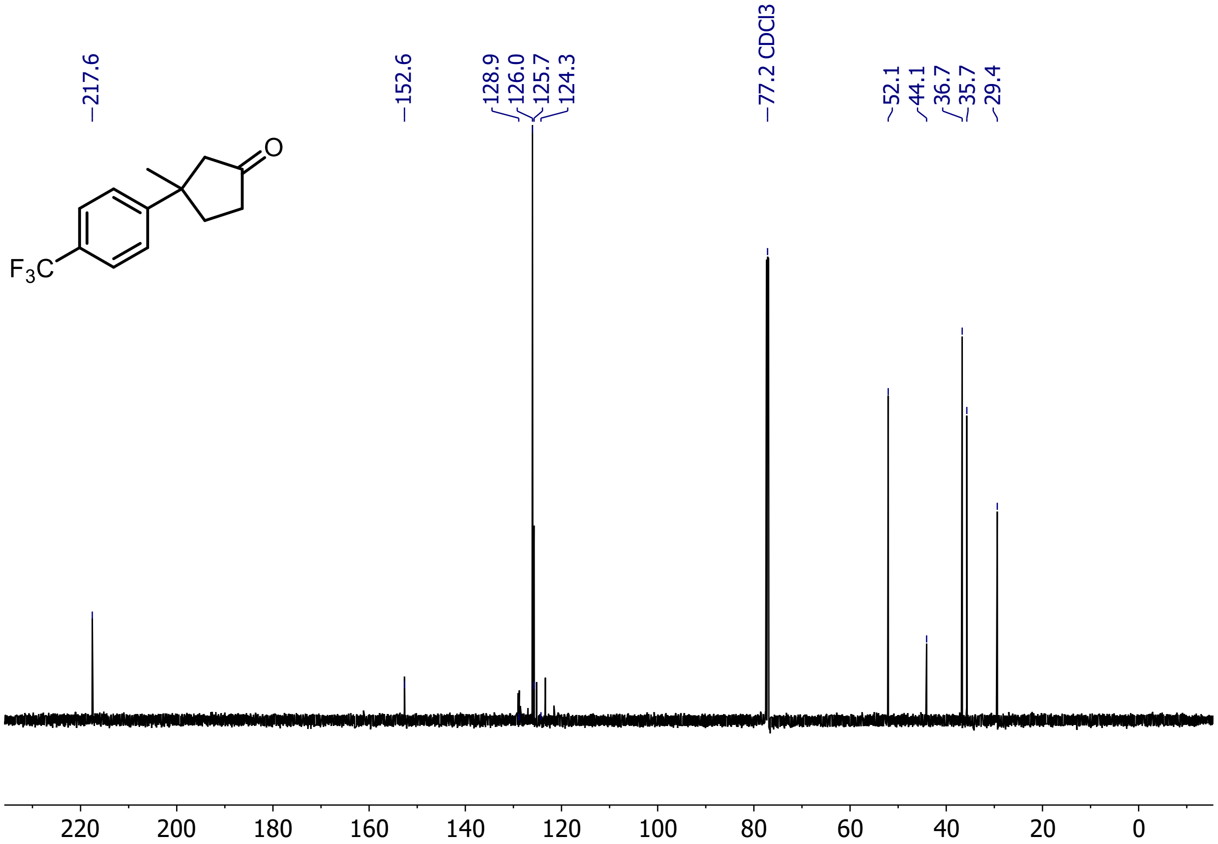

[2k], ^{19}F, $CDCl_3$, 564 MHz

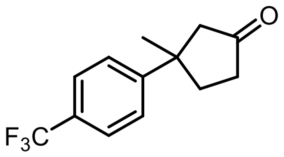

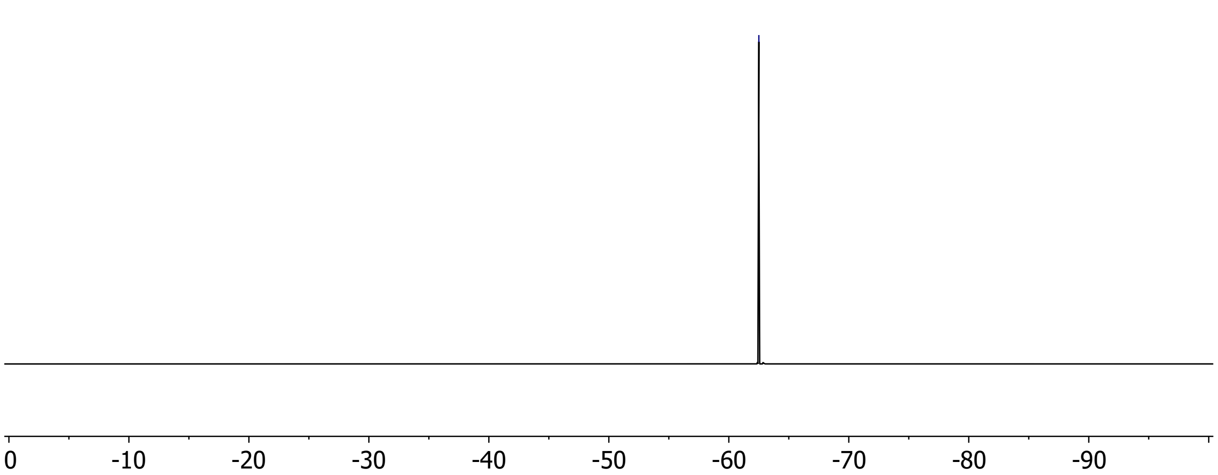

[2l], ^{1}H, $CDCl_3$, 400 MHz

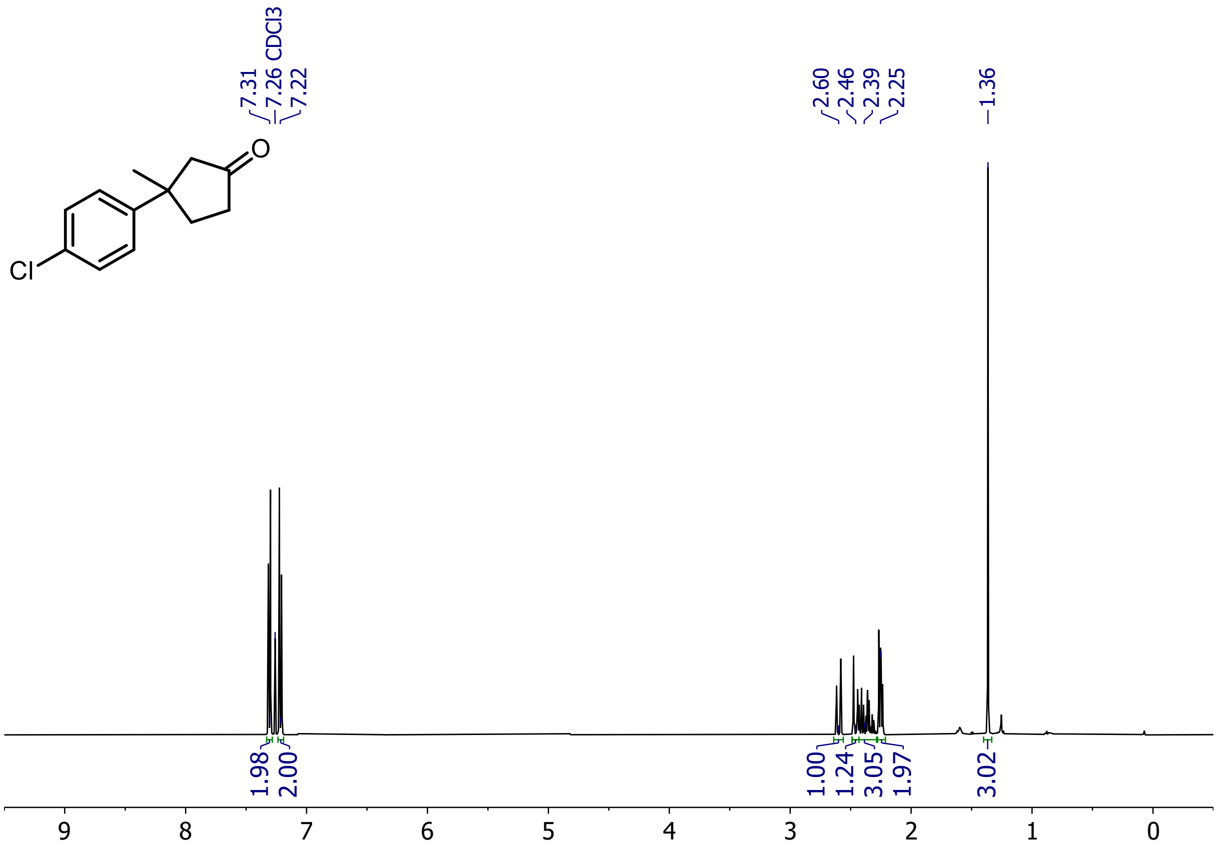

[2l], ^{13}C, $CDCl_3$, 101 MHz

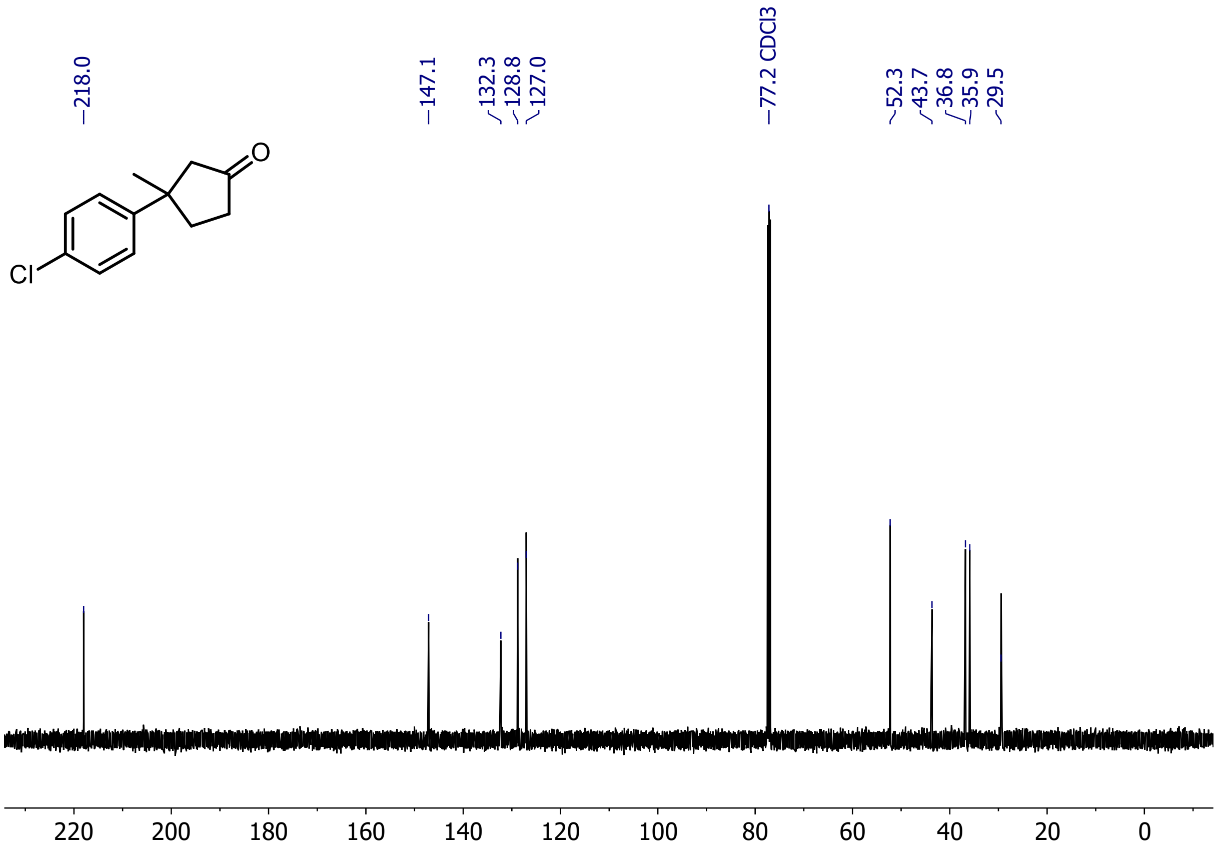

[2m], ^{1}H, $CDCl_3$, 599 MHz

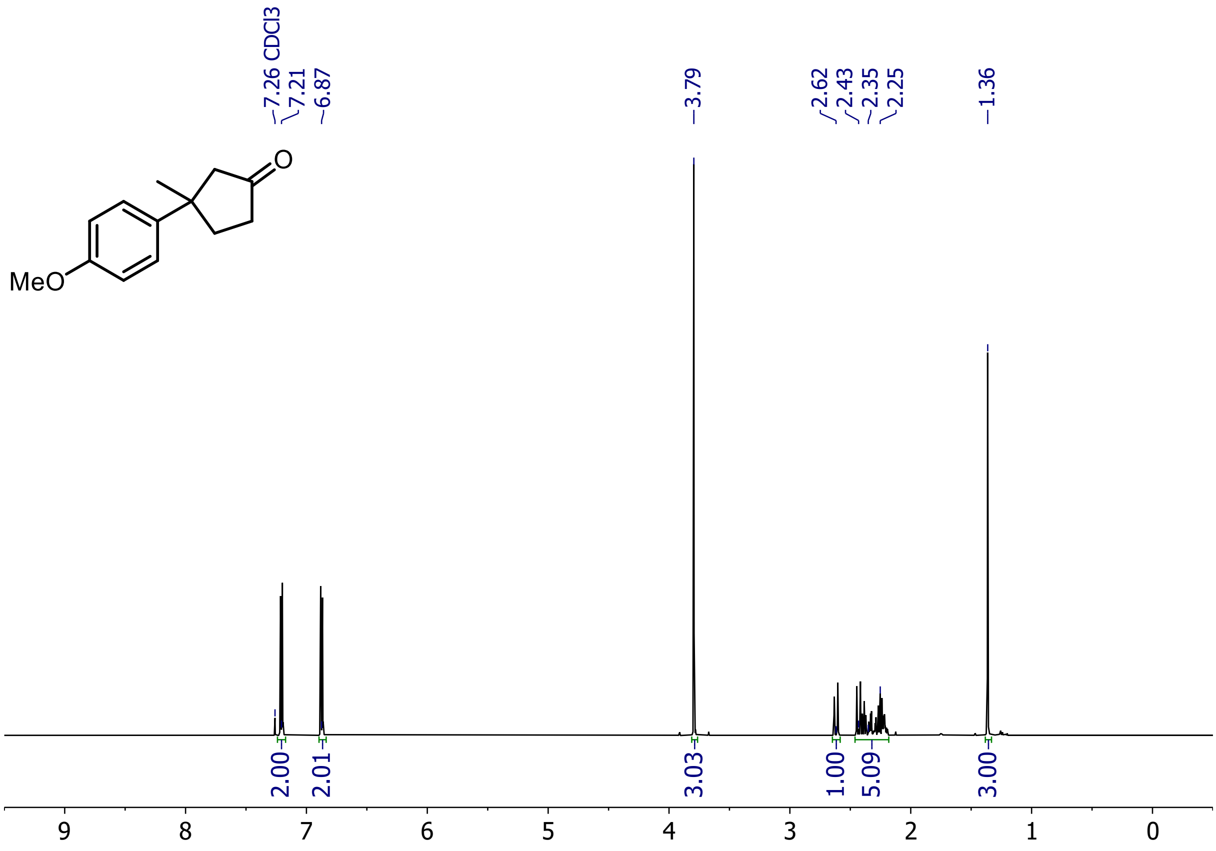

[2m], ^{13}C, $CDCl_3$, 151 MHz

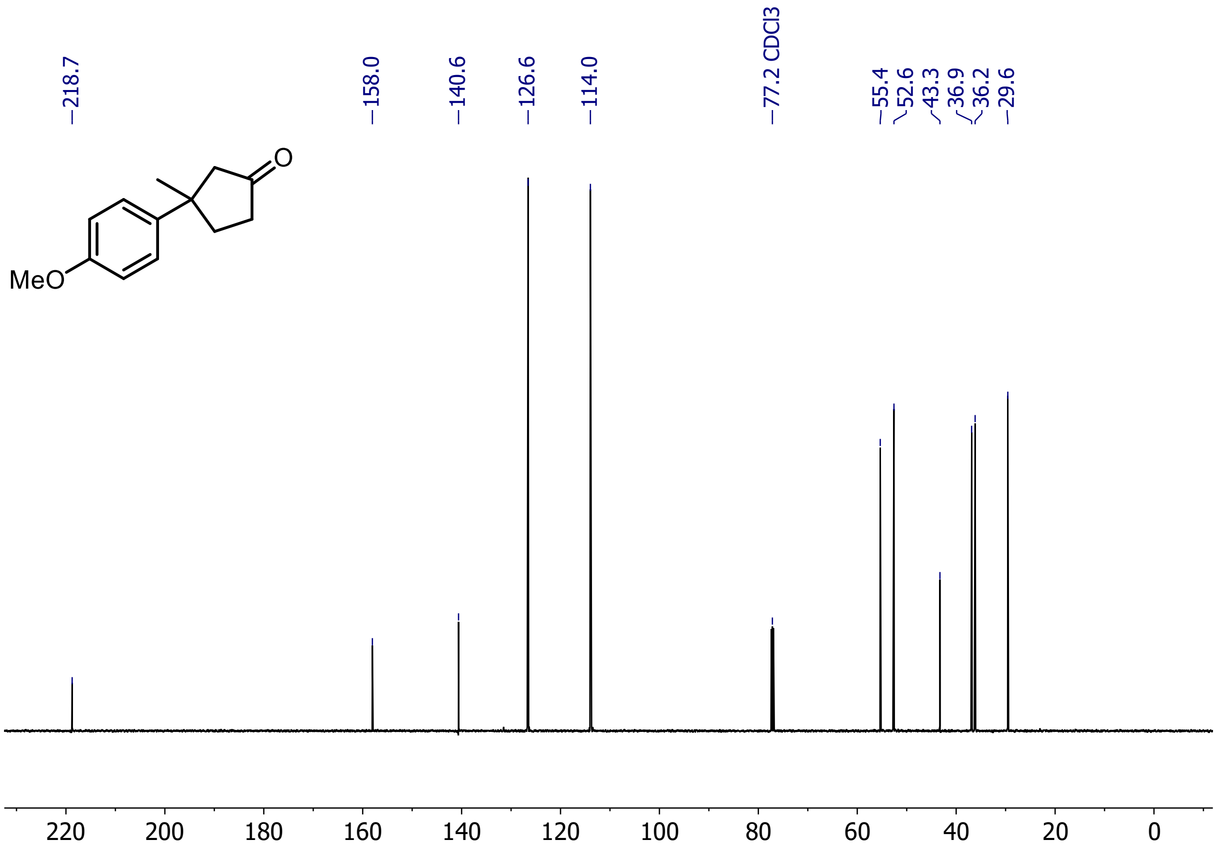

[2n], ^{1}H, $CDCl_3$, 500 MHz

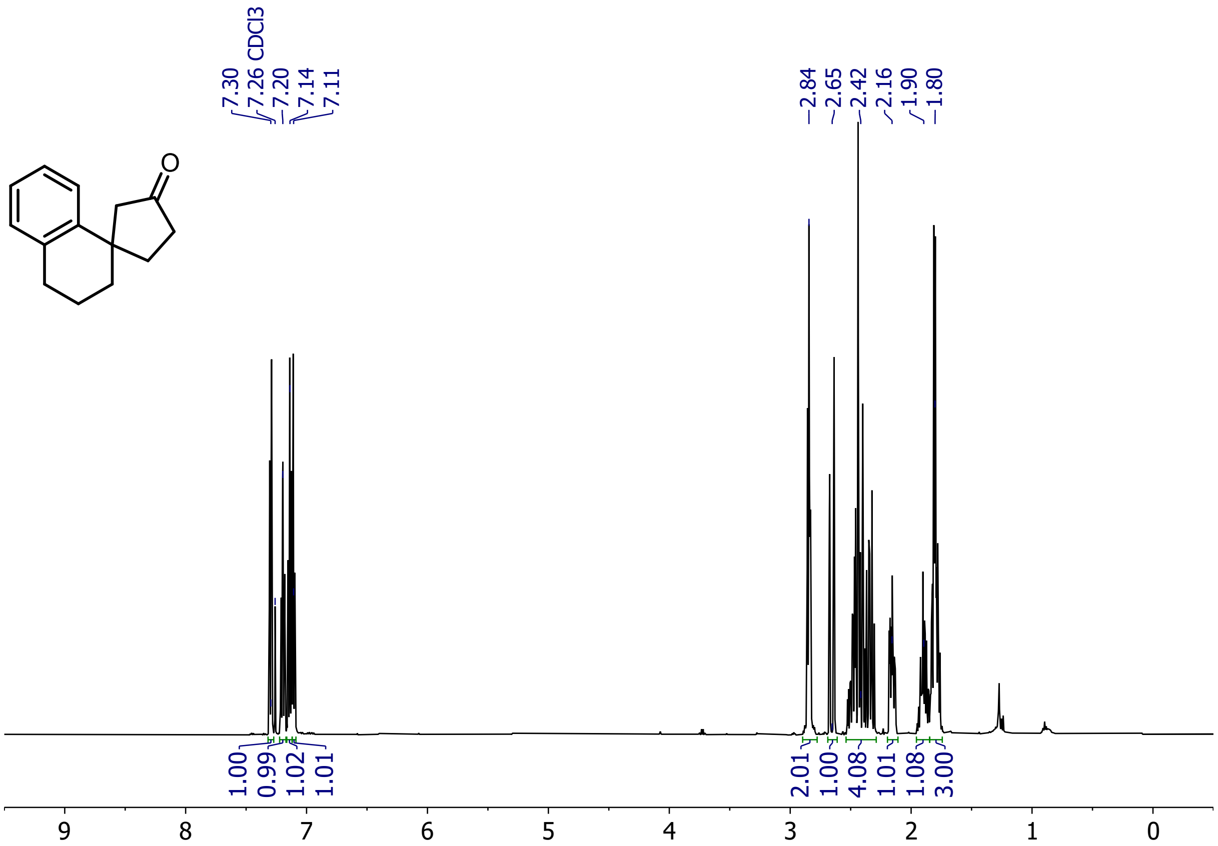

[2n], ^{13}C, $CDCl_3$, 126 MHz

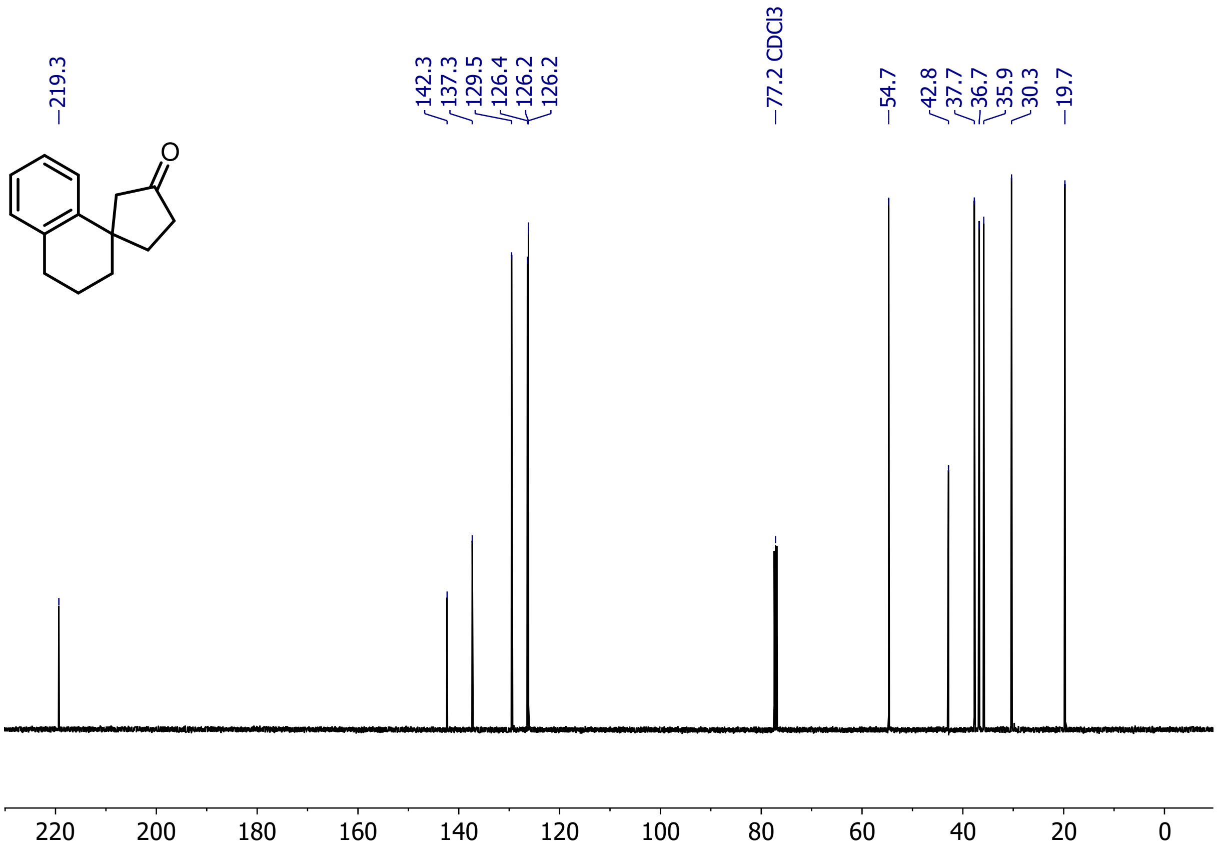

[2o], ^{1}H, $CDCl_3$, 599 MHz

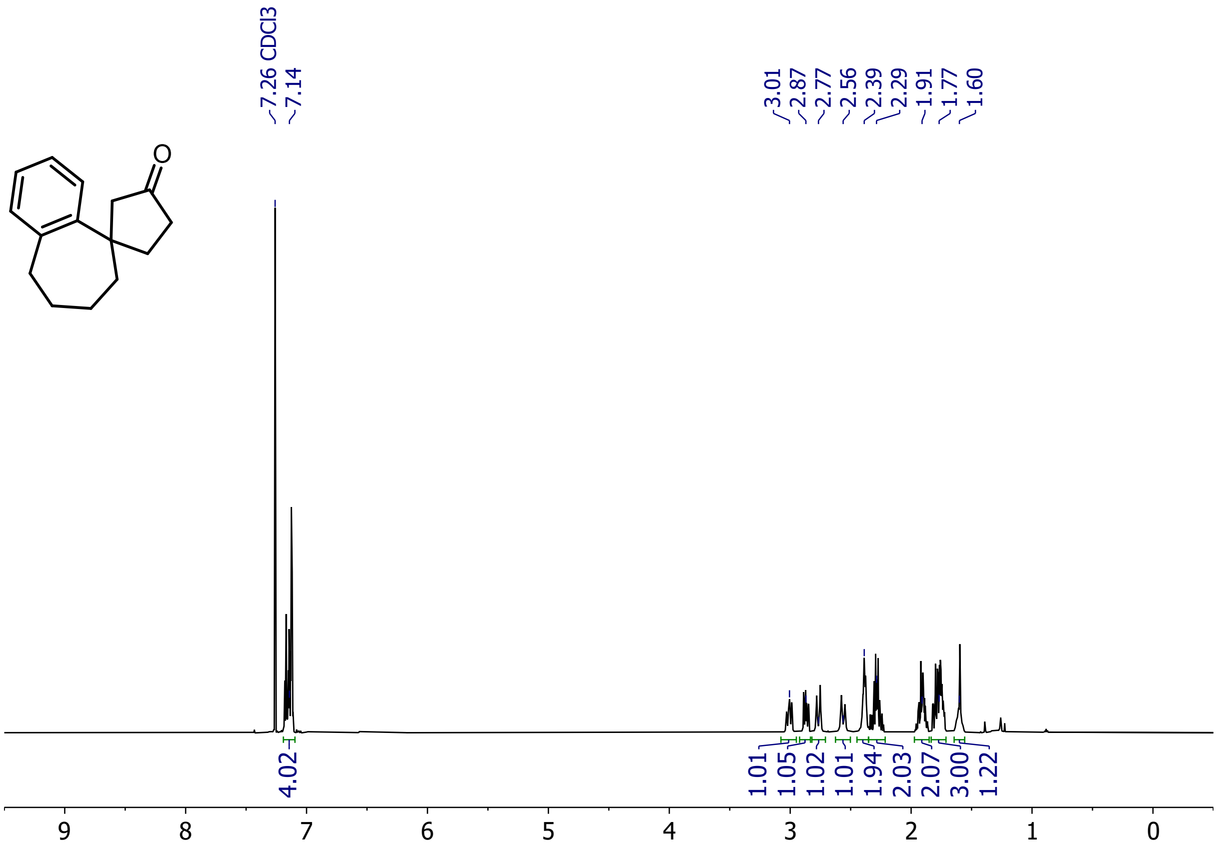

[2o], ^{13}C, $CDCl_3$, 151 MHz

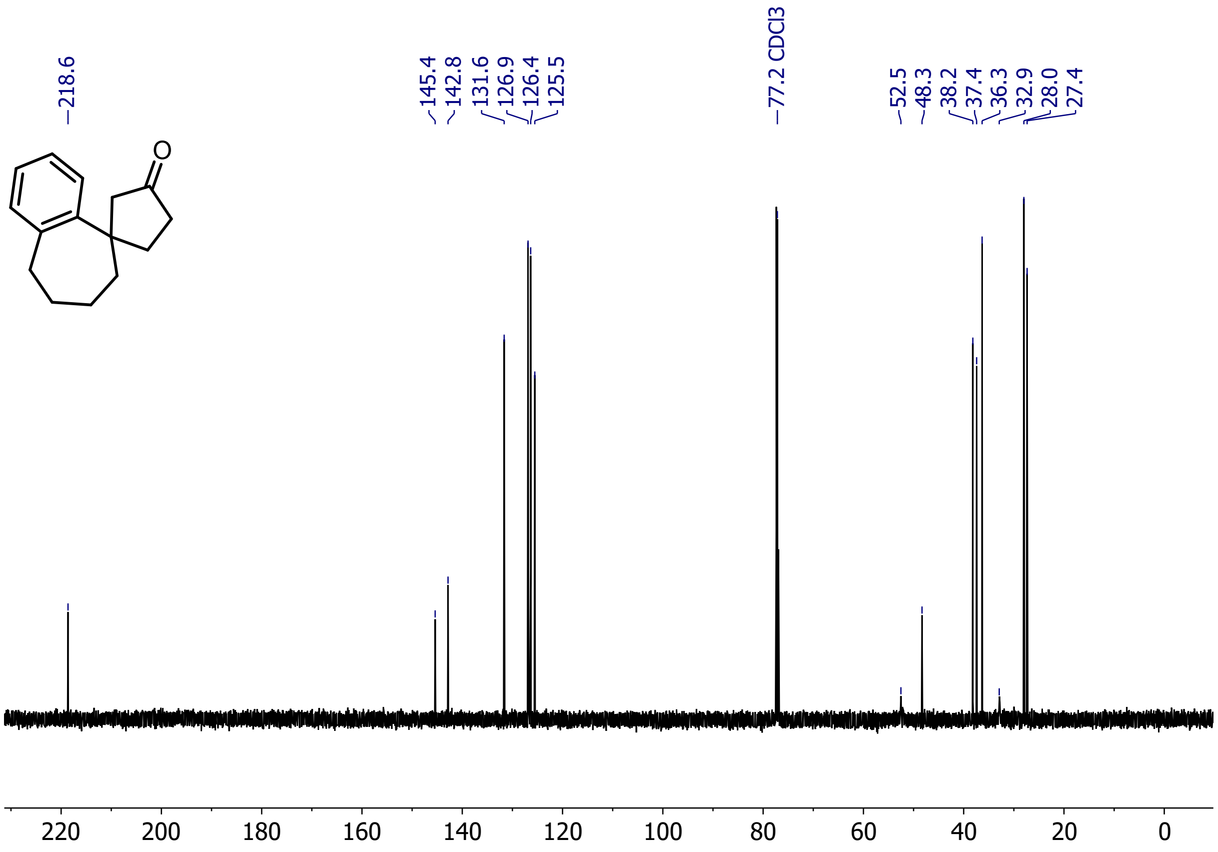

[2p], ^{1}H, $CDCl_3$, 599 MHz

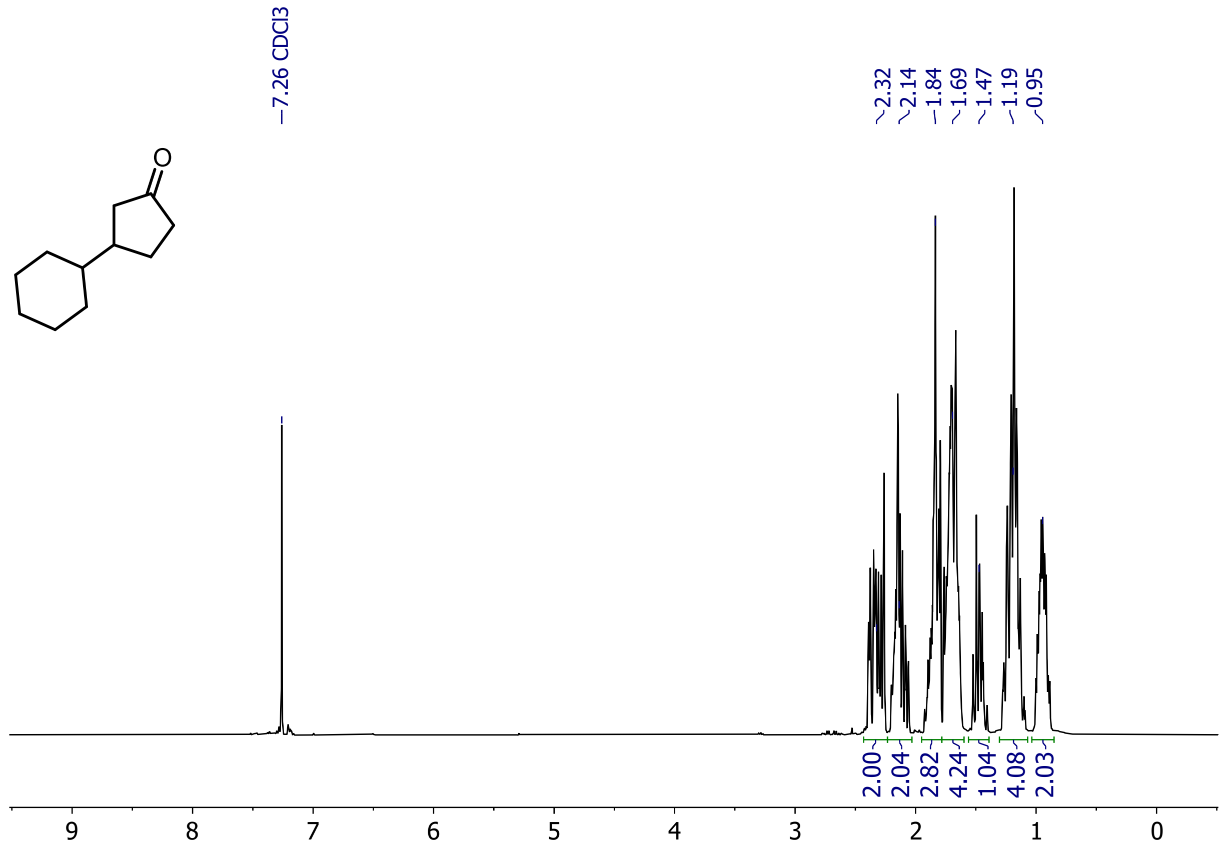

[2p], ^{13}C, $CDCl_3$, 151 MHz

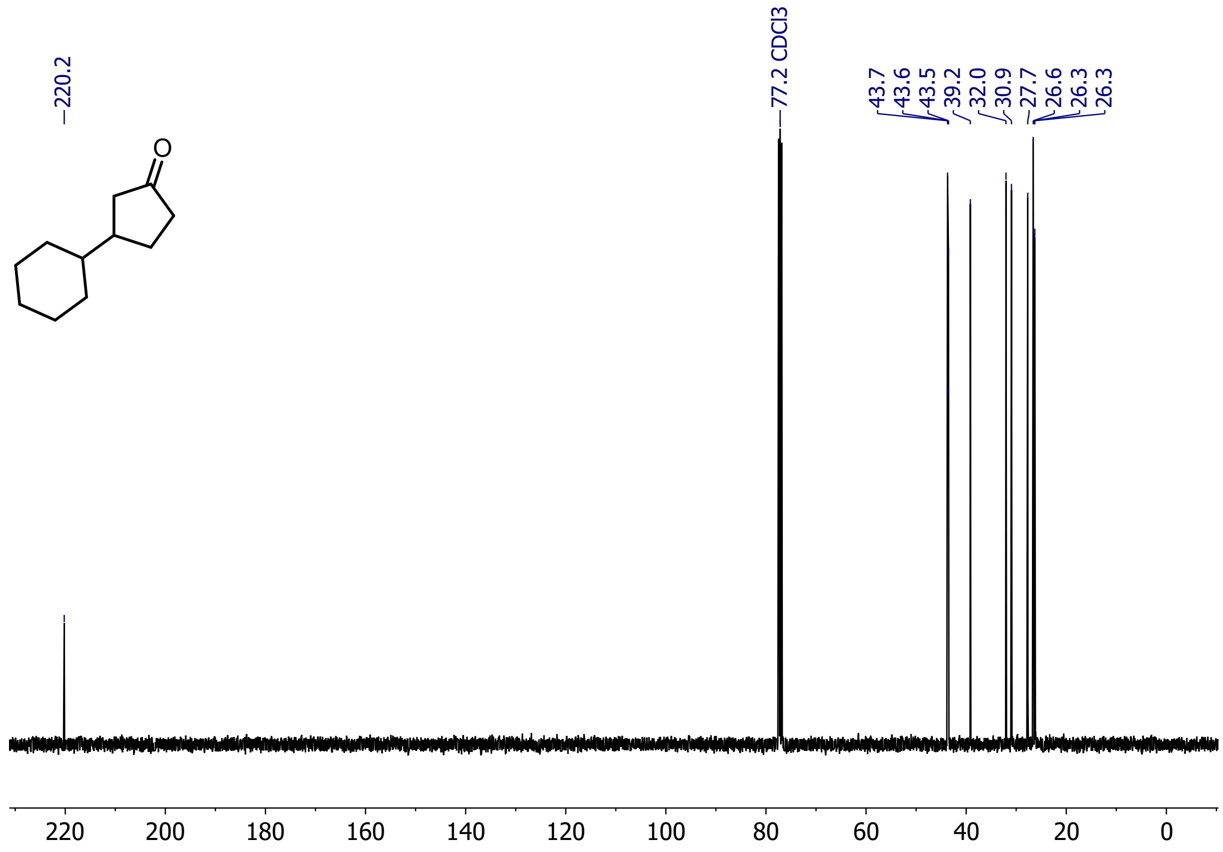

[2q], ^{1}H, $CDCl_3$, 600 MHz

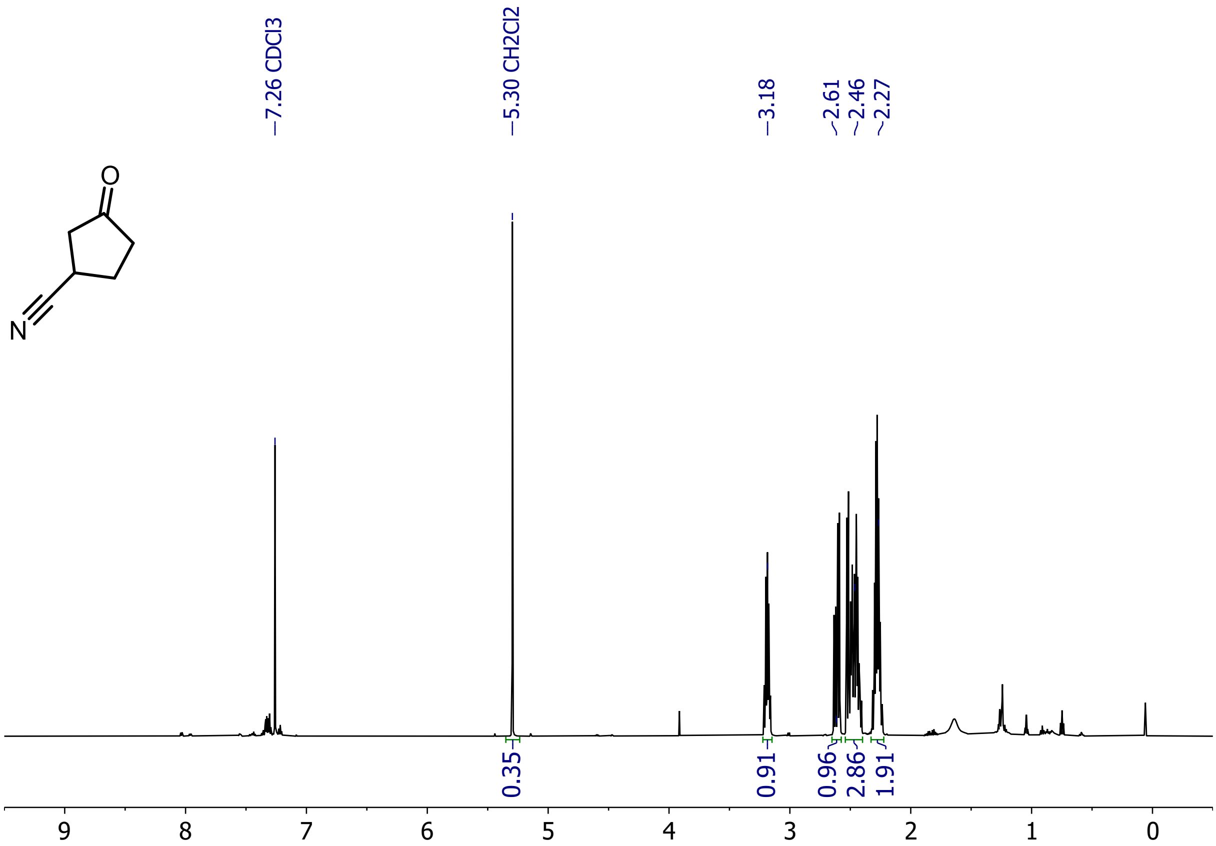

[2q], ^{13}C, $CDCl_3$, 151 MHz

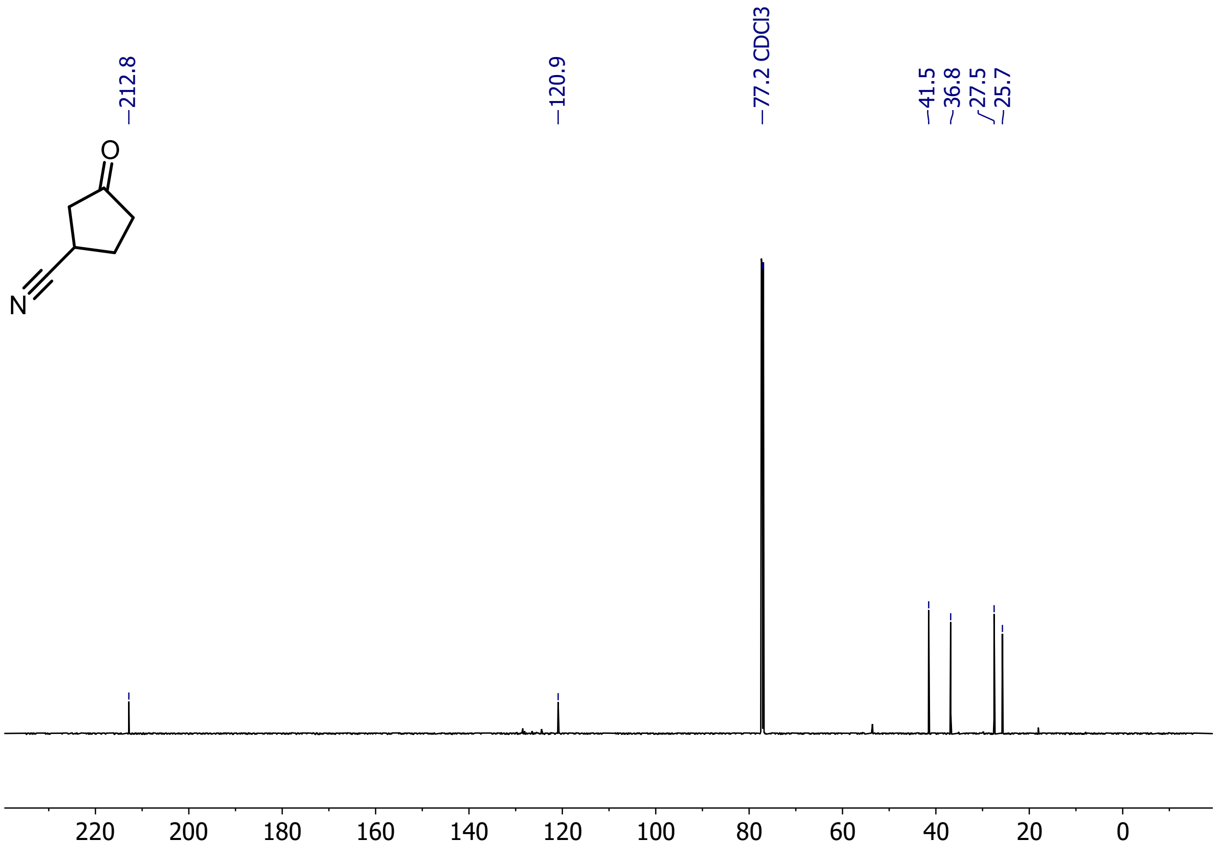

[2r], ^{1}H, $CDCl_3$, 400 MHz

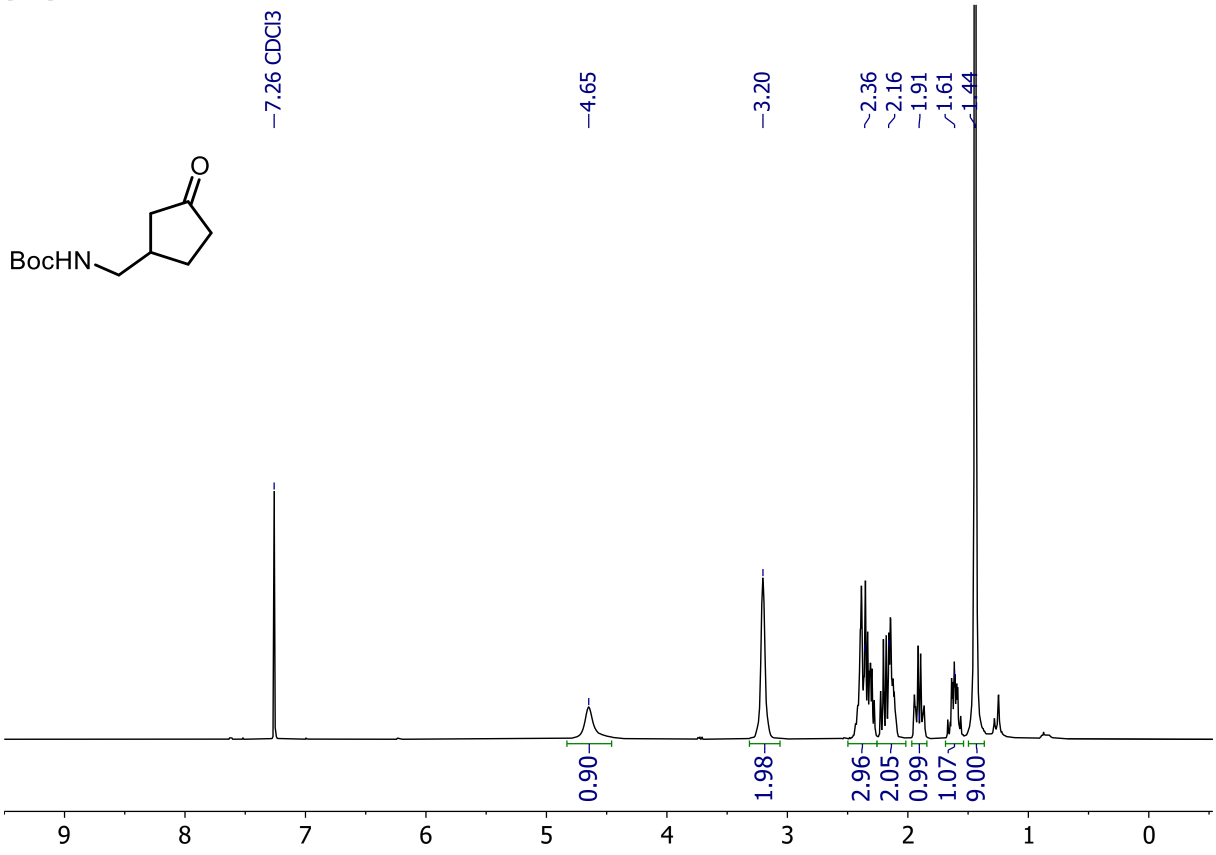

[2r], ^{13}C, $CDCl_3$, 101 MHz

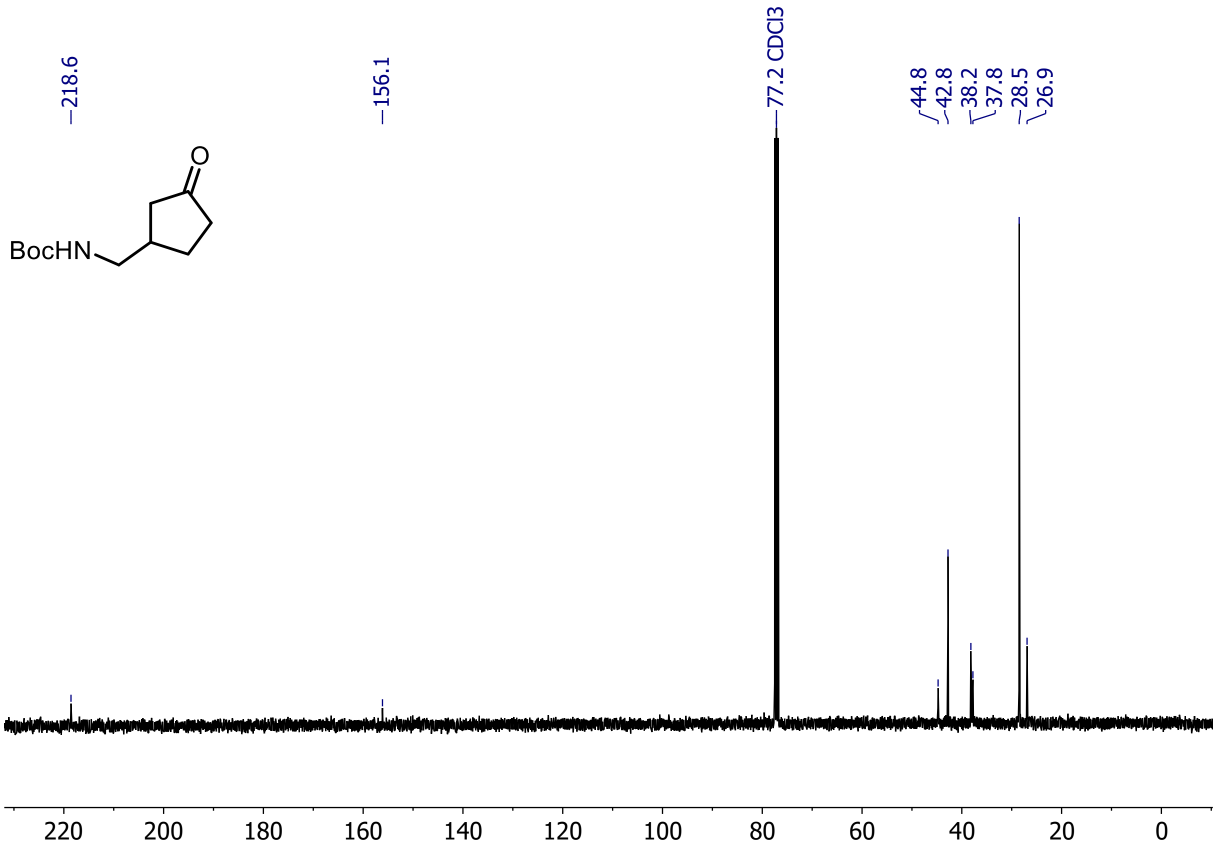

[2s], ^{1}H, $CDCl_3$, 400 MHz

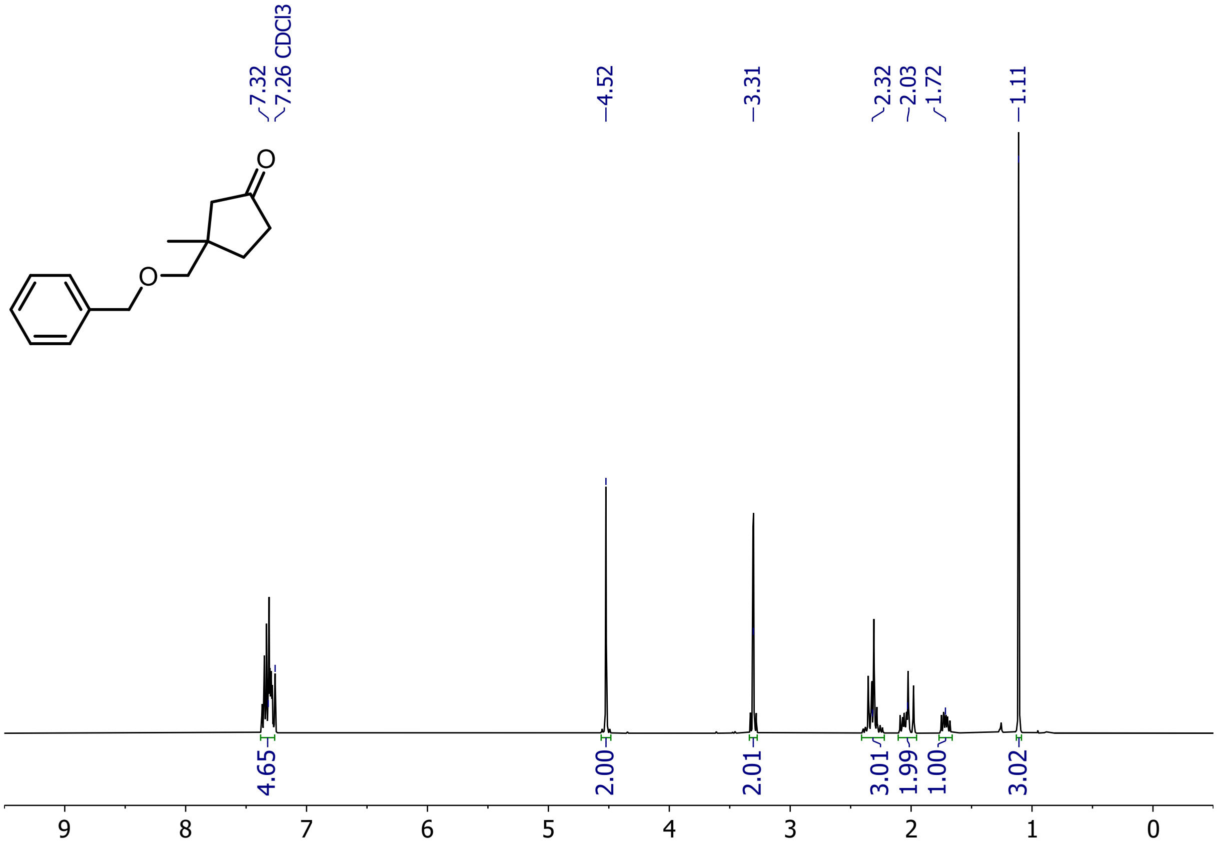

[2s], ^{13}C, $CDCl_3$, 101 MHz

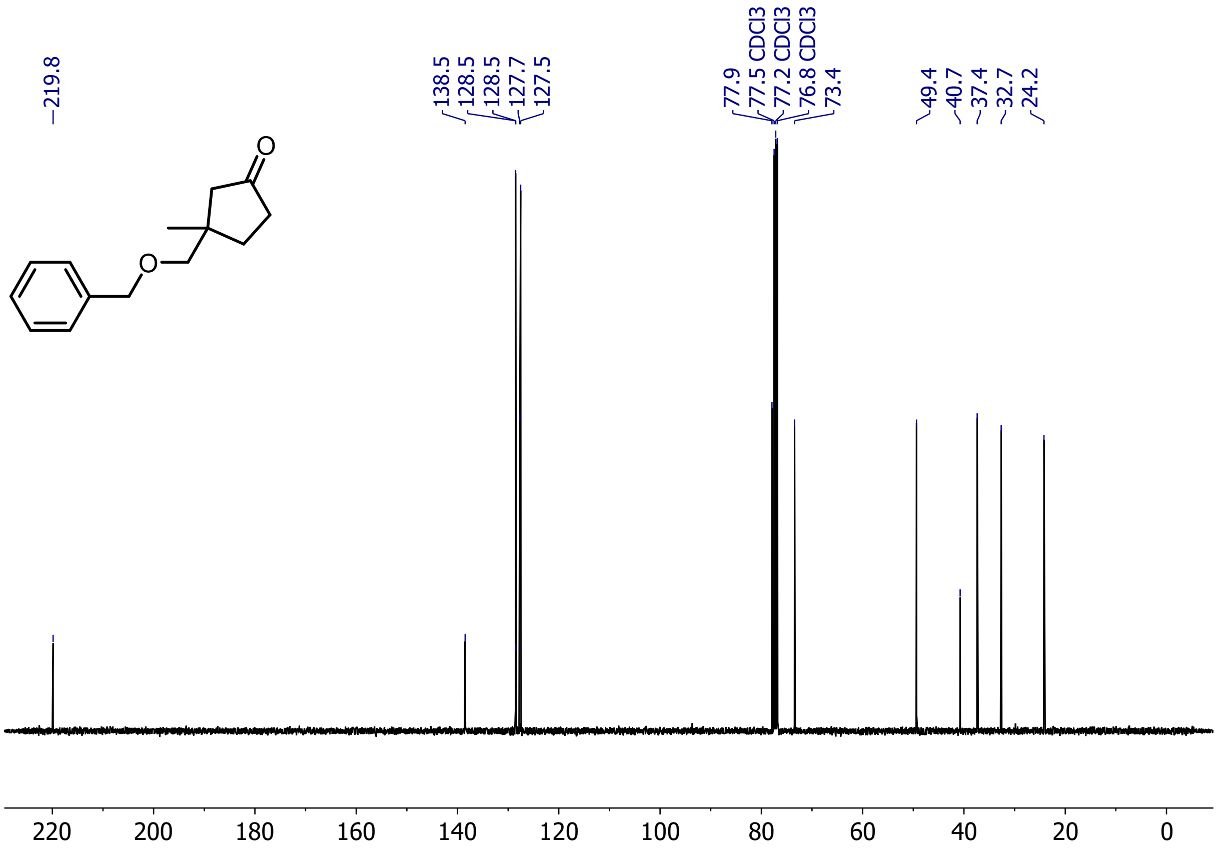

[2t], ^{1}H, $CDCl_3$, 400 MHz

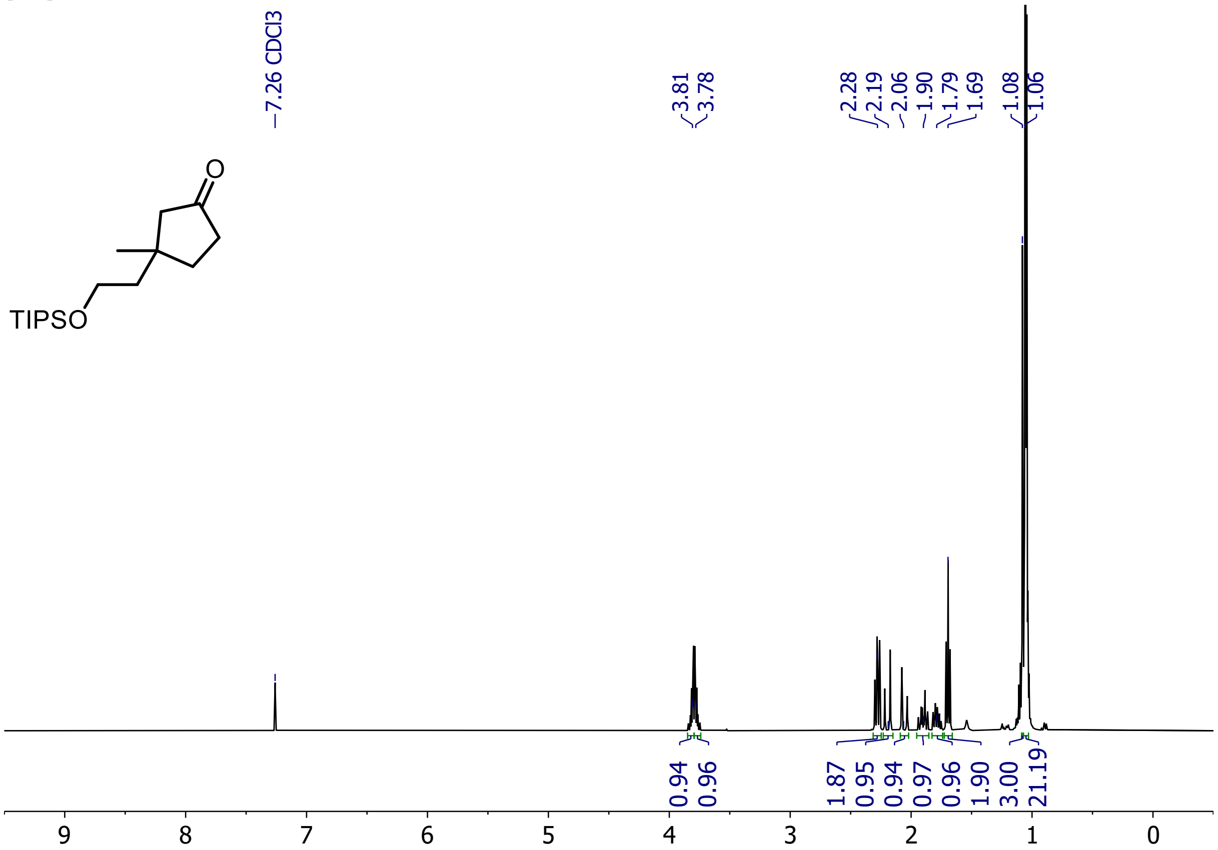

[2t], ^{13}C, $CDCl_3$, 101 MHz

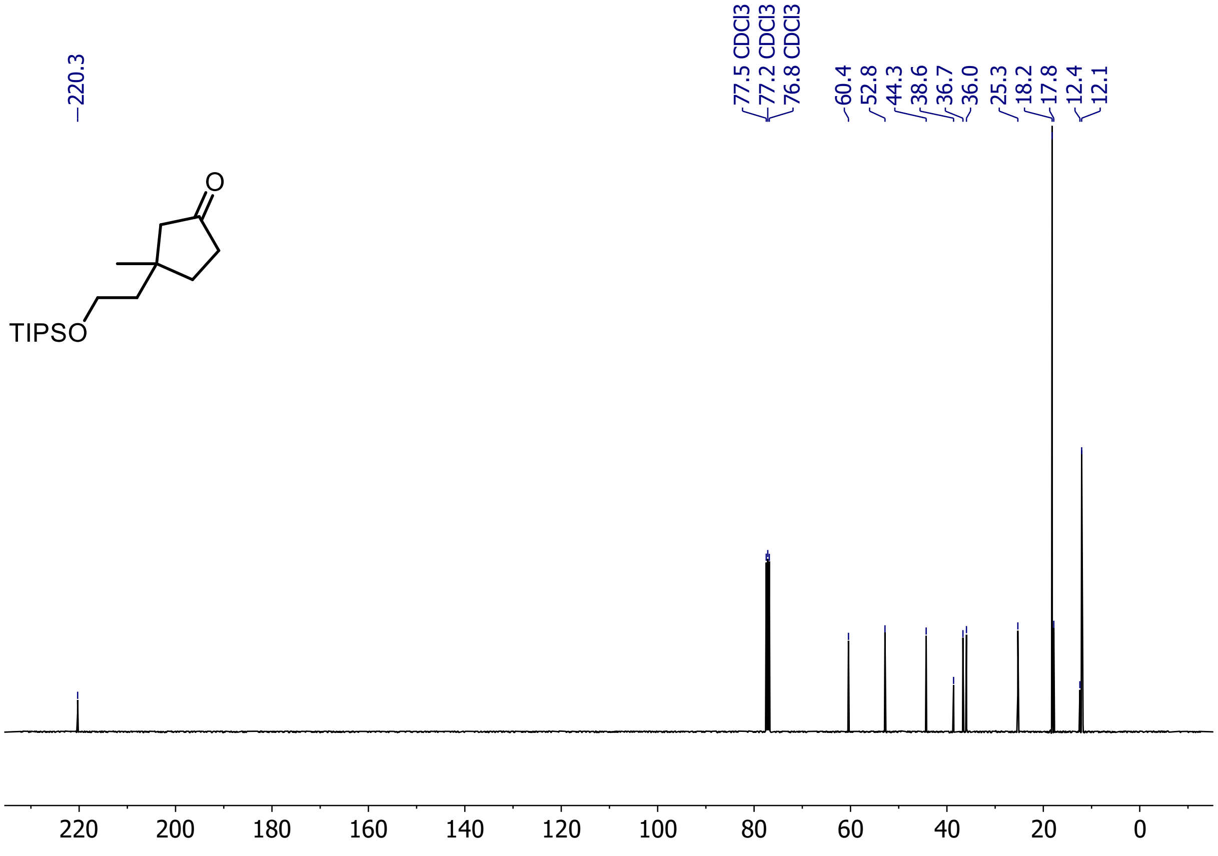

[2u], ^{1}H, $CDCl_3$, 400 MHz

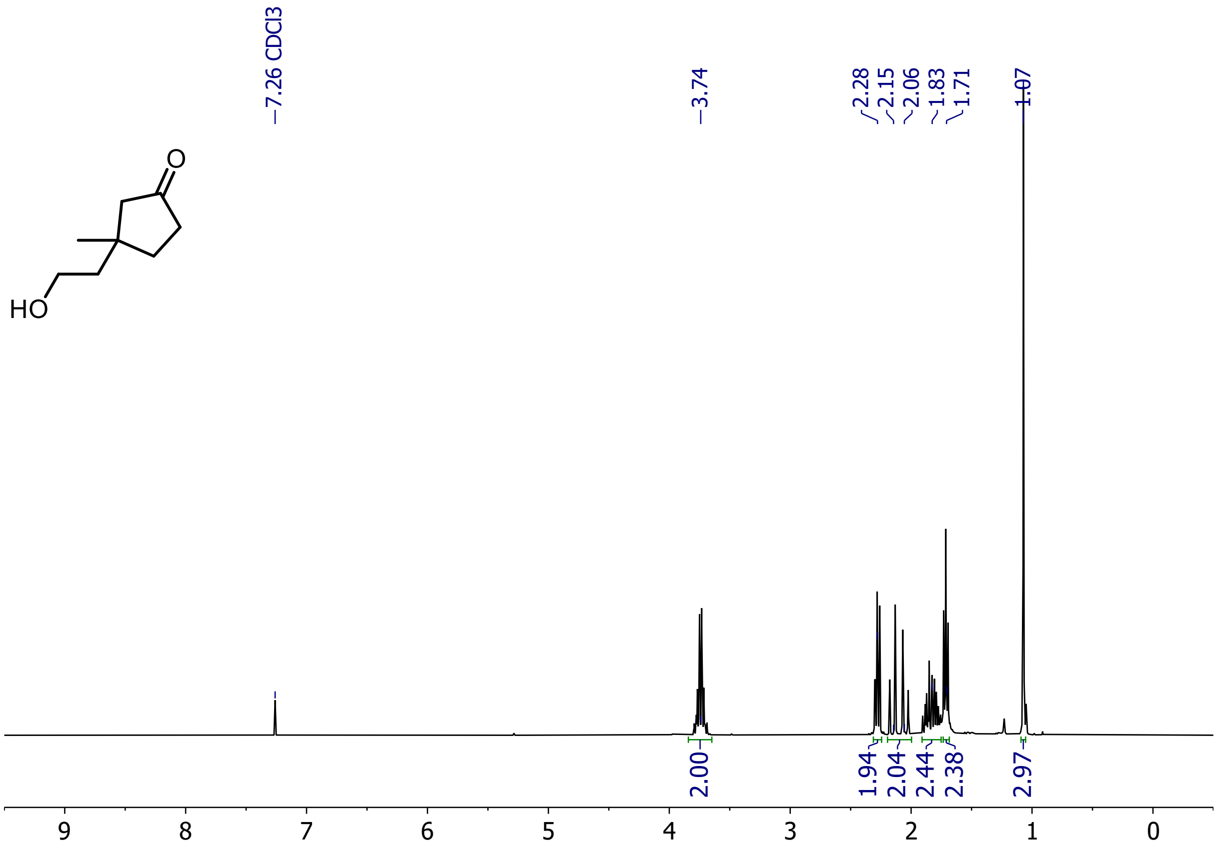

[2u], ^{13}C, $CDCl_3$, 101 MHz

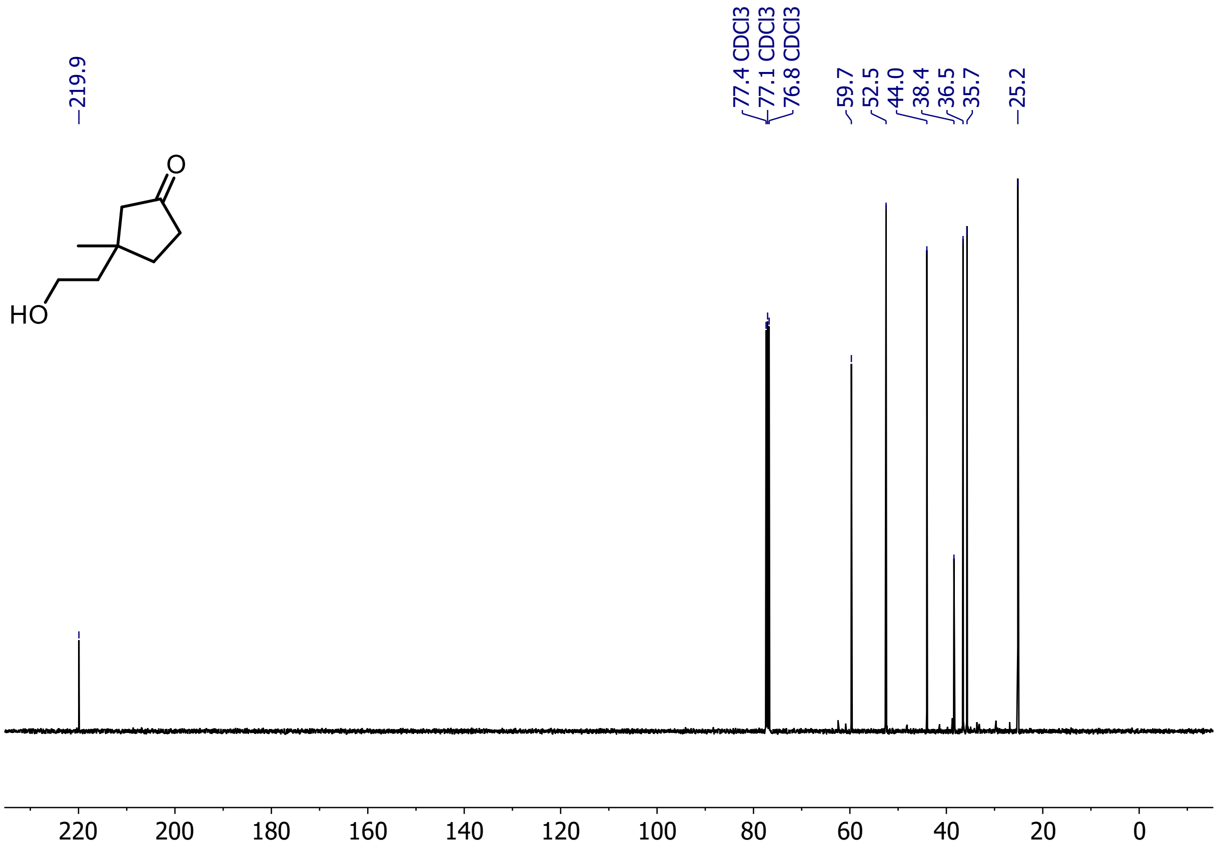

[2v], ^{1}H, $CDCl_3$, 400 MHz

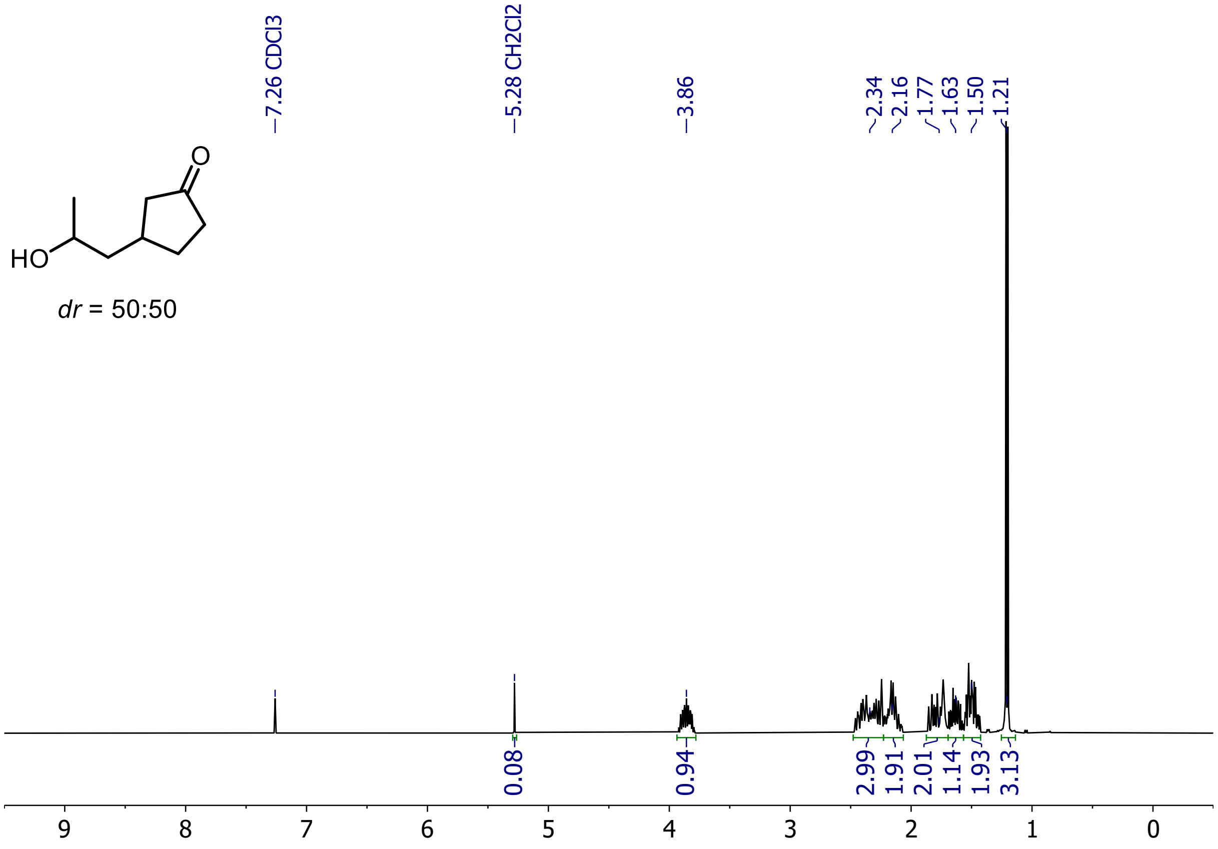

[2v], ^{13}C, $CDCl_3$, 101 MHz

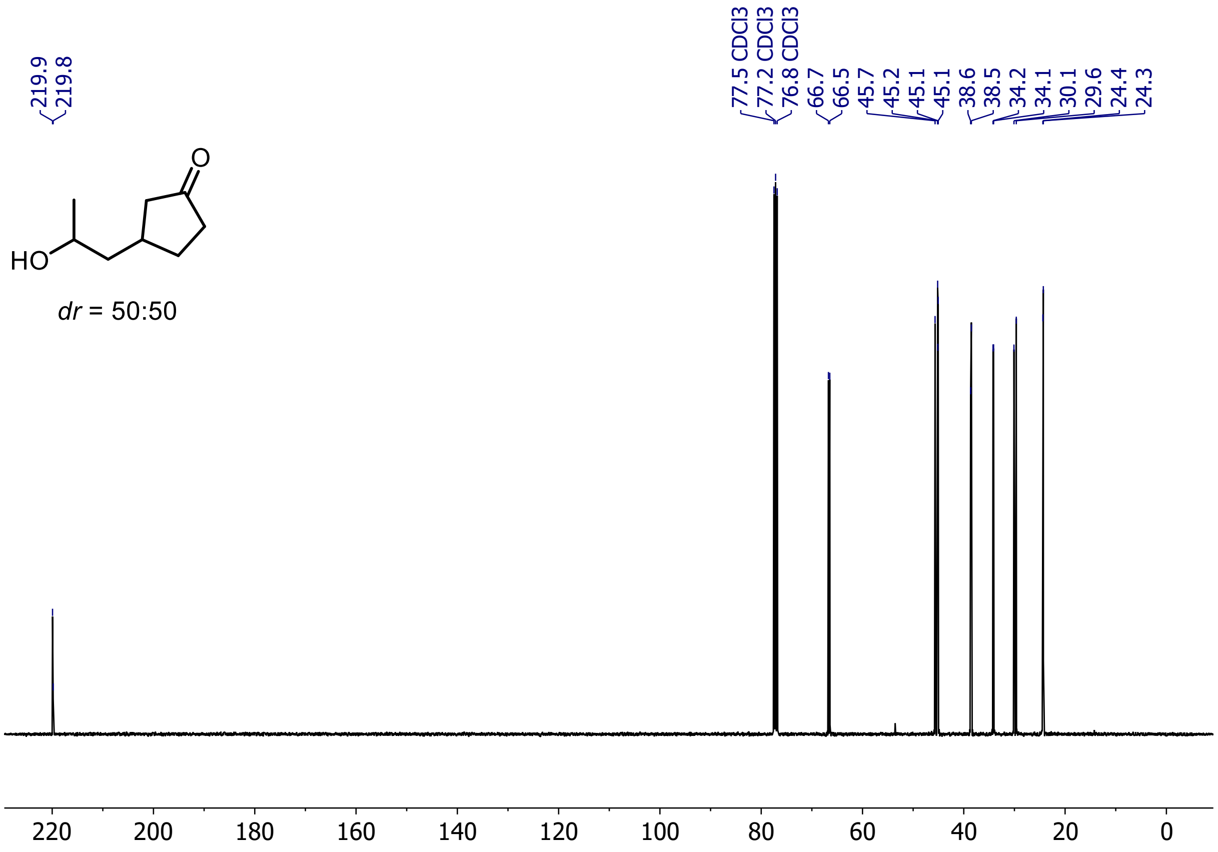

[2w], ^{1}H, $CDCl_3$, 400 MHz

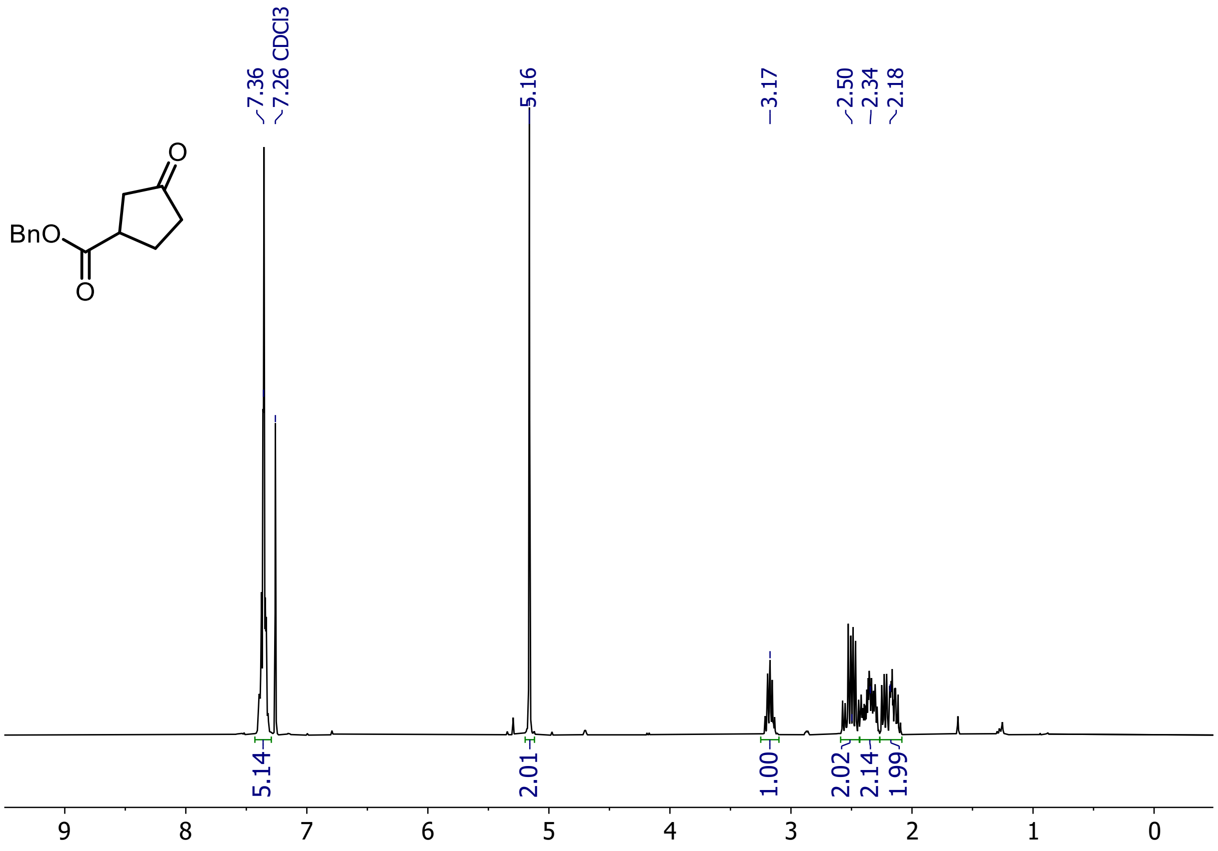

[2w], ^{13}C, $CDCl_3$, 101 MHz

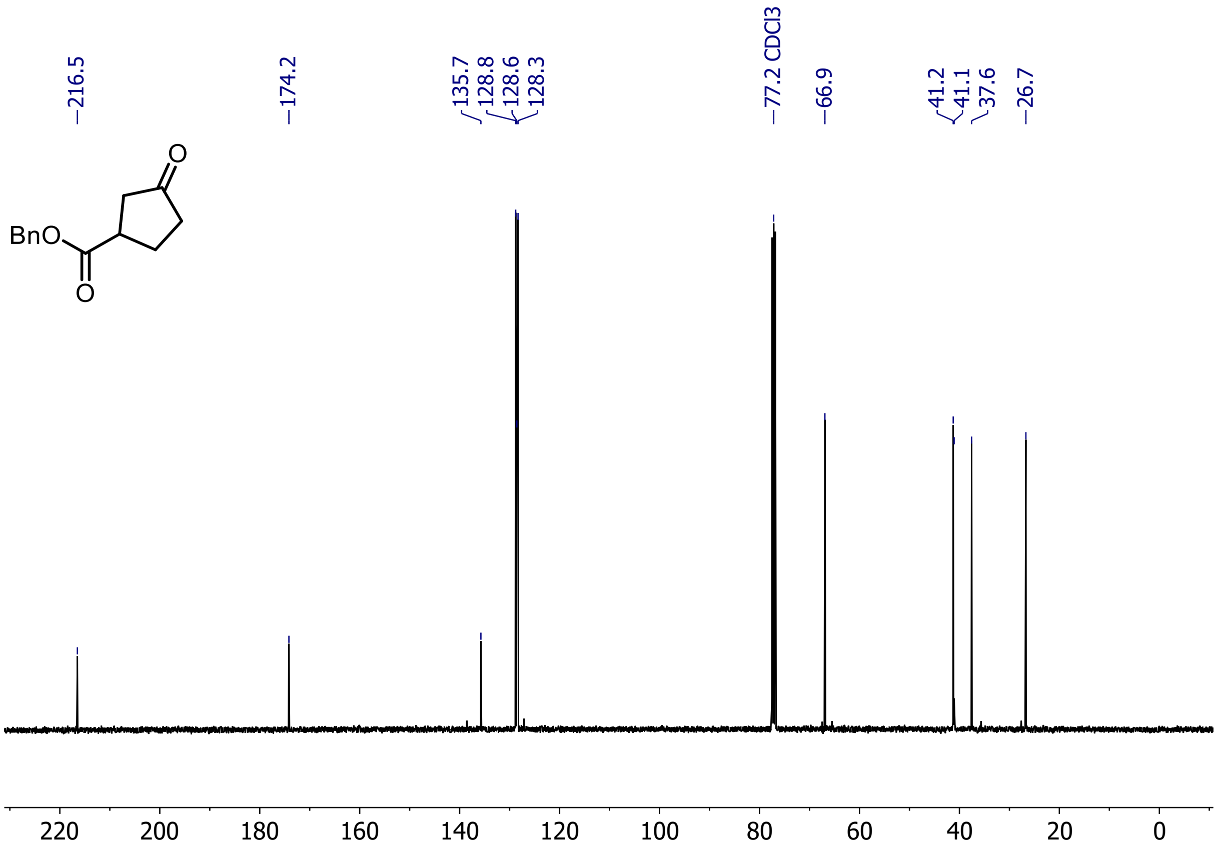

[2x], ^{1}H, $CDCl_3$, 599 MHz

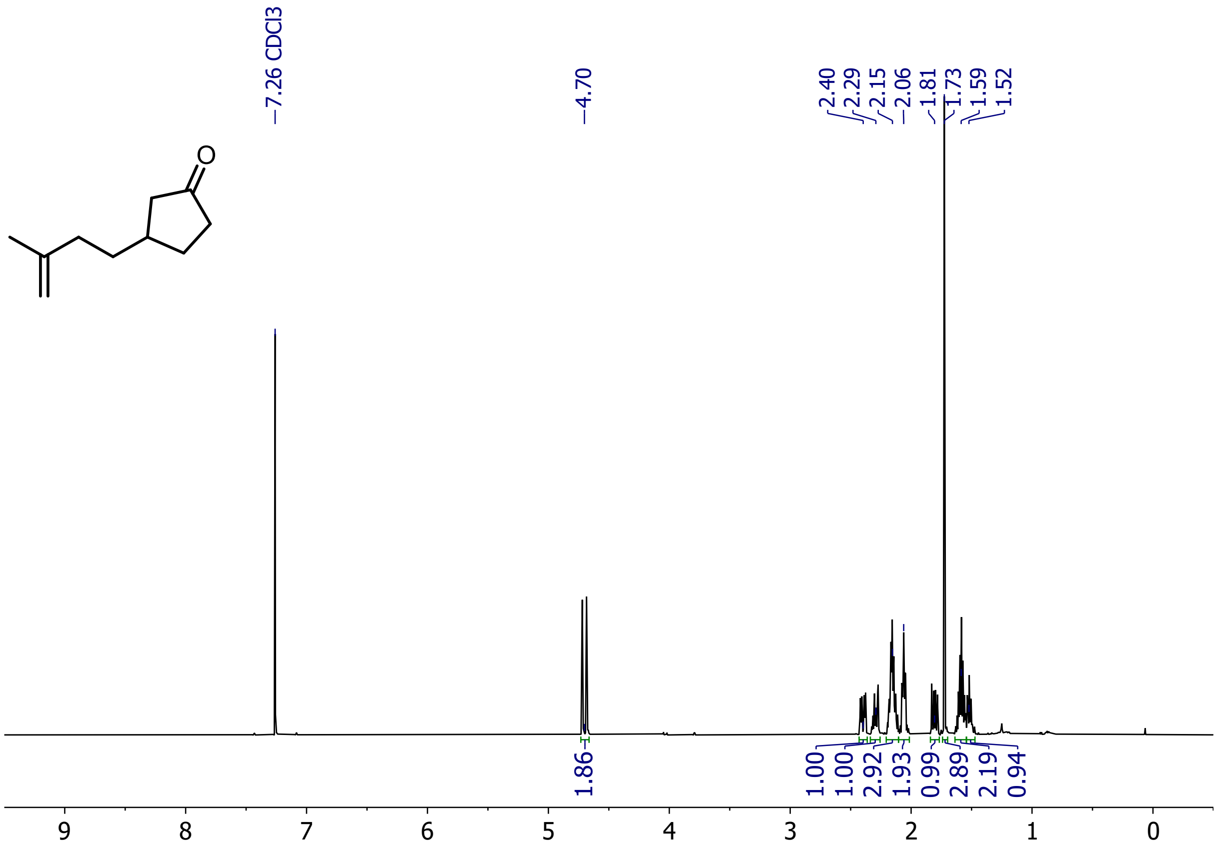

[2x], ^{13}C, $CDCl_3$, 151 MHz

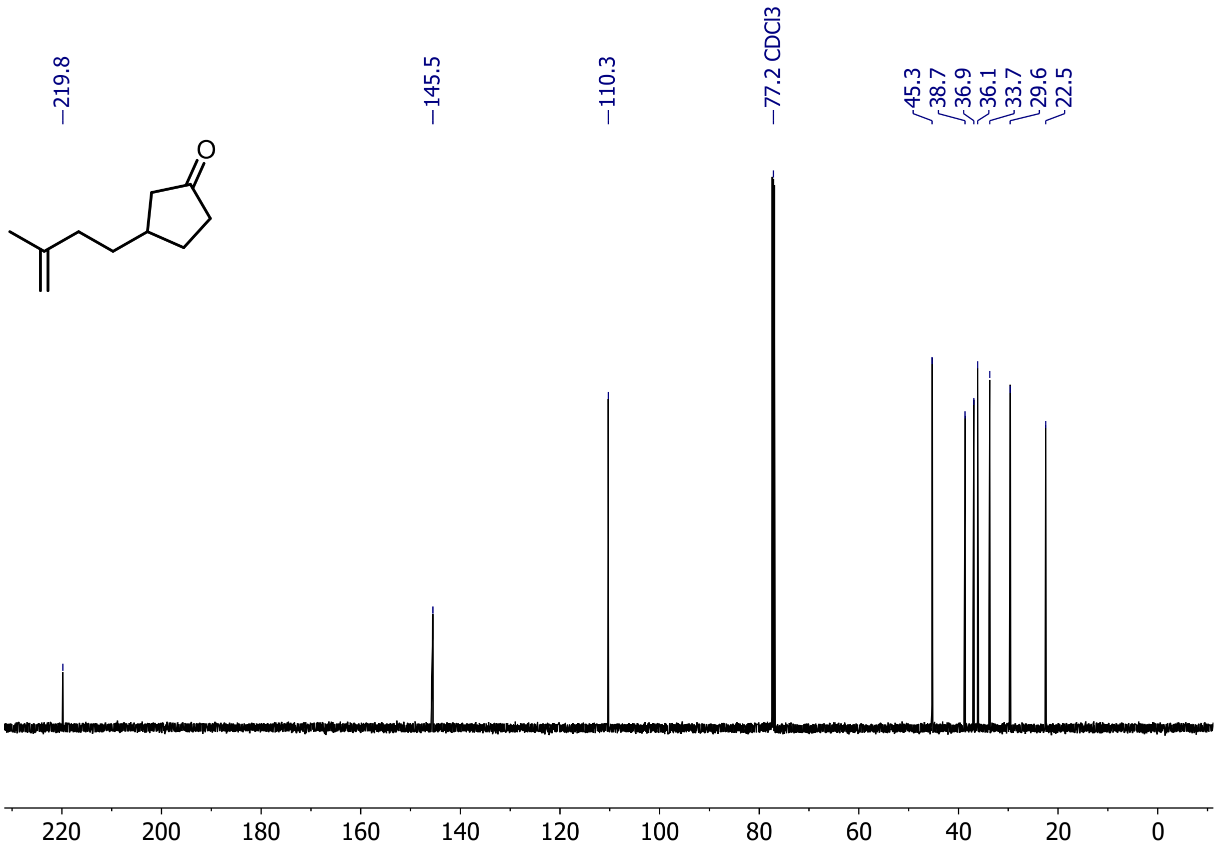

[6], ^{1}H, $CDCl_3$, 400 MHz

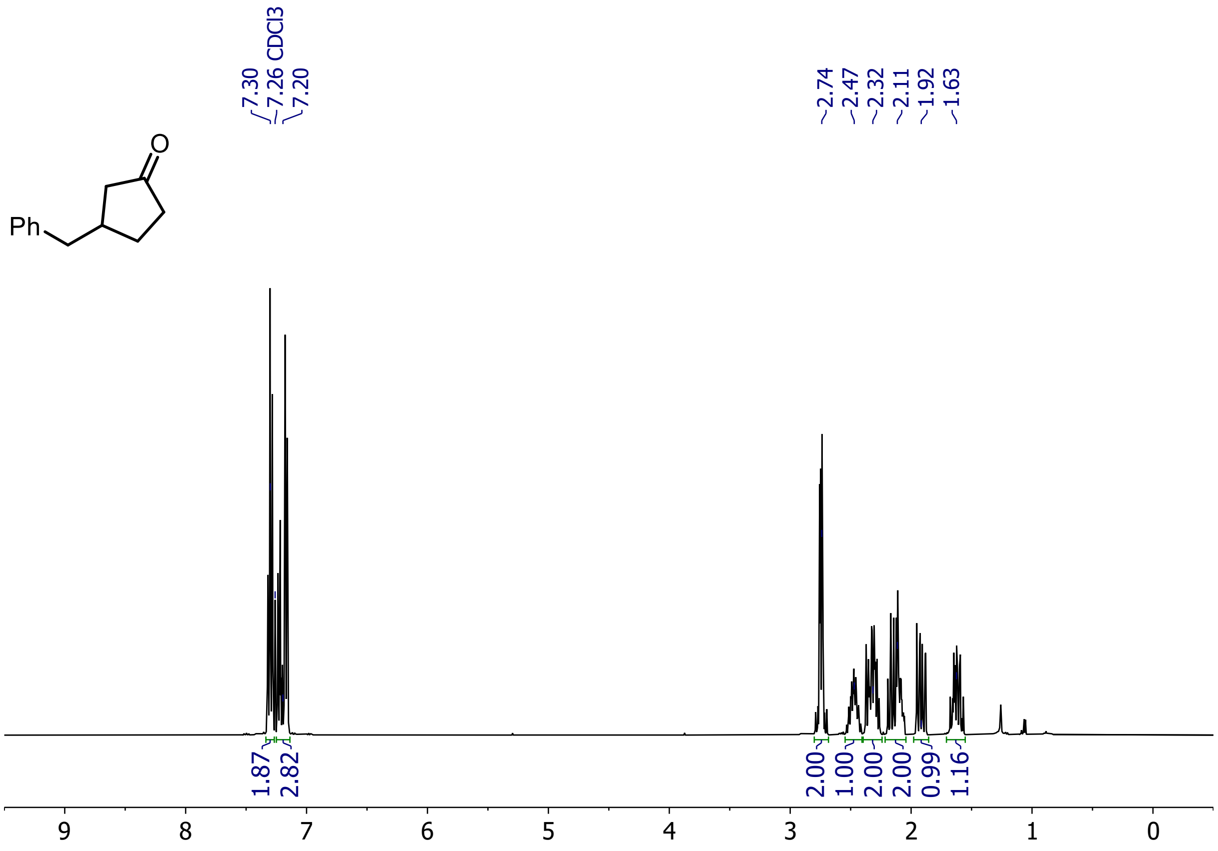

[6], ^{13}C, $CDCl_3$, 101 MHz

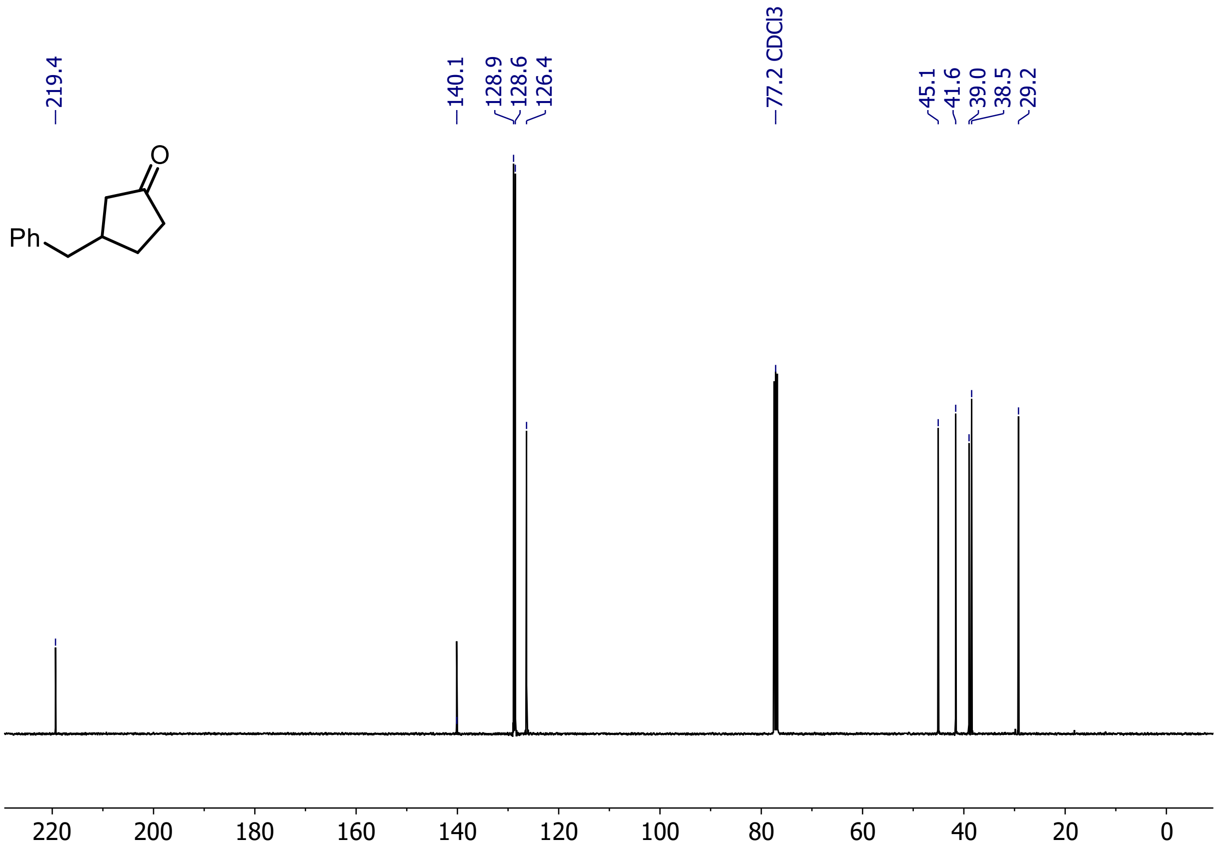

[7], ^{1}H, $CDCl_3$, 400 MHz

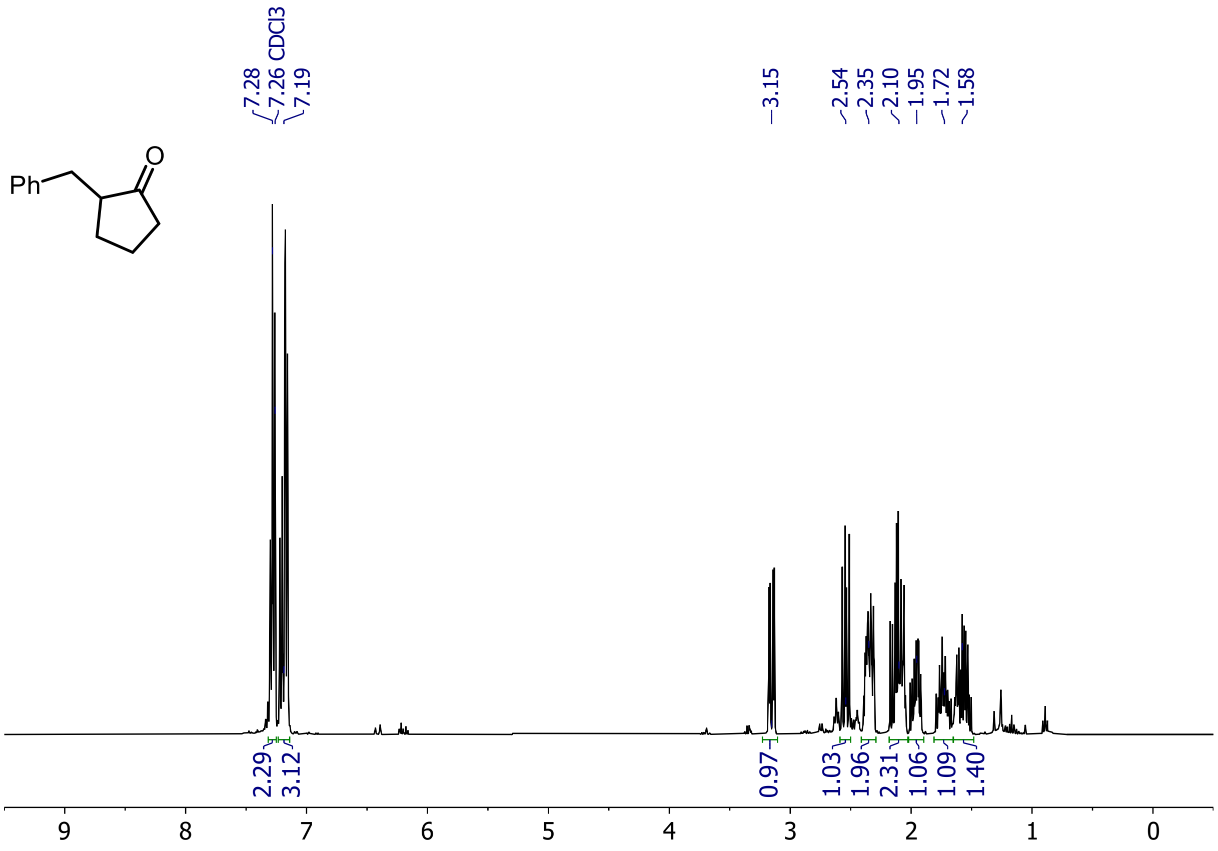

[7], ^{13}C, $CDCl_3$, 101 MHz

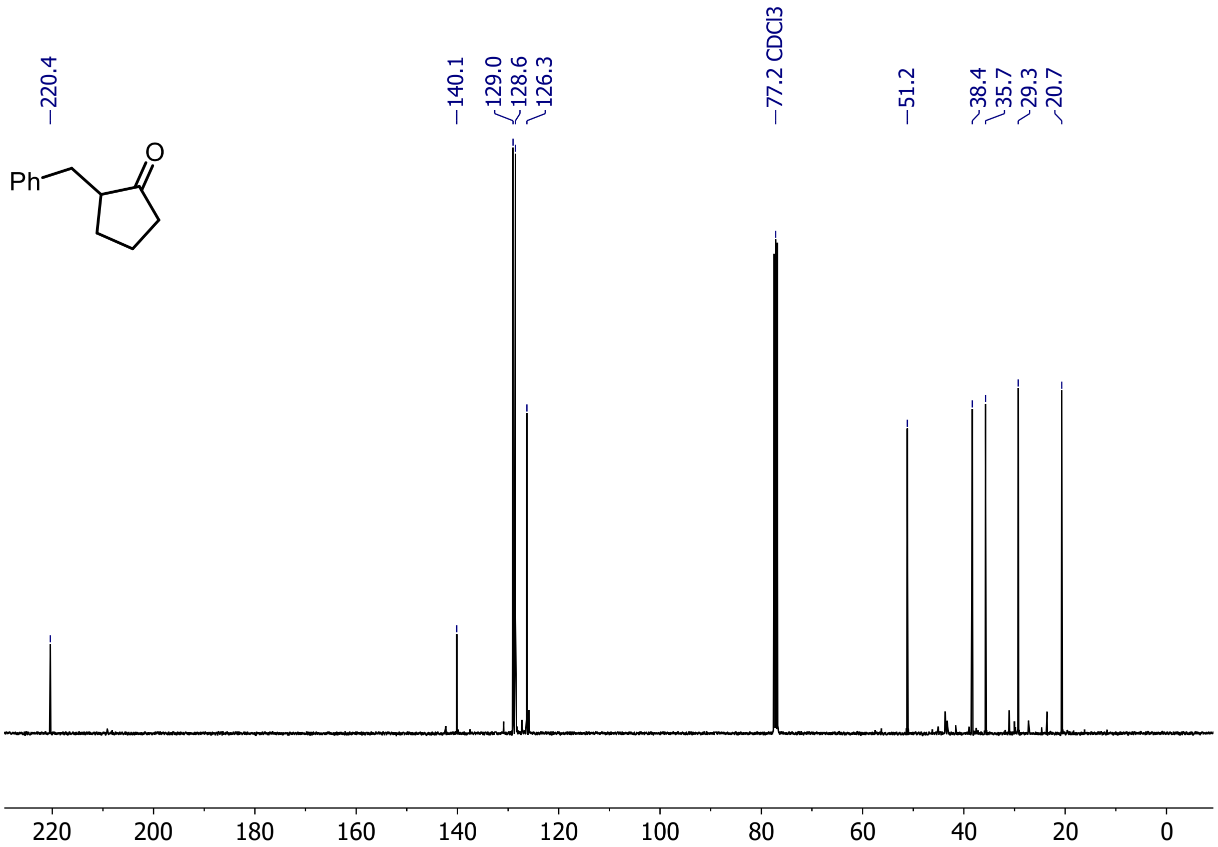

Supplement: Supplementary file 1 — Supporting Information [file ANIE-62-0-s001.pdf]
